# Switchable adhesion of phase-transition eutectogels with integrated machine learning-enhanced intelligent adhesion sensing

**DOI:** 10.1038/s41467-026-74275-7

**Published:** 2026-06-11

**Authors:** JiaQing He, JiaHao Li, HanYang Dong, DeYun Chen, ChangHong Linghu, Qiang Zhou, YinBo Zhu, ShuRong Sheng, HengAn Wu, Wei Feng

**Affiliations:** 1 Institute of Humanoid Robots, Department of Modern Mechanics, University of Science and Technology of China, Hefei, China; 2 Institute of Artificial Intelligence, Hefei Comprehensive National Science Center, Hefei, China; 3 Department of Mechanical Engineering, College of Engineering, City University of Hong Kong, Hong Kong, China; 4 Chemistry Experiment Teaching Center, School of Chemistry and Materials Science, University of Science and Technology of China, Hefei, China; 5 State Key Laboratory of Nonlinear Mechanics, University of Science and Technology of China, Hefei, China

**Keywords:** Gels and hydrogels, Gels and hydrogels, Polymers

## Abstract

Switchable adhesion underpins emerging technologies in robotics, microelectronics, and biomedical engineering. However, achieving switchable surface adhesion that can adapt to substrates with varying material compositions and surface roughness, while simultaneously enabling real-time and wireless monitoring of adhesion strength, poses a substantial challenge. Here, we present a eutectogel-based system that integrates electrothermally switchable adhesion with wireless sensing capability for in situ monitoring of adhesion forces. The switching mechanism is systematically elucidated through a combination of mechanical analysis and molecular-level characterization. The integration of machine-learning assisted adhesion sensing with dynamic gripping and locomotion enables safer and smarter robotic operation in adhesion joints, smart grippers and climbing robots. Demonstrations in adhesion-aware sensing, robotic grasping, and wall climbing validate the system’s practical utility, establishing a pathway toward next-generation intelligent adhesive interfaces that are both adaptive and self-perceptive.

## Abstract (web summary)

Switchable adhesion underpins robotics and wearable adhesion, though optimizing adhesive properties while monitoring adhesive properties is challenging. Here the authors describe a eutectogel switchable adhesive driven by electrothermal stimulus with adhesive property sensing capability.

## Introduction

Strong yet reversible adhesion is essential for many interfacial systems, including climbing robotics^1–6^, transfer printing^7,8^, human–machine interfaces^9,10^, and biomedical engineering^11^. In these applications, the interface must provide sufficient adhesion to support loads while also allowing rapid and controllable detachment. Achieving this capability on surfaces with diverse materials and roughness, however, remains a significant challenge. The fundamental difficulty lies in reconciling two competing requirements: strong interfacial contact that maximizes adhesion and efficient release that enables reversible operation. In addition, practical adhesive systems must adapt to surface irregularities and avoid leaving residues on contacted substrates^12^. These competing requirements make it difficult to realize switchable adhesion that is both strong and universally applicable.

Existing strategies for switchable adhesion mainly rely on three types of mechanisms. One approach uses structural control to regulate contact with the substrate. Examples include pneumatic or hydraulic actuation combined with gecko-inspired elastomeric microfibrillar adhesives^13–18^, interlocked structures^19^, electromagnetic adhesion for ferromagnetic surfaces^3^, dielectric elastomer actuators used in miniaturized climbing robots^20^, and hierarchical microstructures inspired by gecko footpads^21–24^. A second strategy utilizes phase transitions to modulate interfacial contact. Shape-memory polymers and liquid crystal elastomers can undergo reversible transitions between rubbery and glassy states^8,25–30^, enabling conformal contact with rough surfaces followed by detachment through changes in stiffness^31^. Similarly, crystal gels with reversible phase transitions can achieve strong adhesion by forming conformal contact in the soft state and maintaining adhesion in the glassy state^32–39^. The third approach involves switchable molecular interactions. For example, linear or weakly crosslinked polymers^40–43^ containing photoresponsive groups, such as azobenzene, can modulate adhesion through light-induced isomerization^44,45^, while cross-linked systems, such as thermo-responsive hydrogels, have been developed to reduce residue contamination during detachment^46,47^. Despite these advances, most existing systems rely predominantly on a single mechanism, either physical shape locking or reversible molecular interactions. As a result, their performance often becomes limited when applied to adherends with diverse surface chemistry and roughness.

Besides switchable adhesion, another important feature of biological adhesive systems is the integration of adhesion with sensing and regulation. Many organisms achieve stable locomotion by coupling adhesive contact with sensory feedback and muscular control^48,49^. Inspired by this concept, Wang, Li, and coworkers demonstrated integrated sensing and switchable adhesion during gripping by combining capacitive force sensors with stiffness-tunable dry adhesives or micro-adhesive structures^48,50^. Nevertheless, most artificial adhesive systems still treat adhesion and sensing as separate components. The lack of intrinsic sensing capability makes it difficult to monitor the interfacial contact state in real time, which is particularly critical for applications, such as climbing robots, where unreliable attachment may lead to catastrophic failure. Developing adhesive materials that can simultaneously provide strong, switchable adhesion and intrinsic sensing functionality, therefore, remains an important challenge.

Here, we address these challenges by developing crystallizable eutectogels that integrate electrothermally switchable adhesion with intrinsic electrical sensing. Eutectogels are gels composed of a polymer network containing deep eutectic solvents (DES)^51–53^. These materials are typically conductive and exhibit reversible phase transitions. In our system, the crystallization–melting transition of DES enables a synergistic mechanism that combines physical shape locking with switchable molecular interactions at the interface, allowing strong and reversible adhesion across diverse surfaces. Upon electrothermal activation, the gel rapidly transitions from a crystalline state (10^1^ kPa) to a molten state (10^2^ kPa). During the M2C cooling process, the adhesion strength can be tuned between 10^2^ and 10^3^ kPa, which is sufficient to support robotic climbing on vertical surfaces with varied materials and roughness. The switching mechanism is further elucidated through systematic investigations of the crystalline, molten, and intermediate states using experimental characterization and molecular simulations. Importantly, the intrinsic conductivity of eutectogels enables real-time monitoring of adhesion states through electrical parameters, such as resistance and capacitance, which can be wirelessly transmitted via Bluetooth. Machine learning algorithms are further employed to predict adhesion states from electrical signals, enhancing system intelligence and reliability. The deep eutectic solvents used in this work are also environmentally friendly. The ability of eutectogels to achieve conformal contact in the molten state enables robust and reversible adhesion on non-specific surfaces, highlighting their potential for intelligent soft robotic systems and adaptive structural applications.

## Results

### Design principles

**Fig. 1:**
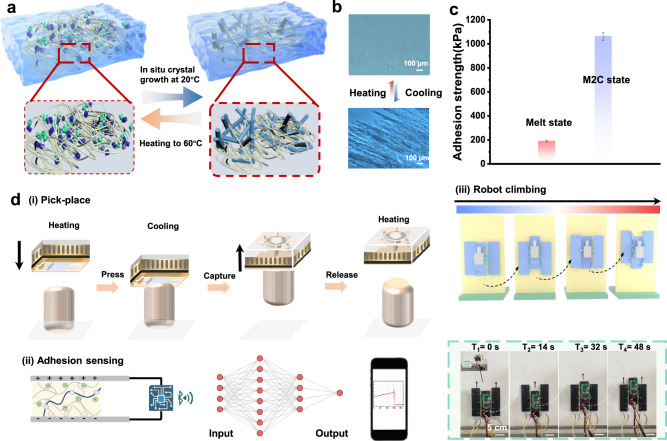
Overview of the characteristics and applications of the eutectogel. **a** Schematic illustration of the temperature-mediated crystallization–melting phase transition of the eutectogel. **b** Microscopic images showing reversible crystallization and melting processes inside the eutectogel. **c** Switchable adhesion performance of the eutectogel, exhibiting much stronger adhesion in the melt-to-crystallization (M2C) state compared with the melting state. Data are presented as mean ± SD (*n* = 3 independent samples). **d** Demonstrations and functionality of the self-sensing and switchable adhesive eutectogel. i Pick-and-place operation of an object through heating and cooling. ii Intelligent sensing of adhesive state enabled by electrical signals and machine learning. iii A climbing robot utilizing the switchable adhesion of the eutectogel. Scale bars, 5 cm.

To realize switchable adhesion across diverse surfaces, we investigate the adhesive behavior of crystallizable eutectogels. Eutectogels are polymer networks formed from DES and monomers (Supplementary Fig. 1). Owing to strong molecular interactions among the components (e.g., hydrogen bonding), the monomer-DES mixture exhibits a lower melting point than its individual constituents^54^. Except for the case of U:C = 2:1, the eutectic solvents are crystal at room temperature with melting point larger than 20 °C. By carefully tuning the DES composition, the melting point can be tailored to the desired temperature. Figure 1 illustrates the temperature-responsive phase transition of eutectogels, along with their composition and the working principle of a wall-climbing robot constructed using this material. The phase transition originates from the reversible crystallization and melting of the DES solvent within the eutectogel. As the temperature varies, the eutectogel switches between a rigid crystalline state and a flexible amorphous state (Fig. 1a), driven by the crystallization dynamics of the DES, as evidenced by the needle-like microstructures (Fig. 1b). This phase transition induces a marked change in adhesion forces, analogous to that of ice. Specifically, the adhesion strength increases from ~100 kPa in the melted state to ~1 MPa in the melt-to-crystalline (M2C) state (Fig. 1c).

The enhanced adhesion in the crystalline state arises from the synergistic effect of interfacial molecular interactions (hydrogen bonding, van der Waals forces, etc.) and physical interlocking between the gel and substrate. We will elaborate on these mechanisms in the following sections. The reversible switch of adhesion empowers eutectogels with versatile functionalities, including on-demand pick-and-release of objects (Fig. 1d-i), adhesion sensing (Fig. 1d-ii), and wall-climbing robotics (Fig. 1d-iii).

### Fabrication of eutectogels and investigation of the mechanical properties

The eutectogels were fabricated by precisely tuning the molar ratio of eutectic solvents and incorporating hydrophilic N-hydroxyethyl acrylamide (HEAA) with an amphiphilic quaternary ammonium monomer (OMA) (Supplementary Fig. 2). Their temperature-induced phase transition arises from the solid–liquid transition of the eutectic solvent. In this work, urea and choline chloride are selected as a representative hydrogen bond donor-acceptor pair. The melt point of DES can be altered by changing the ratio of DES components (Supplementary Fig. 1c). Polarized light microscopy confirms that the entire solid-liquid transition is a crystallization process of the eutectic solvent (Supplementary Fig. 3 and Supplementary Movie 1). The crystal transition induced by this eutectic solvent is also distinctly observed in the corresponding eutectogel (Supplementary Fig. 4 and Movie 2).

**Fig. 2:**
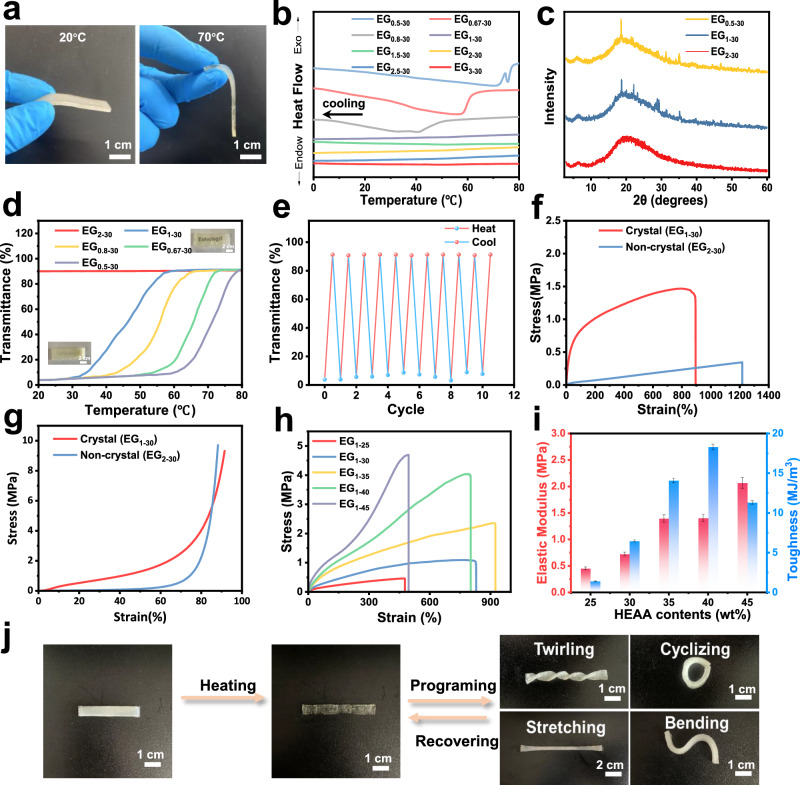
Material characterization of eutectogels. **a** Digital photographs of eutectogel EG_1–30_ at different temperatures, showing an opaque, crystalline, and rigid state at low temperature (20 °C), and a transparent, amorphous, and soft state at high temperature (70 °C). **b** Relationship between melting point and DES composition. The melting point increases with higher choline chloride contents. **c** XRD patterns of crystalline versus amorphous eutectogels. **d** Changes in light transmittance of eutectogels under the influence of temperature at different molar ratios. Scale bars, 2 cm. **e** Changes in light transmittance of crystalline eutectogel EG_1–30_ under temperature cycles of 20–80 °C. **f** Comparison of tensile properties between crystalline and amorphous eutectogels. **g** Comparison of compression properties between crystalline and amorphous eutectogels. **h** Effect of monomer content on the tensile properties of eutectogels. **i** Modulus and toughness of eutectogels at different monomer contents. Data are presented as mean ± SD (*n* = 3 independent samples). **j** Temperature-mediated shape memory properties of crystalline eutectogel EG_1–30_.

The integrity of these eutectogels is firstly investigated to test their prerequisite as switchable adhesives. The eutectogels prepared with different U:C ratios exhibit varied optical and mechanical properties in response to temperature change. The stiffness of the gels varies upon heating and cooling (Fig. 2a) by adjusting the DES composition. To improve interpretability, we have introduced a standardized nomenclature for eutectogels (e.g., EG_x-y_, where x represents the U:C ratio, and y denotes the monomer content). As shown in Fig. 2b, increasing the choline chloride content raises the melting point, while the crystallinity of the eutectogels also increases, as evidenced by the enhanced crystalline peak intensity in XRD patterns (Fig. 2c). The phase transition temperature of eutectogels can also be tailored to occur within ambient conditions (Supplementary Figs. 5 and 6). The eutectogel does not crystallise at room temperature when the molar ratio of urea and choline chloride is larger than 1:1. Except for DES composition, the ratio of polymer network and DES also affect the crystallization behavior. When the content of DES in the eutectogel is 30% or smaller, the crystallization is efficiently inhibited at room temperature (Supplementary Fig. 7). The crystallization tendency gradually diminishes with crystallinity dropping from from 89.58% at 90 wt% DES to a fully amorphous state at 30 wt% DES (Supplementary Fig. 8). The crystallization is also revealed by optical properties: crystalline eutectogels are opaque, with visible transmittance of only 3.8%, while amorphous gels reach ~90% transmittance (Supplementary Fig. 9). Upon heating from 20 to 80 °C, the transmittance of crystalline eutectogels increases from 3.8% to ~90%. The phase transition temperature further shifts with solvent composition, increasing from ~35 to ~70 °C as the U:C ratio changes from 1:1 to 1:2, consistent with DSC results (Fig. 2d). The transmittance change is reversible across heating–cooling cycles (Fig. 2e). At room temperature (20 °C), transmittance monotonically increases with polymer content, reflecting reduced scattering from DES domains (Supplementary Fig. 10).

Eutectogels also exhibit distinct mechanical contrast between crystalline and amorphous states. For example, the tensile fracture strength of eutectogels with U:C = 2:1 with 30% polymer content is only 0.2 MPa in the amorphous state but exceeds 1 MPa in the crystalline state (Fig. 2f and Supplementary Fig. 11). Crystallization enhances modulus (0.108 → 3.234 MPa) and toughness (2.196 → 10.62 MJ/m³) (Supplementary Fig. 12), and compression tests confirm the reinforcement effect (Fig. 2g and Supplementary Fig. 13). In addition, monomer content strongly affects mechanical properties by modulating solvent crystallization. As shown in Fig. 2h, increasing HEAA content from 25 wt% to 35 wt% improves tensile strength (0.15 → 2.3 MPa) and elongation at break (475% → 920%). Further increasing HEAA to 45 wt% raises tensile strength to 4.8 MPa, while elongation decreases to ~500% (Supplementary Table 1). Similarly, the modulus rises steadily with HEAA content (0.5 → 2 MPa; Fig. 2i), whereas toughness increases up to 18.3 MJ/m³ at 40 wt% HEAA before dropping to 11.32 MJ/m³ at 45 wt%, reflecting increased brittleness at higher crosslinking density due to stronger hydrogen bonding. Cyclic tensile tests at fixed strain (500%) demonstrate that the crystalline state greatly enhances cohesion and increases energy dissipation by ~30-fold compared to the amorphous state (Supplementary Figs. 14 and 15). The material’s structural integrity is evidenced by a cyclic compression test. Under a constant load of 50 N over ten cycles, the gel exhibited a mere 8% strain, confirming that crystallization substantially reinforces its cohesive strength (Supplementary Fig. 16). Furthermore, we systematically investigated the influence of the urea-to-choline chloride ratio on the mechanical properties. The elastic modulus is larger for samples with decreasing concentration of urea (EG_0.8–30_ > EG_2–30_), while decreases for sample EG_0.67–30_ and then increases for sample EG_0.5–30_. This complicated trend is probably due to complication of crystallization, hydrogen bonding between DES-DES, polymer chains-polymer chains, and DES-polymer chains (Supplementary Fig. 17).

The mechanical properties of crystalline eutectogels are further examined at different temperatures. Tensile tests reveal that while crystalline eutectogels exhibit strong mechanical performance at room temperature, both tensile strength and elongation at break decrease monotonically with increasing temperature from 20 to 70 °C (Supplementary Fig. 18a). Compression tests at 20 and 70 °C also confirm pronounced change in mechanical behavior across the crystalline-amorphous phase transition (Supplementary Fig. 18b).

Dynamic mechanical analysis is further employed to investigate the change of mechanical properties at different temperatures. The non-crystal eutectogel with (EG_2–30_) only shows slight decrease in storage modulus from 180 kPa (30 °C) to 100 kPa (95 °C) upon heating (Supplementary Fig. 19). In contrast, the crystalline eutectogels show sharp decrease in the mechanical properties: the storge modulus of crystalline eutectogel (EG_0.67–30_) decreases from 3500 kPa (30 °C) to 150 kPa (70 °C), and the storge modulus of crystalline eutectogel (EG_0.5–30_) decreases from 5000 kPa (30 °C) to 500 kPa (70 °C) (Supplementary Fig. 20). Rheological measurements also confirmed the softening of crystalline eutectogels at high temperature. This temperature-dependent property enables programmable shape morphing. As shown in Fig. 2j, the eutectogel remains rigid and opaque in its crystalline state at room temperature. Upon heating above the phase transition temperature, it becomes soft and transparent, allowing external forces, such as stretching, twisting, or bending, to reshape the material. Cooling back to room temperature re-fixes the deformed shape, while reheating above the transition temperature restores the original morphology. In addition, eutectogels exhibit notable self-healing capabilities due to their physically cross-linked network. A dumbbell-shaped sample is cut in half at 20 °C and reattached before being healed at 70 °C for 20 min, followed by recrystallization at 20 °C. The fractured sample recovers nearly completely to its original state (Supplementary Fig. 21a). In contrast, a sample healed for 20 min at 20 °C shows poor recovery in mechanical properties (Supplementary Fig. 21c). Notably, the sample healed at 70 °C restores more than 80% of its original tensile strength (Supplementary Fig. 21b). Furthermore, as no covalent crosslinking exists within eutectogels, they can be recovered and reused through suitable solvents, such as ethanol. The recovered eutectogel retains its crystallization properties (Supplementary Fig. 22). In addition, the DES used in this work is composed of urea (low toxicity) and choline chloride (a biocompatible essential nutrient). Compared to traditional organic solvents or toxic monomers, these components present minimal environmental risk and biological toxicity.

### Switchable adhesion of the eutectogel due to physical shape locking and surface chemistry

**Fig. 3:**
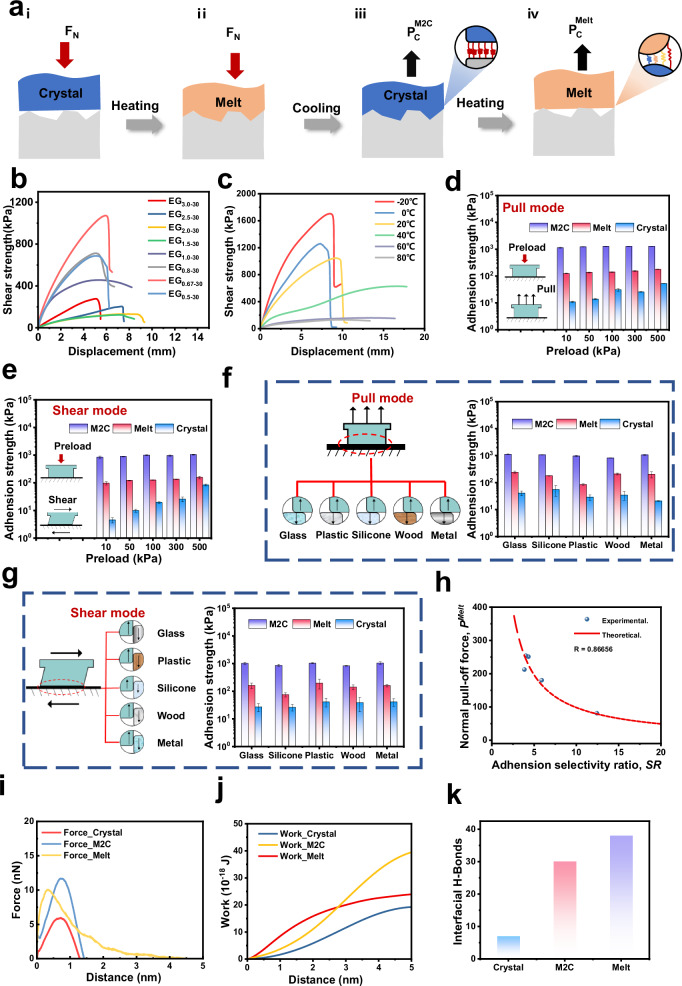
Mechanism of crystallization-enhanced adhesion in eutectogels. **a** Schematic representation of eutectogel adhesion on rough surfaces in crystal (C), melt (M), and melt-crystal states(M2C). **b** Effect of different solvent ratios on the adhesion properties of eutectogels. **c** Effect of temperature on the adhesion properties of eutectogel EG_0.67–30_ in shear adhesion test mode. **d**, **e** Influence of preload on adhesion strength under pull (top) and shear (bottom) modes (measured on smooth glass, aspect ratio = 1). The melt and melt-crystalline states reach adhesion equilibrium at a smaller pre-load pressure (100 kPa), while the crystalline state is difficult to equilibrate (sample information: EG_0.67–30_). **f**, **g** Adhesion strength on various substrates under pull (top) and shear (bottom) modes (aspect ratio = 1 and preload = 200 kPa) of eutectogel EG_0.67–30_. Adhesion characteristics, including high M2C adhesion, low melt adhesion, and minimal crystal adhesion, are consistent across substrates. **d**–**g** The adhesion force of the eutectic gel under different conditions is the average of the data from three tests; the error bars represent the standard deviation. **h** Adhesion selectivity ratio as a function of normal pull force. **i** Molecular dynamics simulation of force conditions and fracture work (**j**) during adhesion at crystallization, melting, and M2C states for eutectogels. The force required to release adhesion and fracture work during the M2C adhesion process is significantly greater than that required for the melt and crystal adhesion processes. **k** The number of interfacial hydrogen bonds formed between eutectogel EG_0.67–30_ and SiO₂ substrate in crystalline, melting, and M2C states (calculated via molecular dynamics simulations).

Next, we manage to find suitable and optimal eutectogel recipes for switchable adhesion. We first studied the mechanism of switchable adhesion of eutectogels. The crystalline eutectogel exhibits distinct adhesion mechanisms compared with conventional adhesive polymers, primarily owing to its superior surface-conformal capability. Heating the eutectogel to the molten state renders it soft and highly conformable, allowing it to adapt to rough substrates under preload (Fig. 3a), distinguishing itself from conventional crosslinked polymers. Upon cooling and crystallization, the gel locks into the substrate topography, giving rise to the M2C adhesion mechanism. Reheating to the molten state softens the gel and significantly weakens adhesion, enabling easy detachment.

The influence of composition on the adhesion strength of eutectogels is further quantified to optimize the composition (Fig. 3b). For U/C > 1:1, the gels (EG_1–30_) are amorphous at room temperature. For samples with different DES, the adhesion strength at room temperature decreases with increasing urea content—from 276.6 kPa at EG_3–30_ to 118.3 kPa at EG_1.5–30_. This reduction is attributed to the lower density of amino groups available for hydrogen bonding with substrates as the urea content decreases. In contrast, when U/C < 1:1, the eutectogels exhibit crystalline features. The adhesion strength first increases and then decreases with less urea content, reaching a maximum of 1.06 MPa at EG_0.67–30_ before declining to 686.3 kPa at EG_0.5–30_. The initial enhancement is ascribed to stronger gel cohesion induced by low-temperature crystallization, while further reduction in urea weakens gel–substrate interactions, lowering adhesion strength. Changes in eutectogel cohesion due to solvent composition variations are characterized through mechanical testing. At a U/C molar ratio of 1:1.5, the eutectogel (EG_0.67–30_) exhibited significantly lower modulus compared to gels with EG_0.5–30_ and EG_0.8–30_ (Supplementary Fig. 17). Therefore, the U/C molar ratio of 1:1.5 is selected as the DES for subsequent studies (EG_0.67–30_), unless otherwise specified. Due to the inherent hydrogen bonding provided by the DES at the interface, the adhesion force maintains a baseline of ~100 kPa. This suggests that the chemical nature of the solvent is the governing factor for adhesion in the melted state, limiting the impact of polymer network modifications.

Given that eutectogel adhesion is governed by temperature-mediated phase transitions, the role of crystallization is evaluated by measuring M2C adhesion at different temperatures (Fig. 3c). From −20 °C to 80 °C, M2C adhesion decreases monotonically with increasing temperature, due to progressive loss of crystallinity and thermal disruption of hydrogen bonding, both of which diminish gel cohesion and adhesive strength. Notably, crystalline eutectogels can be annealed into an amorphous phase, enabling recyclability through repeated melt-crystallization cycles. As shown in Supplementary Fig. 23, adhesion strength on glass substrates remains stable over multiple adhesion–detachment cycles. Similarly, repeated shear-loading tests with maximum deformation of 0.1 mm demonstrate that eutectogels endured over 100 dynamic cycles without degradation of bonding performance (Supplementary Fig. 24). Heavy-load testing further confirms their robustness: a eutectogel pad with an area of only 2.5 cm² supported a 7.5 kg dumbbell in the M2C state (Supplementary Fig. 25). These findings highlight the exceptional fatigue resistance, recyclability, and mechanical adaptability of phase-change eutectogel adhesives. Moreover, because the melting point of the DES can be precisely tuned through composition design, the switching temperature of adhesion can be programmed to meet specific application requirements.

To validate the underlying mechanism, we systematically measured adhesion strength in the crystalline, molten, and M2C states via pull-off and shear tests. In both pull mode (Fig. 3d and Supplementary Fig. 26) and shear mode (Fig. 3e and Supplementary Fig. 27), the molten and M2C states exhibited saturation of adhesion strength at a relatively low preload of 50 kPa, reflecting the softness of the gel during contact formation. In the M2C state, tensile and shear adhesion strengths achieve 1.23 MPa and 0.89 MPa, respectively, whereas the molten state are as markedly lower values of 138.2 kPa and 123.2 kPa. At a preload of 50 kPa, the adhesion-to-preload ratio of eutectogels is ~25, exceeding that of conventional dry adhesives and bioinspired structural elastomers^55,56^. By contrast, adhesion in the crystalline state increases monotonically with preload, attributed to frictional enhancement at higher loads.

The eutectogel also exhibits robust adhesion across diverse substrates, including plastics, plexiglass, wood, and metals in both pull (Fig. 3f and Supplementary Fig. 28) and shear modes (Fig. 3g, Supplementary Figs. 29 and 30). M2C adhesion reached ~1 MPa on all tested surfaces, whereas adhesion in the molten state is reduced by an order of magnitude, and that in the crystalline state decreases by an additional order of magnitude due to its rigidity and poor conformability. The M2C mechanism thus enables strong and versatile adhesion by combining high conformability in the molten state with rigid shape-locking in the crystalline state. For rough surfaces, such as sandpaper, eutectogels demonstrated inverse replication of surface morphology upon crystallization, while maintaining comparable roughness levels (Supplementary Fig. 31). The M2C adhesion mechanism resembles the phase-transition adhesion of shape memory polymers. Accordingly, we apply the rubber-to-glass (R2G) adhesion theory of shape memory polymers to model the M2C process. Here we consider two types of surfaces, rough and smooth, whose areas are denoted as *s* and *s₀*, respectively. The pull-off forces of the M2C and molten states on a reference smooth surface are defined as ${P}_{0}^{{{{\rm{M}}}}2{{{\rm{C}}}}}$ and ${P}_{0}^{{{{\rm{Melt}}}}}$, while those on a rough surface are defined as ${P}_{c}^{{{{\rm{M}}}}2{{{\rm{C}}}}}$ and ${P}_{c}^{{{{\rm{Melt}}}}}$. For the rough surface, we introduce the roughness ${h}_{{{{\rm{rms}}}}}$ and its slope ${h}_{{{{\rm{rms}}}}}^{{\prime} }$; for the reference surface, these quantities are defined as ${h}_{{{{\rm{rms}}}}}^{0}$ and ${h}_{{{{\rm{rms}}}}}^{{\prime} 0}$. The adhesion selectivity ${{{\rm{SR}}}}$ is defined as the ratio of the pull-off forces between the M2C and melt states on a rough surface, which reflects the competitive relationship between the adhesive and deadhesive states. Previous studies have shown that ${{{\rm{SR}}}}$ is inversely proportional to ${h}_{{{{\rm{rms}}}}}^{{\prime} }$, as described in Eq. 1^25^. We further find that ${P}_{c}^{{{{\rm{Melt}}}}}$ is inversely proportional to ${{{\rm{SR}}}}$, as indicated in Eq. 2. The theoretical predictions and experimental results are presented in Fig. 3f, and the adhesion values of eutectogels in both the molten and M2C states are in good agreement with the theoretical models (Eqs. 1 and 2, Fig. 3h). However, adhesion in the crystalline state deviates significantly, highlighting the fundamentally different mechanism dominated by interfacial friction (Supplementary Fig. 32).

1 $${SR}=\frac{{P}_{C}^{{{{\rm{M}}}}2{{{\rm{C}}}}}}{{P}_{C}^{{{\mathrm{Melt}}}}}=\frac{{P}_{0}^{{{{\rm{M}}}}2{{{\rm{C}}}}}}{{P}_{0}^{{{\mathrm{Melt}}}}}.\frac{S}{{S}_{0}}\frac{{h}_{{rms}}^{{\prime} }}{{h}_{{rms}}^{{\prime} 0}}={SR}_{0}.\frac{S}{{S}_{0}}\frac{{h}_{{rms}}^{{\prime} }}{{h}_{{rms}}^{{\prime} 0}} \sim {h}_{{rms}}^{{\prime} }$$

2 $${P}_{c}^{{{\mathrm{Melt}}}} \sim \frac{1}{{h}_{{rms}}^{{\prime} }} \sim \frac{1}{SR}$$

To elucidate the molecular mechanisms governing eutectogel adhesion, we combine molecular simulations with spectroscopic characterization to probe interfacial interactions. Molecular dynamics simulations are first employed to model the evolution of forces during the adhesion process (Supplementary Figs. 33 and 34). As shown in Fig. 3i, the adhesion achieved via M2C conformal locking is markedly stronger than conventional direct adhesion on rough substrates. Both the peak adhesion force and the fracture energy associated with M2C adhesion are substantially higher than those of conventional adhesion (Fig. 3j). Simulation of hydrogen-bond formation between the eutectogel and glass substrate (Fig. 3k) further revealed a greater number of hydrogen bonds in the M2C state. This enhancement likely arises from thermal activation of hydrogen-bonding functional groups (e.g., –NH₂, –OH) in the eutectogel, which promotes interfacial hydrogen bonding, coupled with a heating-induced modulus reduction that increases the real contact area. Subsequent cooling-induced crystallization locks the gel shape and enhances cohesive strength.

To validate these findings, we investigated the molecular mobility of the gel components using low-field proton nuclear magnetic resonance (L-¹H NMR), in situ variable-temperature infrared spectroscopy, and two-dimensional correlation spectroscopy, as revealed in Supplementary Information (Supplementary Figs. 35–41). While initial characterizations focused on urea/choline chloride-based crystalline eutectogels, we conducted a systematic investigation across diverse eutectic solvent systems to verify the universality of this intelligent adhesion behavior. For instance, substituting choline chloride with betaine yielded similar crystallization characteristics; by modulating the urea:betaine molar ratio (e.g., 2:1 vs. 1:2), we successfully transitioned the resulting eutectogels from an amorphous to a crystalline state at room temperature (Supplementary Fig. 42). To further substantiate this strategy, we expanded our scope to include various chemical motifs, such as alcohol/quaternary ammonium salts (sorbitol/choline chloride), metal halides/quaternary ammonium salts (zinc chloride/choline chloride), organic acids/quaternary ammonium salts (lauric acid/tetraoctylammonium bromide), and alcohol/acid systems (menthol/lauric acid). In each case, precisely tuning the composition ratio allowed for the successful preparation of room-temperature crystallizing eutectogels (Supplementary Figs. 43–46). These results confirm that our strategy is highly robust and universally applicable, allowing for the selection of specific eutectic solvents, such as hydrophobic systems for underwater applications, to meet diverse environmental demands.

### Remote sensing and monitoring of adhesive status

Adhesive performance can be compromised by environmental factors (e.g., elevated temperature or humidity), loading conditions, or improper bonding, ultimately leading to premature failure. Real-time monitoring of adhesion is therefore essential to prevent bond degradation and potential catastrophic consequences, especially for those in inaccessible position or confined spaces. Recent advances in electronics enable the transduction of physical quantities, such as light, force, and sound, into electrical signals^48,57^. In particular, ionic and electronic conductors have been used to sense external forces via changes in capacitance or resistance, suggesting that converting adhesive status into electrical signals is a viable strategy for achieving “adhesive informatization.”

**Fig. 4:**
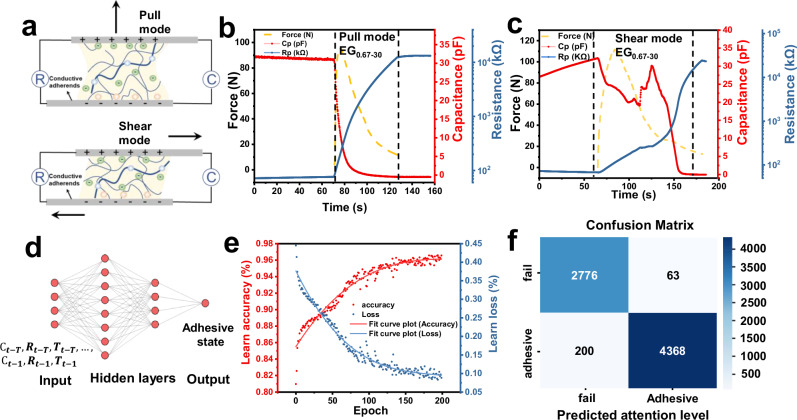
Intelligent sensing of the adhesive state. **a** Schematic diagram of the intelligent sensing adhesive constructed from eutectogel and two layers of stainless-steel metal sheets. **b** Adhesion of the M2C eutectogel EG_0.67–30_ to metal surfaces under tensile loading, and the corresponding changes in capacitance and resistance. The force on the vertical axis represents the applied pull force used to simulate the external force required to change the adhesion state, thereby enabling the monitoring of adhesion state changes. **c** Applied shear force on M2C eutectogel between metallic surfaces in shear mode and corresponding change in capacitance/electrical resistance of eutectogel EG_0.67–30_. **d** Machine learning algorithm model diagram. **e** Learning accuracy and F1 score function over multiple epochs. **f** Corresponding prediction confusion matrix using eutectogel EG_0.67–30_.

In this work, P(HEAOMA) eutectogels are fabricated with choline chloride as the ionic component, imparting intrinsic ionic conductivity. The sensing principle for a eutectogel–metal electrode assembly is illustrated in Fig. 4a and Supplementary Fig. 47. This configuration functions as a supercapacitor, in which the electric double layer (EDL) bilayers formed at the gel–electrode interfaces generate capacitances C_1_ and C_2_. The effective contact area between the gel and the electrode directly influences these capacitances, enabling capacitance changes to report on the ion–conductor interface state. Fractures or internal defects in the gel also substantially increase electrical resistance, allowing cohesive strength to be inferred from resistance measurements.

Adhesion strength is evaluated under shear and tensile loading until fracture, while simultaneously monitoring capacitance and resistance. As shown in Fig. 4b, in the tensile mode, the baseline capacitance and resistance remain stable in the absence of external force. During tensile loading, the capacitance decreases as the interfacial distance increases, and its fluctuations correspond to the formation of interfacial defects. The subsequent increase in resistance indicates that internal gel damage has begun to occur. During the final fracture stage, the capacitance drops sharply while the resistance increases significantly, consistent with the rapid propagation of cracks at the interface between the gel and the adhesive. The fluctuations in the electrical signals and the external force loading behavior throughout the entire process are almost perfectly aligned on the time axis. A similar trend is also observed in the shear mode (Fig. 4c).

To facilitate early warning of adhesive failure, we developed an integrated prediction system centered on an STM32 microcontroller. This system leverages onboard Bluetooth for real-time signal transmission to mobile devices (Supplementary Fig. 48). Using temperature as an external stimulus, we monitored and recorded the dynamic electrical signal changes of eutectogels during the adhesion and peeling processes across a range of thermal conditions (Supplementary Figs. 49 and 50). To predict imminent adhesive failure within a 1-s horizon, we formulated the challenge as a supervised machine-learning task utilizing a lightweight fully connected neural network (FCN) designed for rapid inference. The FCN was trained on an extensive dataset encompassing the temporal evolution of adhesion and non-adhesion states across 37 distinct temperature settings ranging from −20 °C to 80 °C. Each training sample is structured as a flattened input vector derived from a three-second temporal window, incorporating time-series features of capacitance, resistance, and ambient temperature. This input is paired with the adhesive state label immediately following the window, thereby establishing a predictive mapping from past signal evolution to the future adhesion state (Fig. 4d). The model reached its performance peak, characterized by minimum loss and maximum accuracy, at epoch 200 (Fig. 4e). The trained model achieved a robust test-set accuracy of 96.10%, with misclassifications restricted to a small fraction of the extensive 7407-sample test set (Fig. 4f). Crucially for early-warning applications, where the cost of an undetected failure far outweighs that of a false alarm, the model prioritized the minimization of false negatives to yield a high recall rate of 96.39%. This high sensitivity ensures the reliable detection of impending structural compromise, validating that the integration of real-time sensing with machine-learning analysis offers a practical, high-fidelity solution for the remote monitoring of structural adhesive integrity.

### Applications of the switchable adhesive surfaces: smart gripper

Switchable adhesive surfaces offer precise control over object capture and release. The growing demand for robotic soft grippers has spurred interest in smart adhesives with tunable adhesion. Among these, eutectogels exploiting physical phase transitions are particularly promising, as they combine reversible switching mechanisms with straightforward structural tunability, thereby enabling both strong grasping and facile release of target objects. In addition, the above-mentioned synergy of physical shape-locking and switchable chemical bonding enables a robust switch of the surface adhesion.

**Fig. 5:**
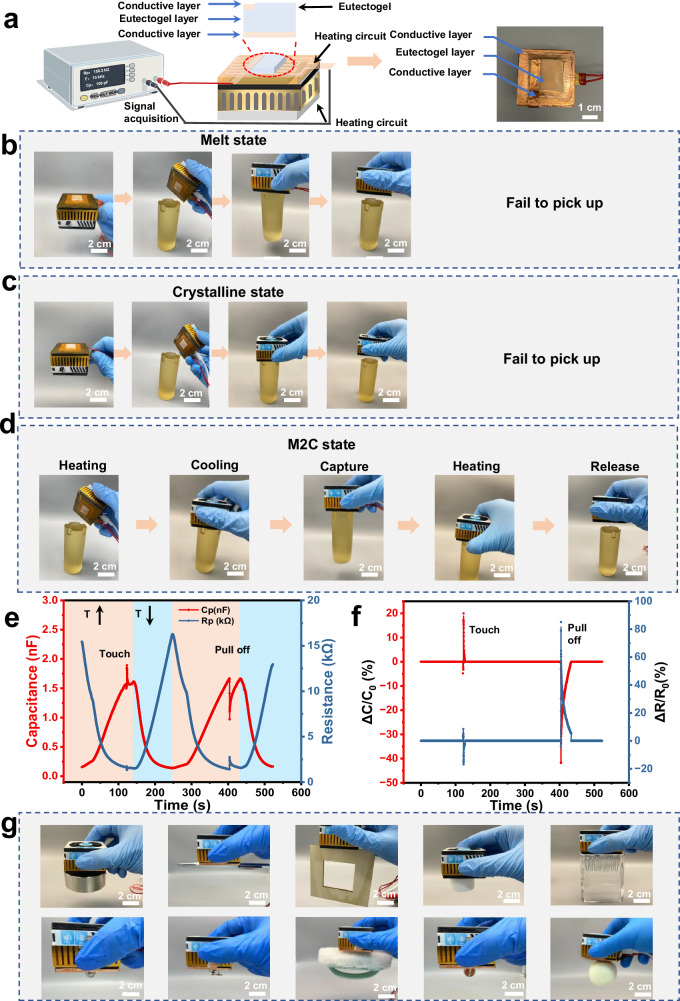
Development of smart adhesive grippers based on crystalline eutectogels. **a** Schematic diagram and photo of an intelligent adhesive gripper structure based on eutectogel. **b** The eutectogel smart gripper in the melt state tends to lose its adhesion to the target object during lifting. **c** In the crystalline state, eutectogel grippers have difficulty grasping target objects through adhesion. **d** Digital photos of the process of picking up target items using eutectogel (EG_0.67–30_) smart gripper based on the M2C process. **e** Electrical signal changes during the process of the eutectogel (EG_0.67–30_) smart gripper grasping target objects. **f** After signal decoupling, the electrical signal shows distinct fluctuation when the intelligent gripper grasps and releases the target object. **g** The eutectogel (EG_0.67–30_) smart gripper is capable of grasping objects with diverse surfaces made from various materials, including metal block and sphere, PTFE hollow cylinder, wooden frame, prismatic metal pen, glass cup and glass sphere, metal thumbtack, porous sponge, and egg.

Here, we integrate the phase-transition-driven switchable adhesion of P(HEAOMA) eutectogels into a temperature-controlled soft gripper. The adhesive strength of the eutectogel originates from its crystalline-amorphous transitions. Unlike rigid grippers, soft grippers are compliant, adaptive, and capable of conforming to irregular geometries. To demonstrate cargo capture, transport, and release, a 2 mm-thick eutectogel layer is mounted on a temperature control unit consisting of a heat sink and a heating film (Fig. 5a), enabling rapid cycling between 20 and 70 °C (Supplementary Fig. 51). In the molten state, the gel exhibits strong adhesion and conforms intimately to object surfaces, but its low modulus restricts load-bearing capacity (Fig. 5b). In the crystalline state, solvent crystallization minimizes interfacial adhesion, preventing reliable lifting of heavy objects (Fig. 5c). Optimal lifting is achieved through a melt-crystallize sequence: heating to the molten state establishes intimate contact with the target, followed by cooling-induced crystallization that mechanically locks the interface and allows stable lifting. Reheating then reduces adhesion, enabling gravity-assisted release. This complete pick-carry-release cycle is shown in Fig. 5d and Supplementary Movie 3. To investigate the long-term behavior of grippers when handling heavy objects, we demonstrated the sustained grasping performance of eutectogel in crystalline, molten, and M2C states. Observations revealed that in both crystalline and molten states, the gripper could not hold heavy objects for long periods. However, after undergoing the M2C process, the gripper maintained a stable state for extended periods while holding heavy objects (Supplementary Figs. 52–54 and Movie 4). We also conducted heavy-load grasping tests at relatively high temperatures (40 °C) and low temperatures (−10 °C) to verify the external environment’s impact on this gripper. Results indicate that the gripper maintains reliable grasping and release capabilities under these conditions (Supplementary Fig. 55 and Movie 5).

Additionally, due to the intrinsic ionic conductivity arising from the ionic composition of eutectogels, monitoring of electrical signals enables the tracking of the gripper’s own state and the magnitude of contact forces during the object’s contact and separation process. Figure 5e, f illustrate the schematic of the gripper detecting electrical signals and the curve of electrical signal changes during the gripping process. Part of the eutectogel is covered with conductive copper, therefore the conductivity/capacitance signal can be probed during gripping. Fluctuations in the electrical signal provide a direct indication of the gel’s state on the gripper. To facilitate a more intuitive comparison between the gripper’s grasping/releasing actions and the signal variations, thermal interference from heating and cooling cycles was eliminated via differential calculation. As shown in Fig. 5f indicating the electrical signal change decoupled from the temperature variation, when the gripper contacts the target object, the capacitance signal increases significantly, while the corresponding resistance signal decreases. Conversely, when the object is released, capacitance decreases and resistance increases. Further testing with weights of varying masses generates different pressures to assess the gripper’s sensitivity to contact force magnitude. The ratio of electrical signal fluctuations produced by weights ranging from 20 to 500 grams clearly demonstrates an approximate multiplicative relationship between different force magnitudes (Supplementary Fig. 56). Supplementary Table 2 shows the control parameters of the eutectogel intelligent gripper in the process of grasping the target object. The energy cost of a pick and release (2 × 30 s) cycle can be calculated by the applied voltage and current, which is about 600 J.

This temperature-programmed adhesion strategy gripper is widely applicable for grasping objects of varying materials, shapes, and sizes (both larger and smaller than its own diameter) (Fig. 5g and Supplementary Movie 6). In the M2C phase, the eutectogel exhibits high adhesive strength, enabling the transport of objects up to 4 kg (Supplementary Fig. 57a). Notably, the adhesive surface remains effective even under dusty conditions and sustains strong adhesion over multiple cycles. The system is capable of heavy-load manipulation, as demonstrated by the reliable handling of a 1 kg object (Supplementary Fig. 57b–d and Movie 7). To better assess the state changes of the eutectogel gripper during the gripping process, temperature sensors monitored the heating and cooling cycles on the gel’s surface. As shown in Supplementary Fig. 51, when the surface temperature reached 70 °C, the eutectogel entered a fully molten state due to thermal transfer efficiency. Similarly, when the temperature dropped to 20 °C, the gel transitioned into a crystalline state. The entire switching process is maintained at approximately 100 s. By comparing various types of grippers recently reported across multiple dimensions, including adhesion force, surface adaptability, grasping time, and energy consumption, grippers constructed from eutectogels based on crystalline transformation demonstrated advantages, such as high adhesion force, broad adaptability, and low energy consumption (Supplementary Table 3).

### Development and application of wall-climbing robots

Wall-climbing robots play a pivotal role in diverse applications, such as infrastructure inspection. A comprehensive comparison between our robot and state-of-the-art systems is provided in Supplementary Table 4. While the current climbing speed of our robot is relatively modest compared to some existing platforms, it offers a balance of versatility and safety. Specifically, our system demonstrates robust adaptability to various wall materials and surface roughness while operating under a relatively low voltage of 6 V. Most importantly, the integration of switchable adhesion with real-time adhesion status sensing, provides a critical safety layer that prevents catastrophic falls, a feature often absent in conventional designs.

**Fig. 6:**
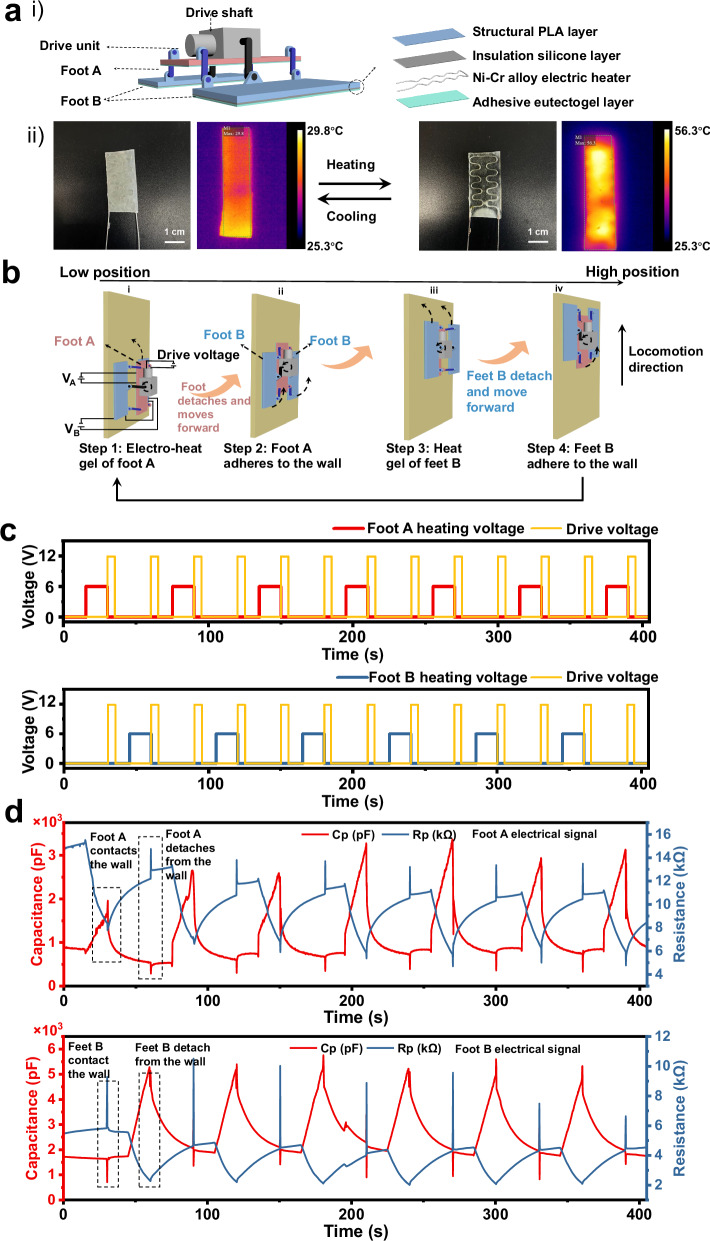
Design principle and fabrication of the wall-climbing robot. **a**-i Illustration of the mechanical design of the three-feet wall-climbing robot (left), and layout of the foot including a supporting layer (size: 130 mm × 50 mm × 5 mm), an insulation layer (size: 50 mm × 25 mm × 2 mm), a metallic layer, and the eutectogel (EG_0.67–30_) layer (size: 50 mm × 25 mm × 2 mm). ii Infrared images probing the heating process of eutectogel with a conductive circuit embedded in the gel (EG_0.67–30_). **b** Schematic diagram of the climbing principle of the wall-climbing robot. The robot climbing motion is realized by human-coordinated control. **c** Control signals for the foot A and feet B of the wall-climbing robot during the heating-adhesion-cooling-crystallization-heating-peeling process, including the heating voltage (6 V, 2 A) and drive voltage (12 V, 3 A). The drive voltage is used to drive the motor of the shaft while the heating voltage is used to apply on foot A and feet B, respectively. **d** The electrical sensing signals of foot A and feet B change during locomotion upon contacting and detaching from the wall, indicating the real-time status of feet.

Our eutectogel-based three-legged wall-climbing robot consists primarily of drive motors and adhesive feet (Supplementary Fig. 58). Locomotion and adhesion are achieved through two independent electric heating circuits and a single electric drive circuit, with all switching and speed regulation integrated into a 3D-printed control unit. The main body and spindle are fabricated via hot-melt extrusion trial 3D printing and light-curing 3D printing, respectively. The robot is functionally divided into a central main foot (foot A) and two lateral feet (feet B) (Fig. 6a). Detailed structural design is shown in Supplementary Figs. 59, 60, and Table 5. The electrothermal eutectogel layer is polymerized directly onto the stainless-steel surface, incorporating a nickel–chromium resistance wire. When supplied with 6 V and 2 A, the wire generates sufficient heat to melt the crystallized eutectogel, enabling reversible phase transitions. As shown in Fig. 6a-ii, heating of the eutectogel with a voltage of 6 V and current of 2 A rapidly melts the crystalline structure within ~15 s, rendering a transparent phase. Upon power interruption, the gel reverts to its opaque crystalline state. Thermal imaging reveals that the eutectogel temperature exceeds 100 °C during heating, and subsequently decreases more slowly during cooling in the absence of a dedicated metal heat sink (Supplementary Fig. 61a). The spatial temperature distribution during operation confirms localized heating of the eutectogel with minimal environmental heat transfer (Supplementary Fig. 61b).

The robot climbing mechanism and locomotion sequence are illustrated in Fig. 6b. The movement cycle consists of several distinct steps: a dual-axis gearmotor mounted on foot A drives the rotary shaft in incremental segments. This segmented rotation, coordinated between the drive and support shafts, causes the main foot (foot A) and the two auxiliary feet (foot B) to lift and lower alternately. This motion enables the robot to progress forward (Supplementary Fig. 62). Supplementary Table 6 lists the control parameters for the eutectogel-based wall-climbing robot during the crawling process. Calculations based on these parameters indicate that the energy consumption per crawling cycle is approximately 1080 J.

The robot demonstrated stable climbing on rough vertical surfaces (Supplementary Fig. 63), aided by optimization of the gel heating/cooling balance. The adhesive state of the eutectogel and the robot’s crawling posture are monitored via electrical signals. Similar to the gripper section, part of the adhesive pad of the climbing robot contacting wall surface is covered with conductive copper, such that the robot’s feet can sense adhesive status in all scenarios, not only on conductive surface. As shown in Fig. 6c, when the middle foot (foot A) is lifted, and the heating switch is activated, the eutectogel heats up to its molten state, causing the resistance to decrease and the capacitance to increase, placing the gel in a viscous state. When the drive switch is activated and the middle foot contacts the wall, the capacitance signal increases significantly. When the heating switch is turned off, the gel temperature decreases, causing the capacitance to decrease and the resistance to increase. When the middle foot detaches from the wall, opposite electrical signal fluctuations occur, manifested as a decrease in capacitance and an increase in resistance. This movement is synchronized with the heating-cooling cycle of the electrothermal gel layer (Supplementary Movie 8). Similar changes also occur in the side feet (foot B) (Fig. 6c). The heating cycles of the main and lateral feet are maintained in opposite phases: when one foot is lifted, its gel layer is heated to induce melting; when it is loaded, heating ceases, and cooling is facilitated via the stainless-steel layer and wall contact. The electrical signals fluctuate in response to wall contact (Fig. 6d), providing a dual sensing function: detecting initial contact and confirming whether the foot has achieved a secure grip. On walls with inclinations of 30°, 60°, and 90°, each lift-adhere cycle requires approximately 15 s of heating to achieve full melting, and 30 s of cooling for recrystallization (Supplementary Movie 9).

**Fig. 7:**
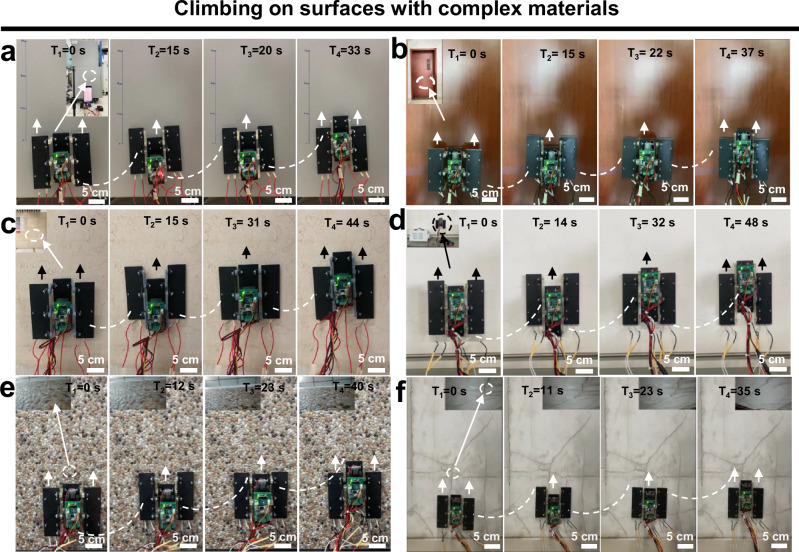
Robot climbing on surfaces of various materials and roughness using eutectogel EG_0.67–30_. **a** Robot climbing on the metallic wall. **b** The robot is climbing on the wooden wall. **c** Robot climbing on the ceramic tile wall. **d** Robot climbing on the lime wall. **e** Robot climbing on the rough stone wall with a camera for inspection. **f** Robot climbing on the cracked lime plaster wall with a camera for inspection.

Performance under reduced voltage is also evaluated. At 3 V, the resistance wire exhibited lower maximum temperatures and slower heating rates (Supplementary Fig. 64). Despite this, operational testing (Supplementary Fig. 65) demonstrates effective locomotion on inclined walls (Supplementary Movie 9). The climbing robot also works for vertical walls composed of diverse materials, including metallic wall (Fig. 7a and Supplementary Movie 10), wooden wall (Fig. 7b and Supplementary Movie 11), ceramic tile wall (Fig. 7c and Supplementary Movie 12), lime wall (Fig. 7d and Supplementary Movie 13), rough stone wall (Fig. 7e and Supplementary Movie 14) and cracked lime plaster wall (Fig. 7fand Supplementary Movie 15). The robot consistently maintains a cycle time of 30–40 s per full 360° rotation across all tested surfaces. The system is also equipped with an onboard camera, enabling real-time imaging of wall surface conditions during climbing. From recorded distance and time, the climbing speed is determined to be 8 cm/min while carrying a combined payload exceeding 700 g (robot mass  > 500 g plus a 200 g camera).

## Discussion

In summary, we have demonstrated a strategy of (electro)thermally switchable eutectogels that overcome critical limitations of current switchable adhesives. By leveraging a reversible crystalline-to-melt phase transition, these eutectogels achieve robust, shape-locking adhesion across an exceptionally wide range of substrates, regardless of material composition or surface roughness. Compared to existing gripping and climbing technologies, our system offers three distinct advantages. (1) High adhesion strength on rough surfaces: while traditional dry adhesives (e.g., gecko-inspired structures) often fail on non-smooth or dusty surfaces, and liquid/gel adhesives lack mechanical stability, our eutectogel achieves an adhesion strength of up to 1700 kPa. This is notably higher than many reported supramolecular gels and stimuli-responsive polymers, particularly on challenging porous or textured substrates. (2) Material versatility and environmental tolerance: unlike adhesives limited to specific polymers or metals, the “shape-locking” mechanism allows our material to conform to diverse interfaces, bridging the gap between soft robotics and heavy-duty industrial grippers. (3) Integrated intelligence: the synergy between high-strength adhesion and real-time remote monitoring (via conductance/capacitance sensing) provides a closed-loop feedback mechanism for wall-climbing robots that is often absent in purely mechanical gripping systems. These advances not only broaden the scope of applicable adherends but also provide a scalable strategy for developing multifunctional, “smart” adhesive interfaces. Future work will focus on optimizing the switching speed and investigating the long-term durability of the eutectogels under extreme environmental conditions, further positioning them as a versatile tool for next-generation soft robotics and automated manufacturing.

## Methods

### Materials

N-hydroxyethyl acrylamide (HEAA, 98%) and N, N-dimethylacrylamide were purchased from Tokyo Chemical Industry. Urea (99%), acetone (AR), ethanol (AR), ether (AR), hydroquinone (99%), and other general reagents were obtained from Sinopharm Chemical Reagent Co. Choline chloride (99%), octadecyl bromide (99%), and N, N-dimethylaminopropyl acrylamide (98%) were purchased from Aladdin Reagent Co. Lauric acid (AR, 98%), menthol (AR, 98%), zinc chloride (AR, 99.5%), tetraoctylammonium bromide, sorbitol (AR, 99.5%), betaine (AR, 99.5%) purchased from Shanghai Macklin Biochemical Co., Ltd. Photoinitiator Irgacure 2959 was supplied by Energy Chemical Reagent Co. Prior to use, urea and choline chloride were vacuum-dried and stored under dry conditions.

### Preparation of DES solvents and monomers

Dried urea and choline chloride were weighed according to the desired molar ratio and transferred into a round-bottom flask. The mixture was heated to 90 °C under continuous stirring until a homogeneous, transparent liquid formed. Other types of eutectic solvents were prepared using similar heating methods.

The long-chain hydrophobic quaternary ammonium monomer was synthesized via a one-step reaction. Specifically, dimethylaminopropyl acrylamide (0.1 mol) and octadecyl bromide (0.12 mol) were charged into a single-neck flask, along with hydroquinone (10 mg) as a polymerization inhibitor. Acetone (50 mL) was introduced as the solvent, and the mixture is stirred magnetically at 60 °C for 4 h. After completion, acetone was removed using a rotary evaporator, and the crude product is precipitated in diethyl ether. The precipitate was collected via filtration, shed three times with ether, and dried under vacuum to eliminate any residual solvent. The chemical structure of the final product was verified by ¹H NMR spectroscopy.

### Preparation of P(HEAOMA) eutectogels

P(HEAA-OMA) eutectogels were synthesized as follows: specified amounts of N-hydroxyethyl acrylamide (HEAA), octadecylmethacryloxyethyl dimethylammonium chloride (OMA), and the photoinitiator IRGACURE 2959 (1 wt% relative to monomers) were dissolved in a urea–choline chloride deep eutectic solvent. The mixture was stirred at 60 °C for 5 min to ensure complete dissolution, followed by sonication to remove entrapped air bubbles. The homogeneous solution was then poured into a rectangular mold composed of glass plates separated by a silicone spacer. Photopolymerization was carried out under UV irradiation at 60 °C for 30 min. For comparison, P(HEAA) eutectogels and other DES types eutectogels prepared in the DES solvent were fabricated using the same procedure. The crystallization process and crystal morphology of the eutectogels were monitored in situ using polarized optical microscopy. To fabricate electrothermal gel modules, a pre-bent resistive wire was fixed in the center of a silica mold, followed by injection of the eutectogel precursor solution. The assembly was then polymerized under UV light for 30 min.

### Characterization

The optical transparency and phase transition behavior were evaluated by recording the transmission spectra at various temperatures using a UV–vis spectrophotometer (Shimadzu UV3700, Japan), with a sample thickness of 2 mm. Variable-temperature FTIR measurements (Bruker TENSOR II) were conducted to monitor the phase transition process between 25 and 70 °C. Additionally, differential scanning calorimetry (TA DSC Q2000) was used to determine the phase transition temperatures of P(HEAA-C18DM) eutectogels over a range of 0–80 °C. XRD measurements were carried out with a Theta Rotating anode X-ray diffractometer TTR III (Rigaku Corporation). Low-field NMR characterization was performed with VTMR20-010V-I. The microscopic surface topographies were measured using a Chotest Technology Inc (Chotest) white light interferometer (SuperView W1). TA Discovery HR-2 Rheometer were used to measure the change of modulus of eutectogels with temperature at different molar ratios. Temperature sweep data were obtained at a fixed frequency (*f* = 6.2 rad/s) and strain (*γ* = 0.5%) covering a range of 0–120 °C (ramp rate: 4 °C/min). TA Discovery DMA850 dynamic mechanical analyzer were used to measure the change of modulus of eutectogels with temperature at different mole ratios. Uniaxial tensile tests were conducted using a universal mechanical testing machine (Sansi Zongheng Technology Co., Ltd., China) equipped with both 100 and 2000 N load cells. Dumbbell-shaped specimens with dimensions of 35 mm × 10 mm × 2 mm were prepared using standard cutters and stretched at a constant crosshead speed of 100 mm/min. For compression testing, cylindrical samples with a diameter of 15 mm were placed between two parallel plates and compressed under identical machine conditions. Cyclic tensile loading–unloading tests were performed by stretching the gel samples to 500% strain and subsequently returning to the initial length at a rate of 100 mm/min. A total of ten consecutive cycles were carried out without intervals between cycles.

### Adhesion performance testing

The adhesion strength of eutectogel samples on various substrate surfaces was evaluated using both shear and pull modes. Eutectogel specimens approximately 1 mm thick were sectioned into 1 cm × 1 cm squares, while the adherend substrate sheets are cut into 5 cm × 5 cm strips. A eutectogel sample was sandwiched between two substrate strips with an overlap area of 1 cm × 1 cm. The contact region was subjected to compressive loads ranging from 10 g to 7 kg for 5 min to ensure complete and uniform adhesion across the overlapping section. During adhesion testing, the upper and lower substrates were secured in the chucks of a universal tensile testing machine. Shear strength is derived from the measured tensile force at a constant strain rate of 10 mm/min. The substrates employed included aluminum, copper, polyethylene plastic, glass, silicone rubber, and wood. In pull-mode adhesion testing, the adherent sample was brought into contact with a rough substrate at a low approach velocity of 10 µm/s. It was then subjected to a constant tensile force for a duration of 5 min, after which separation was performed at a rate of 100 µm/s. To conduct adhesion measurements at various temperatures, samples were preconditioned at each target temperature for approximately 30 min to ensure thermal equilibrium of the intermediate adhesive gel. Repeated thermal adhesion switching was performed by subjecting the same sample to alternating high- and low-temperature cycles. The sample was first placed in an oven at 80 °C until a homogeneous and transparent state was achieved, at which point adhesion was measured. It was then transferred to a desiccator maintained at 20 °C to induce complete crystallization before subsequent adhesion testing. This entire process was repeated for five consecutive cycles.

### Intelligent adhesion monitoring system

Eutectogel was sandwiched between double-layer stainless steel plates and bonded under high-temperature conditions. The assembly was subsequently cooled to room temperature to promote crystallization, resulting in a robust composite supercapacitor. The electrical performance of the supercapacitor, specifically its capacitance and resistance under mechanical deformation, was simultaneously monitored using a digital bridge (Tonghui TH2832) in conjunction with a tensile testing machine.

For remote data acquisition, a Bluetooth-enabled capacitance monitoring module, developed around a customized ST32 microcontroller, is employed. This setup allowed real-time wireless transmission of measurement data to a mobile device for continuous off-site monitoring.

### Fabrication of the gripper

A smart adhesive gripper was fabricated by integrating a eutectogel with a dedicated temperature control system. This system comprised a 24 V, 16 W polyimide low-voltage heating film and a 5 V, 10 W semiconductor thermoelectric cooler. The full assembly enabled rapid and reversible temperature switching between 0 and 100 °C.

### Fabrication of the three-legged climbing robot

The primary structure of the three-legged climbing robot was designed using 3D modeling software and fabricated via 3D printing. Each foot consisted of a four-layer architecture comprising, from bottom to top, a polylactic acid (PLA) structural layer, a silicone insulation layer, a metal conductive layer, and a phase-change gel layer. The biaxial gear motor, drive module, and onboard camera were mounted on the central flat foot. The three feet were mechanically coupled through three interconnected crankshafts.

## Supplementary information

Supplementary Information

Description of Additional Supplementary Files

Movie S1

Movie S2

Movie S3

Movie S4

Movie S5

Movie S6

Movie S7

Movie S8

Movie S9

Movie S10

Movie S11

Movie S12

Movie S13

Movie S14

Movie S15

Transparent Peer Review File

## Source data

Source Data

## Data Availability

The authors declare that all data supporting the findings of this study are available within the article and its Supplementary Information file. Source data are provided with this paper. All data are available from the corresponding author upon request. Source data are provided with this paper.

## Code availability

The generated codes for machine-learning-assisted adhesion sensing during this study are available from the corresponding author upon request or at https://gitee.com/DongHYang/adhesive-analysis.
