# Supplementary material for: Switchable adhesion of phase-transition eutectogels with integrated machine learning-enhanced intelligent adhesion sensing
Source: Nat Commun. 2026 Jun 11;17:7434. doi: 10.1038/s41467-026-74275-7 (PMC13408668; doi:10.1038/s41467-026-74275-7)
Supplement: Supplementary file 1 — Supplementary Information [file 41467_2026_74275_MOESM1_ESM.pdf]

Supplementary Information for

Switchable adhesion of phase-transition eutectogels with integrated machine learning-enhanced intelligent adhesion sensing

JiaQing He,[1†] JiaHao Li,[1†] HanYang Dong,[1,2] DeYun Chen,[1] ChangHong Linghu,[3] Qiang Zhou,[4] YinBo Zhu,[1] ShuRong Sheng,*[2] HengAn Wu,*[1,5] Wei Feng*[1]

Affiliations

[1] Institute of Humanoid Robots, Department of Modern Mechanics, University of Science and Technology of China, Hefei 230027, China

[2] Institute of Artificial Intelligence, Hefei Comprehensive National Science Center, Hefei 230088, China

[3] Department of Mechanical Engineering, College of Engineering, City University of Hong Kong 999077, China

[4] Chemistry Experiment Teaching Center, School of Chemistry and Materials Science, University of Science and Technology of China, Hefei 230026, China

[5] State Key Laboratory of Nonlinear Mechanics, University of Science and Technology of China, Hefei 230027, China

Corresponding Email: fengw@ustc.edu.cn, wuha@ustc.edu.cn, shengshurong@iai.ustc.edu.cn

This PDF file includes:

Supplementary Text
Tables S1 to S6
Figs. S1 to S65
Movies S1 to S15
References (1 to 29)

Supplementary Note 1

Due to the limit of text numbers, we elaborate on the preparation and properties of eutectogels here in the supplementary text.

The deep eutectic solvents (DES) prepared at different molar ratios exhibit distinct melting points. These values display a non-monotonic trend with increasing urea content, initially decreasing before rising again, reaching a minimum of 12 °C at a urea : choline chloride (U:C) = 2:1 molar ratio. Consequently, only the DES with a U:C=2:1 ratio remains liquid at room temperature, while the others exist as solid crystalline phases. The crystallization process of the eutectic solvent was further examined using polarized light microscopy. Upon cooling, the initially liquid solvent at room temperature gradually developed elongated needle-like crystals.

To formulate eutectogels, polymerizable monomers were introduced into the eutectic solvent system, where they functioned as hydrogen bond donors. Specifically, amphiphilic quaternary ammonium monomers capable of providing physical cross-linking were selected as polymerizable hydrogen bond acceptors, thereby minimizing interference from polymer chains with solvent crystallization. A series of P(HEAA-*co*-OMA) eutectogels was synthesized via photopolymerization at 60 °C by incorporating N-hydroxyethyl acrylamide (HEAA), acrylamido octadecyldimethylammonium bromide (OMA), and the photoinitiator IR2959 in specified ratios into the urea and choline chloride-based eutectic solvent.

The crystal growth within the eutectogel was monitored in real time using polarized optical microscopy. As the temperature decreased, needle-like crystals nucleated and progressively proliferated, eventually encompassing the entire gel matrix. The melting point of the resulting eutectogel depends strongly on the molar ratio of urea to choline chloride. When the urea content is less than or equal to that of choline chloride, the eutectogel appears opaque and crystalline at room temperature; conversely, a transparent amorphous gel is formed when urea is in excess. At certain high temperatures, both types transition into a transparent amorphous state.

Crystalline eutectogels are rigid at low temperatures, which dissipates upon heating. In contrast, amorphous eutectogels maintain flexibility across both high and low temperatures. Notably, the crystallization time during cooling is inversely proportional to the choline chloride content: higher choline chloride concentrations result in faster crystallization. Time-resolved microscopy further confirms the rapid growth of needle-like crystals at low temperatures.

By varying the solvent-to-polymer ratio, it was observed that reducing the proportion of eutectic solvent suppresses crystallinity. The solvent within the eutectogel demonstrates phase behavior analogous to water freezing in hydrogels, with increased polymer content leading to diminished crystal formation, indicating a synergistic interaction between the polymer network and the eutectic solvent.

The crystallinity of eutectogels with different deep eutectic solvents (DES) contents was quantitatively analyzed using X-ray diffraction (XRD). The intensity of sharp diffraction peaks gradually decreased with increasing PHEAA content. To quantitatively describe the crystallization behavior, the degree of crystallinity at various compositions is evaluated via JADE software.

$$Xc = \frac{Ic}{Ic+Iw} * 100\% \quad (1)$$

where I_C and I_W are the crystal diffraction peak intensity and amorphous scattering peak intensity, respectively. The analytical results show that as the eutectic content decreased from 90% to 30%, the relative crystallinity also decreased.

Supplementary Table 1. Composition of eutectogel formulations with different monomer contents

Sample	HEAA	OMA	Photo-initiator	DES solvents
**EG_{1-25}**	2.5g	0.3g	0.025g	7.2g
**EG_{1-30}**	3.0g	0.3g	0.030g	6.7g
**EG_{1-35}**	3.5g	0.3g	0.035g	6.2g
**EG_{1-40}**	4.0g	0.3g	0.040g	5.7g
**EG_{1-45}**	4.5g	0.3g	0.045g	5.2g

Supplementary Table 2. Control parameters of eutectogel intelligent gripper for grasping target objects.

Gripper compose	Control parameters	
Synthesis information of eutectogel	$EG_{0.67\text{-}30}$	HEAA: 30 wt%<br>U/C = 1:1.5
Eutectogel size	25 mm × 25mm × 2 mm	
Heating system	Brand	XinKai electrical appliance PI heating film 001
	Size	50 mm×50 mm
	Operating voltage and current	Maximum power: 24 V, 16 W<br>Actual power: 20 V, 0.5 A
Cooling system	Brand	Ice Lord radiator<br>Silent semiconductor heat sink 008
	Size and weight	50 mm×50 mm<br>100 g
	Operating voltage and current	Voltage: 5 V<br>Current: 2 A/ 3 A
Heating time	15-20 s	
Cooling time	20-30 s	

By comparing various types of grippers recently reported across multiple dimensions—including adhesion force, surface adaptability, grasping time, and energy consumption—grippers constructed from eutectogels based on crystalline transformation demonstrated advantages such as high adhesion force and broad adaptability **(Supplementary Table 3)**.

Supplementary Table 3. Performance comparison of adhesion-based intelligent grippers

Gripper type	Preload (N)	Gripper diameter (mm) or area	Max pull - off force (N)	Max lifting ratio	Diameter ratio limits	Surface conditions(surface roughness)	Time to acquire grip (s)	Power required to maintain grasp (W)	Reference
Granular jamming	150	86	100	N/A	0.1-0.85	N/A	0.1-1.1	N/A	10
Gecko-inspired gripper	N/A	180	43	200	> 0.5	N/A	<0.1	0	11
Elastomeric microfibrillar soft adhesive gripper	N/A	18	78 mN	N/A	> 1	Need relatively smooth surface	N/A	4.1 J/m^2 3.3 J/m^2	12
Magnetically switchable soft suction	1.5	20	7.5	80	>0.4	17.7	10	0	13
shape memory polymer gripper	N/A	7 – 60 (Depends on the shape and size of the shape memory polymer)	34.8	78.5	< 0.01 > 1	1 μm - 1 cm	N/A Depends on the heating time	0	14
Tensile-induced buckling gripper	65% stretching strain	240 x 40	0.012	N/A	< 0.5	N/A	N/A Depends on the stretching time	N/A The tensile deformation must be maintained.	15
Finger inspired rigid-flexible-soft structure	12	31 cm	4.0	0.11 (Two-finger clamp) 0.128 (Three-finger clamp)	0.806	N/A	3	N/A Depends on the gas actuator power	16
supramolecular gel-elastomer system	0	16 × 16	0.54 (2.12 kPa)	671	< 1	0.01-10	30	N/A 300 V~ 1 kV	17
Curved loop strip soft gripper	0	N/A Depends on the length of the strip		1.7×10^4	< 1	N/A	1.76	N/A	18
Magnetoactive bistable soft gripper	0	N/A	0.029 N	N/A	< 1	N/A	10	N/A	19
Our work	0 – 5 N	2 cm Depends on the length of the eutectogel size	100 N	88.23	> 1	0.01- 1 cm	10 – 20 Depends on the heating time	20V* 0.5 A Depends on the heating time	

Supplementary Table 4. Recent reports on climbing robots with intelligent adhesion

Climbing robots: adhesion mechanism	Adhesive material	Adhesion Switching Performance	Wall material (Whether it adapts to different surface)	Wall Roughness (Whether suitable for rough surfaces)	Crawling speed	Payload (payload-to-weight ratio)	Energy density	Reference
Pneumatic	Ecoflex	6-8N	glass, sand paper, Acrylics	Smooth and semi-smooth wall (roughness <20 μm)	2.86 cm/min	40g(self mass) 200 g (5 times body weight)	N/A	20
Dielectric elastomer	Dielectric elastomer VHB	3.0533 N (32mm Diameter) 5.09 kPa – 0 kPa	Wood, paper, glass	Exact roughness value unknown, relatively flat.	530.76 cm/min (0.75 body lengths)	10 g	V = 6 kV 23 Hz	21
Electrochemical	Borate ester hydrogels	11.5 kPa - 0 kPa	Multiple materials: glass, metal, ceramics, plastics	Exact roughness value unknown, relatively flat	7.8 cm/min (0.8 body lengths)	175 g (self mass)	V = 3 V 5 s	22
Electro-permanent magnet	Permanent magnet	535.4 N (41*41mm^2) 318.26 kPa-0kPa	Ferromagnetic	Tolerant to rough surface	4200 cm/min (2.12 body lengths)	8 kg (self mass) 2 kg (0.4 body weight)	V = 29.6 V I = 35.18 A 4.07 J	23
Mushroom-shaped dry adhesion and microspike bioadhesive	PDMS and chitosan hydrogel	1 mN (0.3 mm^2) 3.3 kPa-0.33 kPa	Mucus tissue	Relatively smooth, roughness < 30 μm	N/A	20 times body weight, 3 times volume	N/A	24
Growing-climbing mechanism	Microstructured biofilms	7-10 kPa	Multiple materials	Tolerant to rough surface	N/A	521.1g 1330g (1.55 body weight)	N/A	25
Water-enhanced sliding suction mechanism	Water-silicone (capillary forces)	N/A	Polyoxymethylene, PMMA, aluminium alloy	Exact roughness value unknown, relatively flat.	114 cm/min (0.475 body length)	96 g (self mass) 1 kg (10.42 body weight)		26
Electroadhesive	Electroadhesive footpad	3N (4 kV , 39*84 mm^2) 0.92 kPa	Multiple materials	Exact roughness value unknown, relatively flat.	6-90 mm (self length) 24.6 cm/min (2.73 body length)	0.2-3 g(self mass)	V = 3 kV	27
Magnetic-controlled adhesion	magnetically triggered MR elastomers	1.35 N/cm^2-2.93N/cm^2 29.3kPa	Multiple materials: Ecoflex, silicon wafer, pigskin	Exact roughness value unknown, relatively flat.	N/A	N/A	0 T-0.36 T I = 9 A	28
Directional microprismatic structure bioadhesion	PDMS Microstructural friction	<70kPa	Multiple materials	Exact roughness value unknown, relatively flat.	1.4 body length /s (84 body length /min)	0.63g and 0.24g (self mass)		29
Our work	Phase changing eutectogels	Crstal state:10 kPa Melt state: 100kPa M-C state: 1000kPa	Multiple materials, including metals, plastics, ceramics, glass, etc.	Tolerate to rough surfaces, roughness can be up to millimeters.	8cm/min (0.65 body lengths)	500g (self mass) 200g (0.4 body weight)	V = 6 V I = 2 A >10 s	

Supplementary Table 5. Parameters of two-axis geared drive motors used in the wall-climbing robot.

Essential parameter	
Product name	DC dual-axis reduction motor
Brand	Chiming motor
Gearbox size	46 mm by 32 mm by 22.5 mm
Axle size	5.5 mm by （14.5 +14.5 mm）
Operating voltage	12 V
Rotation speed	0.055 sec/360 degree(Adjustable)
Reduction ratio	340:1
Rated torque and torque capacity	7 kgf.cm<br>16 kgf.cm
Rated current and Full load current	0.4 A<br>1.4 A
Gear type	metal

Supplementary Table 6. Control parameters of wall climbing robot.

Climbing robot compose	Control parameters	
Synthesis information of eutectogel	$EG_{0.67\text{-}30}$	HEAA: 30 wt%<br>U/C = 1:1.5
Eutectogel size	50 mm × 25 mm × 2 mm	
Heating system	Product name	Brand: Bo Xiang<br>Nichrome heating wire
	Size (diameter)	0.6mm
	Operating voltage and current	Foot A: 6 V, 2 A<br>Foot B: 12 V, 4 A
Drive motor	Brand	Chiming motor
	Size	Table S5
	Operating voltage and current	Voltage: 5 V<br>Current: 2 A/ 3 A
Heating time	15-20 s	
Cooling time	20-30 s	

Calculating the energy consumption for one complete cycle

First, the energy consumption for a single actuator (leg) in one action is calculated as follows: voltage 6V multiplied by current 2A, multiplied by the action duration of 15 seconds, resulting in 6 V × 2 A × 15 s = 180 J.

The total energy consumed by three actuators in one action is 180 J × 3 = 540 J.

Since one complete cycle consists of two actions, the total energy consumption is 540 J × 2 = 1080 J.

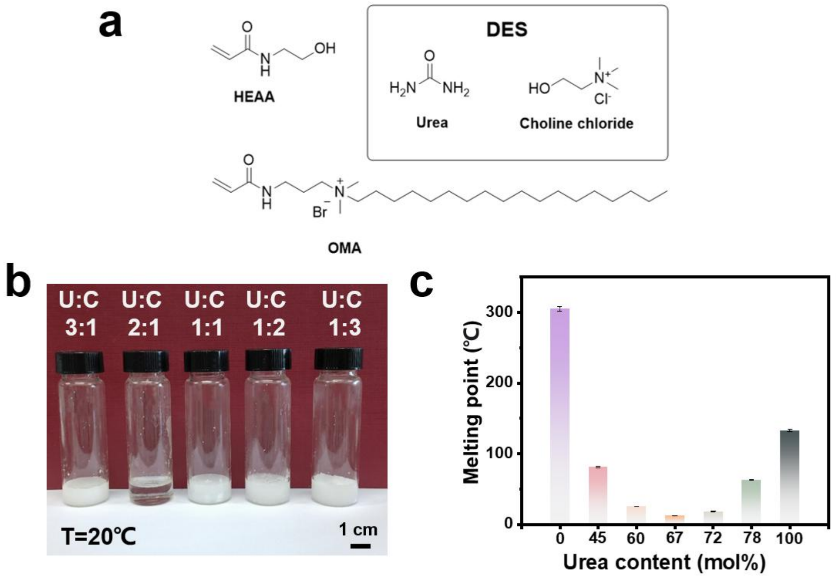

Supplementary Fig. 1. Deep eutectic solvent (DES) and monomers. (a) Chemical structures of DES and monomers. (b) Urea/choline chloride eutectic solvents at different molar ratios at room temperature. Only the U:C=2:1 DES is liquid. (c) Melting points of eutectic solvents at different urea contents (error bar: Standard deviation of melting point test data for three groups).

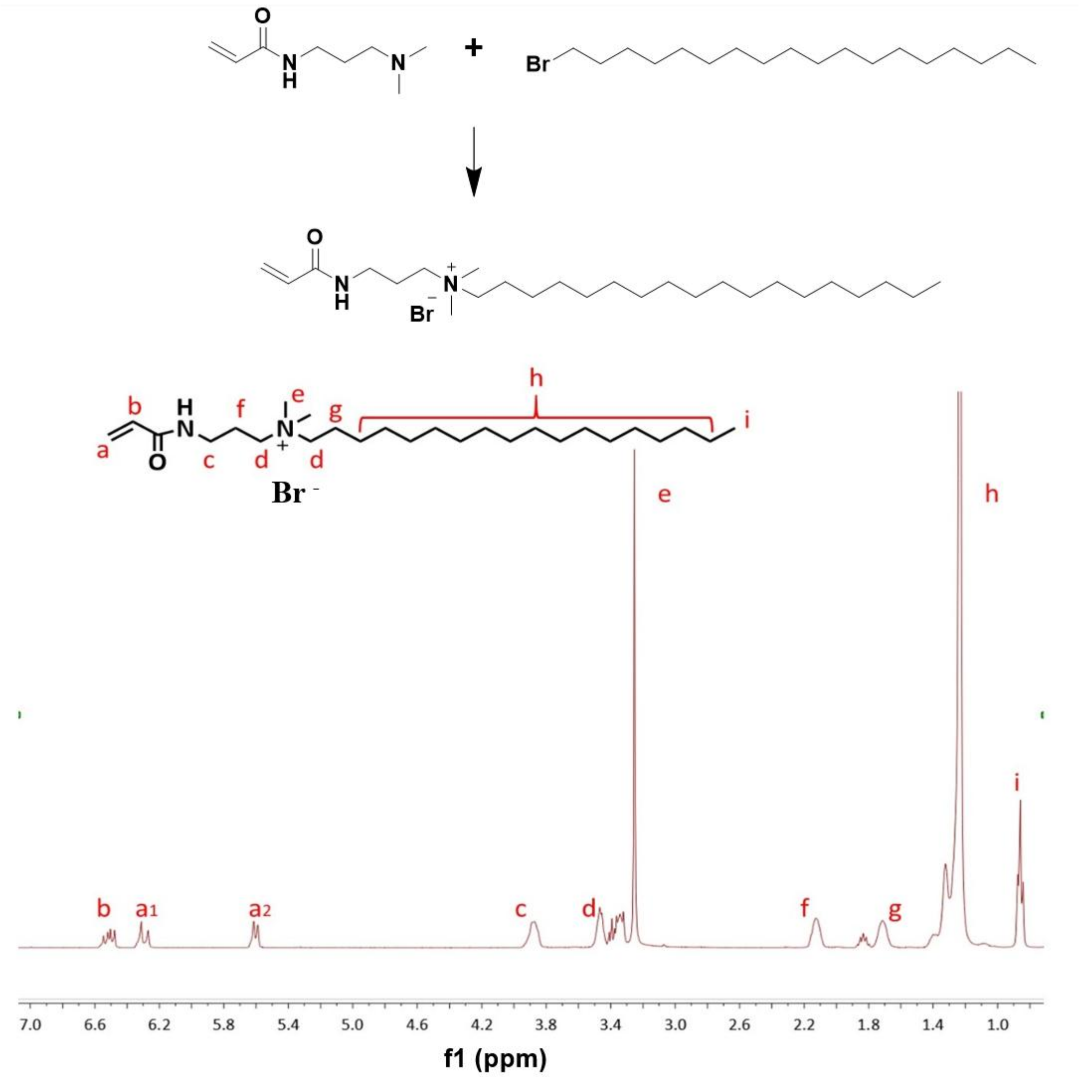

Supplementary Fig. 2. Synthesis procedure and H^1-NMR spectra of octadecyldimethylaminopropyl acrylamide monomer ($CDCl_3$, 400MHz).

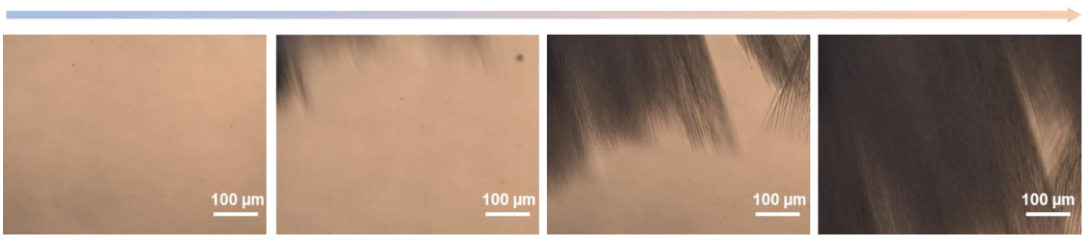

Supplementary Fig. 3. Microscopic images showing the crystallization of eutectic solvents.

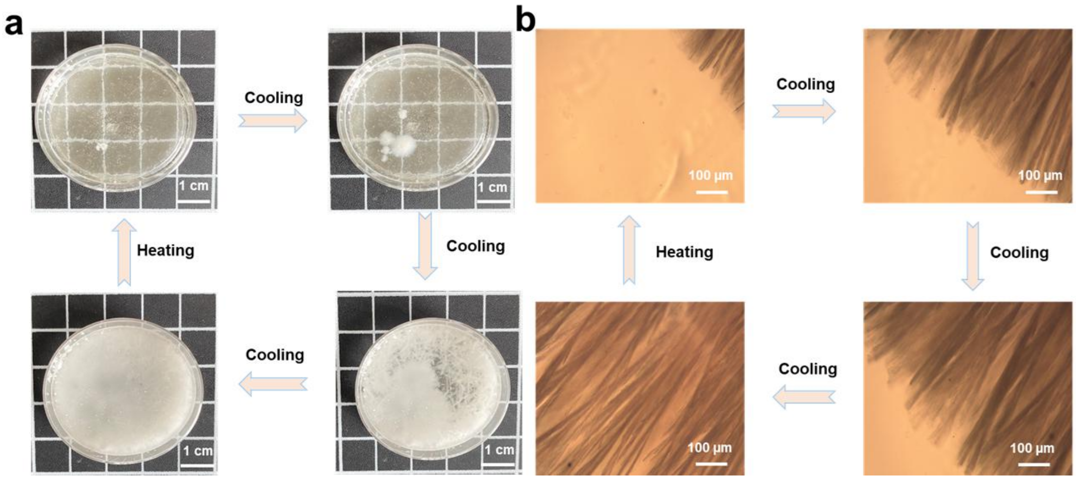

Supplementary Fig. 4. Demonstration of the cooling and crystallization process of eutectogel. (a) Images of eutectogel $EG_{1\text{-}30}$ during temperature change. (b) Microscopic images of crystallization and melting of eutectogel $EG_{1\text{-}30}$.

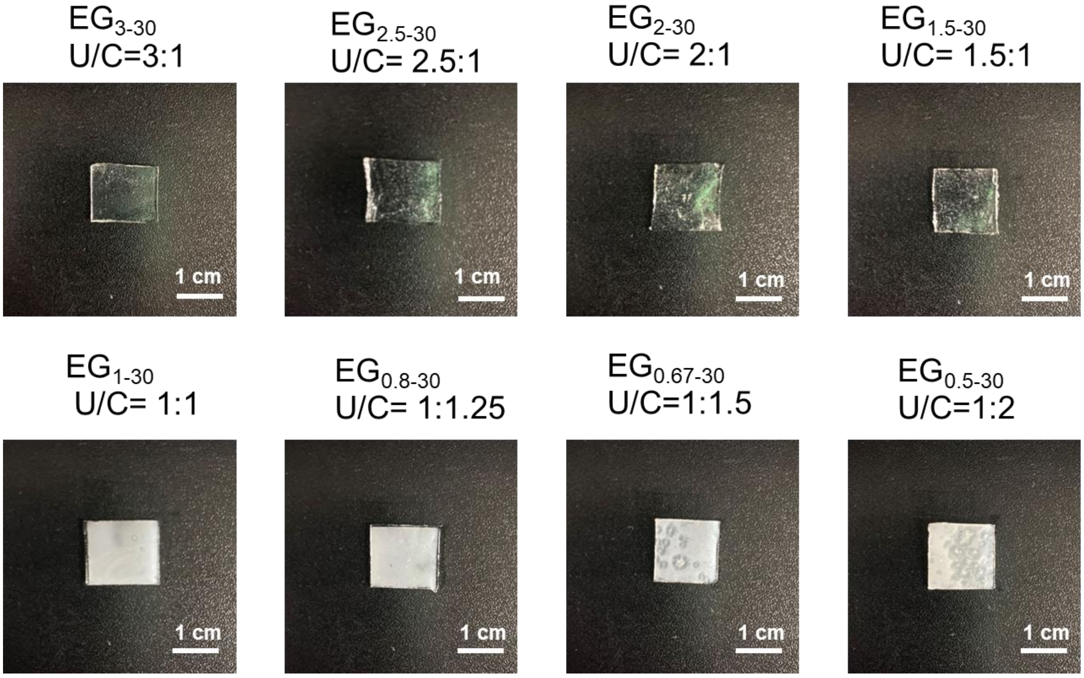

Supplementary Fig. 5. Crystallization of eutectogels with different DES. The eutectogel crystallizes at room temperature when the molar ratio of urea to choline chloride is less than 1:1. When the molar ratio of urea and choline chloride is greater than 1:1, the eutectogel does not crystallize at room temperature.

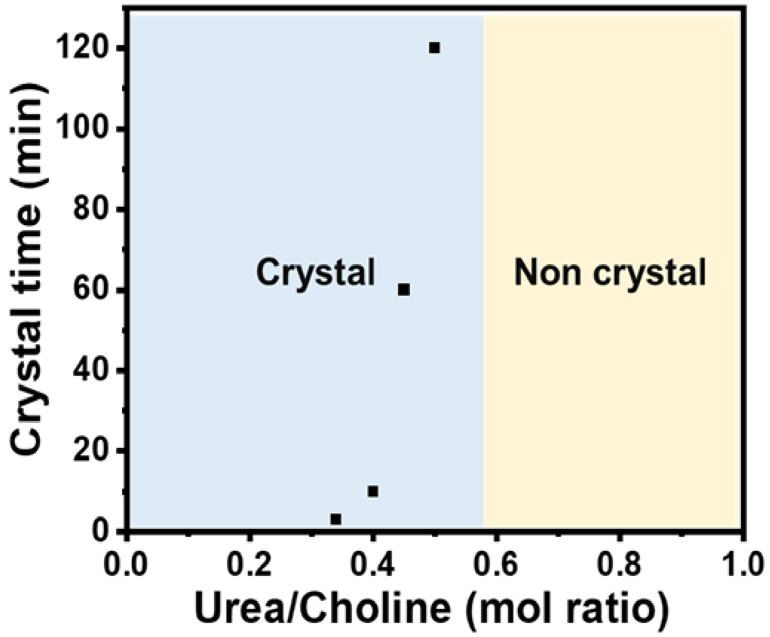

Supplementary Fig. 6. Crystallization time of eutectogels with different U:C ratios in DES. The higher the content of choline chloride, the faster the eutectogel crystallizes.

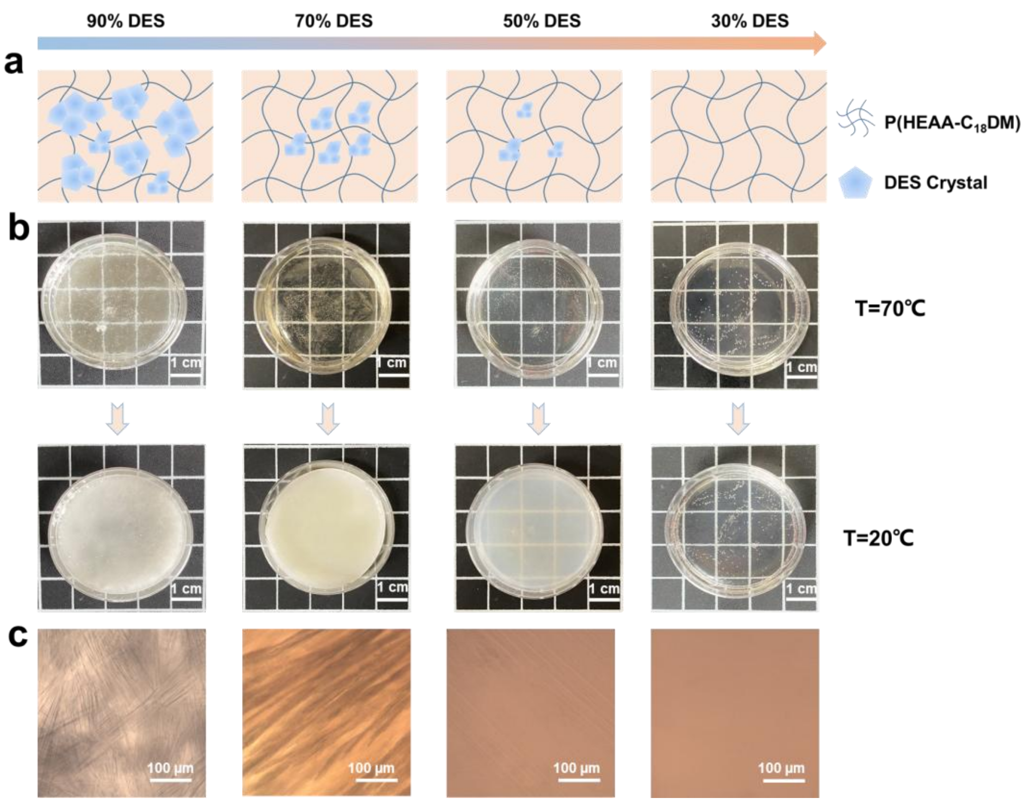

Supplementary Fig. 7. Crystallization of eutectogel made from different monomer compositions. (a) Schematic illustration of the crystallization process, (b) eutectogel at high (70°C) and low (20°C) temperatures, (c) microscopic images of eutectogel at room temperature (20 °C). The increase in monomer solid content significantly limits solvent crystallization (sample information: EG_{1-y}).

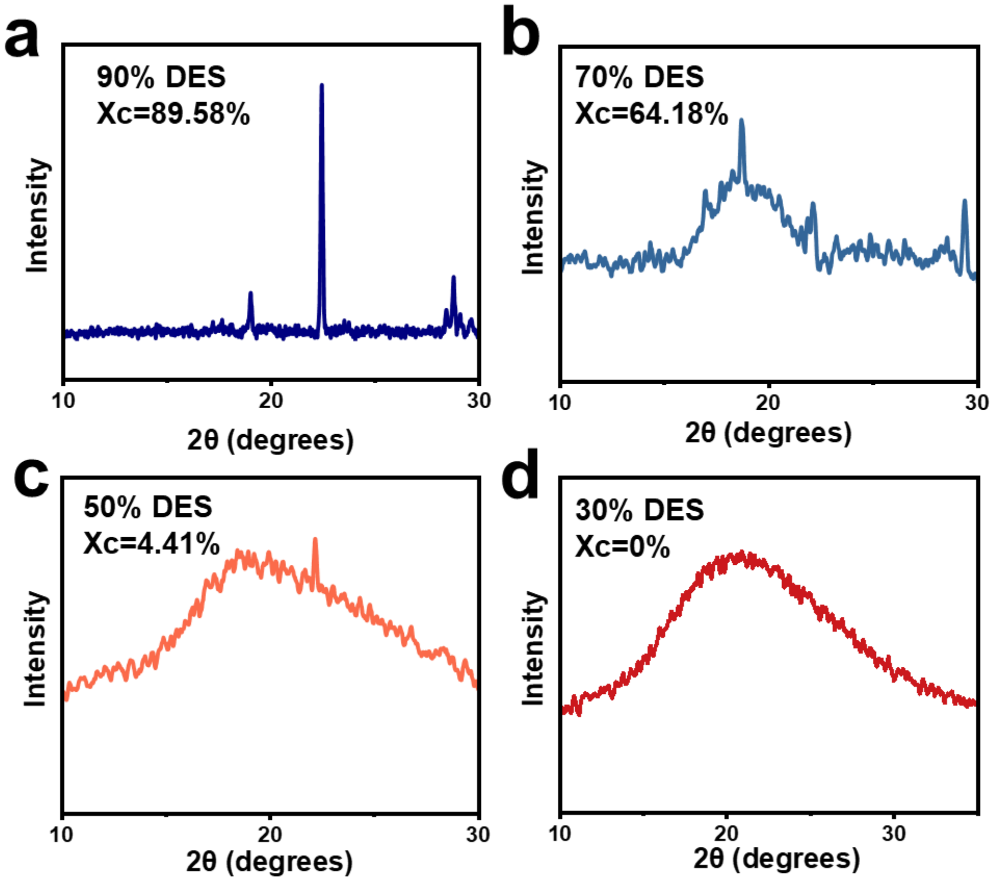

Supplementary Fig. 8. The effect of concentrations of deep eutectic solvents on the crystallisation of eutectogels. (a) The crystallinity of the eutectogel at 90% wt DES is 89.58%, (b) the crystallinity of the eutectogel at 70% DES is 64.18%, (c) the crystallinity of the eutectogel at 50% DES is 4.41%, and (d) the crystallinity of the eutectogel at 30% DES is 0%. The crystallinity is calculated using Jade software based on XRD patterns (sample information: EG_{1-y}).

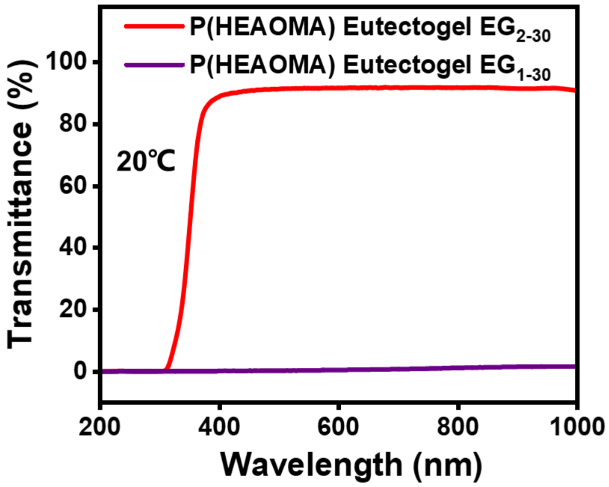

Supplementary Fig. 9. UV spectra of non-crystalline and crystalline eutectogels. In the visible light range (400-800 nm), non-crystalline eutectogel $EG_{2\text{-}30}$ is transparent, while crystallized eutectogel $EG_{1\text{-}30}$ is opaque.

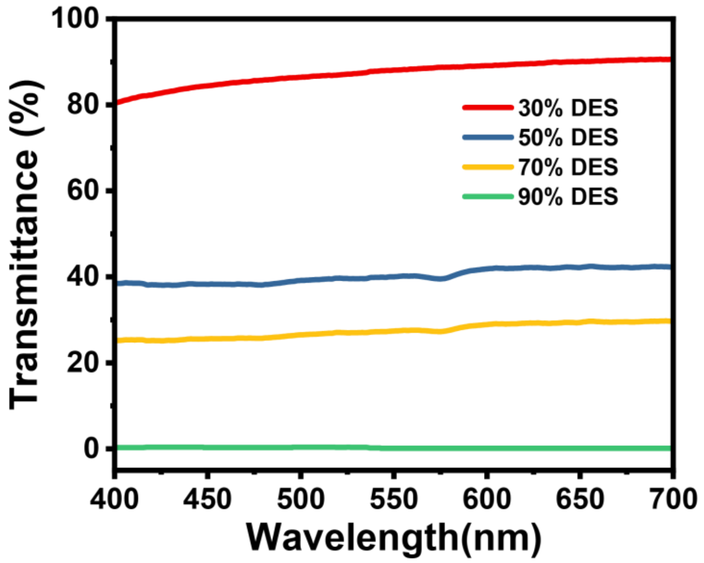

Supplementary Fig. 10. Ultraviolet-visible spectra of P(HEAOMA) crystalline eutectogels made from different compositions (sample information: EG_{1-y}).

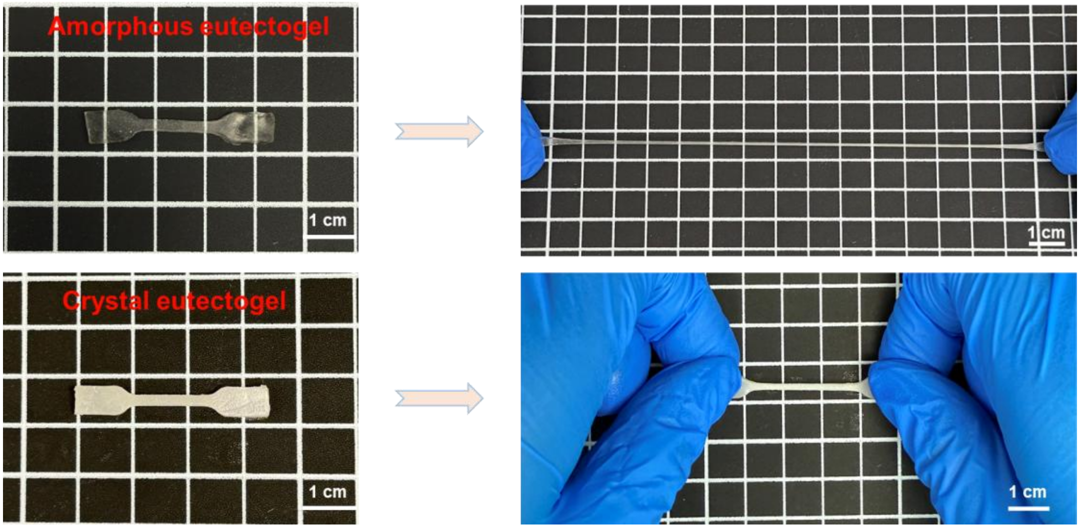

Supplementary Fig. 11. Crystalline eutectogel $EG_{1\text{-}30}$ and amorphous eutectogel $EG_{2\text{-}30}$ while stretching.

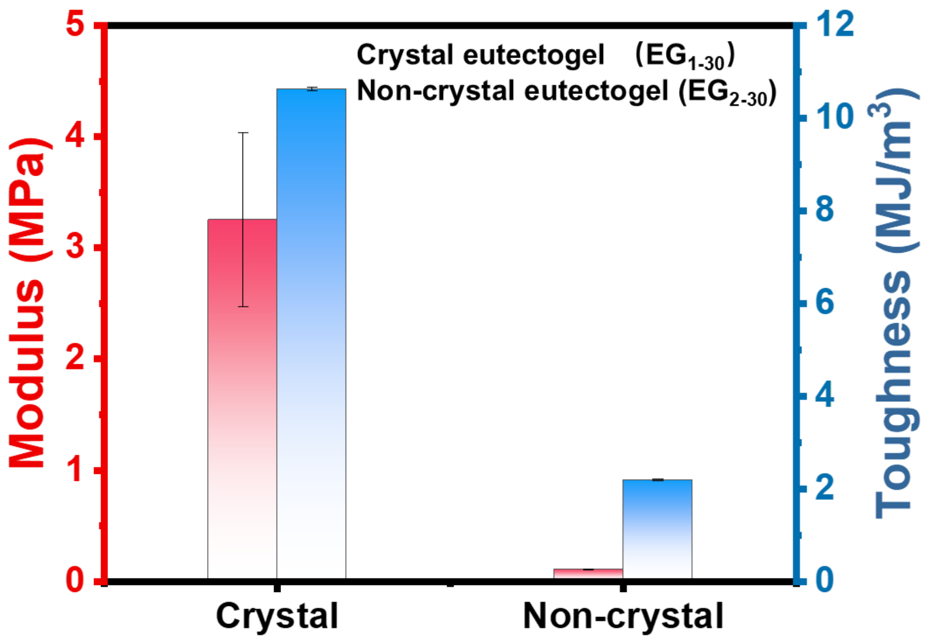

Supplementary Fig. 12. Comparison of modulus and toughness of crystalline eutectogel EG_{1-30} and non-crystalline eutectogel EG_{2-30}.(error bar: Standard deviation of the test data for the three groups)

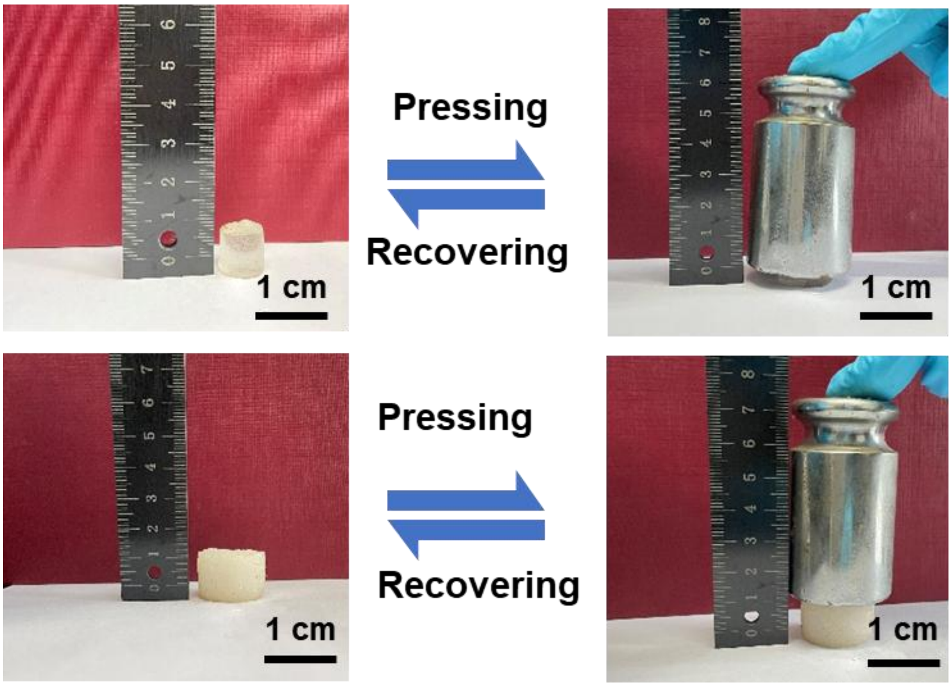

Supplementary Fig. 13. Compression tests of amorphous eutectogel EG_{2-30} and crystalline eutectogel EG_{1-30}.

Supplementary Note 2

To further compare the mechanical properties of the two types of eutectogels, cyclic tensile tests were conducted under large-strain conditions. Both gels were subjected to ten consecutive cycles at 500% tensile strain without inter-cycle recovery intervals.

Significant hysteresis was observed during the initial loading–unloading cycle. As shown in Fig. S15a, the crystalline eutectogel exhibited high mechanical strength in the first cycle, attributable to its crystalline microstructure. However, a substantial decrease in performance occurred between the first and second cycles, likely due to the irreversible disruption of crystalline domains under large strain and insufficient recovery time. From the second to the tenth cycle, the mechanical response stabilized with minimal further degradation.

The energy dissipation behavior, illustrated in Fig. S14b, further supports this interpretation. The dissipation energy reached 2.7 MJ/m³ in the first cycle, reflecting substantial energy absorption via crystal deformation and fracture. In subsequent cycles, the dissipation energy decreased from 446 kJ/m³ to 156 kJ/m³, indicating that although the crystalline structure enhances dissipation, its contribution diminishes after the initial yielding.

In contrast, the amorphous eutectogel (Fig. S15a) showed lower initial mechanical strength and similarly exhibited a noticeable drop between the first and second cycles, likely due to incomplete network recovery. Minimal further changes occurred in subsequent cycles. The dissipation energy of the amorphous gel (Fig. S16b) was significantly lower than that of its crystalline counterpart and remained relatively stable after the first cycle, decreasing only slightly throughout the remaining cycles.

These results highlight a pronounced difference in mechanical and dissipative properties between crystalline and amorphous eutectogels, underscoring the critical role of microstructure in governing their response to cyclic loading.

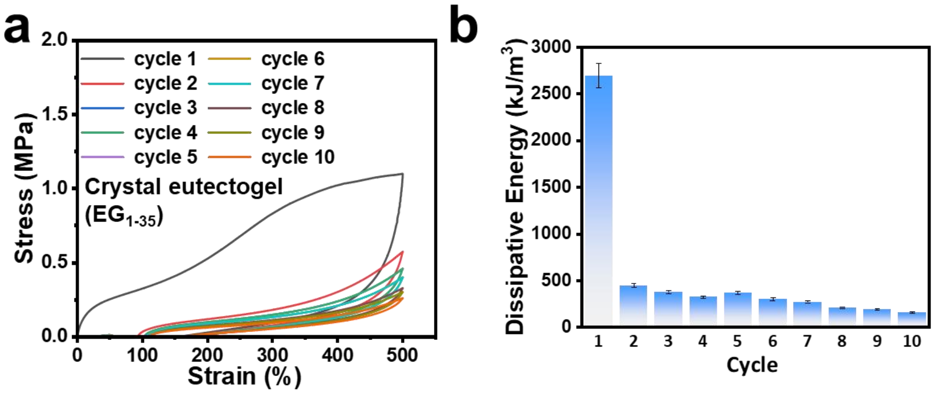

Supplementary Fig. 14. Mechanical tensile cycling of crystalline eutectogels. (a) Cyclic tensile curves of crystalline eutectogel EG_{1-35} at 500% strain. (b) Dissipation energy during cyclic tensile at 500% strain of crystalline eutectogel EG_{1-35} (error bar: standard deviation of the test data for the three groups).

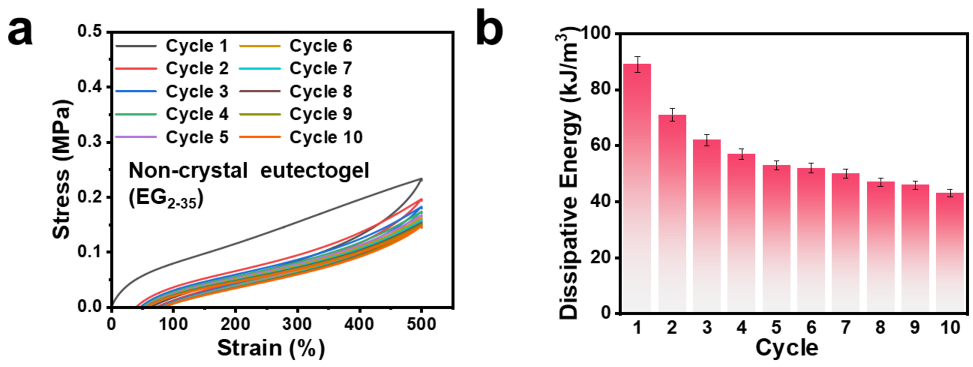

Supplementary Fig. 15. Mechanical tensile cycling of non-crystalline eutectogels. (a) Cyclic tensile test curves of non-crystalline eutectogel EG_{2-35} at 500% strain. (b) Dissipation energy during cyclic tensile tests at 500% strain of non-crystalline eutectogel EG_{2-35} (error bar: standard deviation of the test data for the three groups).

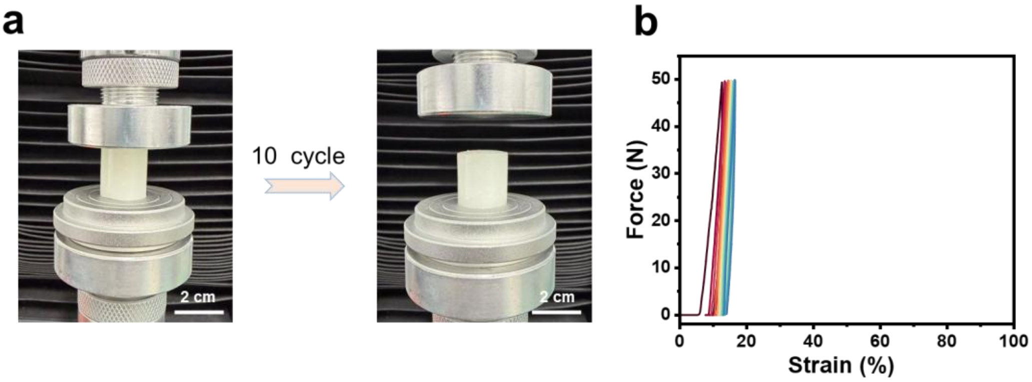

Supplementary Fig. 16. Behavior of crystalline eutectogels under external compression. (a) Comparison before and after ten compression cycles of sample EG_{1-30} under a constant stress of 50 N/25 MPa. (b) Strain change of sample EG_{1-30} after ten compression cycles at a constant stress of 50 N/25 MPa (strain = 8%).

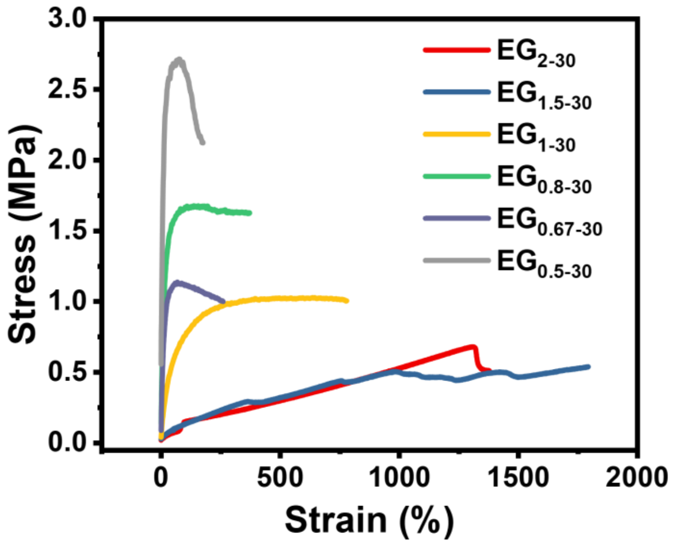

Supplementary Fig. 17. Effect of solvent composition on the tensile tests of eutectogels.

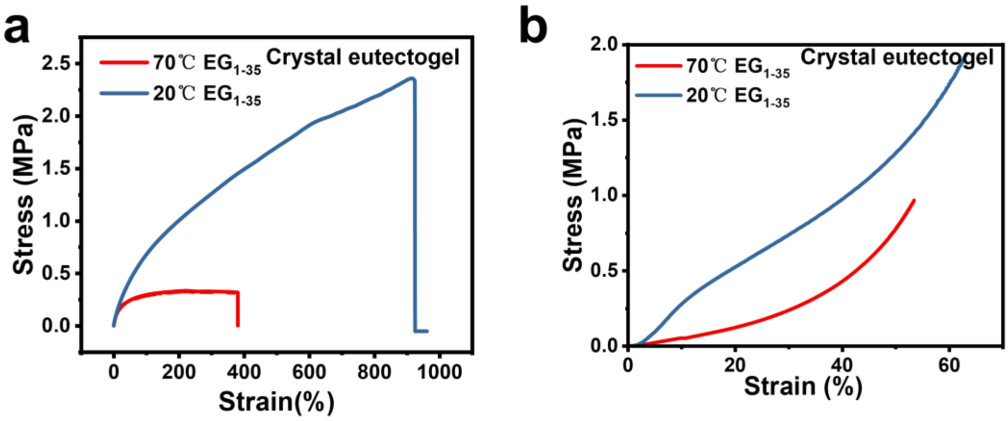

Supplementary Fig. 18. Mechanical behavior of crystalline eutectic gels at different temperatures. Tensile (a) and compressive (b) tests of eutectogel $EG_{1\text{-}35}$ at high (70 °C) and room temperatures (20°C).

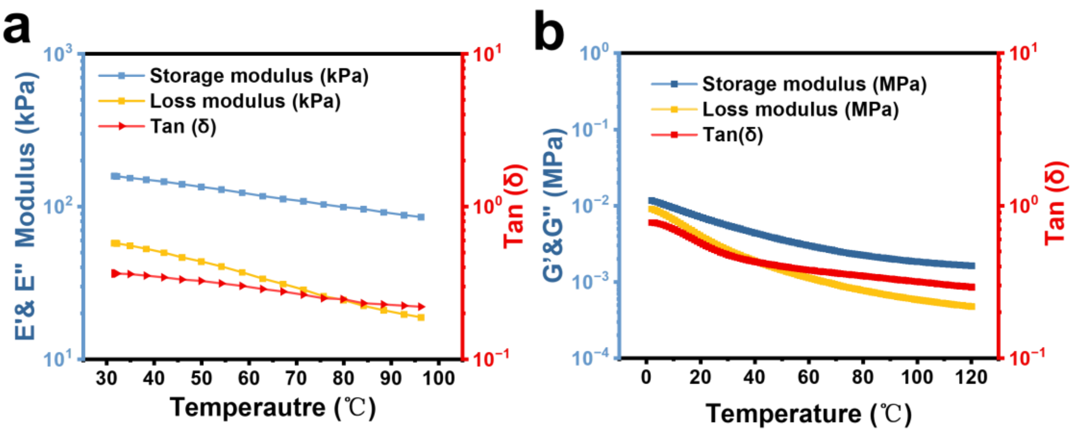

Supplementary Fig. 19. Changes in the modulus of amorphous eutectogels at different temperatures. (a) Dynamic mechanical analysis (DMA) and (b) rheological analysis of amorphous eutectogels of amorphous eutectogel $EG_{2\text{-}30}$.

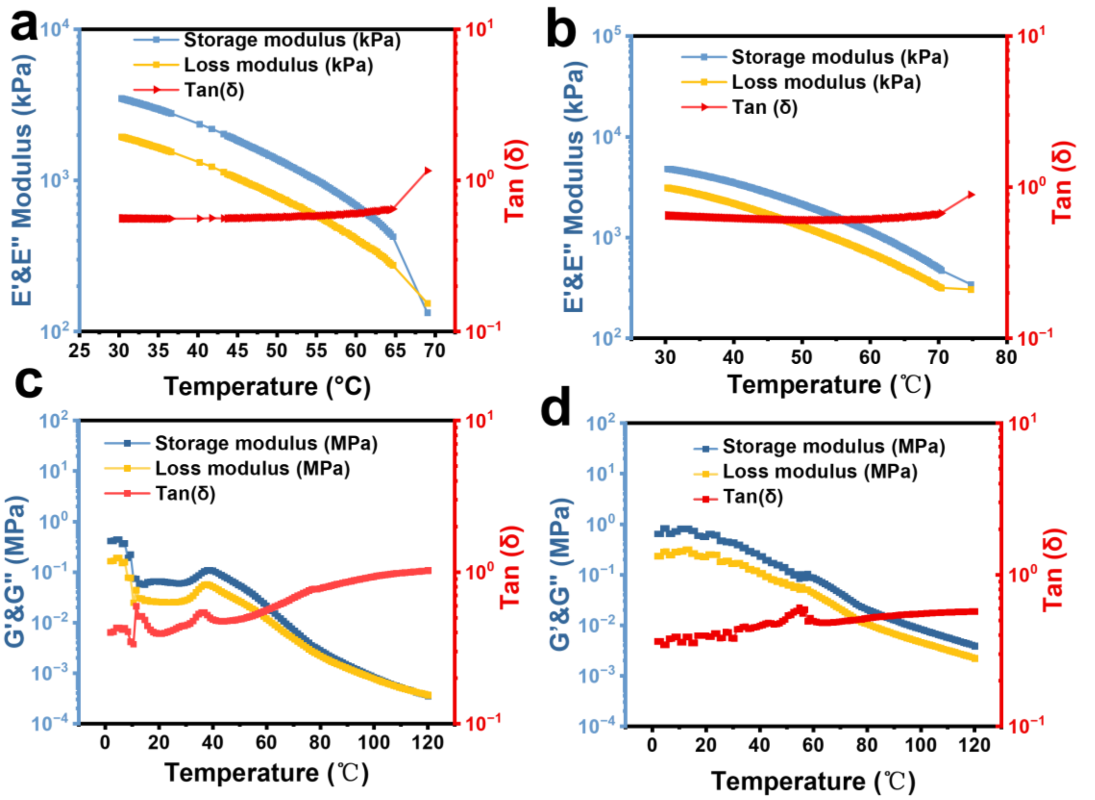

Supplementary Fig. 20. Changes in the modulus of crystalline eutectogels at different temperatures. (a) Dynamic mechanical analysis of eutectogel $EG_{0.67\text{-}30}$. (b) Dynamic mechanical analysis of eutectogel $EG_{0.5\text{-}30}$. (c) Rheological measurement of the eutectogel $EG_{0.67\text{-}30}$, showing a peak in tan δ at 38°C. (d) Rheological measurement of the eutectogel $EG_{0.5\text{-}30}$, showing a peak in tan δ at 58°C.

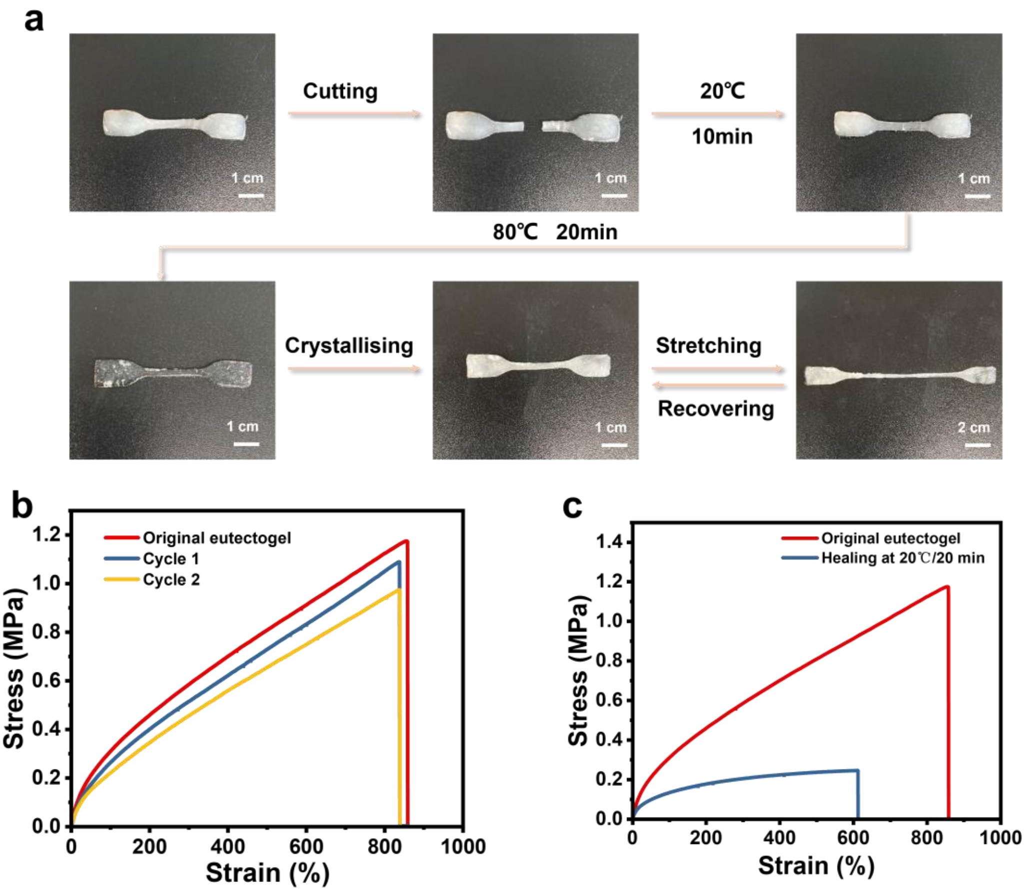

Supplementary Fig. 21. Self-healing process of eutectogel EG_{1-30}. (a) Self-healing process. (b) Tensile fracture curves of eutectogels after self-healing at 20°C for 20 min. (c) Mechanical properties after self-healing at 70°C for 20 min.

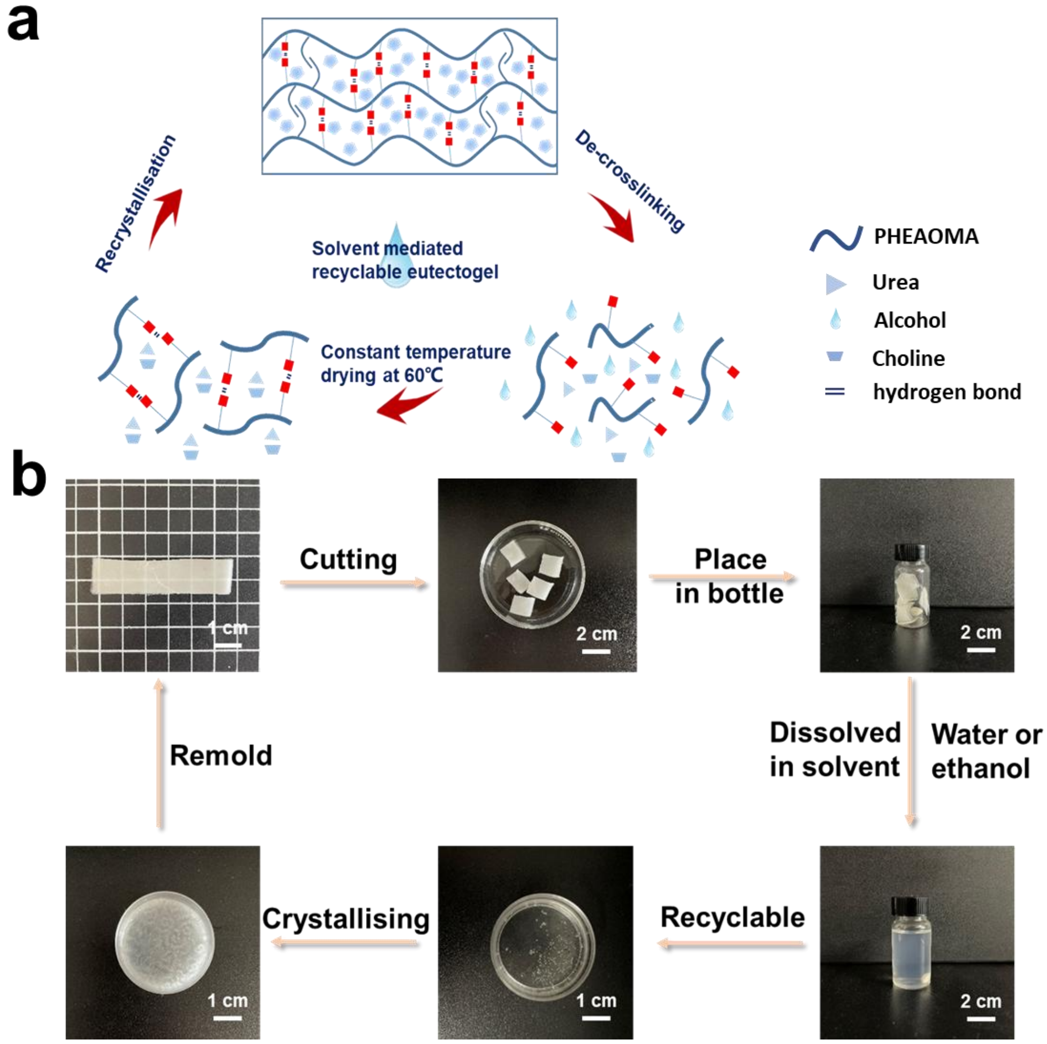

Supplementary Fig. 22. Recyclability of eutectogels. (a) Schematic diagram of eutectogel recovery process. (b) Recovery process of the eutectogel $EG_{1\text{-}30}$.

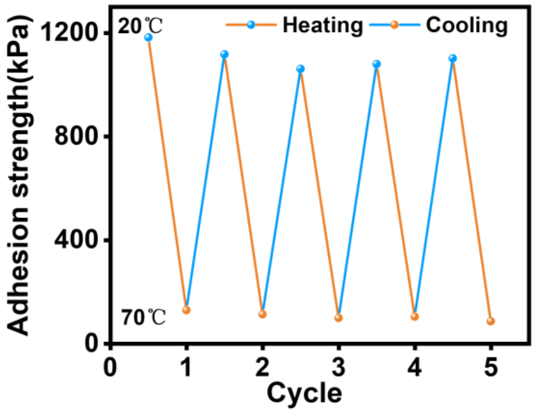

Supplementary Fig. 23. M2C adhesion cycle under temperature mediation (20°C-70°C). After five heating and cooling cycles, the adhesion performance exhibited negligible degradation (sample information: $EG_{0.67\text{-}30}$).

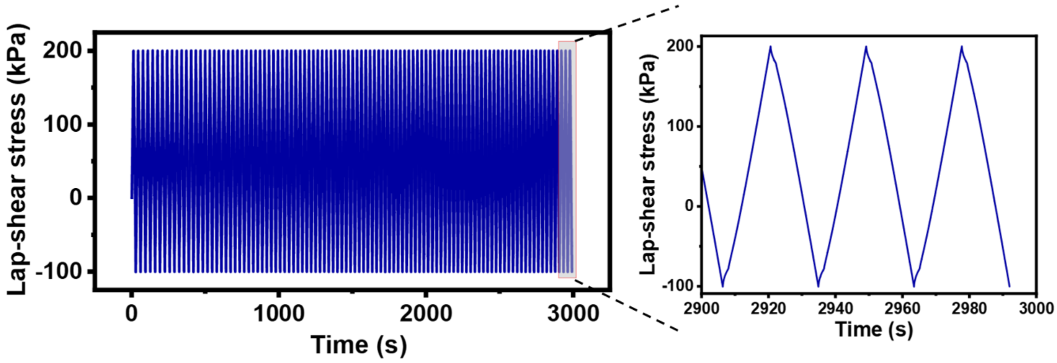

Supplementary Fig. 24. Repeated loading-unloading cycles of eutectogel $EG_{0.67\text{-}30}$ under tensile and shear stress of 200 kPa.

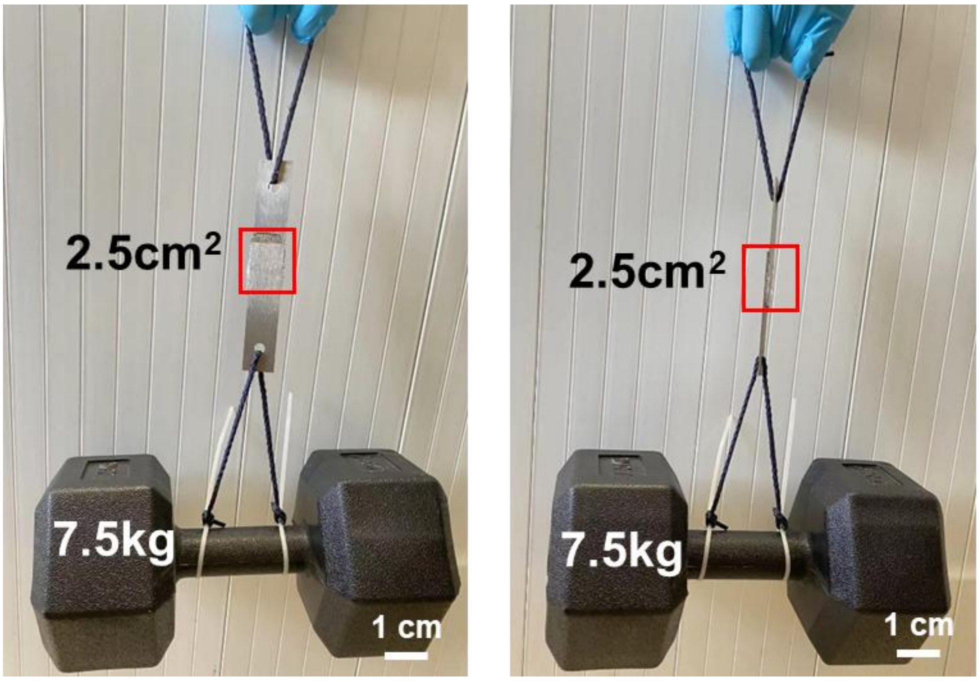

Supplementary Fig. 25. The eutectogel $EG_{0.67\text{-}30}$ demonstrates strong adhesion to stainless-steel substrates, supporting loads of up to 7.5 kg.

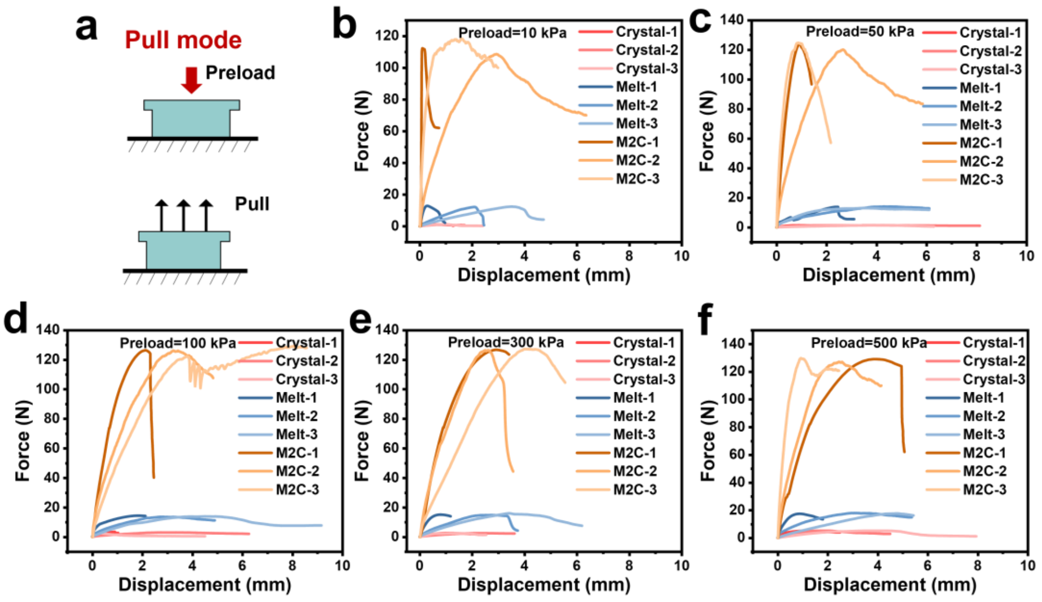

Supplementary Fig. 26. Force-displacement curves of eutectogel adhesion on glass substrates under 10–500 kPa preload in pull mode. (a) Schematic diagram of the adhesion process in pull mode. (b-f) Force-Displacement Curve at 10–500 kPa (measured on smooth glass, aspect ratio = 1) (sample information: $EG_{0.67\text{-}30}$).

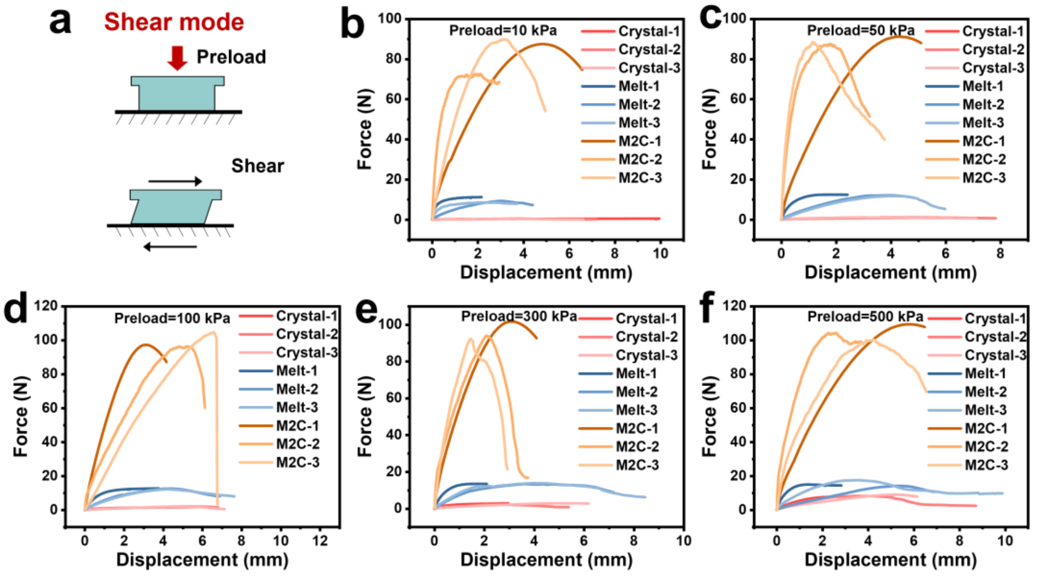

Supplementary Fig. 27. Force-displacement curves of eutectogel $EG_{0.67\text{-}30}$ adhesion on glass substrates under 10–500 kPa preload in shear mode. (a) Schematic diagram of the adhesion process in shear mode. (b-f) Force-displacement Ccurve at 10–500 kPa (measured on smooth glass, aspect ratio = 1).

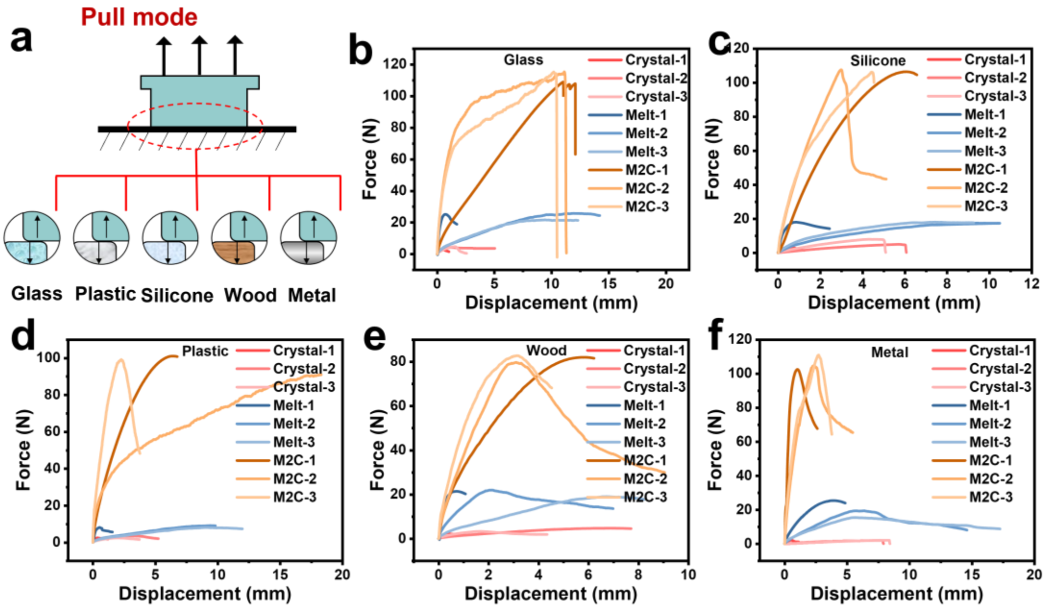

Supplementary Fig. 28. Force-displacement curves of adhesion of eutectogel $EG_{0.67\text{-}30}$ on different material surfaces during pull mode. (a) Schematic diagram of the adhesion process in pull mode. (b-f) Force-displacement curve at different surface (aspect ratio = 1 and preload = 200 kPa).

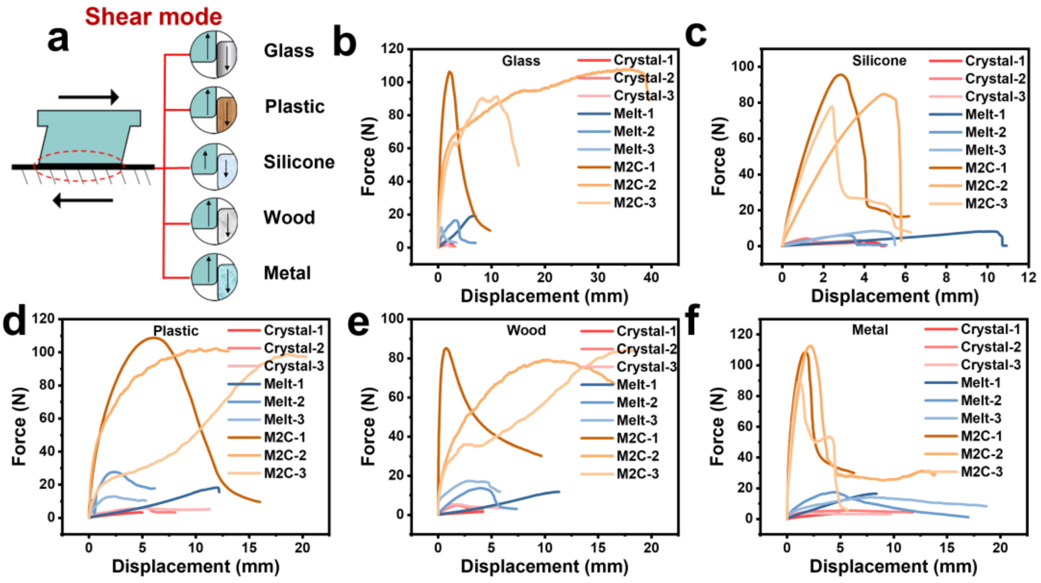

Supplementary Fig. 29. Force-displacement curves of adhesion of eutectogel $EG_{0.67\text{-}30}$on different material surfaces during shear mode. (a) Schematic diagram of the adhesion process in shear mode. (b-f) Force-displacement curve at different surface (aspect ratio = 1 and preload = 200 kPa).

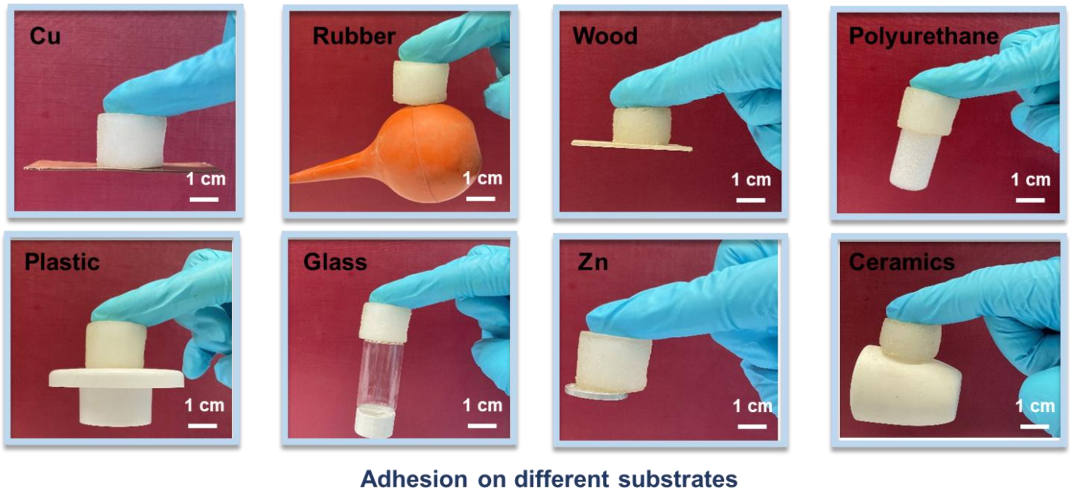

Supplementary Fig. 30. Eutectogels are adhesive to surfaces made of different materials (sample information: $EG_{0.67\text{-}30}$).

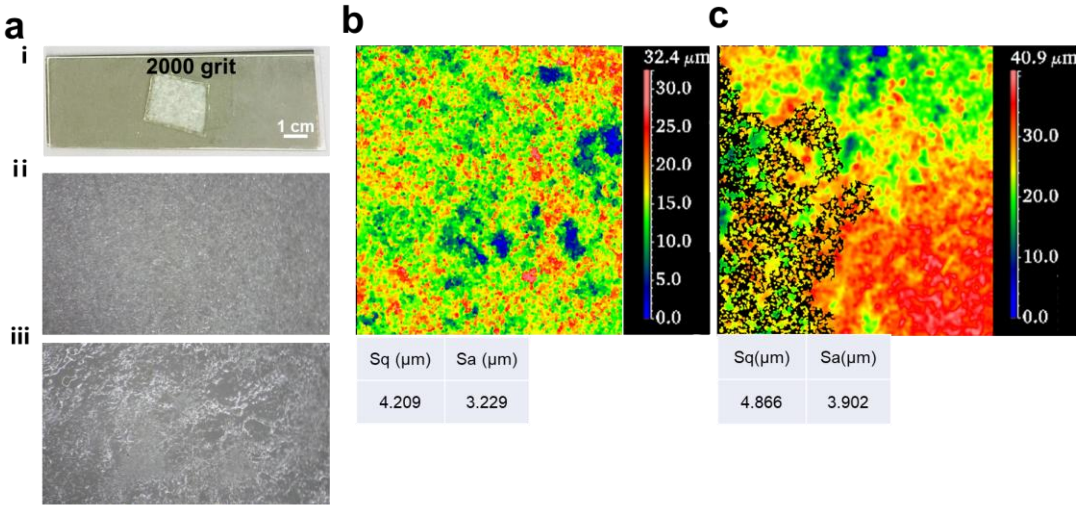

Supplementary Fig. 31. Conformal adhesion in crystalline eutectogels. (a) (i) Eutectogel adhering to coarse-grit sandpaper M2C; (ii) Microstructure of the sandpaper surface; and (iii) Microstructure of the gel surface. (b) Surface topography of the 2000 grit sandpaper. (c) Surface topography of the eutectogel adhered to 2000-grit sandpaper after the M2C process (sample information: $EG_{0.67-30}$).

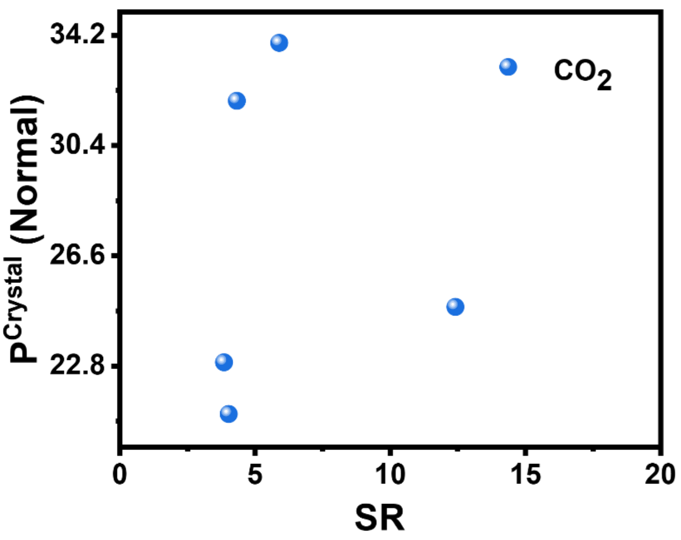

Supplementary Fig. 32. Adhesion selectivity of eutectogels in the crystalline state. Its irregularity indicates that eutectogels in the crystalline state do not follow the adhesion selectivity theory proposed by Linghu et al.[1] for shape memory polymers (sample information: $EG_{0.67\text{-}30}$).

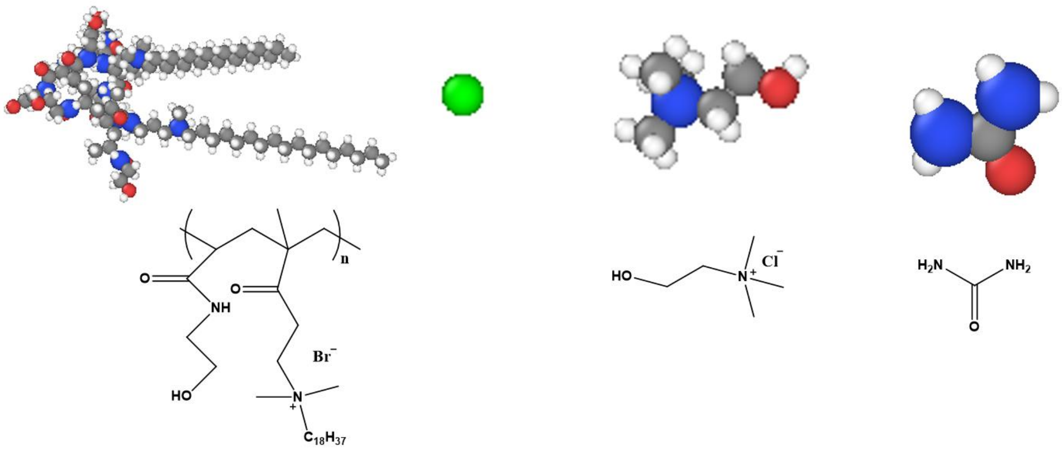

Supplementary Fig. 33. Molecular structures used in the molecular dynamics simulation process: polymer chains, choline chloride, and urea (sample information: $EG_{0.67-30}$).

Supplementary Note 3

Molecular Dynamics:

All simulations were carried out using the Large-scale Atomic/Molecular Massively Parallel Simulator (LAMMPS)[2], and the models were constructed to compute the debonding force of crystal and M2C states. A simulation box with dimensions of 128 Å × 40 Å × 541 Å was created under periodic boundary conditions. All of the models are built using CHARMM-GUI[3] and the SiO_2 is fixed during the simulation. The number ratio of the polymers, choline chloride and urea molecules is set as 305:22:8 to build the eutectogel networks, which is selected based on the same mass ratio in experiments. The eutectogel networks were first heated to 800 K and relaxed at 500 K to soften, and then cooled to 300 K for annealing, allowing them to solidify and adhere to the SiO_2 surface. The difference between the Crystal and M2C models lies in the annealing process: during the annealing of M2C, an additional pressure was applied to the eutectogel networks toward the SiO_2 surface, resulting in a larger interfacial adhesion area. After model construction, we use a spring, whose spring constant is 2000 kcal/(mol·Å^2), to pull up the eutectogel networks with 5e-3 Å/fs to a debonded state under NVT conditions at 300 K for crystal and M2C. For the melt state, the eutectogel networks were pulled under NVT conditions at 360 K. As shown in **Fig. S34**, the adhesion areas between the SiO_2 and eutectogel networks are increasing progressively in three states. The debonding forces are calculated by the internal forces of the spring. The OVITO[4] was employed for visualization.

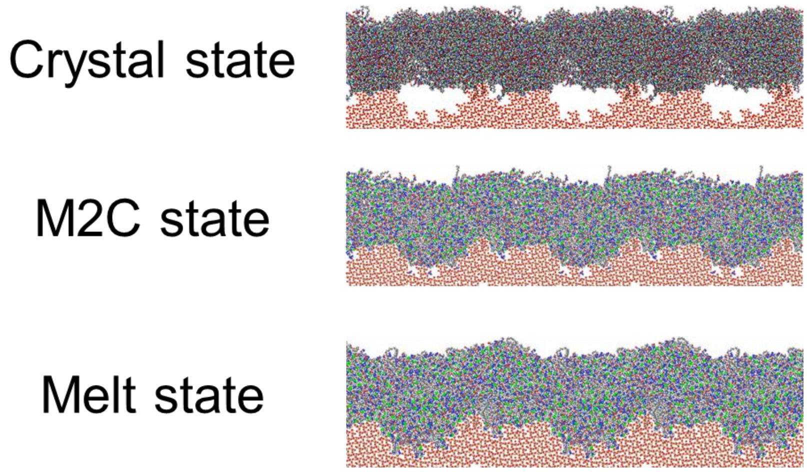

Supplementary Fig. 34. Schematic diagram of adhesion processes in crystalline state, M2C state, and molten state as simulated by molecular dynamics.

In the eutectogel network, the shortest relaxation-time peaks correspond to the P(HEAOMA) polymer, while the two broader peaks at longer relaxation times are attributed to the DES components (**Supplementary Fig. 35**). Comparison between crystalline and amorphous eutectogels shows that the transverse relaxation time (T_2) is consistently shorter in the crystalline state for both the polymer and solvent peaks, indicating restricted molecular motion due to solvent crystallization. Two-dimensional low-field NMR spectra provided T_1–T_2 probability density distributions of the system. In the amorphous gel, the T_1/T_2 ratio indicates that PHEAA polymer chains exhibit very low mobility, while the DES solvent remains relatively mobile with a clear liquid-like signature (**Supplementary Fig. 36**). The spin population morphology for PHEAA appears spindle-shaped and orthogonal to the diagonal, consistent with restricted chain motion, whereas the DES solvent shows an elongated spindle parallel to the diagonal, indicative of free movement in an open environment. In contrast, in the crystalline eutectogel, both polymer and solvent populations have T_1/T_2 ratios exceeding 10, signifying low mobility and solid-like behavior[56]. The PHEAA morphology remains similar, but the DES solvent population becomes a short spindle misaligned with the diagonal, suggesting severe restriction of solvent motion—strong evidence of system crystallization (**Supplementary Fig. 37-S38**). Complementary 2D-SAXS measurements corroborated these structural changes (**Supplementary Fig. 39**). Furthermore, temperature-dependent low-field NMR revealed that, upon heating, the peaks corresponding to both polymer and solvent components increased in intensity, with the solvent peaks showing the most pronounced growth. This indicates thermal dissociation of the solvent from its crystalline phase at elevated temperatures (**Supplementary Fig. 40**).

In situ variable-temperature infrared spectroscopy is used to track hydrogen-bond evolution during heating. The broad band at 3200-3600 cm^{-1}, assigned to νs (–OH) and νs (–NH_2), reports on hydrogen-bonding states in the eutectic system. In amorphous gels, νs (–NH_2) bands undergo pronounced red shifts (3306 → 3320 cm^{-1}; 3190 → 3196 cm^{-1}) upon heating (**Supplementary Fig. 41a-c**), indicating disruption of urea–choline chloride hydrogen bonds. A concurrent red shift of the νs (C=O) band (~1660 cm^{-1}) confirms dissociation of urea–DES hydrogen bonds. In contrast, crystalline gels display slight blue shifts in νs (–NH_2) (3196 → 3190 cm^{-1}; 3310 → 3106 cm^{-1}) during heating (**Supplementary Fig. 41h-j**), suggesting strengthening of urea–choline chloride hydrogen bonds. This is consistent with partial dissolution of eutectic crystals releasing free urea and choline chloride, which then form additional intermolecular hydrogen bonds. However, the νs (C=O) band still exhibits a red shift, likely arising from disruption of urea–urea hydrogen bonds. These trends indicate that the crystalline–molten phase transition is driven by competition between intra- and intermolecular hydrogen bonding in the urea–choline chloride network.

Two-dimensional correlation spectroscopy (2D-COS) further resolves the temperature response hierarchy of functional groups. In amorphous gels, the sequence derived from NODA analysis is: νs ($(CH_3)_3N^+$) (1450–1600 cm^{-1}, choline chloride) → νs (C=O) (1660 cm^{-1}, urea) → νs (–OH) (3417 cm^{-1}, choline chloride) → νs (–NH_2) (3307 cm^{-1}, urea), indicating that quaternary ammonium groups are most thermally labile

(**Supplementary Fig. 41d-g**). In crystalline gels, the order shifts to: νs (–OH) → νs (–NH_2) → νs (C=O) → νs ($(CH_3)_3N^+$), suggesting that hydroxyl groups in the crystalline matrix respond first to heating, initiating crystal breakdown into a liquid eutectic solvent(**Supplementary Fig. 41k-n**). In both systems, solvent-associated groups respond prior to polymer backbone signals, underscoring that temperature modulation of urea–choline chloride hydrogen bonding governs dynamic phase separation in eutectogels. Due to the intrinsic properties of DES, the eutectogel is hygroscopic and sensitive to humidity levels exceeding 80%. However, controlled drying of the eutectogels in an oven effectively mitigates performance degradation caused by moisture uptake. After comprehensive evaluation of the performance of eutectogels at different ratios, the eutectogel with a urea-choline chloride molar ratio of 1:1.5 and a monomer solid content of 30 wt% was selected as the primary subject for subsequent studies.

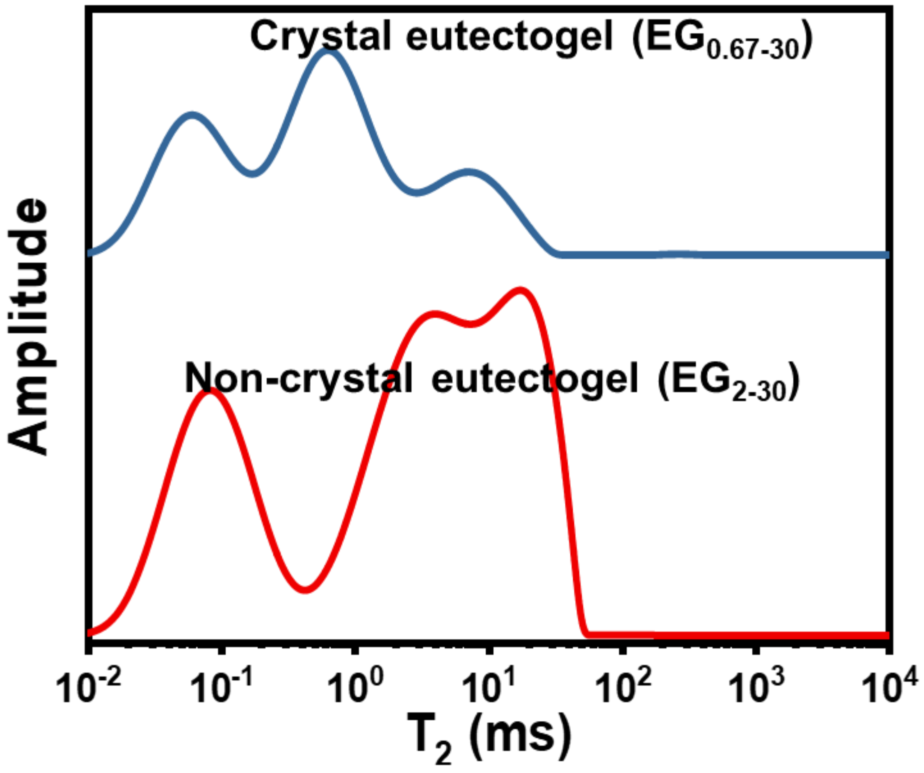

Supplementary Fig. 35. One-dimensional low-field NMR mapping of crystalline and amorphous eutectogels.

Supplementary Note 4

Two primary forms of low-field NMR relaxation are recognized: longitudinal relaxation (T_1) and transverse relaxation (T_2)[5, 6]. The T_1 time, referred to as the spin–lattice relaxation time, corresponds to the recovery of the magnetic moment toward thermal equilibrium. In contrast, T_2, known as the spin–spin relaxation time, characterizes the loss of phase coherence among individual magnetic moments in the transverse plane. It is frequently feasible to acquire T_1 and T_2 relaxation times almost simultaneously through correlated relaxation measurements. Two-dimensional low-field NMR maps yield a joint probability density distribution representing combinations of T_1–T_2 values.

T_2 relaxation times reflect the mobility of ^1H nuclei, with longer times indicating higher mobility. The T_1/T_2 ratio serves as an indicator of molecular constraint; a higher ratio corresponds to reduced mobility. This approach allows the decoupling of ^1H signals that exhibit similar mobility but differ in migration rates, thereby revealing heterogeneity within the system. From the perspective of migration dynamics, a T_1/T_2 ratio of 10 can be regarded as a threshold distinguishing solid-like from liquid-like behavior (**Supplementary Fig. 37**).

Furthermore, the morphology of spin populations in the 2D relaxation maps conveys structural and dynamic information. A circular distribution suggests that the components are in equilibrium within a confined environment. A spindle-shaped distribution indicates exchange processes occurring in an open system. When this spindle is aligned parallel to the diagonal, it reflects unrestricted exchange within a fully open space. Conversely, an orthogonal spindle-shaped distribution suggests confinement within a partially restricted space, where molecular motion is hindered by the local environment (**Supplementary Fig. 38**)[7].

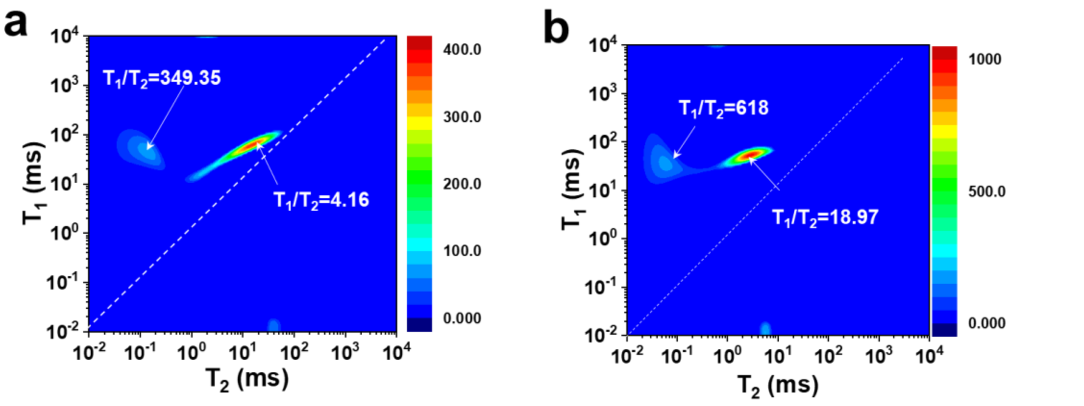

Supplementary Fig. 36. Molecular characterization of crystalline and amorphous eutectogels. (a) Two-dimensional low-field NMR spectra of amorphous eutectogels. (b) Two-dimensional low-field NMR spectra of crystalline eutectogels. The results show that the motion of DES is restricted in the crystalline state compared with the amorphous state (sample information: $EG_{0.67\text{-}30}$ and $EG_{2\text{-}30}$).

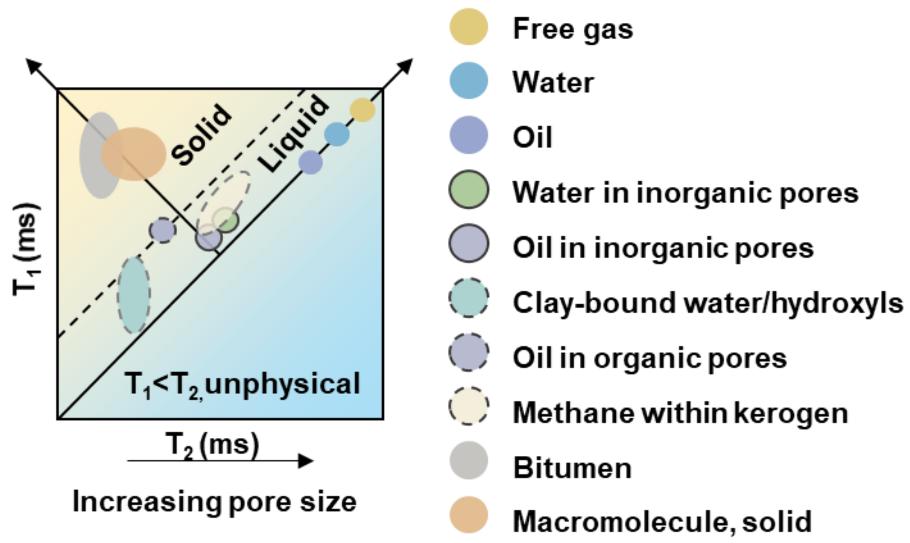

Supplementary Fig. 37. NMR-T_1-T_2 relaxation map for different fluid types and conditions adapted from. The short $T_{1,2}$ times indicate a small pore structure assigned to bound water. The long $T_{1,2}$ times indicates a large pore structure assigned to free water (increasing activity of ^1H). The large T_1/T_2 ratio indicates a large molecular weight or high-regularity lattice structure assigned to solid matter (decreasing mobility of ^1H).

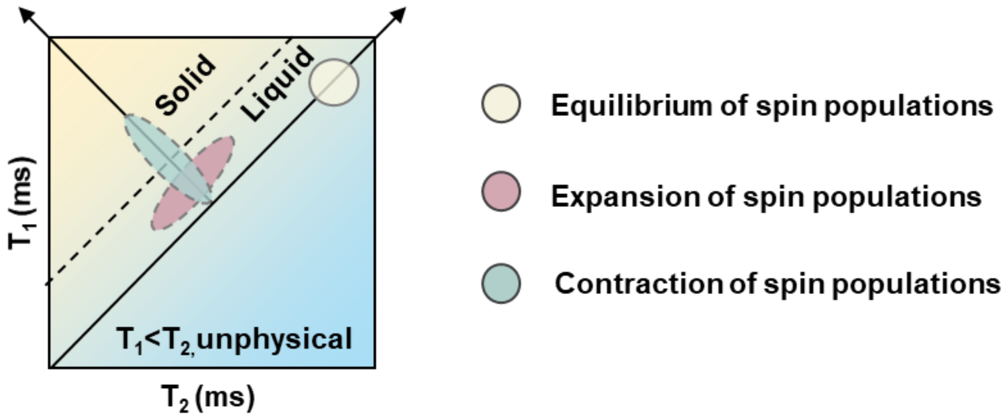

Supplementary Fig. 38. NMR-T_1-T_2 relaxation map for different fluid states. The circular pattern indicates stable entropy, where ^{1}H remains confined and does not exchange with the external environment. The spindle pattern aligned parallel to the diagonal represents an open space where ^{1}H exchanges with the external environment, resulting in increasing entropy. The spindle pattern perpendicular to the diagonal indicates a semi-closed space where ^{1}H is constrained by the external environment, leading the decreasing entropy.

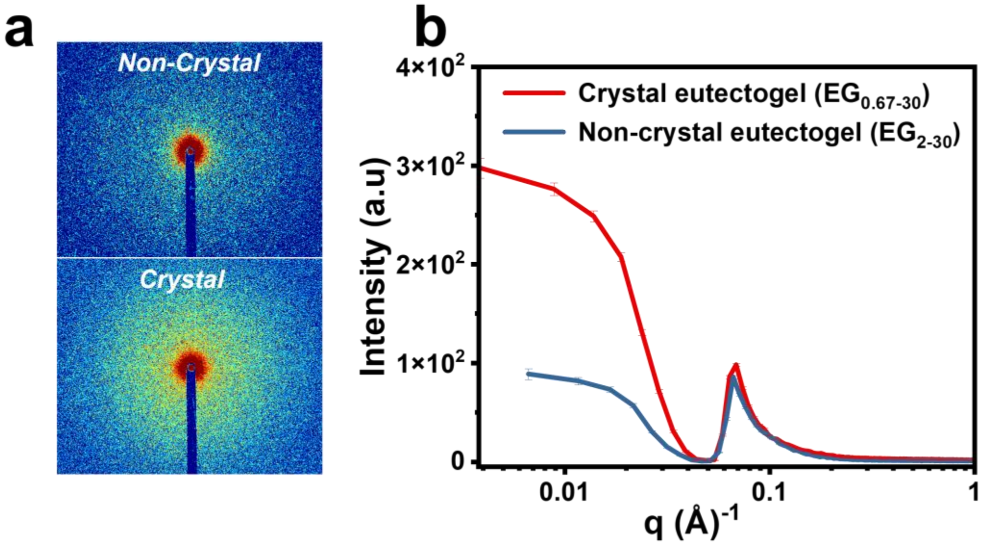

Supplementary Fig. 39. Comparison of small-angle X-ray scattering characterization of crystalline and amorphous eutectogels. (a) 2D-SAXS images of crystalline and amorphous eutectogels. (b) SAXS scattering intensity diagrams of crystalline $EG_{0.67\text{-}30}$ and amorphous $EG_{2\text{-}30}$ eutectogels.

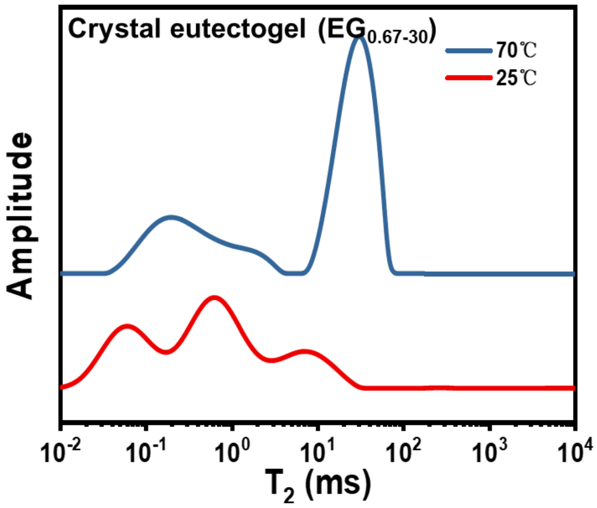

Supplementary Fig. 40. Low-field NMR patterns of crystalline eutectogels at high and low temperatures, indicating thermal dissociation of DES at high temperature (70°C).

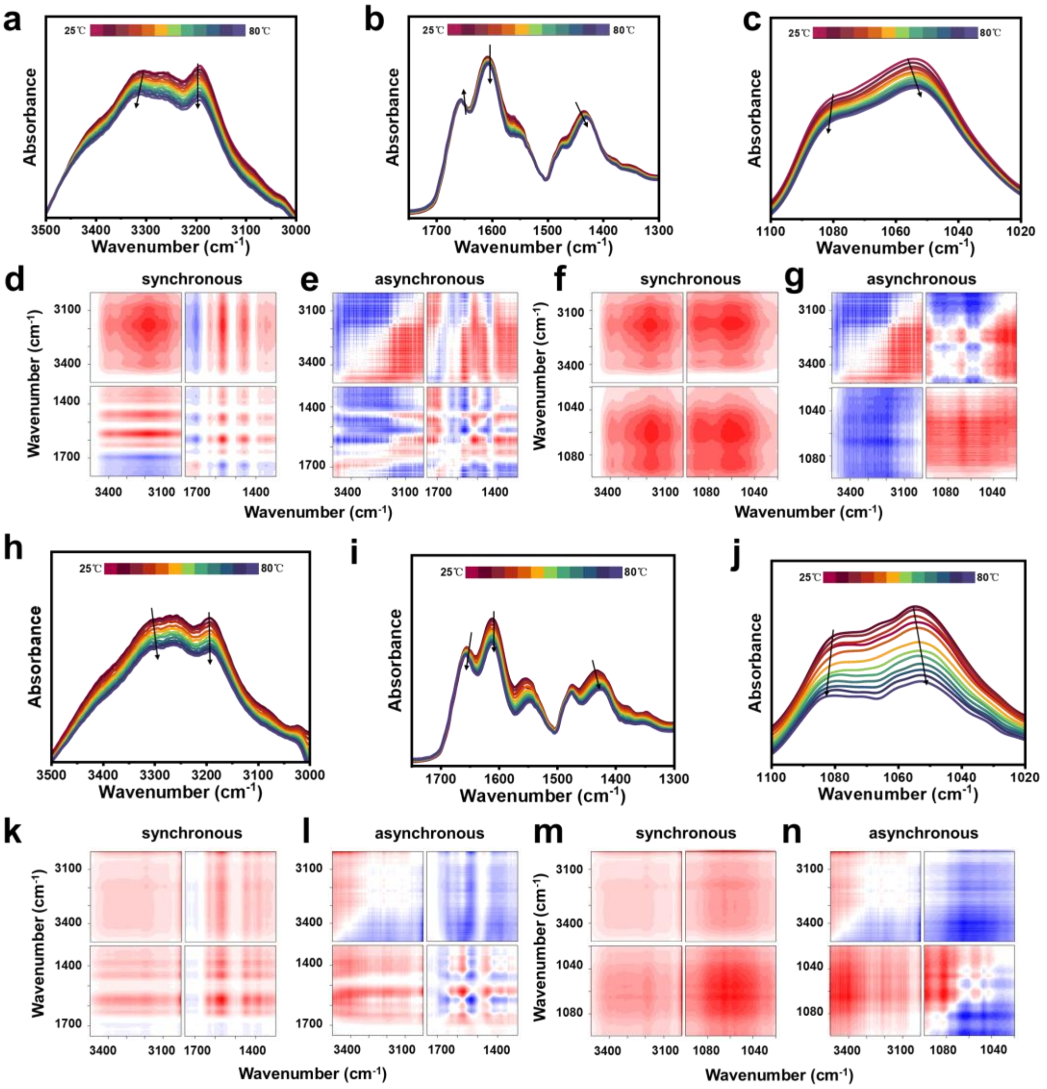

Supplementary Fig. 41. ATR-FTIR spectra of eutectogels. (a-c) In situ variable temperature FTIR spectra of amorphous eutectogel EG$_{2\text{-}30}$ in the temperature range 25°C-80°C. (d-g) Two-dimensional synchronous and asynchronous spectra of amorphous eutectogel EG$_{2\text{-}30}$ during warming from 25°C to 80°C. (h-j) In situ variable temperature FTIR spectra of crystalline eutectogel EG$_{0.67\text{-}30}$ in the temperature range 25°C-80°C. (k-n) Two-dimensional synchronous and asynchronous spectra of crystalline eutectogel EG$_{0.67\text{-}30}$ during warming from 25 °C to 80 °C.

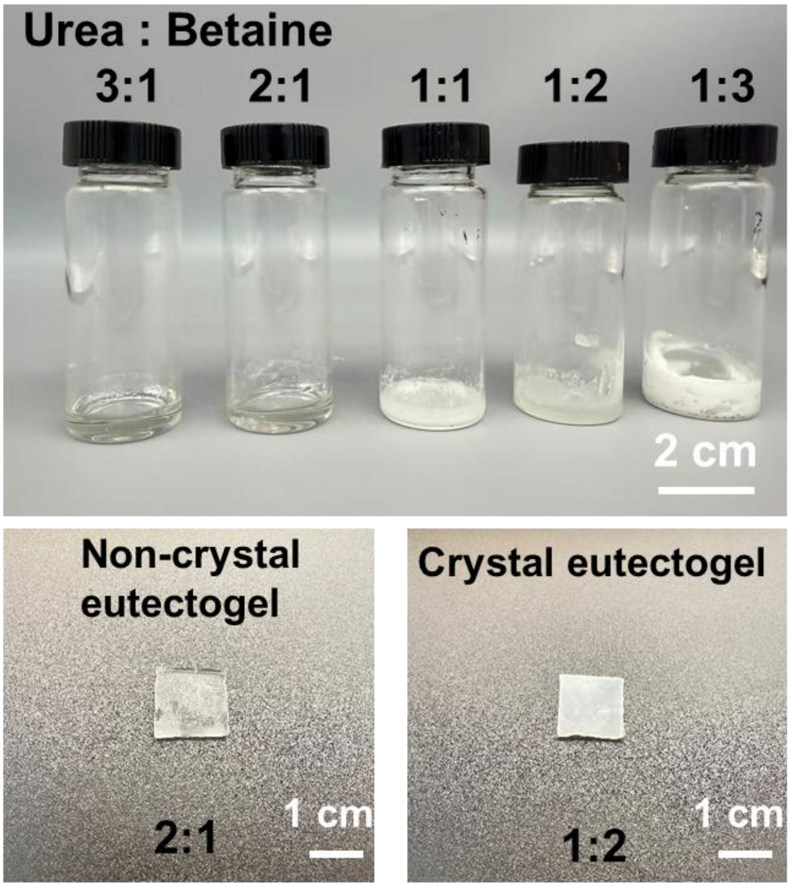

Supplementary Fig. 42. Crystallization behavior of eutectic solvents prepared with urea and betaine as hydrogen bond donors and acceptors, respectively, and comparison with corresponding eutectogels (sample information: Non-crystal eutectogel $EG_{2\text{-}30}$ and crystal eutectogel $EG_{0.5\text{-}30}$).

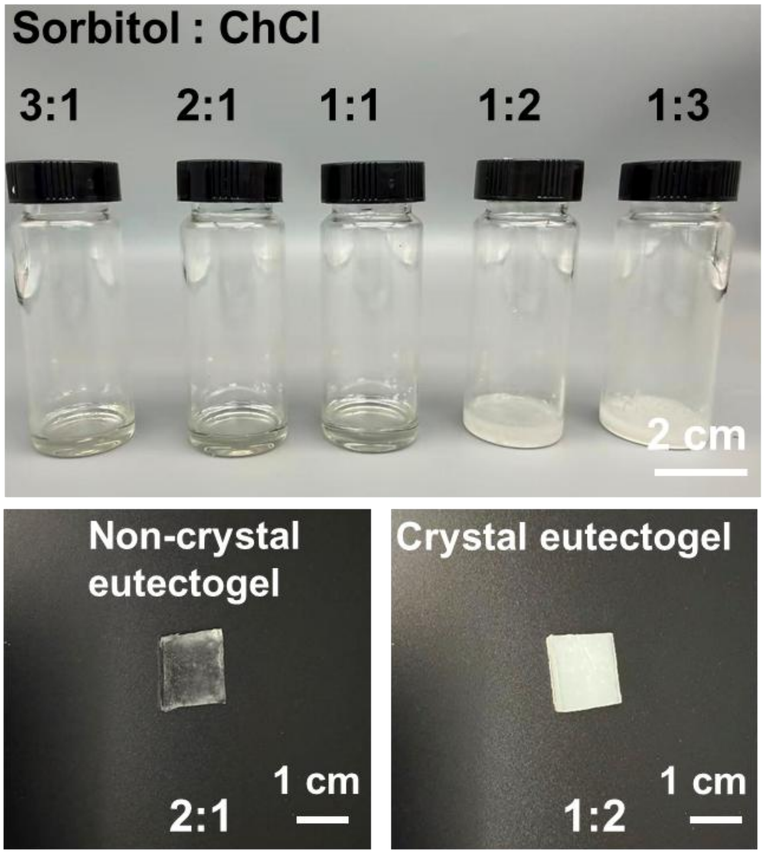

Supplementary Fig. 43. Crystallization behavior of eutectic solvents prepared with sorbitol and choline chloride as hydrogen bond donors and acceptors, respectively, and comparison with corresponding eutectogels (sample information: Non-crystal eutectogel $EG_{2\text{-}30}$ and crystal eutectogel $EG_{0.5\text{-}30}$).

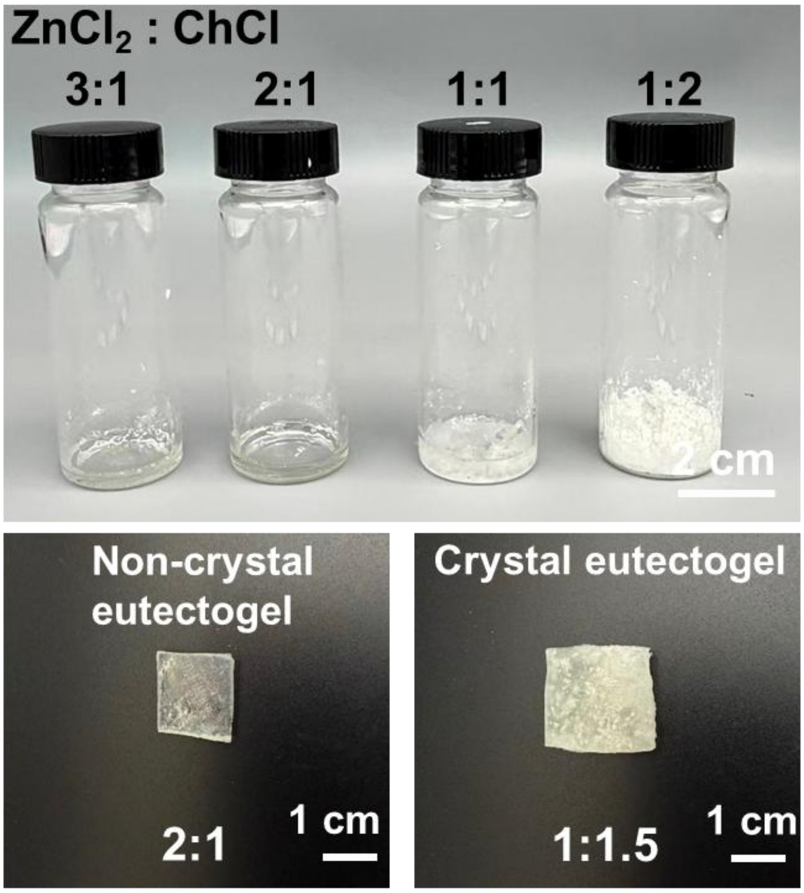

Supplementary Fig. 44. Crystallization behavior of eutectic solvents prepared with $ZnCl_2$ and choline chloride as hydrogen bond donors and acceptors, respectively, and comparison with corresponding eutectogels (sample information: Non-crystal eutectogel $EG_{2\text{-}30}$ and crystal eutectogel $EG_{0.5\text{-}30}$).

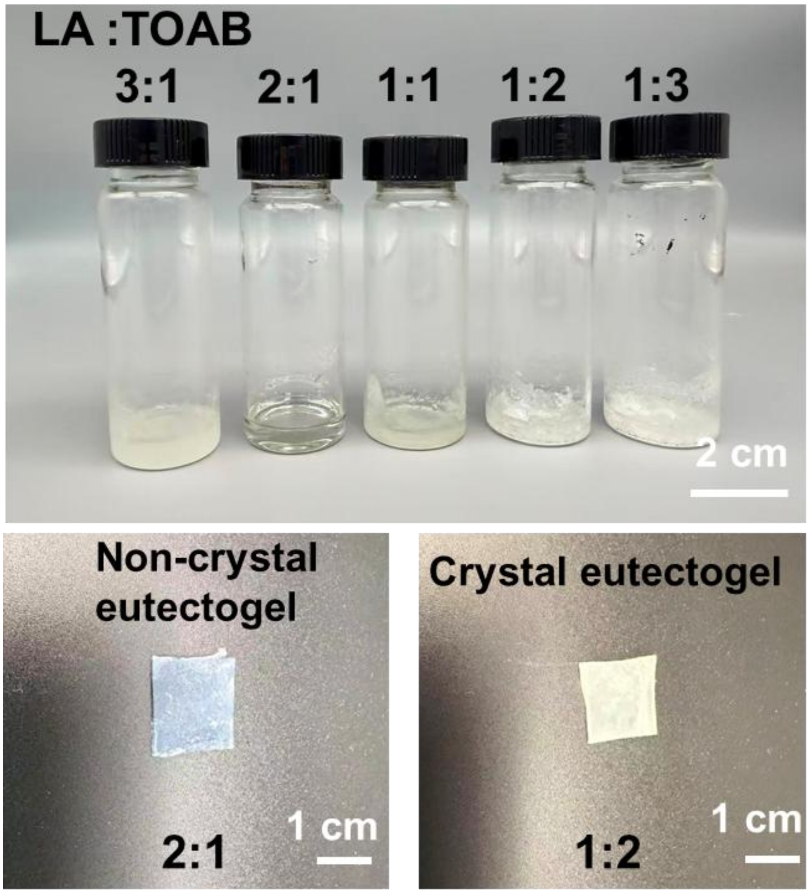

Supplementary Fig. 45. Crystallization behavior of eutectic solvents prepared with lauric acid and Tetra-octylammonium bromide as hydrogen bond donors and acceptors, respectively, and comparison with corresponding eutectogels (sample information: Non-crystal eutectogel $EG_{2\text{-}30}$ and crystal eutectogel $EG_{0.5\text{-}30}$, hydrophobic monomer: DMA).

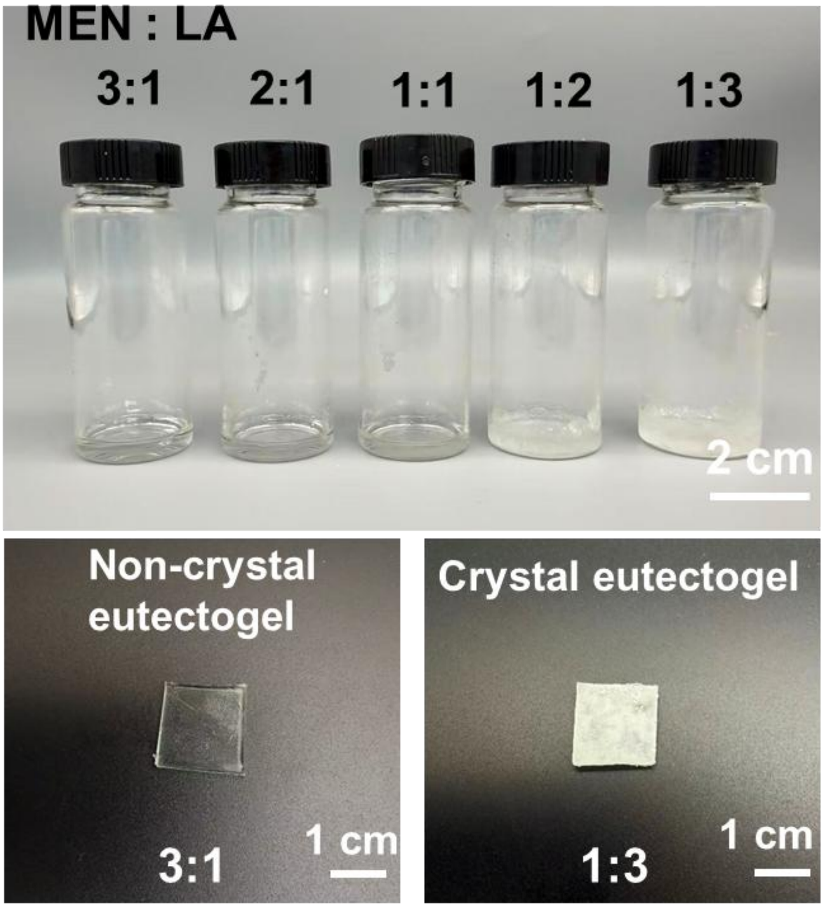

Supplementary Fig. 46. Crystallization behavior of eutectic solvents prepared with menthol and lauric acid as hydrogen bond donors and acceptors, respectively, and comparison with corresponding eutectogels (sample information: Non-crystal eutectogel $EG_{3\text{-}30}$ and crystal eutectogel $EG_{0.33\text{-}30}$, hydrophobic monomer: DMA).

Supplementary Note 5

Owing to the challenges associated with direct observation of interfacial phenomena, adhesion strength remains difficult to predict quantitatively. It is governed by both the density of interaction sites and the energy of individual bonds. For instance, hydrogen bonds typically operate at lengths between 0.2 and 0.4 nm, indicating that interfacial adhesion is fundamentally a nanoscale process. Similarly, in the context of supercapacitors, the capacitance of the electric double layer (EDL)—formed at the interface between ionic and electronic conductors—depends on multiple factors such as ion concentration and species. Based on the classical EDL model (**Supplementary Fig. 47**), the total EDL capacitance can be approximated as the series combination of the Helmholtz layer capacitance (C_H) and the diffuse layer capacitance (C_D). The simplified expression for the EDL capacitance is given by:

$$C_{EDL} = \left(\frac{1}{C_H} + \frac{1}{C_D}\right)^{-1} = \eta_A \bullet \varphi(d, \varepsilon, C, \phi, T) \bullet A \qquad (2)$$

A, is the contact area of the ionic conductor and electronic conductor. CH and CD are considered to be in proportion to the contact area (A). The value of EDL capacitance is affected by many factors, including η_A (roughness ratio between the actual and ideally smooth surface), ε (dielectric constant of the ionic conductor), d (the thickness of Helmholtz layer), C (ionic species and concentrations), ϕ (the surface potential) and T (temperature). Shklovskii et al. estimated that the apparent width of the Helmholtz layer is as small as 0.3 Å[8]. Brown et al. measured the size of the EDL under solvent conditions[9]. They found that the width of the Helmholtz layer in NaCl solvent is ≈0.8 nm. Although the width of the Helmholtz layer is dependent on other factors, such as the ion size and solvent environment, it can still be considered that the interaction of interface adhesion and the effect of the EDL are on the same scale.

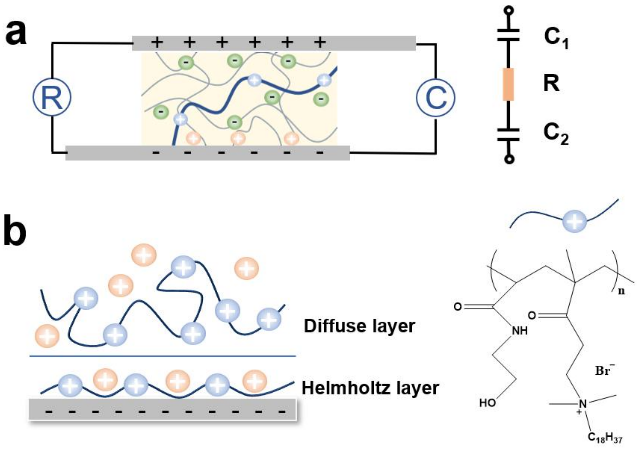

Supplementary Fig. 47. (a) Schematic diagram of supercapacitors and equivalent circuits based on eutectogels and metal electrodes. (b) Schematic diagram of the electrochemical double layer and the internal structure of eutectogel.

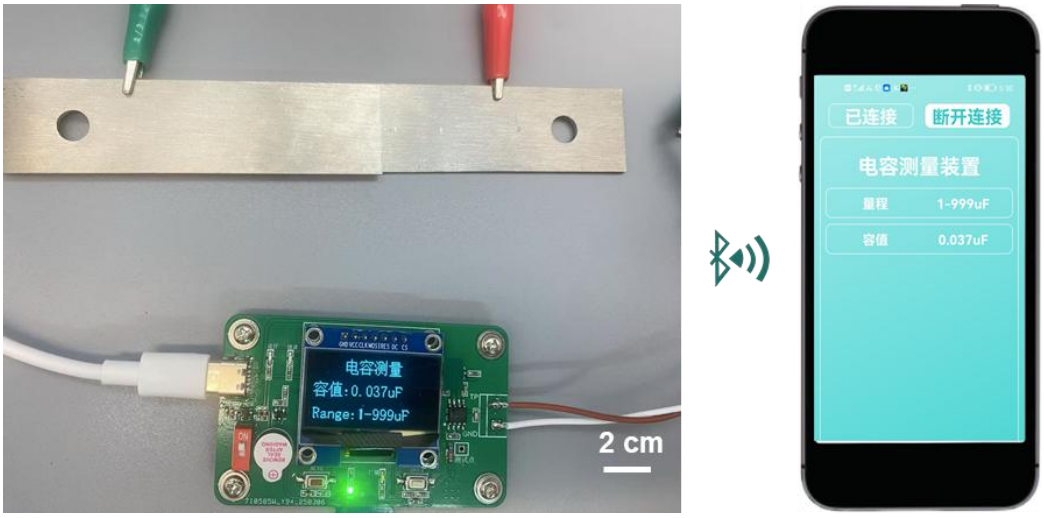

Supplementary Fig. 48. A Bluetooth-enabled capacitance monitoring module, based on the ST32 chip, was developed to monitor the capacitance of the supercapacitor fabricated with metal electrodes and eutectogel. The capacitance values are transmitted in real time via Bluetooth to a smartphone terminal.

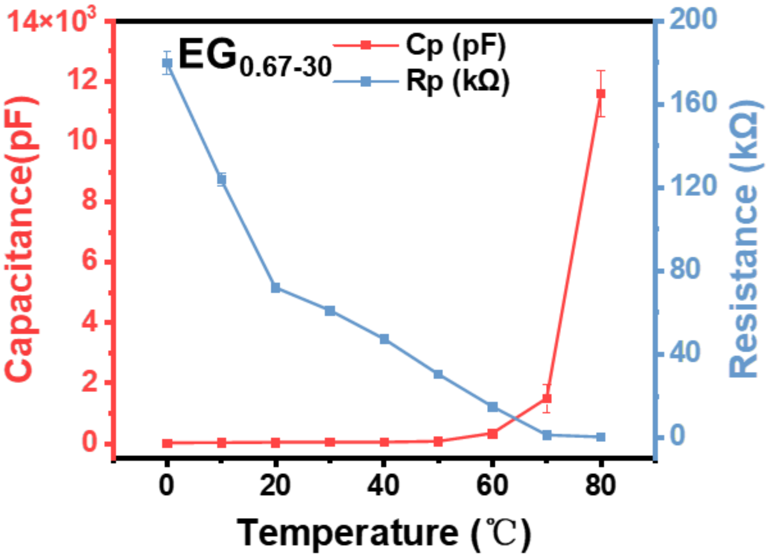

Supplementary Fig. 49. The electrical resistance Rp and capacitance Cp values of eutectogel adhesives at different temperatures. Cp gradually increases while Rp decreases with increasing temperature.

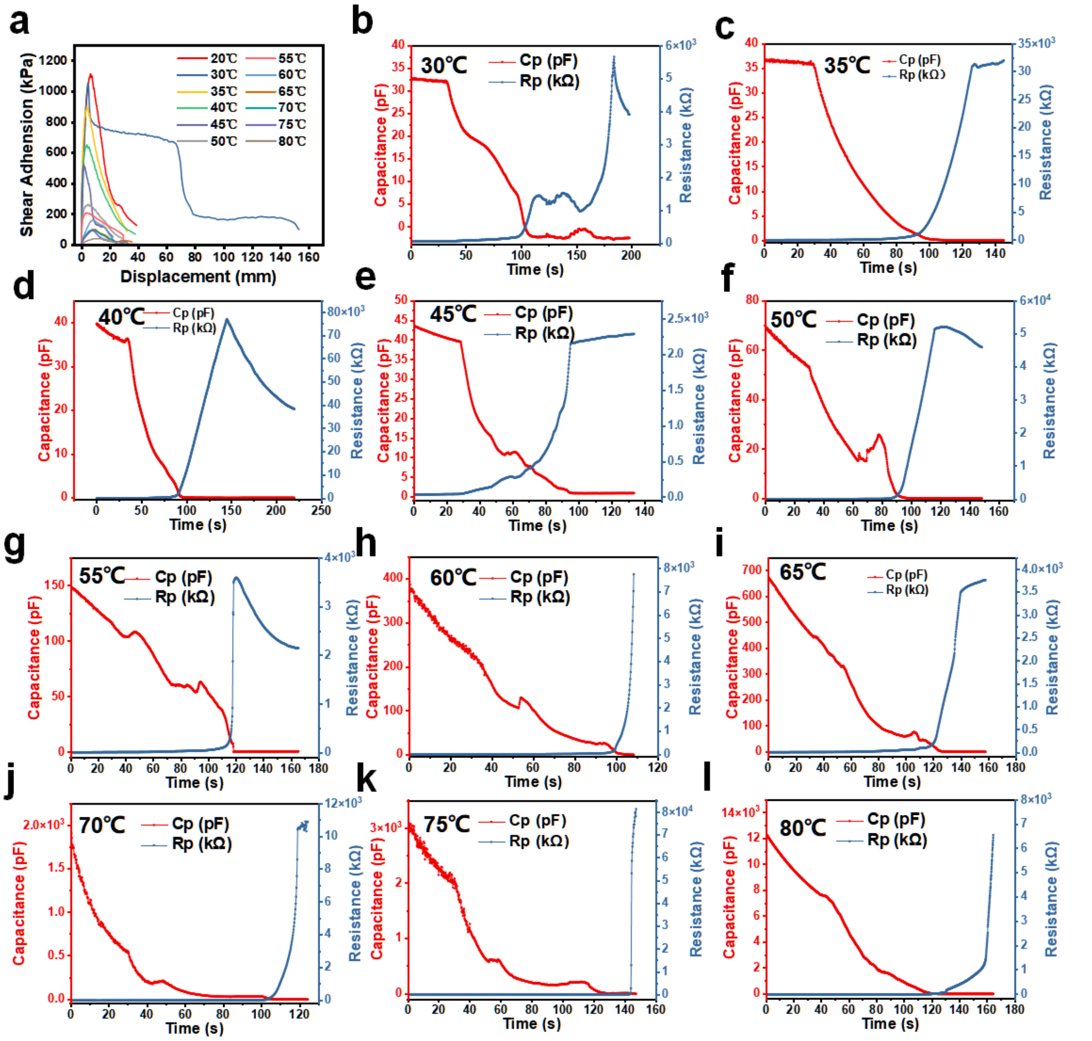

Supplementary Fig. 50. Changes in electrical signals during adhesion-release testing under different temperature conditions. (a) The adhesion performance of eutectogel is influenced by temperature. (b-l) Monitoring of electrical signals during the adhesion failure process of eutectogel under external force when temperature serves as an environmental factor (sample information: $EG_{0.67\text{-}30}$).

Supplementary Note 6

The failure prediction model is a custom fully-connected Multi-Layer Perceptron (MLP) developed for binary failure classification, with the input dimension corresponding to the number of input features $\mathrm{n_{vars}}$. An optional L_2-normalization is applied to the input feature tensor along the last dimension for feature standardization before subsequent processing. The backbone network comprises three successive linear layers: the first linear layer projects the input features from $\mathrm{n_{vars}}$ to $4\mathrm{n_{vars}}$, the second linear layer expands the feature dimension to $8\mathrm{n_{vars}}$, and the third linear layer contracts the dimension back to $4\mathrm{n_{vars}}$. Each linear layer is followed by a Layer Normalization and a Sigmoid Linear Unit (SiLU) activation function, which enables effective non-linear feature learning and stable network training. A single linear classification head maps the high-dimensional feature representations to a scalar value, and a sigmoid activation function constrains the final output to the interval [0,1], representing the predicted failure probability of the sample.

F1-score is selected as the evaluation metric for the binary classification task, which is the harmonic mean of precision and recall with a value range of [0,1]. Recall is critical for failure prediction, as it measures the model's ability to identify all actual failure cases and avoid missing potential failures. For binary classification, three core metrics from the confusion matrix are defined as follows: TP (True Positive), FP (False Positive) and FN (False Negative).

Precision, recall and F1-score are expressed as:

$$Precision = \frac{TP}{TP + FP} \tag{3}$$

$$Recall = \frac{TP}{TP + FN} \tag{4}$$

$$F1 = \frac{2TP}{2TP + FP + FN} \tag{5}$$

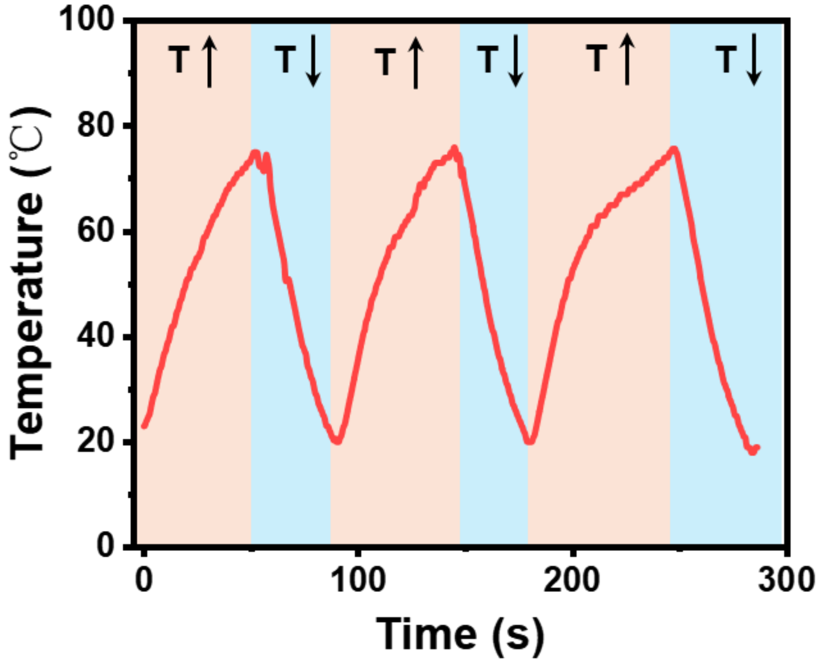

Supplementary Fig. 51. Temperature curve of eutectogel during heating and cooling in the operation of the eutectogel smart gripper.

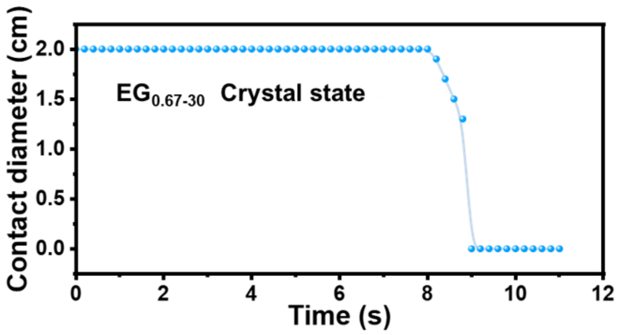

Supplementary Fig. 52. The behavior of a eutectogel ($EG_{0.67\text{-}30}$) smart gripper when grasping an object (100 g) in the crystalline state.

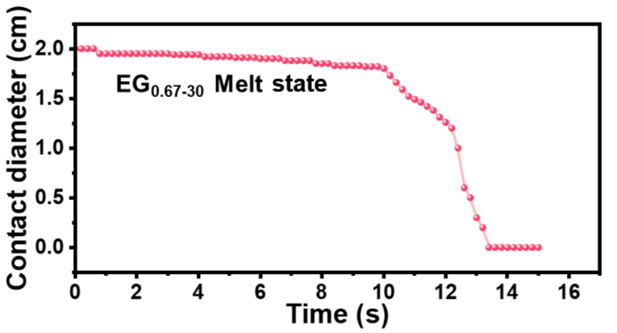

Supplementary Fig. 53. The behavior of a eutectogel ($EG_{0.67\text{-}30}$) smart gripper when grasping an object (250 g) in the melting state.

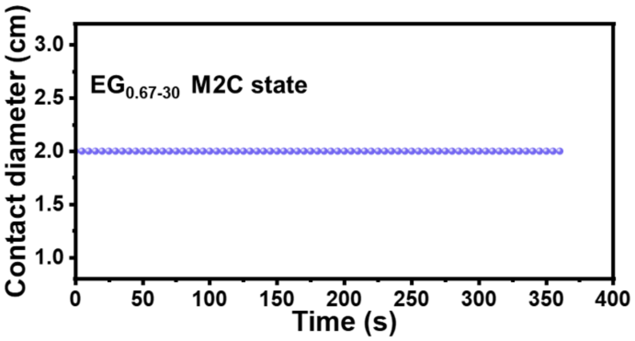

Supplementary Fig. 54. The behavior of a eutectogel ($EG_{0.67\text{-}30}$) smart gripper when grasping an object (500 g) in the M2C state.

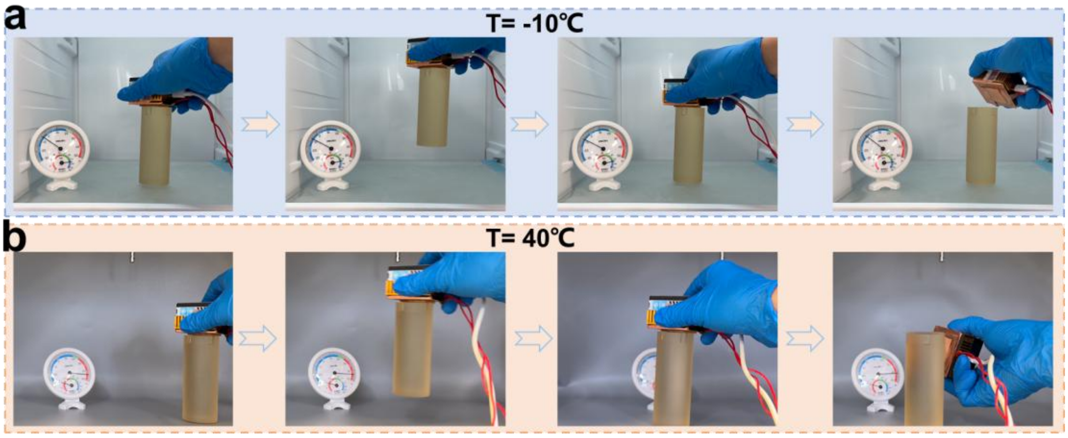

Supplementary Fig. 55. The grasping and releasing process of an intelligent gripper using eutectogel under different temperature conditions. (a) Eutectogel ($EG_{0.67-30}$) intelligent gripper grabs digital pictures of target objects at low temperature (-10°C). (b) Eutectogel ($EG_{0.67-30}$) intelligent gripper grabs digital pictures of target objects at high temperature (40°C).

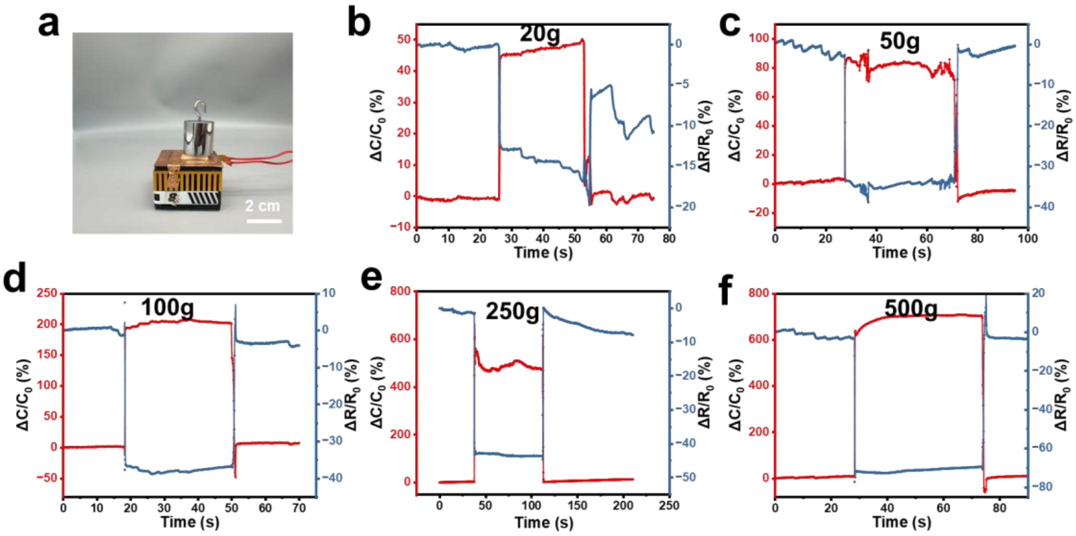

Supplementary Fig. 56. The eutectogel smart gripper detects the magnitude of pressure exerted by different weights through electrical signals. (a) Digital images of weights placed on the surface of the gripper. (b-f) Electrical signal variation curve generated when 20 g-500 g weights are placed on the eutectogel ($EG_{0.67\text{-}30}$) surface of the gripper.

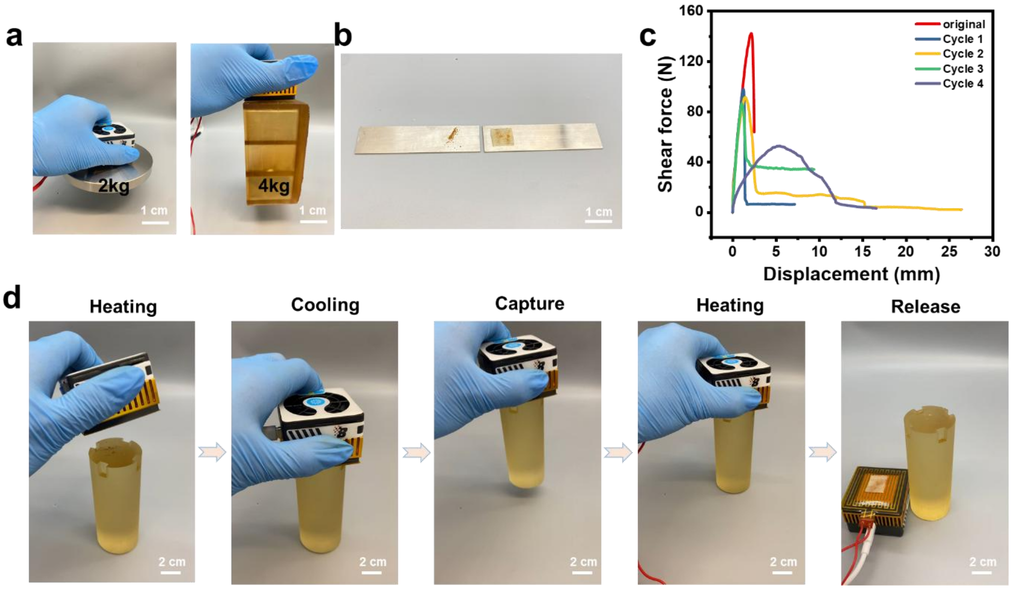

Supplementary Fig. 57. Gripping performance of the eutectogel-based intelligent gripper. (a) Target objects lifted via a grasper utilizing the M2C adhesion mechanism, including a 2 kg metal disk and a 4 kg resin frame embedded with metal components. (b) Adhesion performance of the eutectogel on a dust-contaminated metal substrate. (c) Cyclic shear adhesion test of the eutectogel on a dusty metal surface. The material retains effective adhesion after four repeated attachment–detachment cycles. (d) Repeated grasping and release of objects from dusty surfaces using eutectogel-based M2C adhesion.

Supplementary Note 7

Foot A houses the gear-reduction drive motors and control module, whereas the lateral feet provide auxiliary adhesion. Each foot comprises four stacked layers: (1) a polylactic acid (PLA) structural support layer, (2) a silicone insulation layer, (3) a stainless-steel metal layer, and (4) an electrothermal eutectogel layer. The PLA layer provides mechanical stability, the silicone layer insulates against heat damage, and the stainless-steel layer functions both as a heat dissipation interface and an adhesion substrate. These layers are assembled using screws and adhesive.

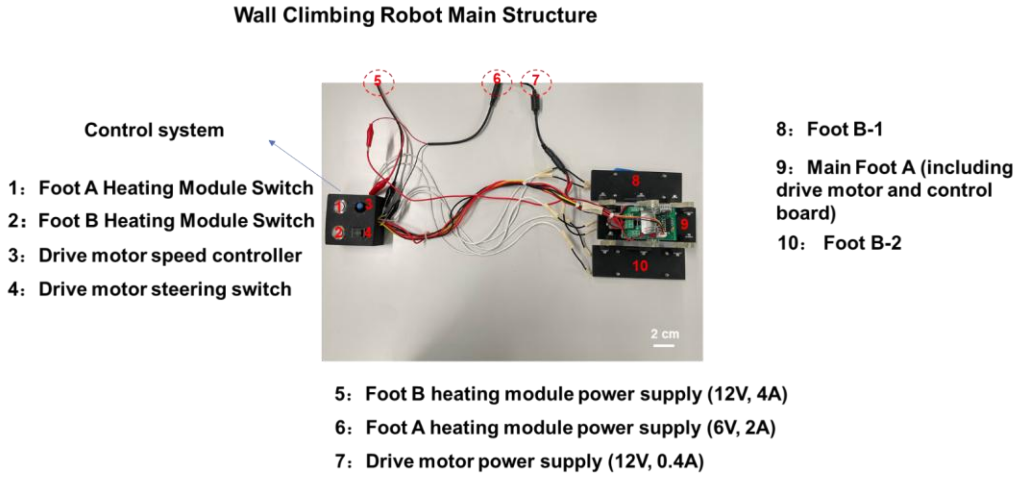

Supplementary Fig. 58. Structure and control components of the wall-climbing robot.

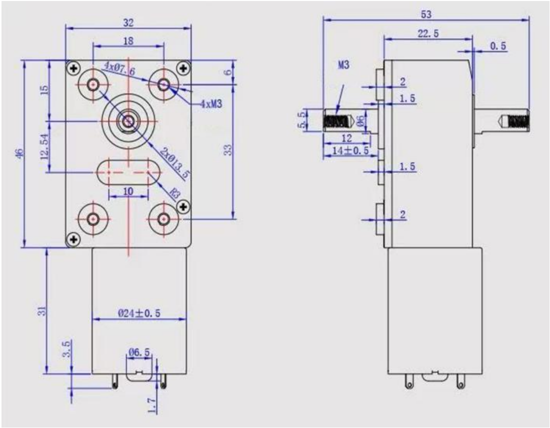

Supplementary Fig. 59. Design of two-axis geared drive motors of the wall-climbing robot.

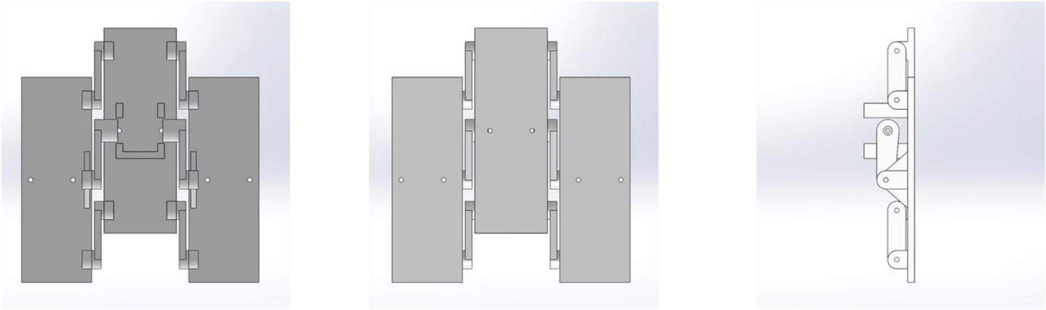

Supplementary Fig. 60. 3D view of the 3D printed mechanical structure of the wall-climbing robot.

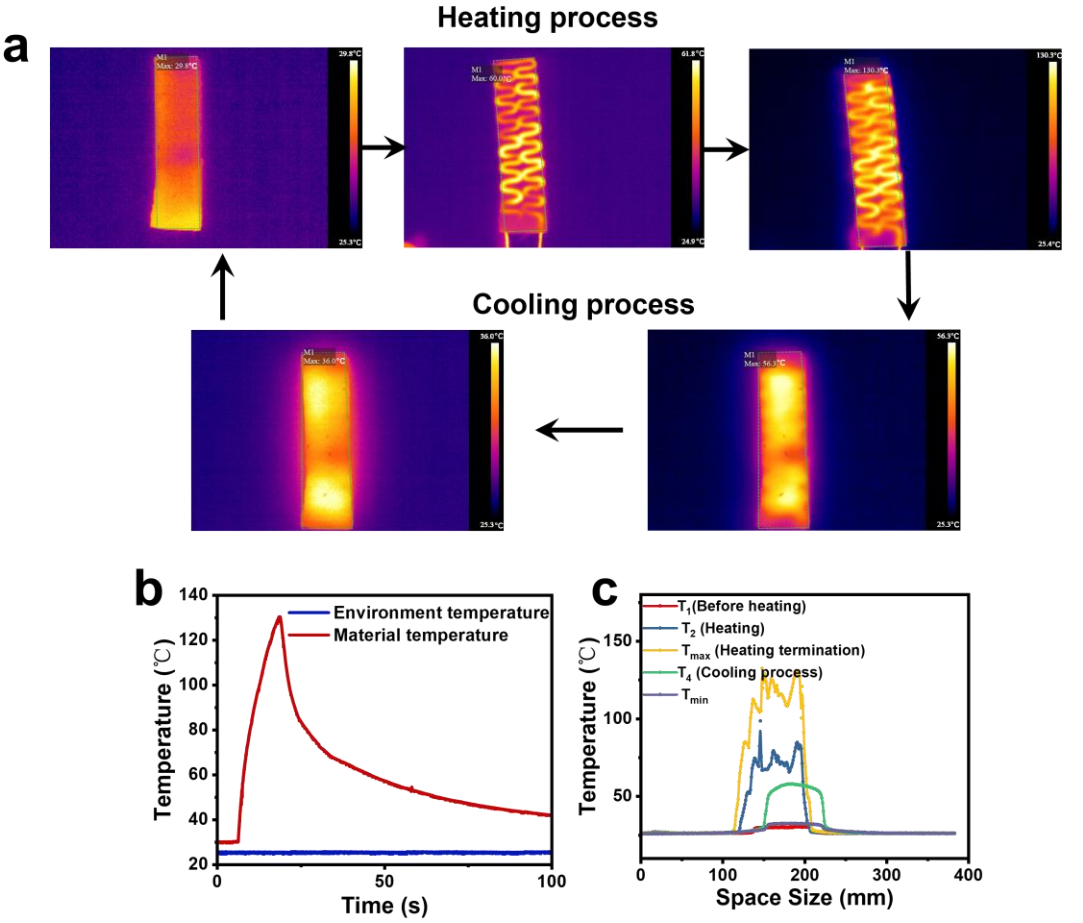

Supplementary Fig. 61. Temperature behavior of eutectogel heated by nickel-chromium alloy heating wires (6V, 2A). (a) Infrared thermography images of the melting and cooling crystallisation process of eutectogels heated by a resistance wire. (b) Temperature profiles for melting and cooling of eutectogels heated by a resistance

wire. (c) Spatial temperature profiles for melting and cooling of eutectogels heated by a resistance wire.

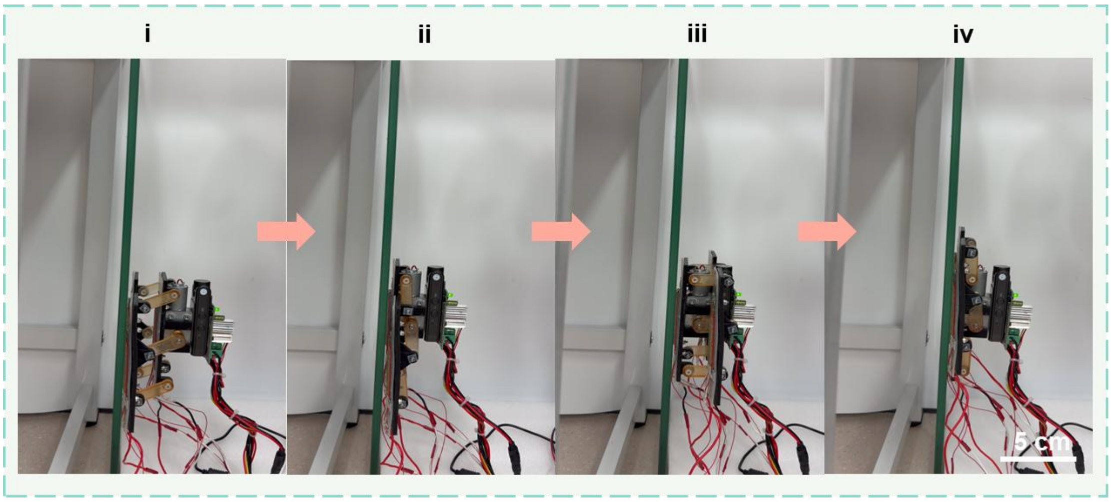

Supplementary Fig. 62. Images of a wall-climbing robot climbing on a 90° vertical wall.

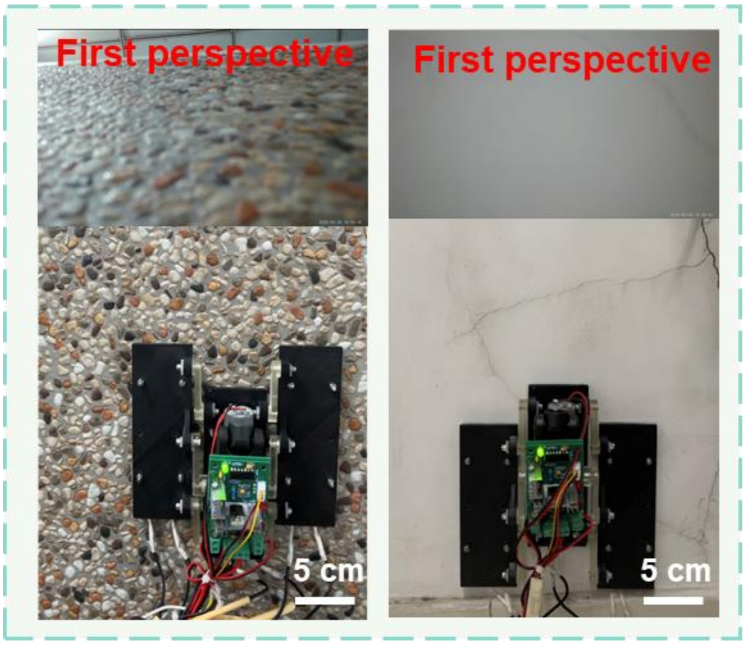

Supplementary Fig. 63. The robot is climbing on a rough stone wall and a lime wall. The camera equipped on the climbing robot can transmit first-view images for wall inspection.

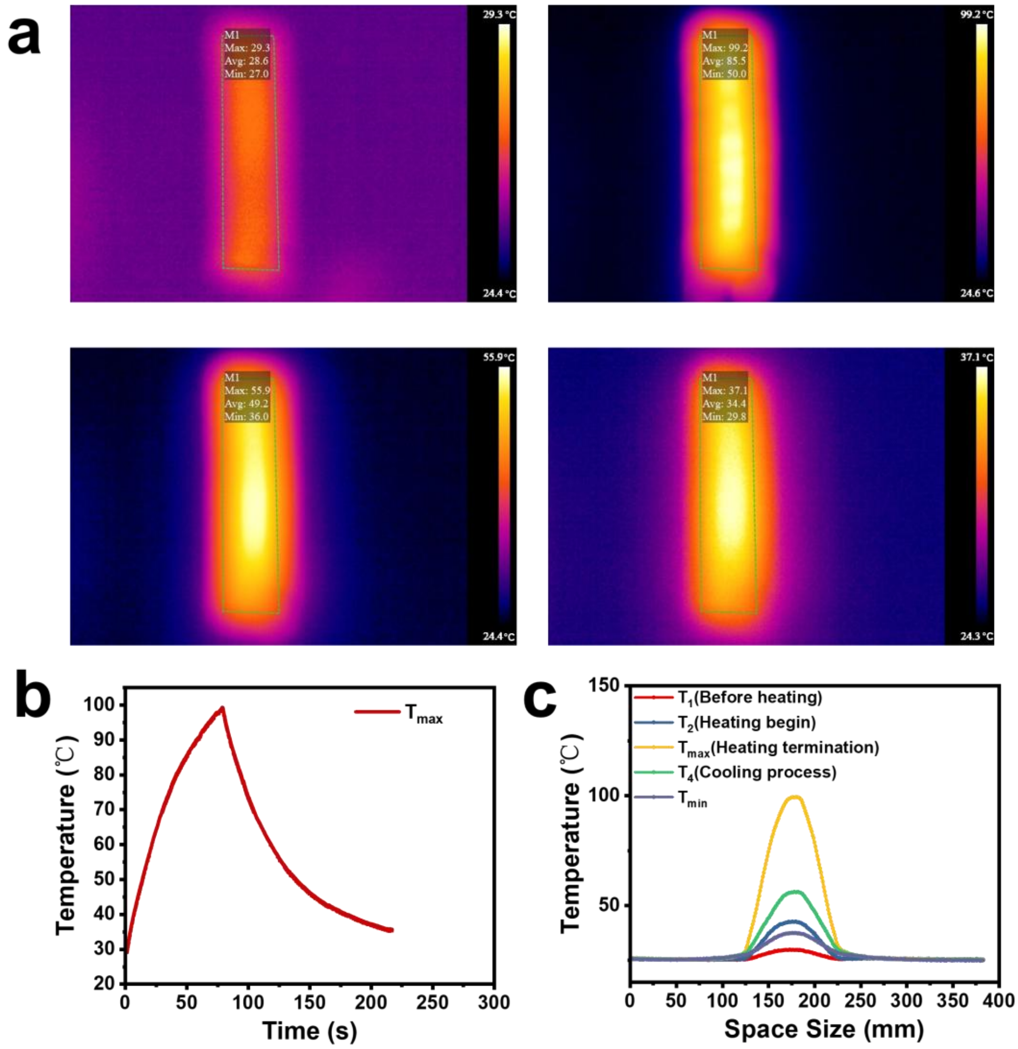

Supplementary Fig. 64. Temperature behavior of eutectic gel heated by nickel-chromium alloy heating wires (3V, 2A). (a) Infrared thermogram of eutectogel heated by resistance wire at 3V heating voltage and 2A current. (b) Temperature profiles for melting and cooling of eutectogels heated by a resistance wire. (c) Spatial temperature profiles for melting and cooling of eutectogels heated by a resistance wire.

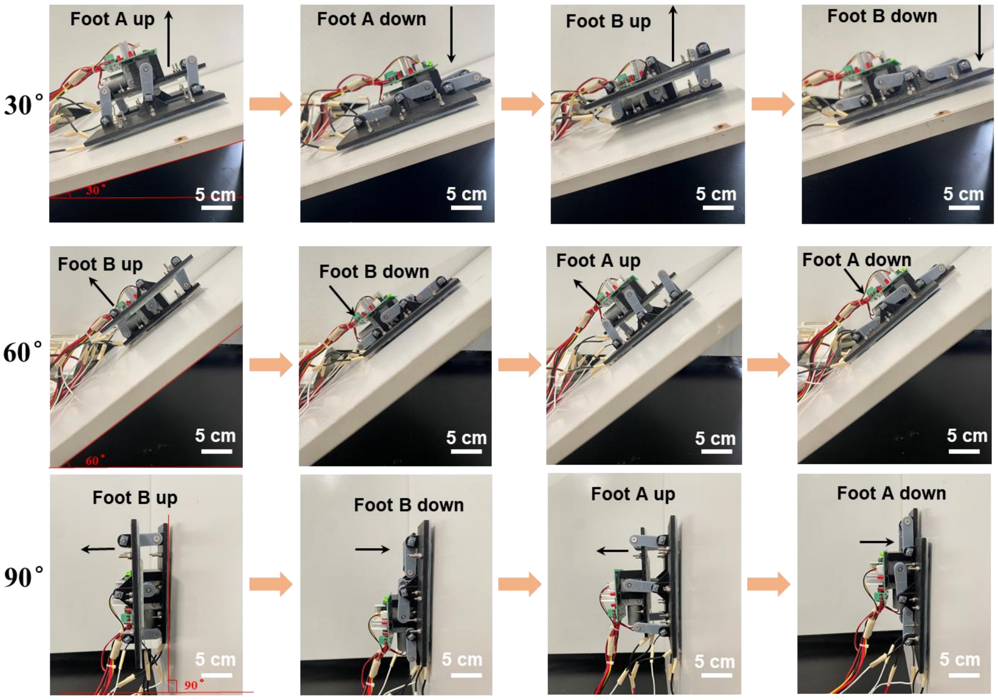

Supplementary Fig. 65. The crawling process of a three-legged wall-climbing robot on the 30°, 60°, and 90° planes, respectively.

Supplementary References

1. Linghu C*, et al.* Overcoming the adhesion paradox and switchability conflict on rough surfaces with shape-memory polymers. *Proceedings of the National Academy of Sciences* **120**, e2221049120 (2023).

2. Thompson AP*, et al.* LAMMPS - a flexible simulation tool for particle-based materials modeling at the atomic, meso, and continuum scales. *Computer Physics Communications* **271**, 108171 (2022).

3. Jo S, Kim T, Iyer VG, Im W. CHARMM-GUI: A web-based graphical user interface for CHARMM. *Journal of Computational Chemistry* **29**, 1859-1865 (2008).

4. Stukowski A. Visualization and analysis of atomistic simulation data with OVITO–the Open Visualization Tool. *Modelling and Simulation in Materials Science and Engineering* **18**, 015012 (2010).

5. Baran E, Birczyński A, Dorożyński P, Kulinowski P. Low-field time-domain NMR relaxometry for studying polymer hydration and mobilization in sodium alginate matrix tablets. *Carbohydrate Polymers* **299**, 120215 (2023).

6. Wang S, Lin R, Cheng s, Tan M. Water dynamics changes and protein denaturation in surf clam evaluated by two-dimensional LF-NMR T1-T2 relaxation technique during heating process. *Food Chemistry* **320**, 126622 (2020).

7. Liu Y, Song Y, Wu P. Self-Evolving Hierarchical Hydrogel Fibers with Circadian Rhythms and Memory Functions. *Advanced Materials* **36**, 2404506 (2024).

8. Skinner B, Loth MS, Shklovskii BI. Capacitance of the Double Layer Formed at the Metal/Ionic-Conductor Interface: How Large Can It Be? *Physical Review Letters* **104**, 128302 (2010).

9. Brown MA, Goel A, Abbas Z. Effect of Electrolyte Concentration on the Stern Layer Thickness at a Charged Interface. *Angewandte Chemie International Edition* **55**, 3790-3794 (2016).
10. Amend, J. R., Brown, E., Rodenberg, N., Jaeger, H. M. & Lipson, H. A Positive Pressure Universal Gripper Based on the Jamming of Granular Material. IEEE Trans. Robot. 28, 341–350 (2012).

11. Hawkes, E. W., Christensen, D. L., Han, A. K., Jiang, H. & Cutkosky, M. R. Grasping without squeezing: Shear adhesion gripper with fibrillar thin film. in

2015 IEEE International Conference on Robotics and Automation (ICRA) 2305–2312 (2015).

12. Song, S., Drotlef, D.-M., Majidi, C. & Sitti, M. Controllable load sharing for soft adhesive interfaces on three-dimensional surfaces. Proc. Natl. Acad. Sci. 114, E4344–E4353 (2017).

13. Koivikko, A., Drotlef, D.-M., Sitti, M. & Sariola, V. Magnetically switchable soft suction grippers. Extreme Mech. Lett. 44, 101263 (2021).

14. Linghu, C. et al. Universal SMP gripper with massive and selective capabilities for multiscaled, arbitrarily shaped objects. Sci. Adv. 6, eaay5120 (2020).

15. An, S., Cao, Y. & Jiang, H. A mechanically robust and facile shape morphing using tensile-induced buckling. Sci. Adv. 10, eado8431 (2024).

16. Lyu, B. et al. Humanoid finger with rigid-flexible-soft structure. Nat. Commun. 16, 9905 (2025).

17. Gao, D. et al. A supramolecular gel-elastomer system for soft iontronic adhesives. Nat. Commun. 14, 1990 (2023).

18. Liu, W. et al. Curved loop strip soft gripper. Int. J. Mech. Sci. 305, 110746 (2025).

19. Wen, H. et al. Magnetoactive bistable soft actuators for programmable large shape transformations at low magnetic fields. Nat. Commun. 16, 9714 (2025).

20. Tang Y, Zhang Q, Lin G, Yin J. Switchable Adhesion Actuator for Amphibious Climbing Soft Robot. *Soft Robotics* **5**, 592-600 (2018).

21. Gu G, Zou J, Zhao R, Zhao X, Zhu X. Soft wall-climbing robots. *Science Robotics* **3**, eaat2874 (2018).

22. Huang J*, et al.* Electrically programmable adhesive hydrogels for climbing robots. *Science Robotics* **6**, eabe1858 (2021).

23. Hong S, Um Y, Park J, Park H-W. Agile and versatile climbing on ferromagnetic surfaces with a quadrupedal robot. *Science Robotics* **7**, eadd1017 (2022).

24. Wu Y, Dong X, Kim J-k, Wang C, Sitti M. Wireless soft millirobots for climbing three-dimensional surfaces in confined spaces. *Science Advances* **8**, eabn3431 (2022).

25. Qin K*, et al.* Parthenocissus-inspired soft climbing robots. *Science Advances* **11**, eadt9284 (2025).

26. Yue T, Bloomfield-Gadêlha H, Rossiter J. Snail-inspired water-enhanced soft sliding suction for climbing robots. *Nature Communications* **15**, 4038 (2024).

27. Pang W*, et al.* A soft microrobot with highly deformable 3D actuators for climbing and transitioning complex surfaces. *Proceedings of the National Academy of Sciences* **119**, e2215028119 (2022).

28. Min H*, et al.* Stiffness-tunable velvet worm–inspired soft adhesive robot. *Science Advances* **10**, eadp8260.

29. Zheng M, Wang D, Zhu D, Cao S, Wang X, Zhang M. PiezoClimber: Versatile and Self-Transitional Climbing Soft Robot with Bioinspired Highly Directional Footpads. *Advanced Functional Materials* **34**, 2308384 (2024).
