# Supplementary material for: Switchable adhesion of phase-transition eutectogels with integrated machine learning-enhanced intelligent adhesion sensing
Source: Nat Commun. 2026 Jun 11;17:7434. doi: 10.1038/s41467-026-74275-7 (PMC13408668; doi:10.1038/s41467-026-74275-7)
Supplement: Supplementary file 2 — Description of Additional Supplementary Files [file 41467_2026_74275_MOESM2_ESM.pdf]

Description of Additional Supplementary Files

Supplementary Movie 1.
Description:
Crystal growth of urea-choline chloride deep eutectic solvent at room temperature.

Supplementary Movie 2.
Description:
Crystalline eutectogel Internal eutectic solvent crystallises and grows at room temperature.

Supplementary Movie 3.
Description:
Intelligent gripper based on the adhesion process of eutectogel M2C for transporting target objects.

Supplementary Movie 4.
Description:
Long-term adhesion behavior of eutectogel-based smart grippers in crystalline, molten states, and during the M2C process.

Supplementary Movie 5.
Description:
The eutectogel smart gripper's object-grasping process at high temperatures (40°C) and low temperatures (-10°C).

Supplementary Movie 6.
Description:
The smart gripper based on eutectogel M2C adhesion grasps and transports objects of varying shapes, sizes, and materials.

Supplementary Movie 7.
Description:
Intelligent gripper based on eutectogel M2C adhesion process for transporting heavy objects (1 kg).

Supplementary Movie 8.
Description:
A climbing robot developed based on eutectogel climbing on a glass wall.

Supplementary Movie 9.
Description:
A climbing robot developed based on eutectogels crawling on surfaces with slopes of 30°, 60°, 90°.

Supplementary Movie 10.
Description:
A climbing robot developed based on eutectogel climbing on a metal wall.

Supplementary Movie 11.

Description:
A climbing robot developed based on eutectogel climbing on a wooden wall.

Supplementary Movie 12.

Description:
A climbing robot developed based on eutectogel climbing on a lime wall.

Supplementary Movie 13.

Description:
A climbing robot developed based on eutectogel climbing on a tile wall.

Supplementary Movie 14.

Description:
The robot equipped with a camera is climbing on a rough stone wall.

Supplementary Movie 15.

Description:
The robot equipped with a camera is climbing on a cracked lime wall for inspection.
