# Supplementary material for: Switchable adhesion of phase-transition eutectogels with integrated machine learning-enhanced intelligent adhesion sensing
Source: Nat Commun. 2026 Jun 11;17:7434. doi: 10.1038/s41467-026-74275-7 (PMC13408668; doi:10.1038/s41467-026-74275-7)
Supplement: Supplementary file 18 — Transparent Peer Review File [file 41467_2026_74275_MOESM18_ESM.pdf]

nature portfolio

Peer Review File

Switchable adhesion of phase-transition eutectogels with integrated machine learning-enhanced intelligent adhesion sensing

Corresponding Author: Professor Wei Feng

This file contains all reviewer reports in order by version, followed by all author rebuttals in order by version.

Version 0:

Reviewer comments:

Reviewer #1

(Remarks to the Author)
crystallization-melting mechanism with a real-time adhesion monitoring capability and its wall-climbing robot. While the work includes extensive characterization and impressive demonstrations, I regret to recommend rejection of the manuscript in its current form for publication in Nature Communications. This recommendation is based on several fundamental concerns regarding the study's insufficient novelty, and significant issues with data presentation and scientific rigor that undermine the credibility of the findings.

The manuscript combines several concepts (materials, mechanism, applications) that are individually well-established in the field. Consequently, it fails to demonstrate a significant breakthrough or a substantial advance over the state-of-the-art.
1. The use of a Urea/Choline Chloride DES is the most common and extensively studied combination, as established by Abbott et al. Chem. Commun. 1, 70 (2003) and utilized in numerous subsequent eutectogel studies (e.g., Zhao et al. J. Mater. Chem. A. 10, 4222 (2022); Yang et al. ACS Appl. Mater. Interfaces 15, 55244 (2023)). More importantly, the strategy of using a crystallization-melting mechanism to switch adhesion has already been reported. The work by Chen et al. ACS Nano, 18, 23968, (2024) demonstrated this with PEG crystallization, and the work by Xu et al. Adv. Sci. 11, 2400938 (2024) is particularly concerning as it not only uses an utectogel for switchable adhesion but also demonstrates the exact same application of a wall-climbing robot. Furthermore, the core mechanism (controlling adhesion via a temperature-induced stiffness change) is conceptually analogous to the well-known principles of shape memory polymers (glass-to-rubber transition) and phase-separating hydrogels. The authors do not provide any quantitative or qualitative comparison to differentiate their mechanism or performance from these highly relevant prior works (e.g., Fang et al. Adv. Funct. Mater. 33, 2301505 (2023)-hydrogel; Linghu et al. Sci. Adv. 6, aay5120 (2020)-shape memory polymer; Annapooranan et al. PNAS, 121, e2412635121 (2024)-liquid crystal elastomer, making it impossible to assess the claimed advance.
2. The concept of an integrated adhesion monitoring system has been explored in prior works (e.g., Wang et al. Sci. Adv. 9, adf4051 (2023); Li et al. Chem. Eng. J. 523, 168354 (2025), which demonstrated real-time sensing during pick-and-place operations. Critically, the authors fail to integrate this sensing capability into their primary application (the climbing robot), which operates on non-conductive surfaces where the EDL-based sensing mechanism is not viable. The three demonstrations (pick-and-place, sensing, robot) are presented as isolated showcases rather than an integrated, synergistic system. Had the authors demonstrated a robot capable of sensing its own adhesion status, the impact would have been far greater. As it stands, this feature feels disconnected and its novelty is diluted.
3. The manuscript does not provide a compelling case for the performance superiority of this system. For instance, the climbing speed of the robot (~8 cm/min) is not benchmarked against other state-of-the-art climbing robots to demonstrate an advantage. The extensive molecular-level analyses (MD, NMR, SAXS, FTIR), while well-executed, primarily serve to confirm expected physical phenomena (i.e., that crystallization restricts molecular motion) rather than providing fundamentally new scientific insights that lead to a performance breakthrough.
4. The manuscript's structure is difficult to follow. A clear, logical thread connecting material optimization to final application performance is absent (which eutectogel composition exhibited the most pronounced switchable adhesion capability, as determined through experimental and analytical investigations). The manuscript would benefit from a significant reduction in the number of main-text figures, focusing only on the most critical data and moving supporting results to the SI.
5. A major flaw is the consistent omission of crucial experimental parameters. The exact sample compositions (U:C ratio, DES:monomer ratio) used for the key adhesion tests (Fig. 3b-f, h), molecular-level analyses (Fig. 4, 5), and application

demonstrations are not specified. Without this information, the results are neither credible nor reproducible. For example, Fig. 2d-i lack complete compositional information, making the data difficult to interpret.
6. Page 4: The sentence "Except for the case of U:C = 2:1, the melting points... are below ambient temperature... such that the mixture remains solid..." is a direct logical contradiction.
7. Fig. 2f and 2g: These plots appear identical despite being labeled as tensile and compression tests, respectively.
8. Fig. S16/S17: The blue curve is labeled "20°C Non-crystal eutectogel," which should likely be "20°C Crystal eutectogel." There are also inconsistencies between the figure legend (80°C) and the graph legend (70°C).
9. The presentation of adhesion data primarily as bar graphs is insufficient. Raw force-displacement curves or force-time curves for all adhesion tests are necessary for a rigorous assessment. The number of repeats and the nature of the error bars are not specified.
10. The claim on page 14 regarding "stronger gel cohesion" requires direct evidence from tensile tests (stress-strain curves) for the corresponding U:C ratios, which are not provided.
11. The absence of Dynamic Mechanical Analysis (DMA) is a notable omission for a study centered on temperature-dependent mechanical transitions.
12. The theoretical model (Eq. 1, 2) is presented without adequate explanation of its terms or the underlying physics (e.g., the roles of h_rms and shape locking), rendering it incomprehensible to non-specialist readers.

(Remarks on code availability)

Reviewer #2

(Remarks to the Author)
The authors have conducted a systematic work on an interesting reversible adhesive. By utilizing the controllable crystallization of the eutectogels, the authors achieved programmable attachment/detachment of adhesives on various substrates, particularly rough surfaces. Comparing the previous works on reversible adhesives, the adhesion strength of these eutectogels is much higher, while the surface adaptability is better. Real-time monitoring of adhesion strength is very important for exploring adhesive-enabled devices. Both spectroscopy analyses and theoretical calculations were conducted to get insights into the reversible adhesion mechanism. Also, the authors verified the potential applications of these smart adhesives in grasping and climbing robots. Overall, this paper is acceptable for publication in Nature Communications after addressing the following concerns.
(1) In the melted state, the eutectogel still has an adhesion strength of ~100 kPa. This is a relatively large value. Is it possible to further reduce the adhesion strength by alternating the composition of the polymer networks while maintaining the crystallization-melting phase transition?
(2) In Figure S16, the red line represents: 70 oC Crystal eutectogel, while the figure caption stated that “Tensile tests of crystalline eutectogels at high (80 °C) and room temperatures (20°C).”. Figure S17 has the same mistake.
(3) The size of the phase-change gel layer applied in the climbing robot should be clearly described in the experimental section.
(4) On page 3, it was claimed that “the adhesion strength can be tuned from 200 kPa to 1000 kPa”, while on page 5, it was claimed that “Specifically, the adhesion strength increases from ~100 kPa in the melted state to ~1 MPa in the melt-to-crystalline (M2C) state”. I suggest the authors give a consistent description.
(5) Please identify the samples used in Figure S11a and b
(6) In the caption of Figure 2, it was indicated that (f) Comparison of compression properties between crystalline and amorphous eutectogels, (g) Comparison of tensile properties between crystalline and amorphous eutectogels. However, Figures 2f and 2g are the same data. Please double-check the figures.
(7) Cyclic tensile tests indicate that the eutectogels undergo considerable mechanical strength reduction and permanent deformation during repeated stretching. This may not be beneficial for the practical application of the eutectogels in grasping and climbing robots. I suggest the authors give a detailed description of their strategies for addressing these problems.
(8) The authors are suggested to clearly list the control parameters for the grasping and robotic climbing experiments.
(9) Since the heating and cooling process of the adhesives can also be affected by the environmental temperatures, I suggest the authors clearly identify the temperature in grasping and robotic climbing experiments.

(Remarks on code availability)

Reviewer #3

(Remarks to the Author)
I co-reviewed this manuscript with one of the reviewers who provided the listed reports. This is part of the Nature Communications initiative to facilitate training in peer review and to provide appropriate recognition for Early Career Researchers who co-review manuscripts.

(Remarks on code availability)

Reviewer #4

(Remarks to the Author)

• Authors report an electrothermally switchable adhesion method for gripping and wall climbing. Electrothermally switchable adhesion has been shown before and the novelty the authors present here is their material selection. The material properties have been reported well in detail.
• Authors also claim the sensing ability their material have. It is not clear for me how the proposed material can be used as a conformable gripper if it sandwiched between metal plates? A photograph from the demonstration would be helpful.
• I understood that the sensing capability is only for sensing if something has been dropped. Would it be possible to use this sensing method for analyzing when the gripper is in contact with the object?
• Because of the soft and conformable gripping surface, I thought the authors would show gripping of different shaped objects (round, sharp, very soft, porous…) and small objects (smaller than gripper diameter) but I did not see this. Is that possible with the suggested gripping method? Also, how long can the gripper hold the heavy objects before dropping them? I.e. What is the long-term behavior of the gripper?
• The results are well reported and analyzed but I don't see the significance of these results to the field. If the main novelty is the sensing ability, only sensing the fracture is not impressive. Can different forces be measured with this gripper? How the environment affects to the sensing accuracy?
• Authors cite some relevant literature in their paper but more detailed comparison table would be useful. How large adhesion forces this gripper can produce compared to similar size different technologies? How much energy does this gripper need compared to other gripping technologies? Some papers to consider for the comparison:
1. S. Song, D. Drotlef, C. Majidi, & M. Sitti, Controllable load sharing for soft adhesive interfaces on three-dimensional surfaces, Proc. Natl. Acad. Sci. U.S.A. 114 (22) E4344-E4353, https://doi.org/10.1073/pnas.1620344114 (2017).
2. Anastasia Koivikko, Dirk-Michael Drotlef, Metin Sitti, Veikko Sariola, Magnetically switchable soft suction grippers, Extreme Mechanics Letters, Volume 44, 2021,101263, ISSN 2352-4316 https://doi.org/10.1016/j.eml.2021.101263.
• Authors claim that the material they propose is sustainable and environmentally friendly, but these claims are not supported in the paper.
• The paper does not have conclusion section and the discussion is really brief. It is not clear for me how these results compared to other reported gripping and climbing methods (Table was provided but it was more list like no comparison there) in the field and what is the novelty this research brings to the field.
• Methodology and analysis methods seem overall sound and well justified. The sensing experiment in Fig 7 however is not clear. b and c and simultaneous graphs but b dos not have time axis so it is difficult to see when the fracture happens exactly.
• The gripper is electromechanically switchable. How does the surrounding environment changes affect the gripper's performance?
Minor comments:
What is the x-axis in 3 b and d, same than c and e?
Fig. 4b what are work and forces 0 and 1? Same with 4c

(Remarks on code availability)

Version 1:

Reviewer comments:

Reviewer #1

(Remarks to the Author)
I thank the authors for their careful and extensive revision and for addressing my earlier concerns in a constructive manner. At the initial stage, I was not fully convinced that the novelty was sufficiently differentiated from prior studies, as phase-change–enabled switchable adhesion has been widely explored, and the additional use of electrical sensing and machine-learning tools did not immediately appear to provide a clear conceptual distinction. In particular, I initially viewed the machine-learning component as potentially ancillary to the core contribution, and I was uncertain whether it materially strengthened the fundamental advance beyond existing approaches.
However, through the revision process, the authors have clarified the central contribution more effectively. The integration strategy is now presented in a way that aligns well with the demonstrated adhesion performance and the system-level demonstrations, and the manuscript reads as a coherent and well-integrated materials–device–system study rather than a collection of loosely connected elements.
Overall, I am satisfied that the revision has substantially improved the manuscript's clarity and positioning, and I support publication in its current form.

(Remarks on code availability)

Reviewer #2

(Remarks to the Author)
The authors have conducted supplementary experiments to answer my concerns. The manuscript was modified well. I have no additional questions.

(Remarks on code availability)

Reviewer #3

(Remarks to the Author)
I co-reviewed this manuscript with one of the reviewers who provided the listed reports. This is part of the Nature Communications initiative to facilitate training in peer review and to provide appropriate recognition for Early Career Researchers who co-review manuscripts.

(Remarks on code availability)

Reviewer #4

(Remarks to the Author)
The authors have responded all the questions I raised previously. The authors have increased the significance of their research by focusing on the sensing capabilities of their adhesive, but the work is still not innovative or novel enough. Some of the answers were not sufficient enough. Additionally, after reading the paper draft I still have major concerns listed below:
1. The whole manuscript is difficult to follow, especially the introduction chapter. The very start of the introduction has long sentences with complicated structure and it feels like the text does not have focus and is a bit all over the place. I recommend the authors would rewrite the draft keeping extra focus in the readability and flow.
2. In Fig. 4 b and c, are the x-axes identical and the events happening simultaneously? It is difficult to match the two graphs since they are not in the same picture or on top of each other. Can authors add some labels so the figures are easier to read? Can you present the resistance and capacitance signal as function of time similar to pull experiment but you re not pulling anything? I would like to see if they behave identically and you re actually measuring just temperature.
3. The smart gripper experiments need more attention. In the Figure 5h, electrical signals from the gripper are shown. The resistance and capacitance follow the heating cycle quite clearly. How can the information about gripping be extracted here? The resistance and capacitance would behave similarly even if the gripper is not picking anything? Fully decoupled experiment is needed where it can be seen that the sensors are capturing attachment and detachment, not just variations in the temperature.
4. In Figure 6 f and g, it is not clear how the legs are actuated, are the figures f and g corresponding to figures c and d? When are they actually detaching and attaching? Can you mark these events to the figure or even overlap with the control signal.
5. In figure S55e, why is the signal so distorted?
In conclusion, I recommend major revision or reject for the manuscript.

(Remarks on code availability)

Version 2:

Reviewer comments:

Reviewer #1

(Remarks to the Author)
[Editor's Note: This reviewer was asked to evaluate the response to Reviewer #4's comments]

The authors answered all the questions of the reviewer satisfactorily.

(Remarks on code availability)

Open Access This Peer Review File is licensed under a Creative Commons Attribution 4.0 International License, which permits use, sharing, adaptation, distribution and reproduction in any medium or format, as long as you give appropriate credit to the original author(s) and the source, provide a link to the Creative Commons license, and indicate if changes were made.
In cases where reviewers are anonymous, credit should be given to 'Anonymous Referee' and the source.
The images or other third party material in this Peer Review File are included in the article's Creative Commons license, unless indicated otherwise in a credit line to the material. If material is not included in the article's Creative Commons license and your intended use is not permitted by statutory regulation or exceeds the permitted use, you will need to obtain permission directly from the copyright holder.
To view a copy of this license, visit https://creativecommons.org/licenses/by/4.0/

Response Letter to Reviewers

Dear Reviewers,

Thank you very much for reviewing our manuscript entitled "Dynamic adhesion switching and sensing functionality in crystallization-controlled eutectogel networks" (Research Article, No. NCOMMS-25-79283). We sincerely thank the reviewers for insightful and helpful comments. We have considered the comments carefully and made all necessary changes to fully address the comments point by point. In the following section, the comments of reviewers are in black color, and our response is in blue while the revision in the formal manuscript is in red.

Reviewer #1 (Remarks to the Author):

Comment: Crystallization-melting mechanism with a real-time adhesion monitoring capability and its wall-climbing robot. While the work includes extensive characterization and impressive demonstrations, I regret to recommend rejection of the manuscript in its current form for publication in Nature Communications. This recommendation is based on several fundamental concerns regarding the study's insufficient novelty, and significant issues with data presentation and scientific rigor that undermine the credibility of the findings.

Response: We sincerely thank you for the rigorous and critical evaluation of our manuscript. We have taken these concerns very seriously and have performed a comprehensive overhaul of the manuscript. We believe these substantial revisions directly address the fundamental concerns raised and significantly elevate the quality of our work.

Comment: The manuscript combines several concepts (materials, mechanism, applications) that are individually well-established in the field. Consequently, it fails to demonstrate a significant breakthrough or a substantial advance over the state-of-the-art.

1. The use of a Urea/Choline Chloride DES is the most common and extensively studied combination, as established by Abbott et al. Chem. Commun. 1, 70 (2003) and utilized in numerous subsequent eutectogel studies (e.g., Zhao et al. J. Mater. Chem. A. 10, 4222 (2022); Yang et al. ACS Appl. Mater. Interfaces 15, 55244 (2023)). More importantly, the strategy of using a crystallization-melting mechanism to switch adhesion has already been reported. The work by Chen et al. ACS Nano, 18, 23968, (2024) demonstrated this with PEG crystallization, and the work by Xu et al. Adv. Sci. 11, 2400938 (2024) is particularly concerning as it not only uses an eutectogel for switchable adhesion but also demonstrates the exact same application of a wall-climbing robot. Furthermore, the core mechanism (controlling adhesion via a temperature-induced stiffness change) is conceptually analogous to the well-known principles of shape memory polymers (glass-to-rubber transition) and phase-separating hydrogels. The authors do not provide any quantitative or qualitative comparison to differentiate their mechanism or performance from these highly relevant prior works (e.g., Fang et al. Adv. Funct. Mater. 33, 2301505 (2023)-hydrogel; Linghu et al. Sci. Adv. 6, aay5120 (2020)-shape memory polymer; Annapooranan et al. PNAS, 121, e2412635121 (2024)-liquid crystal elastomer, making it impossible to assess the claimed advance.

Response: We deeply appreciate the reviewer's meticulous literature review and the challenge

regarding the novelty of our work. We have carefully revised the manuscript and incorporated additional content based on your feedback.

Regarding the novelty of our study, we would like to offer further elaboration. With full respect, we are aware that previous literature has explored switchable adhesion using DES/shape memory polymers/liquid crystal elastomers and its use in climbing robots. However, our goal was not to develop new DES chemistries, but rather to leverage a simple, reliable DES mixture as a platform for system-level innovation by integration of machine-learning-assisted real-time monitoring. By capturing and analyzing adhesion states during the dynamic processes of gripping and climbing, our approach can effectively prevent unexpected failures and enable 'smart maintenance'—capabilities that represent a notable step forward beyond existing studies. Therefore, we propose an integration strategy that bridges the gap between adhesion switching and active state awareness.

Though climbing robots were demonstrated in a few previous reports you mentioned, they lack the real-time sensing of adhesive state during climbing, which is demonstrated in our revised manuscript. Specifically, apart from a system that can form switchable adhesion with surfaces of various materials and roughness, we demonstrate the system capable of real-time sensing of the adhesion interface while simultaneously retaining the flexibility to reprogram adhesion strength. By providing instantaneous data on the contact status and bond strength, this ensures that robotic adhesion can preemptively detect potential failures (e.g., partial detachment), thereby achieving a level of operational safety and autonomous 'smart' maintenance that was previously unattainable with switchable adhesion alone. This integration is a critical advancement, as it enables real-time monitoring of the adhesive interface, ensuring safer robotic operations and facilitating proactive 'smart' maintenance of the system.

We believe our work is not merely a combination of existing concepts, but a fundamental advancement in "intelligent adhesive interfaces" that addresses a critical failure mode in soft robotics.

To address your concerns, we have made the following major revisions:

Direct quantitative comparison: Respectfully, we have read through the literature you mentioned [Abbott et al. Chem. Commun. 1, 70 (2003), Zhao et al. J. Mater. Chem. A. 10, 4222 (2022); Yang et al. ACS Appl. Mater. Interfaces 15, 55244 (2023), Chen et al. ACS Nano, 18, 23968 (2024), Xu et al. Adv. Sci. 11, 2400938 (2024), Fang et al. Adv. Funct. Mater. 33, 2301505 (2023)-hydrogel; Linghu et al. Sci. Adv. 6, aay5120 (2020)-shape memory polymer; Annapooranan et al. PNAS, 121, e2412635121 (2024)-liquid crystal elastomer], although some of these reports are with switchable adhesion and demonstration of climbing robots, but the integration of sensing adhesion state in these applications is not reported. We have added new comparative analysis tables in Supplementary Information by comparing our eutectogel with representative literature with switchable adhesion, and their application in smart gripper and climbing robot while we address this overlap in present study.

Table S2. Performance comparison of adhesion-based intelligent grippers

Gripper type	Preload (N)	Gripper diameter (mm) or area	Max pull - off force (N)	Max lifting ratio	Diameter ratio limits	Surface conditions(surface roughness)	Time to acquire grip (s)	Power required to maintain grasp (W)	Reference
Granular jamming	150	86	100	N/A	0.1-0.85	N/A	0.1-1.1	N/A	10
Gecko-inspired gripper	N/A	180	43	200	> 0.5	N/A	<0.1	0	11
Elastomeric microfibrillar soft adhesive gripper	N/A	18	78 mN	N/A	> 1	Need relatively smooth surface	N/A	4.1 J/m^2<br>3.3 J/m^2	12
Magnetically switchable soft suction	1.5	20	7.5	80	>0.4	17.7	10	0	13
shape memory polymer gripper	N/A	7 – 60 (Depends on the shape and size of the shape memory polymer)	34.8	78.5	< 0.01<br>> 1	1 μm - 1 cm	N/A Depends on the heating time	0	14
Tensile-induced buckling gripper	65% stretching strain	240 x 40	0.012	N/A	< 0.5	N/A	N/A Depends on the stretching time	N/A The tensile deformation must be maintained.	15
Finger inspired rigid-flexible-soft structure	12	31 cm	4.0	0.11 (Two-finger clamp) 0.128 (Three-finger clamp)	0.806	N/A	3	N/A Depends on the gas actuator power	16
supramolecular gel-elastomer system	0	16 × 16	0.54 (2.12 kPa)	671	< 1	0.01-10	30	N/A<br>300 V~ 1 kV	17
Curved loop strip soft gripper	0	N/A Depends on the length of the strip		1.7×10^4	< 1	N/A	1.76	N/A	18
Magnetoactive bistable soft gripper	0	N/A	0.029 N	N/A	< 1	N/A	10	N/A	19
Our work	0 – 5 N	2 cm Depends on the length of the eutectogel size	100 N	88.23	> 1	0.01- 1 cm	10 – 20 Depends on the heating time	20V* 0.5 A Depends on the heating time	

Table S4. Recent reports on climbing robots with intelligent adhesion

Climbing robots: adhesion mechanism	Adhesive material	Adhesion Switching Performance	Wall material (Whether it adapts to different surface)	Wall Roughness (Whether suitable for rough surfaces)	Crawling speed	Payload (payload-to-weight ratio)	Energy density	Reference
Pneumatic	Ecoflex	6-8N	glass, sand paper, Acrylics	Smooth and semi-smooth wall (roughness <20 μm)	2.86 cm/min	40g(self mass) 200 g (5 times body weight)	N/A	20
Dielectric elastomer	Dielectric elastomer VHB	3.0533 N (32mm Diameter) 5.09 kPa – 0 kPa	Wood, paper, glass	Exact roughness value unknown, relatively flat.	530.76 cm/min (0.75 body lengths)	10 g	V = 6 kV 23 Hz	21
Electrochemical	Borate ester hydrogels	11.5 kPa - 0 kPa	Multiple materials: glass, metal, ceramics, plastics	Exact roughness value unknown, relatively flat	7.8 cm/min (0.8 body lengths)	175 g (self mass)	V = 3 V 5 s	22
Electro-permanent magnet	Permanent magnet	535.4 N (41*41mm^2) 318.26 kPa-0kPa	Ferromagnetic	Tolerant to rough surface	4200 cm/min (2.12 body lengths)	8 kg (self mass) 2 kg (0.4 body weight)	V = 29.6 V I = 35.18 A 4.07 J	23
Mushroom-shaped dry adhesion and microspike bioadhesive	PDMS and chitosan hydrogel	1 mN (0.3 mm^2) 3.3 kPa-0.33 kPa	Mucus tissue	Relatively smooth, roughness < 30 μm	N/A	20 times body weight, 3 times volume	N/A	24
Growing-climbing mechanism	Microstructured biofilms	7-10 kPa	Multiple materials	Tolerant to rough surface	N/A	521.1g 1330g (1.55 body weight)	N/A	25
Water-enhanced sliding suction mechanism	Water-silicone (capillary forces)	N/A	Polyoxymethylene, PMMA, aluminium alloy	Exact roughness value unknown, relatively flat.	114 cm/min (0.475 body length)	96 g (self mass) 1 kg (10.42 body weight)		26
Electroadhesive	Electroadhesive footpad	3N (4 kV , 39*84 mm^2) 0.92 kPa	Multiple materials	Exact roughness value unknown, relatively flat.	6-90 mm (self length) 24.6 cm/min (2.73 body length)	0.2-3 g(self mass)	V = 3 kV	27
Magnetic-controlled adhesion	magnetically triggered MR elastomers	1.35 N/cm^2-2.93N/cm^2 29.3kPa	Multiple materials: Ecoflex, silicon wafer, pigskin	Exact roughness value unknown, relatively flat.	N/A	N/A	0 T-0.36 T I = 9 A	28
Directional microprismatic structure bioadhesion	PDMS Microstructural friction	<70kPa	Multiple materials	Exact roughness value unknown, relatively flat.	1.4 body length /s (84 body length /min)	0.63g and 0.24g (self mass)		29
Our work	Phase changing eutectogels	Crstal state:10 kPa Melt state: 100kPa M-C state: 1000kPa	Multiple materials, including metals, plastics, ceramics, glass, etc.	Tolerate to rough surfaces, roughness can be up to millimeters.	8cm/min (0.65 body lengths)	500g (self mass) 200g (0.4 body weight)	V = 6 V I = 2 A >10 s	

We elaborate this point in the manuscript with the following text.

On page 3: Furthermore, the seamless integration of switchable adhesion and real-time sensing remains a critical challenge in the development of advanced climbing robots. This integration will be essential for precisely monitoring the interface contact state and adhesion force, thereby preventing catastrophic falls caused by unreliable attachment under nonideal conditions. Such advancements will enable a higher level of bionic intelligence, bridging the gap between artificial systems and the sophisticated sensory-motor functions of natural organisms like geckos.

To overcome these limitations, here we develop crystallizable eutectogels that integrate electrothermally switchable adhesion with intrinsic electrical sensing. Eutectogels are a kind of gels consisting of polymer network with embedded deep eutectic solvents (DES)[51, 52, 53]. Most

of them are conductive and have phase transition. The reversible crystallization-melting transition of DES enables a synergistic mechanism of physical shape locking and switchable molecular interactions with adherends, thereby achieving strong and reversible adhesion across diverse surfaces. Upon electrothermal activation, the gel rapidly transitions from a crystalline state (10^1 kPa) to a molten state (10^2 kPa). Through the M2C cooling process, its adhesion properties are tuned between 10^2 kPa and 10^3 kPa, sufficient to support robotic climbing on vertical surfaces with varied materials and roughness. The switching mechanism is elucidated through systematic investigation of the crystalline, molten, and transition states, supported by experimental characterization and molecular simulations. Notably, the inherent conductivity of eutectogels allows real-time monitoring of adhesion states via electrical parameters such as resistance and capacitance, which can be wirelessly transmitted through Bluetooth. Machine learning algorithms further enhance the performance by predicting adhesion states from electrical signals. The deep eutectic solvents used here are also environmentally friendly. The ability of eutectogels to achieve conformal contact in the molten state underpins robust, reversible adhesion on non-specific surfaces, highlighting their potential for intelligent soft robotic systems and adaptive structural applications.

We have cited the mentioned reports in the revised manuscript.

On page 3: More recently, shape-memory polymers and liquid crystal elastomers have emerged as candidates for switchable adhesion[8, 33, 34, 35, 36, 37, 38].

[35] Linghu C, *et al.* Universal SMP gripper with massive and selective capabilities for multiscaled, arbitrarily shaped objects. *Science Advances* **6**, eaay5120 (2020).

[36] Chen L, *et al.* Phase-Change Stamp with Highly Switchable Adhesion and Stiffness for Damage-Free Multiscale Transfer Printing. *ACS Nano* **18**, 23968-23978 (2024).

[38] Annapooranan R, Yeerella RH, Chambers RJ, Li C, Cai S. Soft elasticity enabled adhesion enhancement of liquid crystal elastomers on rough surfaces. *Proceedings of the National Academy of Sciences* **121**, e2412635121 (2024).

On page 3: Other notable advances include the use of crystal gels with reversible phase transitions that can form conformal contact in the soft state and render high adhesion performance in the glassy state, thereby enabling switchable adhesion forces[40, 41, 42, 43, 44, 45, 46, 47]. Besides switchable adhesion, another lesson we can learn from the creatures in nature, e.g., reptiles, is the integrated sensing and muscle regulation, affording coordinated motion[48, 49]. Wang, Li and coworkers demonstrated remarkable progress that the integrated sensing and switchable adhesion during gripping using capacitive force sensor and stiffness-tunable dry adhesives/micro-adhesive structures[48, 50].

[46] Xu H, Li H, Zhang Y, Guan Y, Zhang Y. Strong and Thermo-Switchable Gel Adhesion Based on UCST-Type Phase Transition in Deep Eutectic Solvent. *Advanced Science* **11**, 2400938 (2024).

[47] Fang Y, *et al.* Phase Change Hydrogels for Bio-Inspired Adhesion and Energy Exchange Applications. *Advanced Functional Materials* **33**, 2301505 (2023).

On page 4: Eutectogels are a kind of gels consisting of polymer network with embedded deep

eutectic solvents (DES)[51, 52, 53].

[51] Abbott AP, Capper G, Davies DL, Rasheed RK, Tambyrajah V. Novel solvent properties of choline chloride/urea mixtures. *Chemical Communications*, 70-71 (2003).

[52] Zhao Y*, et al.* Quasi-solid conductive gels with high thermoelectric properties and high mechanical stretchability consisting of a low cost and green deep eutectic solvent. *Journal of Materials Chemistry A* **10**, 4222-4229 (2022).

[53] Yang J-Y*, et al.* Biocompatible, Antibacterial, and Stable Deep Eutectic Solvent-Based Ionic Gel Multimodal Sensors for Healthcare Applications. *ACS Applied Materials & Interfaces* **15**, 55244-55257 (2023).

In addition, to demonstrate the generalizability of this DES strategy, we add supplementary data on other eutectogels using five representative classes of DES. These systems include: (i) amine + quaternary ammonium salt, (ii) alcohol + quaternary ammonium salt, (iii) metal halide + quaternary ammonium salt, (iv) acid + quaternary ammonium salt, and (v) alcohol + acid. Specifically, hydroxyethyl acrylamide (HEAA) and N,N-dimethylacrylamide (DMA) were employed as monomers for hydrophilic and hydrophobic eutectic solvents, respectively, both maintained at a consistent mass fraction of 30 wt%.

Corresponding revision in the manuscript:

On page 19: While initial characterizations focused on urea/choline chloride-based crystalline eutectogels, we conducted a systematic investigation across diverse eutectic solvent systems to verify the universality of this intelligent adhesion behavior. For instance, substituting choline chloride with betaine yielded similar crystallization characteristics; by modulating the urea:betaine molar ratio (e.g., 2:1 vs. 1:2), we successfully transitioned the resulting eutectogels from an amorphous to a crystalline state at room temperature (**Supplementary Fig. S42**). To further substantiate this strategy, we expanded our scope to include various chemical motifs, such as alcohol/quaternary ammonium salts (sorbitol/choline chloride), metal halides/quaternary ammonium salts (zinc chloride/choline chloride), organic acids/quaternary ammonium salts (lauric acid/tetraoctylammonium bromide), and alcohol/acid systems (menthol/lauric acid). In each case, precisely tuning the composition ratio allowed for the successful preparation of room-temperature crystallizing eutectogels (**Supplementary Figs. S43-46**). These results confirm that our strategy is highly robust and universally applicable, allowing for the selection of specific eutectic solvents, such as hydrophobic systems for underwater applications, to meet diverse environmental demands.

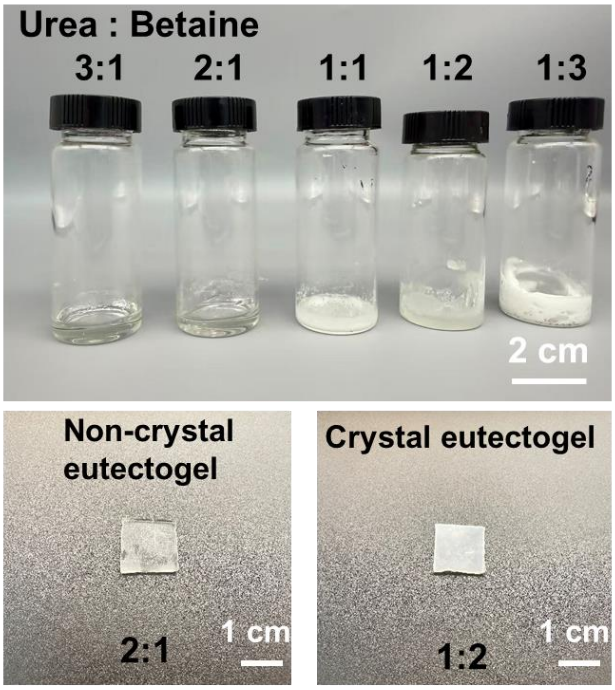

Fig. S42. Crystallization behavior of eutectic solvents prepared with urea and betaine as hydrogen bond donors and acceptors, respectively, and comparison with corresponding eutectogels (sample information: Non-crystal eutectogel $EG_{2\text{-}30}$ and crystal eutectogel $EG_{0.5\text{-}30}$).

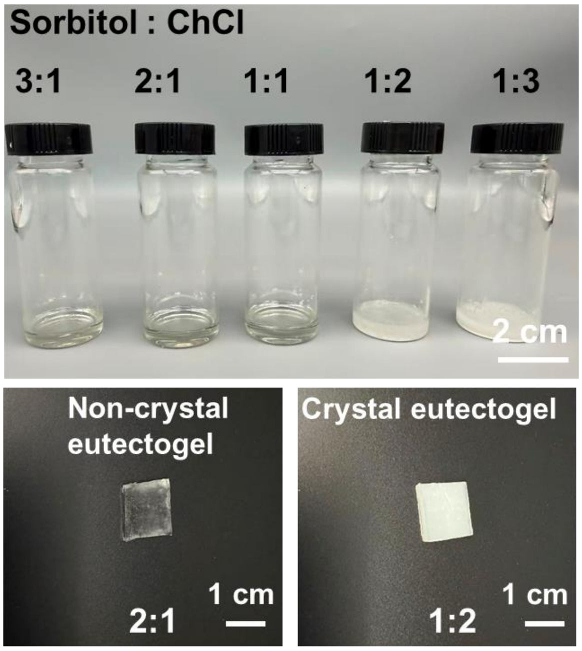

Fig. S43. Crystallization behavior of eutectic solvents prepared with sorbitol and choline chloride as hydrogen bond donors and acceptors, respectively, and comparison with corresponding eutectogels (sample information: Non-crystal eutectogel $EG_{2\text{-}30}$ and crystal eutectogel $EG_{0.5\text{-}30}$).

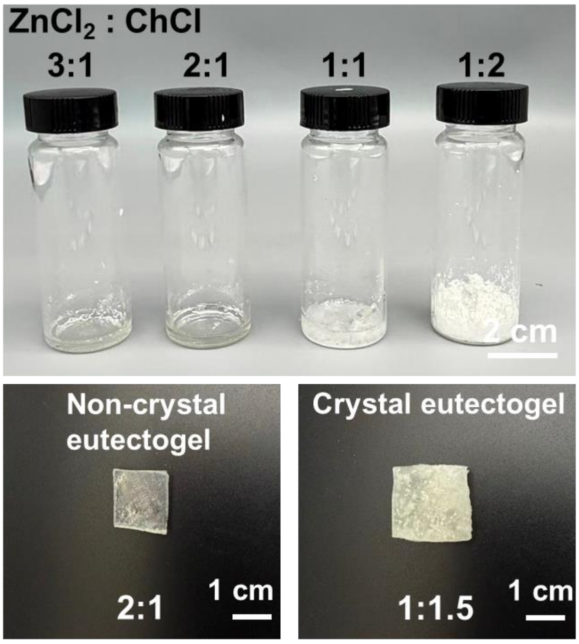

Fig. S44. Crystallization behavior of eutectic solvents prepared with $ZnCl_2$ and choline chloride as hydrogen bond donors and acceptors, respectively, and comparison with corresponding eutectogels (sample information: Non-crystal eutectogel $EG_{2\text{-}30}$ and crystal eutectogel $EG_{0.5\text{-}30}$).

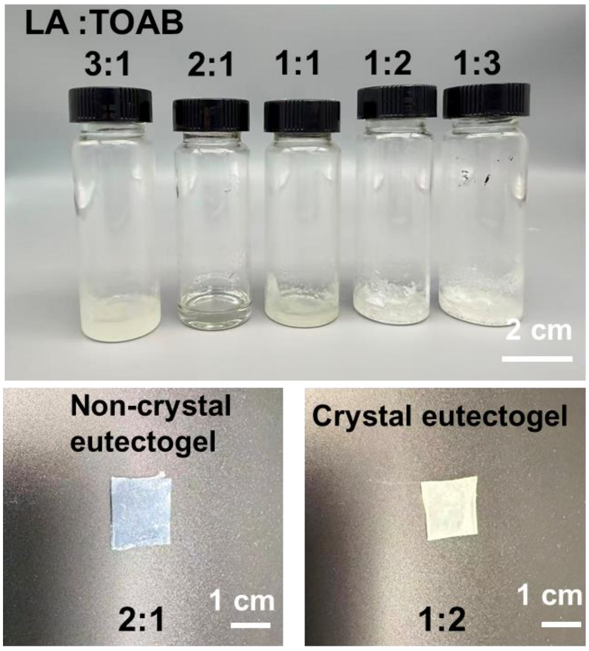

Fig. S45. Crystallization behavior of eutectic solvents prepared with lauric acid and Tetra-octylammonium bromide as hydrogen bond donors and acceptors, respectively, and comparison with corresponding eutectogels (sample information: Non-crystal eutectogel $EG_{2\text{-}30}$ and crystal eutectogel $EG_{0.5\text{-}30}$, hydrophobic monomer: DMA).

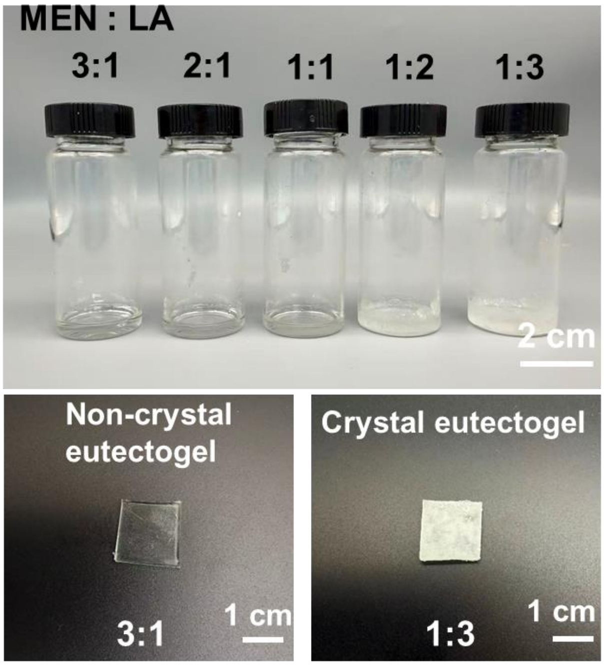

Fig. S46. Crystallization behavior of eutectic solvents prepared with menthol and lauric acid as hydrogen bond donors and acceptors, respectively, and comparison with corresponding eutectogels (sample information: Non-crystal eutectogel $EG_{3\text{-}30}$ and crystal eutectogel $EG_{0.33\text{-}30}$, hydrophobic monomer: DMA).

We believe this transition from "switchable adhesion" to "intelligent, failure-predictive adhesion" represents a substantial conceptual progress that is highly relevant to the multidisciplinary audience of Nature Communications.

Comment 2. The concept of an integrated adhesion monitoring system has been explored in prior works (e.g., Wang et al. Sci. Adv. 9, adf4051 (2023); Li et al. Chem. Eng. J. 523, 168354 (2025), which demonstrated real-time sensing during pick-and-place operations. Critically, the authors fail to integrate this sensing capability into their primary application (the climbing robot), which operates on non-conductive surfaces where the EDL-based sensing mechanism is not viable. The three demonstrations (pick-and-place, sensing, robot) are presented as isolated showcases rather than an integrated, synergistic system. Had the authors demonstrated a robot capable of sensing its own adhesion status, the impact would have been far greater. As it stands, this feature feels disconnected and its novelty is diluted.

Response: We sincerely thank the reviewer for these insightful and constructive comments. We agree that integrating sensing capabilities directly into the robotic platform significantly enhances the system's synergy and practical impact. Following your valuable suggestions, we have performed the following revisions.

1. Literature Review and Clarification of Novelty. We have included the recommended literature [Wang et al. Sci. Adv. 9, adf4051 (2023); Li et al. Chem. Eng. J. 523, 168354 (2025)] in our reference list. These works provide pioneering examples of integrating sensing with switchable adhesion. In the revised Introduction section, we have explicitly discussed these

advancements to better contextualize our work.

On page 3: Besides switchable adhesion, another lesson we can learn from the creatures in nature, e.g., reptiles, is the integrated sensing and muscle regulation, affording coordinated motion[46, 47]. Wang, Li and coworkers demonstrated remarkable progress that the integrated sensing and switchable adhesion during gripping using capacitive force sensor and stiffness-tunable dry adhesives/micro-adhesive structures[46, 48].

2. Integration of sensing into the robotic gripper and climbing system. To address the concern regarding "isolated showcases," we have further integrated the smart gripper and the climbing robot with real-time sensing functionality. Rather than treating them as separate demonstrations, we now present a synergistic system where the eutectogel serves both as the adhesive and the sensory medium.

While the reviewer rightly points out the limitations of EDL-based sensing on non-conductive surfaces, our system primarily leverages the capacitance and resistance fluctuations of the eutectogel itself. Specifically, we adhered part of the gripper with copper to probe the change of resistance and capacitance of the system during gripping. These signals respond to the mechanical deformation and temperature-induced conductive changes during the "contact-load-detach" cycle, which allows for state monitoring even on non-conductive substrates. We demonstrated that electrical signals provide distinct, repeatable fluctuations during contact, lifting, and release phases, allowing the system to "know" if an object is securely held.

We made revision in the manuscript:

On page 25: Additionally, due to the intrinsic ionic conductivity arising from the ionic composition of eutectogels, monitoring of electrical signals enables the tracking of the gripper's own state and the magnitude of contact forces during the object's contact and separation process. **Fig. 5g** and **5h** illustrate the schematic of the gripper detecting electrical signals and the curve of electrical signal changes during the gripping process. Part of the eutectogel is covered with conductive copper, therefore the conductivity/capacitance signal can be probed during gripping. The fluctuations in the electrical signal provide a direct visual representation of the gel's state on the gripper. When contacting and releasing objects, the electrical signal exhibits distinct opposite changes (**Fig. 5h**). Further testing with weights of varying masses generates different pressures to assess the gripper's sensitivity to contact force magnitude. The ratio of electrical signal fluctuations produced by weights ranging from 20 to 500 grams clearly demonstrates an approximate multiplicative relationship between different force magnitudes (**Supplementary Fig. S55**).

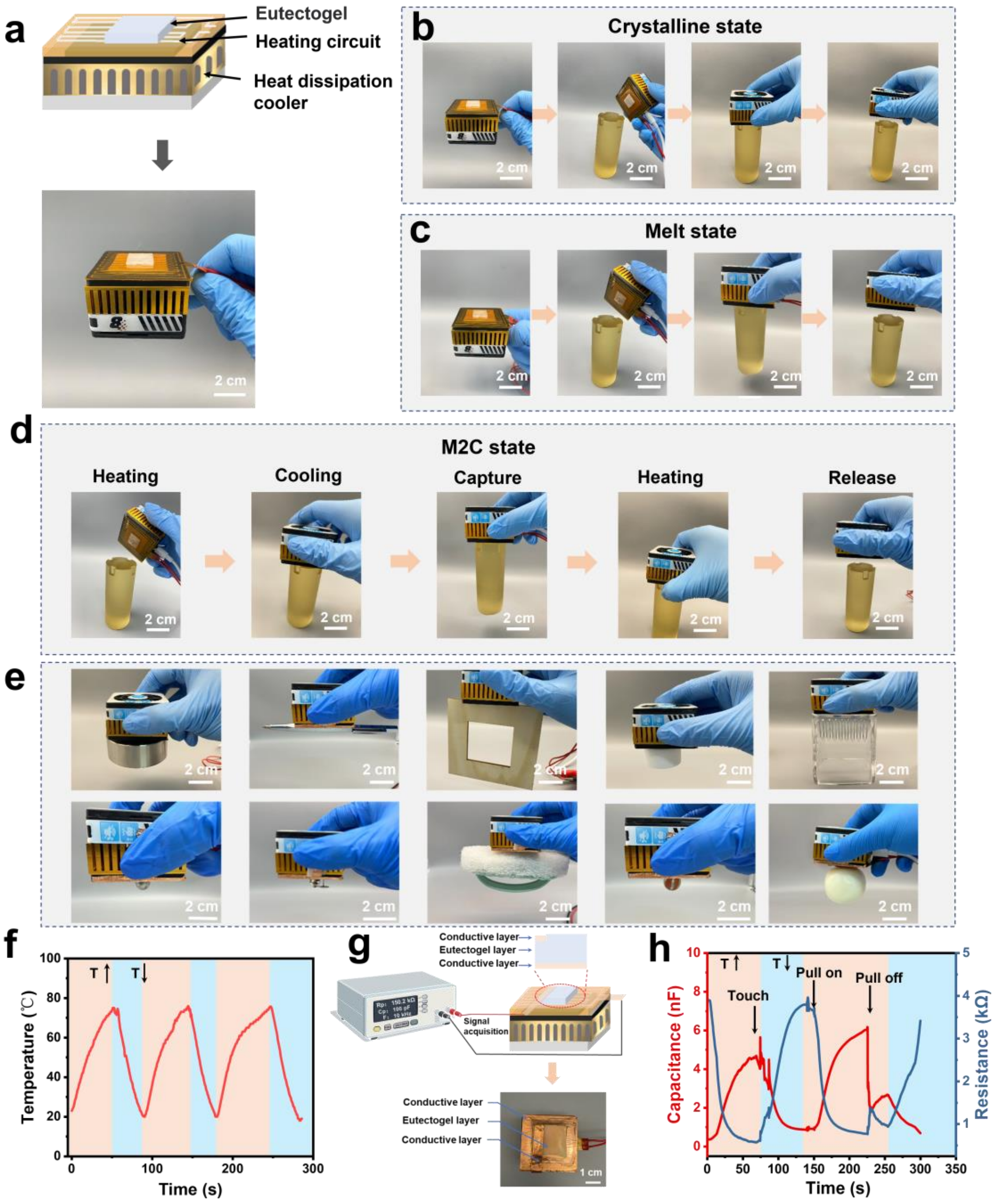

Fig. 5 Development of smart adhesive grippers based on crystalline eutectogels. (a) Schematic diagram of an intelligent adhesive gripper structure based on eutectogel. (b) In the crystalline state, eutectogel smart grippers have difficulty grasping target objects through adhesion. (c) The eutectogel smart gripper in the melt state tends to lose its adhesion to the target object during lifting. (d) Digital photos of the process of picking up target items using eutectogel smart gripper based on the M2C process. (e) The eutectogel ($EG_{0.67\text{-}30}$) smart gripper grasps target objects with diverse surfaces made from various materials, including metal block and sphere, PTFE hollow cylinder, wooden frame, prismatic metal pen, glass cup and glass sphere, metal thumbtack, porous sponge and egg. (f) Temperature curve of eutectogel ($EG_{0.67\text{-}30}$) during heating and cooling in the operation of the eutectogel smart gripper. (g) Underlying mechanism diagram of electrical signals monitored by a digital bridge during the operation of a

eutectogel ($EG_{0.67\text{-}30}$) holder. (h) Electrical signal changes during the process of the eutectogel ($EG_{0.67\text{-}30}$) smart gripper grasping target objects.

For the climbing robot: In the revised manuscript, we have integrated adhesion sensing into the climbing robot and implemented real-time monitoring during the alternating tripod gait. The signal spikes correlate precisely with the contact and separation of the central and lateral legs from the wall, providing the robot with "proprioception" of its adhesion status during locomotion.

We have corresponding revision in the "Development and application of wall-climbing robots" section:

On page 29: The robot demonstrated stable climbing on rough vertical surfaces (**Supplementary Fig. 59**), aided by optimization of the gel heating/cooling balance. The state of the eutectogel in the robot's foot and the robot's crawling posture are monitored via electrical signals. Similar to the gripper section, part of the adhesive pad of climbing robot contacting wall surface is covered with conductive copper, such that the robot's feet can sense adhesive status in all scenarios not only on conductive surface. As shown in **Fig. 6e** and **6f**, when the middle foot (Foot A) lifts, the eutectogel heats up, causing its resistance to decrease and capacitance to increase. Upon re-contact with the wall surface, the capacitance signal exhibits a marked increase. As the temperature drops, capacitance decreases and resistance increases. When the middle foot detaches from the wall, electrical signal fluctuations occur, characterized by decreased capacitance and increased resistance. This motion is synchronized with the heating and cooling cycles of the electrothermal gel layer (**Fig. 6e, Movie S8**). The heating cycles of the main and lateral feet are maintained in opposite phases: when one foot is lifted, its gel layer is heated to induce melting; when it is loaded, heating ceases and cooling is facilitated via the stainless-steel layer and wall contact.

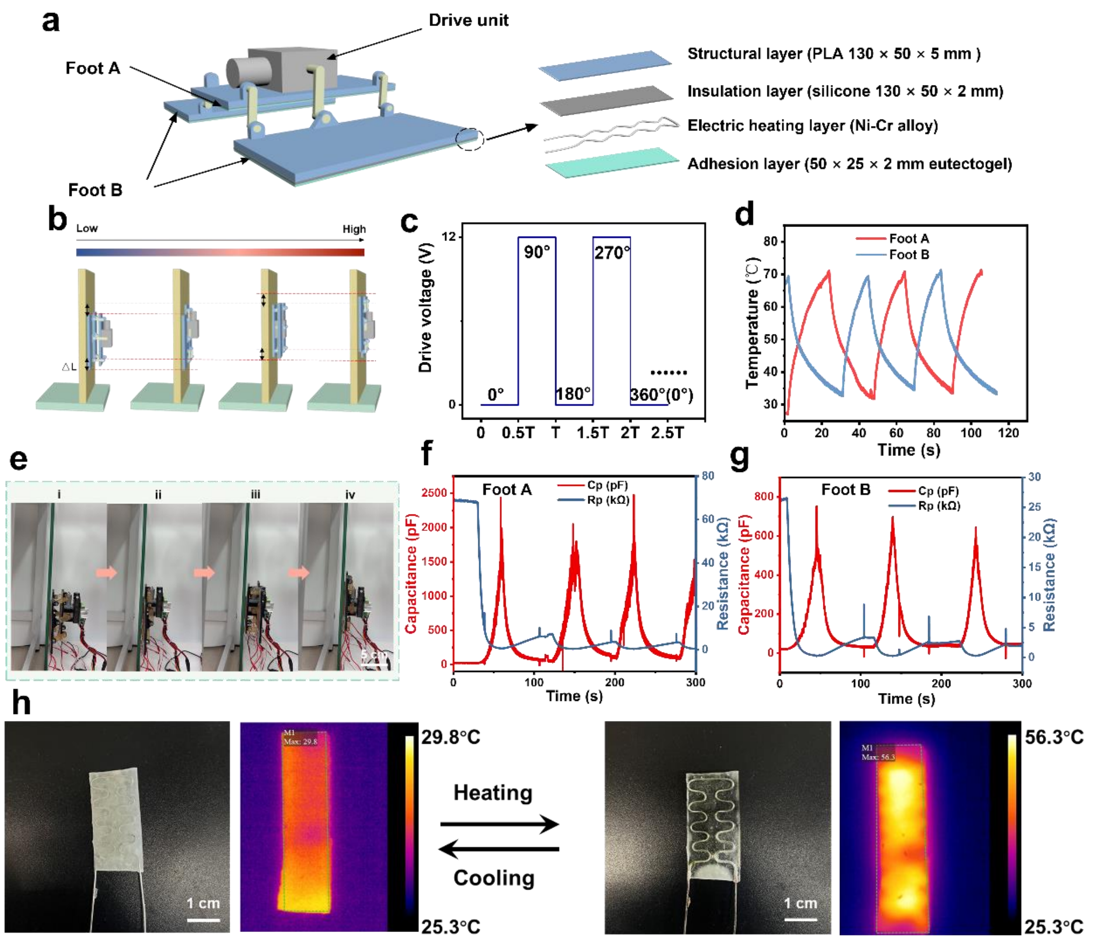

Fig. 6 Design principle and fabrication of the wall-climbing robot. (a) Illustration of the mechanical design of the three-feet wall-climbing robot (left), and layout of the foot including a supporting layer (size: 130 mm × 50 mm × 5 mm), an insulation layer (size: 50 mm × 25 mm × 2 mm), a metallic layer, and the eutectogel ($EG_{0.67\text{-}30}$) layer (size: 50 mm × 25 mm × 2 mm). (b) Schematic diagram of the climbing principle of the wall-climbing robot. The robot climbing motion is realized by human-coordinated control. Over each cycle, the soft robot climbs up by a distance of ΔL. (c) The drive motor rotates the shaft at 12V. Controlling on-off of the drive circuit realizes the intermittent rotation of the dual axes of the gear motor between 0-360°. (d) Temperature curve of eutectogel heating and cooling in robotic feet during three-legged robot crawling. (e) Images of a wall-climbing robot climbing on a 90° glass wall. (f) Electrical signal changes during the heating adhesion-cooling crystallization-heating peeling process of the middle foot (Foot A) of the wall-climbing robot. (g) Electrical signal changes during the heating adhesion-cooling crystallization-heating peeling process of the side foot (Foot B) of a wall-climbing robot. (h) Infrared images probing the heating process of eutectogel with a conductive circuit embedded in the gel ($EG_{0.67\text{-}30}$).

We believe these additional experiments and the refined discussion effectively demonstrate the synergy of our system, addressing the perceived disconnection between sensing and mobility.

Comment 3. The manuscript does not provide a compelling case for the performance superiority of this system. For instance, the climbing speed of the robot (~8 cm/min) is not

benchmarked against other state-of-the-art climbing robots to demonstrate an advantage. The extensive molecular-level analyses (MD, NMR, SAXS, FTIR), while well-executed, primarily serve to confirm expected physical phenomena (i.e., that crystallization restricts molecular motion) rather than providing fundamentally new scientific insights that lead to a performance breakthrough.

Response: We sincerely thank you for this critical evaluation. We acknowledge that the climbing speed of our robot is relatively modest (~8 cm/min) compared to a few reports. However, we believe that the advance of our system is evaluated through its unique balance of versatility, safety, and operational efficiency rather than raw speed alone. We have addressed these concerns as follows:

Benchmarking performance (addressing speed vs. versatility) As suggested, we have added a comprehensive comparison with existing climbing robots in **Supplementary Table S3**. While robots using electromagnetic or vacuum adhesion offer higher speeds, they often struggle with non-conductive surfaces or high energy consumption.

Our robot leverages the conformal nature of the gel phase transition, endowing it with high adaptability to traverse a much wider variety of wall materials and surface roughness. In addition, unlike dielectric-elastomer or magnetic-actuated robots requiring high voltages (100 V – 1000 V), our system operates safely at a mere 6 V. More importantly, we have integrated real-time adhesion status sensing, a feature that provides a safety layer often missing in faster, purely mechanical systems.

Our revisions to the manuscript are as follows:

On page 28: Wall-climbing robots play a pivotal role in diverse applications, such as infrastructure inspection. A comprehensive comparison between our robot and state-of-the-art systems is provided in Supplementary Table S4. While the current climbing speed of our robot is relatively modest compared to some existing platforms, it offers a balance of versatility and safety. Specifically, our system demonstrates robust adaptability to various wall materials and surface roughness while operating under a relatively low voltage of 6 V. Most importantly, the integration of switchable adhesion with real-time adhesion status sensing, provides a critical safety layer that prevents catastrophic falls—a feature often absent in conventional designs.

Table S4. Recent reports on climbing robots with intelligent adhesion

Climbing robots: adhesion mechanism	Adhesive material	Adhesion Switching Performance	Wall material (Whether it adapts to different surface)	Wall Roughness (Whether suitable for rough surfaces)	Crawling speed	Payload (payload-to-weight ratio)	Energy density	Reference
Pneumatic	Ecoflex	6-8N	glass, sand paper, Acrylics	Smooth and semi-smooth wall (roughness <20 μm)	2.86 cm/min	40g(self mass) 200 g (5 times body weight)	N/A	20
Dielectric elastomer	Dielectric elastomer VHB	3.0533 N (32mm Diameter) 5.09 kPa – 0 kPa	Wood, paper, glass	Exact roughness value unknown, relatively flat.	530.76 cm/min (0.75 body lengths)	10 g	V = 6 kV 23 Hz	21
Electrochemical	Borate ester hydrogels	11.5 kPa - 0 kPa	Multiple materials: glass, metal, ceramics, plastics	Exact roughness value unknown, relatively flat	7.8 cm/min (0.8 body lengths)	175 g (self mass)	V = 3 V 5 s	22
Electro-permanent magnet	Permanent magnet	535.4 N (41*41mm^2) 318.26 kPa-0kPa	Ferromagnetic	Tolerant to rough surface	4200 cm/min (2.12 body lengths)	8 kg (self mass) 2 kg (0.4 body weight)	V = 29.6 V I = 35.18 A 4.07 J	23
Mushroom-shaped dry adhesion and microspike bioadhesive	PDMS and chitosan hydrogel	1 mN (0.3 mm^2) 3.3 kPa-0.33 kPa	Mucus tissue	Relatively smooth, roughness < 30 μm	N/A	20 times body weight, 3 times volume	N/A	24
Growing-climbing mechanism	Microstructured biofilms	7-10 kPa	Multiple materials	Tolerant to rough surface	N/A	521.1g 1330g (1.55 body weight)	N/A	25
Water-enhanced sliding suction mechanism	Water-silicone (capillary forces)	N/A	Polyoxymethylene, PMMA, aluminium alloy	Exact roughness value unknown, relatively flat.	114 cm/min (0.475 body length)	96 g (self mass) 1 kg (10.42 body weight)		26
Electroadhesive	Electroadhesive footpad	3N (4 kV , 39*84 mm^2) 0.92 kPa	Multiple materials	Exact roughness value unknown, relatively flat.	6-90 mm (self length) 24.6 cm/min (2.73 body length)	0.2-3 g(self mass)	V = 3 kV	27
Magnetic-controlled adhesion	magnetically triggered MR elastomers	1.35 N/cm^2-2.93N/cm^2 29.3kPa	Multiple materials: Ecoflex, silicon wafer, pigskin	Exact roughness value unknown, relatively flat.	N/A	N/A	0 T-0.36 T I = 9 A	28
Directional microprismatic structure bioadhesion	PDMS Microstructural friction	<70kPa	Multiple materials	Exact roughness value unknown, relatively flat.	1.4 body length /s (84 body length /min)	0.63g and 0.24g (self mass)		29
Our work	Phase changing eutectogels	Crstal state:10 kPa Melt state: 100kPa M-C state: 1000kPa	Multiple materials, including metals, plastics, ceramics, glass, etc.	Tolerate to rough surfaces, roughness can be up to millimeters.	8cm/min (0.65 body lengths)	500g (self mass) 200g (0.4 body weight)	V = 6 V I = 2 A >10 s	

In accordance with your suggestion, we have relocated the routine molecular characterization data (MD, NMR, SAXS, and FTIR) to Supplementary Information (page 38-47). This restructuring allows our manuscript to focus more sharply on the central scientific narrative. By streamlining the presentation, we believe the fundamental novelty and the supplementary characterization of our eutectogels are now conveyed with greater clarity.

Comment 4. The manuscript's structure is difficult to follow. A clear, logical thread connecting material optimization to final application performance is absent (which eutectogel composition exhibited the most pronounced switchable adhesion capability, as determined through experimental and analytical investigations). The manuscript would benefit from a significant reduction in the number of main-text figures, focusing only on the most critical data and moving supporting results to the SI.

Response: We sincerely appreciate the reviewer's constructive feedback regarding the manuscript's structure and clarity. We have performed a comprehensive revision to establish a more logical thread from material optimization to robotic application.

Our specific actions are as follows:

We first introduced the fabrication process of eutectogels and investigation of the mechanical properties. We then focus on the preparation and structural characterization (DSC, XRD, optical properties, tensile tests, DMA etc) of eutectogels in this section. Then we proceed to the section of “Switchable adhesion of the eutectogel due to physical shape locking and surface chemistry”. Here we adjusted the interpretation flow by first discussing the M2C adhesion mechanism, then investigating the influence of DES composition on the adhesion performance and establishing U/C molar ratio of 1:1.5 as the optimal DES for subsequent studies. Then we studied the temperature-assisted switchable adhesion of eutectogels and underlying mechanism with molecular simulation.
Corresponding revision can be found on page 13-16 in the revised manuscript.

The influence of composition on the adhesion strength of eutectogels is further quantified to optimize the composition (**Fig. 3b**). For U/C > 1:1, the gels ($EG_{1\text{-}30}$) are amorphous at room temperature. For samples with different DES, the adhesion strength at room temperature decreases with increasing urea content-from 276.6 kPa at $EG_{3\text{-}30}$ to 118.3 kPa at $EG_{1.5\text{-}30}$. This reduction is attributed to the lower density of amino groups available for hydrogen bonding with substrates as the urea content decreases. In contrast, when U/C < 1:1, the eutectogels exhibit crystalline features. The adhesion strength first increases and then decreases with less urea content, reaching a maximum of 1.06 MPa at $EG_{0.67\text{-}30}$ before declining to 686.3 kPa at $EG_{0.5\text{-}30}$. The initial enhancement is ascribed to stronger gel cohesion induced by low-temperature crystallization, while further reduction in urea weakens gel–substrate interactions, lowering adhesion strength. Changes in eutectogel cohesion due to solvent composition variations are characterized through mechanical testing. At a U/C molar ratio of 1:1.5, the eutectogel ($EG_{0.67\text{-}30}$) exhibited significantly lower modulus compared to gels with $EG_{0.5\text{-}30}$ and $EG_{0.8\text{-}30}$ (**Supplementary Fig. S17**). Therefore, the U/C molar ratio of 1:1.5 is selected as the DES for subsequent studies ($EG_{0.67\text{-}30}$), unless otherwise specified. Due to the inherent hydrogen bonding provided by the DES at the interface, the adhesion force maintains a baseline of ~100 kPa. This suggests that the chemical nature of the solvent is the governing factor for adhesion in the melted state, limiting the impact of polymer network modifications.

Given that eutectogel adhesion is governed by temperature-mediated phase transitions, the role of crystallization is evaluated by measuring M2C adhesion at different temperatures (**Fig. 3c**). From −20 °C to 80 °C, M2C adhesion decreases monotonically with increasing temperature, due to progressive loss of crystallinity and thermal disruption of hydrogen bonding, both of which diminish gel cohesion and adhesive strength. Notably, crystalline eutectogels can be annealed into an amorphous phase, enabling recyclability through repeated melt-crystallization cycles. As shown in **Supplementary Fig. S23**, adhesion strength on glass substrates remains stable over multiple adhesion–detachment cycles. Similarly, repeated shear-loading tests with maximum deformation of 0.1 mm demonstrate that eutectogels endured over 100 dynamic cycles without degradation of bonding performance (**Supplementary Fig. S24**). Heavy-load testing further confirms their robustness: a eutectogel pad with an area of only 2.5 cm^2 supported a 7.5 kg dumbbell in the M2C state (**Supplementary Fig. S25**). These findings

highlight the exceptional fatigue resistance, recyclability, and mechanical adaptability of phase-change eutectogel adhesives. Moreover, because the melting point of the DES can be precisely tuned through composition design, the switching temperature of adhesion can be programmed to meet specific application requirements.

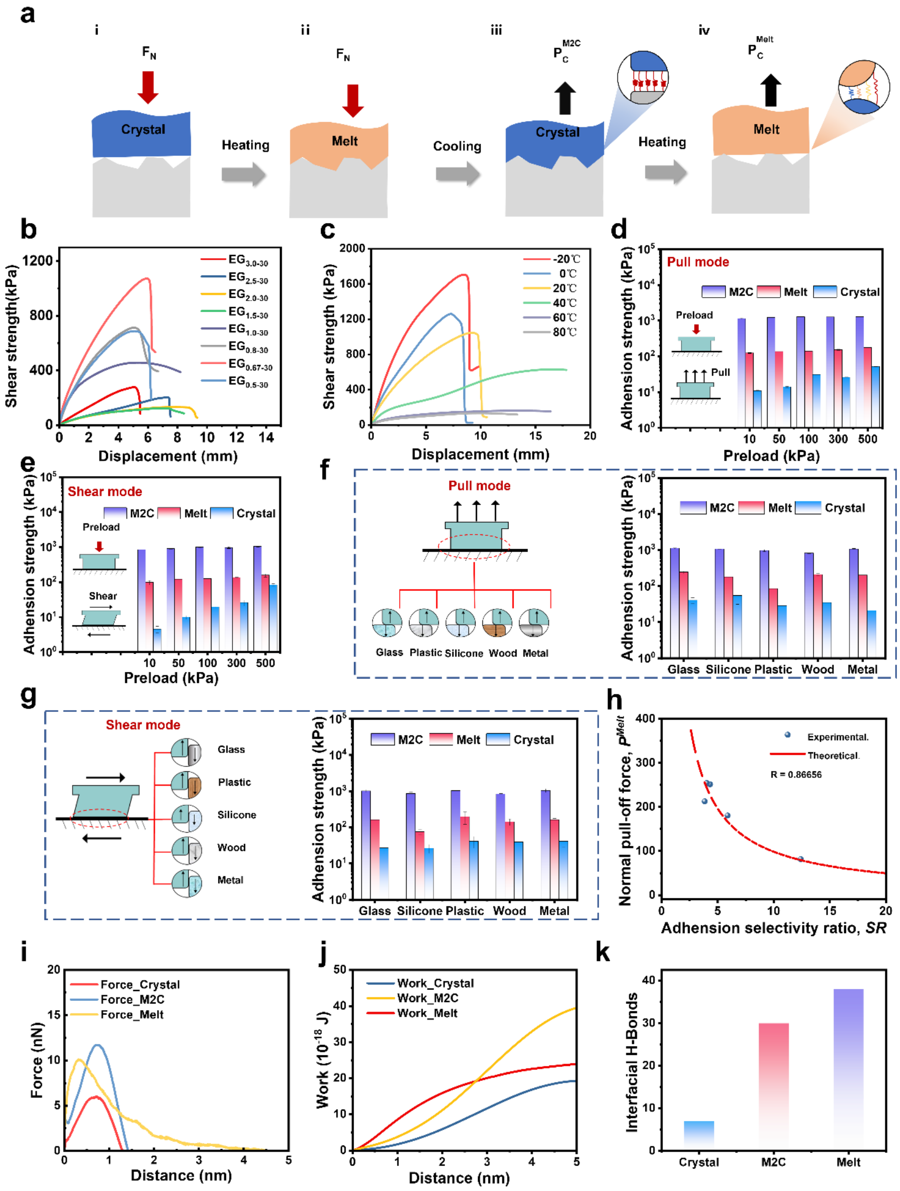

Fig. 3 Mechanism of crystallization-enhanced adhesion in eutectogels. (a) Schematic representation of eutectogel adhesion on rough surfaces in crystal (C), melt (M), and melt-crystal states(M2C). (b) Effect of different solvent ratios on the adhesion properties of eutectogels. (c) Effect of temperature on the adhesion properties of eutectogel $EG_{0.67\text{-}30}$ in shear adhesion test mode. (d-e) Influence of preload on adhesion strength under pull (top) and shear (bottom) modes (measured on smooth glass, aspect ratio = 1). The melt and melt-crystalline states reach adhesion equilibrium at a smaller pre-load pressure (100 kPa), while the crystalline state is difficult to equilibrate (Sample information: $EG_{0.67\text{-}30}$). (f-g) Adhesion strength on various substrates under pull (top) and shear (bottom) modes

(aspect ratio = 1 and preload = 200 kPa) of eutectogel $EG_{0.67-30}$. Adhesion characteristics, including high M2C adhesion, low melt adhesion, and minimal crystal adhesion, are consistent across substrates. (h) Molecular dynamics simulations of force conditions (i) and fracture work (j) during adhesion at crystallization, melting, and M2C states for eutectogels. The force required to release adhesion and fracture work during the M2C adhesion process is significantly greater than that required for the Melt and Crystal adhesion processes. (k) The number of interfacial hydrogen bonds formed between eutectogel $EG_{0.67-30}$ and SiO_2 substrate in crystalline, melting, and M2C states (calculated via molecular dynamics simulations).

In addition, as you suggested, we have moved the NMR spectra, variable-temperature IR spectra to Supplementary Information to improve the manuscript's flow and ensure that the reader remains focused on the core innovations. We believe these changes have strengthened the logical connection between our material-level findings and the final application performance.

Comment 5. A major flaw is the consistent omission of crucial experimental parameters. The exact sample compositions (U:C ratio, DES:monomer ratio) used for the key adhesion tests (Fig. 3b-f, h), molecular-level analyses (Fig. 4, 5), and application demonstrations are not specified. Without this information, the results are neither credible nor reproducible. For example, Fig. 2d-i lack complete compositional information, making the data difficult to interpret.

Response: We sincerely apologize for the omission of these critical experimental parameters in the original manuscript. We fully agree with the reviewer that precise compositional details are indispensable for the credibility and reproducibility of the study.

Following your guidance, we have conducted a thorough audit of the entire manuscript and its Supplementary Information to ensure all data are fully contextualized. To improve interpretability, we have introduced a standardized nomenclature for eutectogels (e.g., EG_{x-y}, where x represents the U:C ratio and y denotes the monomer content), and add the description throughout the text and figures (including Fig. 3, 4, 5) where necessary.

Our revisions to the manuscript are as follows:

In the revised manuscript, we have added a description of the synthesized information.

On page 7: To improve interpretability, we have introduced a standardized nomenclature for eutectogels (e.g., EG_{x-y}, where x represents the U:C ratio and y denotes the monomer content).

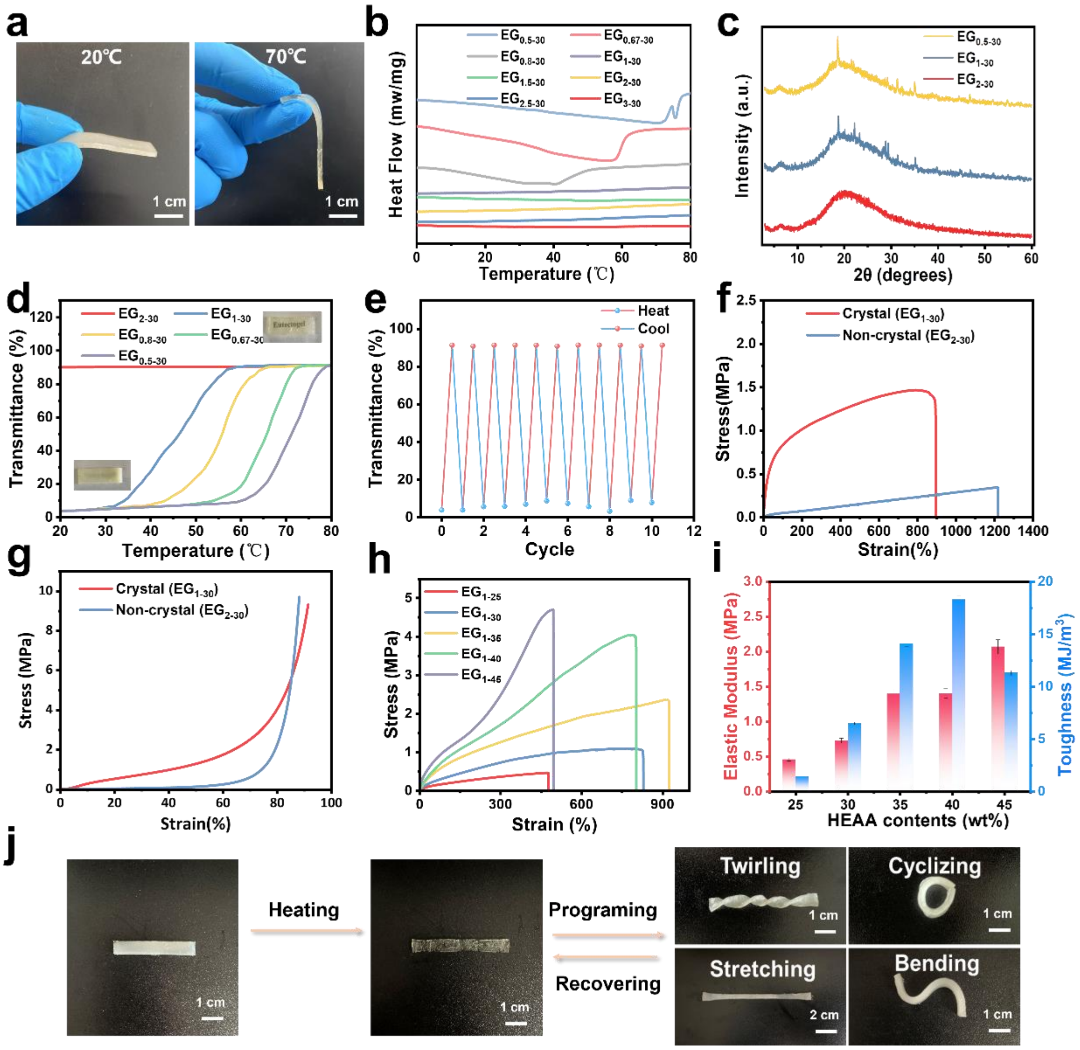

Fig. 2 Material characterization of eutectogels. (a) Digital photographs of eutectogel $EG_{1\text{-}30}$ at different temperatures, showing an opaque, crystalline, and rigid state at low temperature (20°C), and a transparent, amorphous and soft state at high temperature (70°C). (b) Relationship between melting point and DES composition. The melting point increases with higher choline chloride contents. (c) XRD patterns of crystalline versus amorphous eutectogels. (d) Changes in light transmittance of eutectogels under the influence of temperature at different molar ratios. (e) Changes in light transmittance of crystalline eutectogel $EG_{1\text{-}30}$ under temperature cycles of 20°C-80°C. (f) Comparison of tensile properties between crystalline and amorphous eutectogels. (g) Comparison of compression properties between crystalline and amorphous eutectogels. (h) Effect of monomer content on the tensile properties of eutectogels. (i) Modulus and toughness of eutectogels at different monomer contents. (j) Temperature-mediated shape memory properties of crystalline eutectogel $EG_{1\text{-}30}$.

We also added more technical details regarding the gripping and climbing robot experiments.

Table S3. Control parameters of eutectogel intelligent gripper for grasping target objects.

Gripper compose	Control parameters	
Synthesis information of eutectogel	$EG_{0.67\text{-}30}$	HEAA: 30 wt%<br>U/C = 1:1.5
Eutectogel size	25 mm × 25mm × 2 mm	
Heating system	Brand	XinKai electrical appliance PI heating film 001
	Size	50 mm×50 mm
	Operating voltage and current	Maximum power: 24 V, 16 W<br>Actual power: 20 V, 0.5 A
Cooling system	Brand	Ice Lord radiator<br>Silent semiconductor heat sink 008
	Size and weight	50 mm×50 mm<br>100 g
	Operating voltage and current	Voltage: 5 V<br>Current: 2 A/ 3 A
Heating time	15-20 s	
Cooling time	20-30 s	

Table S5. Parameters of two-axis geared drive motors used in the wall-climbing robot.

**Essential parameter**	
Product name	DC dual-axis reduction motor
Brand	Chiming motor
Gearbox size	46 mm by 32 mm by 22.5 mm
Axle size	5.5 mm by （14.5 +14.5 mm）
Operating voltage	12 V
Rotation speed	0.055 sec/360 degree(Adjustable)
Reduction ratio	340:1
Rated torque and torque capacity	7 kgf.cm<br>16 kgf.cm
Rated current and Full load current	0.4 A<br>1.4 A
Gear type	metal

Table S6. Control parameters of wall climbing robot.

Climbing robot compose	Control parameters	
Synthesis information of eutectogel	$EG_{0.67-30}$	HEAA: 30 wt%<br>U/C = 1:1.5
Eutectogel size	50 mm × 25 mm × 2 mm	
Heating system	Product name	Brand: Bo Xiang<br>Nichrome heating wire
	Size (diameter)	0.6mm
	Operating voltage and current	Foot A: 6 V, 2 A<br>Foot B: 12 V, 4 A
Drive motor	Brand	Chiming motor
	Size	Table S5
	Operating voltage and current	Voltage: 5 V<br>Current: 2 A/ 3 A
Heating time	15-20 s	
Cooling time	20-30 s	

Comment 6. Page 4: The sentence "Except for the case of U:C = 2:1, the melting points... are below ambient temperature... such that the mixture remains solid..." is a direct logical contradiction.

Response: We have revised the corresponding sentence:

On page 5: Except for the case of U:C = 2:1, the eutectic solvents are crystal and solid at room temperature with melting point larger than 20°C.

Comment 7. Fig. 2f and 2g: These plots appear identical despite being labeled as tensile and compression tests, respectively.

Response: We are sorry for the mistake. We have already corrected the errors in Figures 2f and 2g.

Our revisions to the manuscript are as follows:

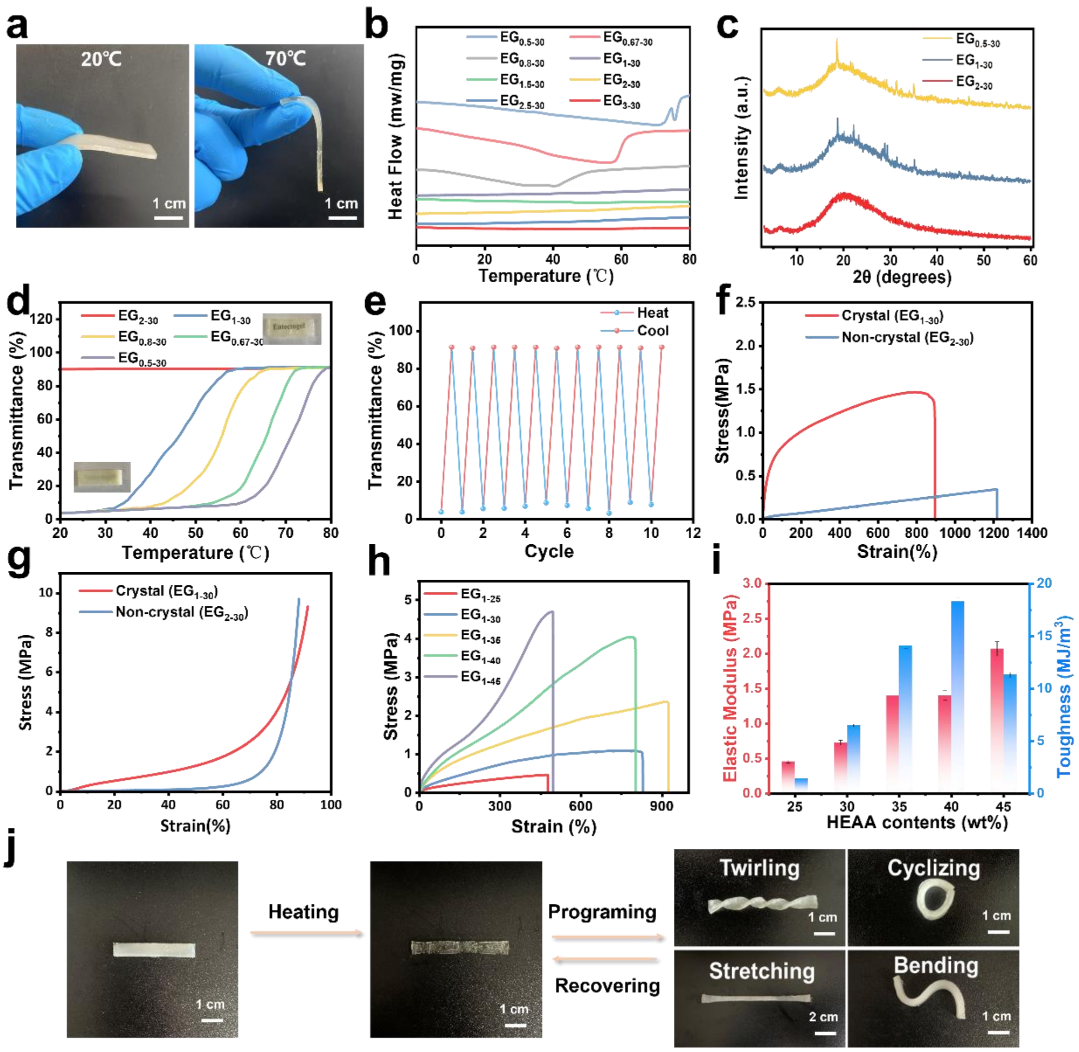

Fig. 2 Material characterization of eutectogels. (a) Digital photographs of eutectogel EG_{1-30} at different temperatures, showing an opaque, crystalline, and rigid state at low temperature (20°C), and a transparent, amorphous and soft state at high temperature (70°C). (b) Relationship between melting point and DES composition.

The melting point increases with higher choline chloride contents. (c) XRD patterns of crystalline versus amorphous eutectogels. (d) Changes in light transmittance of eutectogels under the influence of temperature at different molar ratios. (e) Changes in light transmittance of crystalline eutectogel $EG_{1\text{-}30}$ under temperature cycles of 20°C-80°C. (f) Comparison of tensile properties between crystalline and amorphous eutectogels. (g) Comparison of compression properties between crystalline and amorphous eutectogels. (h) Effect of monomer content on the tensile properties of eutectogels. (i) Modulus and toughness of eutectogels at different monomer contents. (j) Temperature-mediated shape memory properties of crystalline eutectogel $EG_{1\text{-}30}$.

Comment 8. Fig. S16/S17: The blue curve is labeled "20°C Non-crystal eutectogel," which should likely be "20°C Crystal eutectogel." There are also inconsistencies between the figure legend (80°C) and the graph legend (70°C).

Response: Sorry for the mistakes. We have corrected the temperature labeling errors in Figures S16 and S17 which are now Fig. S18a and Fig. S18b, respectively.

Our revisions to the manuscript are as follows:

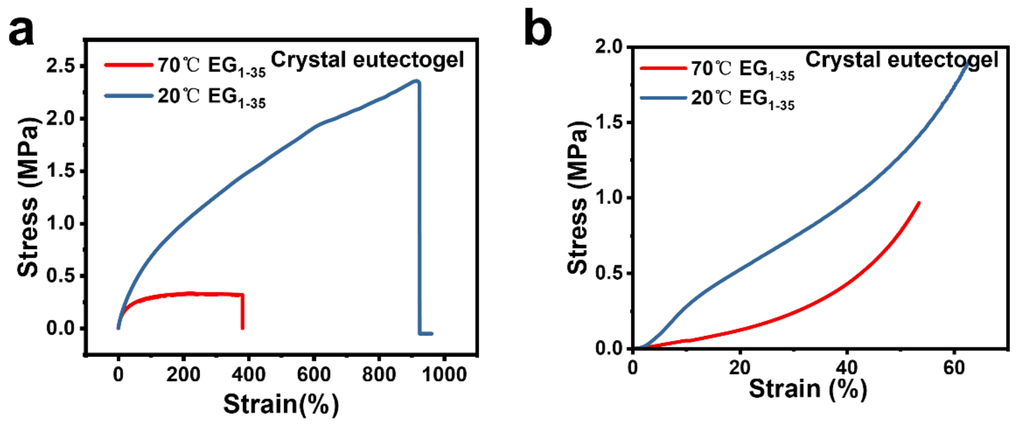

Fig. S18. Tensile (a) and compressive (b) tests of eutectogel $EG_{1\text{-}35}$ at high (70 °C) and room temperatures (20°C).

Comment 9. The presentation of adhesion data primarily as bar graphs is insufficient. Raw force-displacement curves or force-time curves for all adhesion tests are necessary for a rigorous assessment. The number of repeats and the nature of the error bars are not specified.

Response: Thank you for your constructive comment. We have provided the complete force-displacement curves in the Supplementary Information. Each data point in the bar charts shown in Figures 3b-e represents the mean value from three replicate tests, with the error bars indicating the standard deviation across the three sets of data.

Our revisions to the manuscript are as follows.

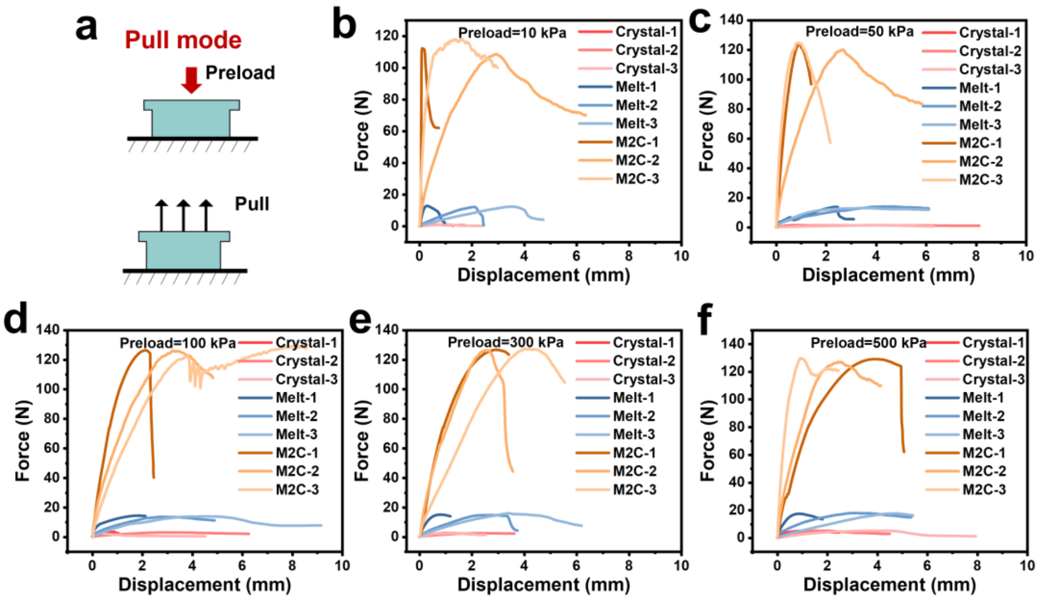

Fig. S26. Force-displacement curves of eutectogel adhesion on glass substrates under 10–500 kPa preload in pull mode. (a) Schematic diagram of the adhesion process in pull mode. (b-f) Force-displacement curve at 10–500 kPa (measured on smooth glass, aspect ratio = 1) (sample information: $EG_{0.67\text{-}30}$).

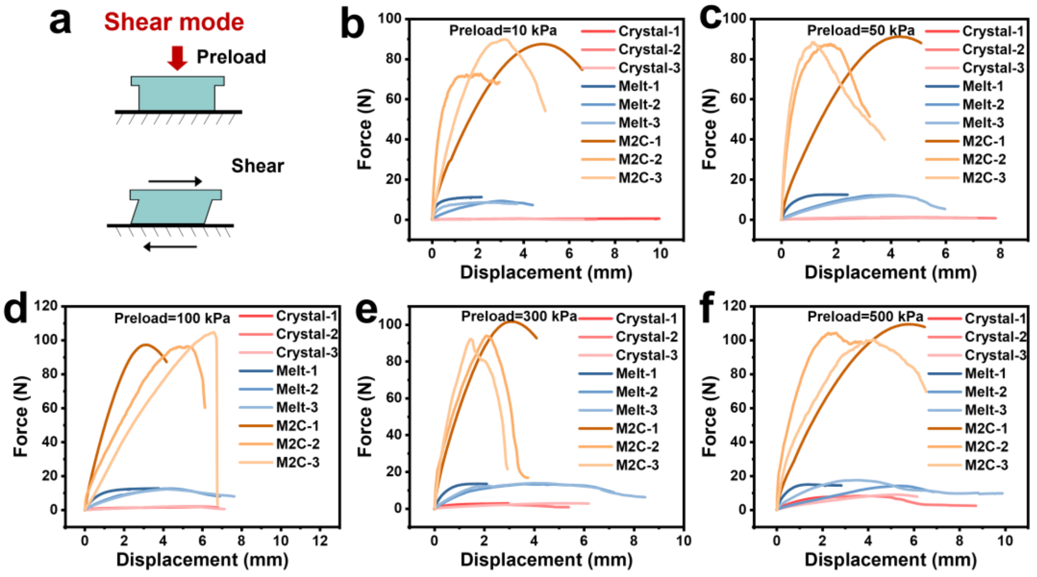

Fig. S27. Force-displacement curves of eutectogel $EG_{0.67\text{-}30}$ adhesion on glass substrates under 10–500 kPa preload in shear mode. (a) Schematic diagram of the adhesion process in shear mode. (b-f) Force-displacement curve at 10–500 kPa (measured on smooth glass, aspect ratio = 1).

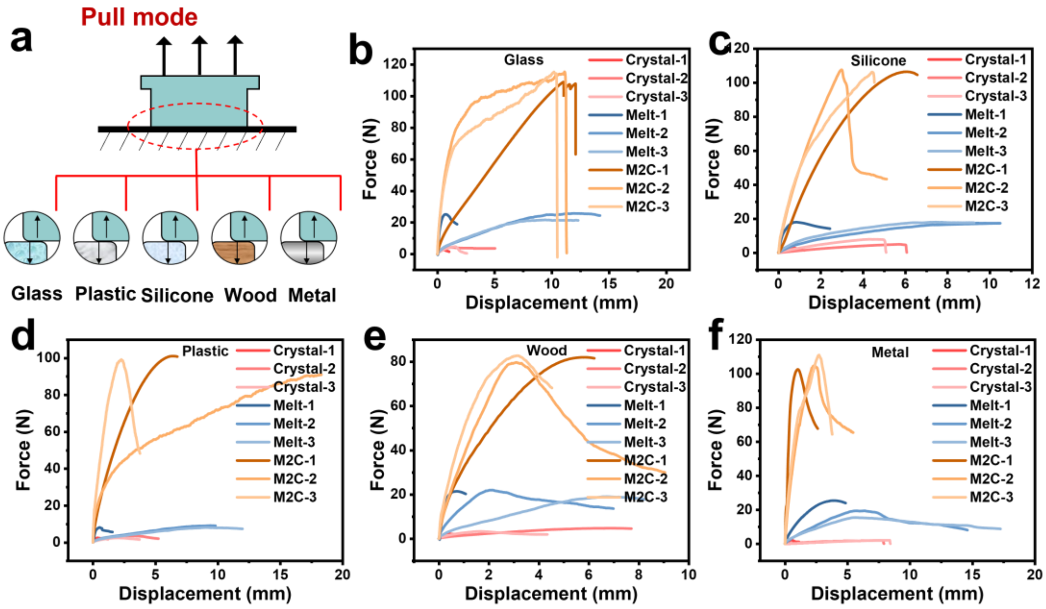

Fig. S28. Force-displacement curves of adhesion of eutectogel $EG_{0.67\text{-}30}$ on different material surfaces during pull mode. (a) Schematic diagram of the adhesion process in pull mode. (b-f) Force-displacement curve at different surface (aspect ratio = 1 and preload = 200 kPa).

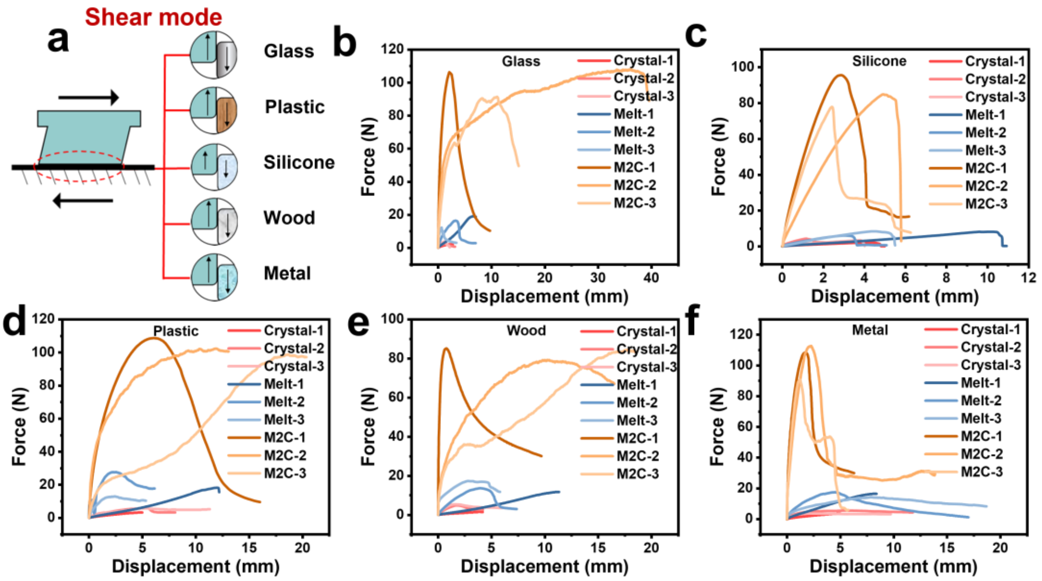

Fig. S29. Force-displacement curves of adhesion of eutectogel $EG_{0.67\text{-}30}$on different material surfaces during shear mode. (a) Schematic diagram of the adhesion process in shear mode. (b-f) Force-displacement curve at different surface (aspect ratio = 1 and preload = 200 kPa).

Comment 10. The claim on page 14 regarding "stronger gel cohesion" requires direct evidence from tensile tests (stress-strain curves) for the corresponding U:C ratios, which are not provided.

Response: Thank you for your suggestion. We have provided stress-strain curves for different molar ratios in the Supplementary Information. The figures clearly show that the modulus and

yield stress of the gel are significantly higher at U/C=1:2 compared to U/C=1:1.5. Therefore, the stronger cohesive force within the gel is demonstrated.

Our revisions to the manuscript are as follows.

On page 9: Furthermore, we systematically investigated the influence of the urea-to-choline chloride ratio on the mechanical properties. The elastic modulus is larger for samples with decreasing concentration of urea ($EG_{0.8-30}$>EG_{2-30}), while decreases for sample $EG_{0.67-30}$ and then increases for sample $EG_{0.5-30}$. This complicated trend is probably due to complication of crystallization, hydrogen bonding between DES-DES, polymer chains-polymer chains and DES-polymer chains (**Supplementary Fig. S17**).

On page 13: Changes in eutectogel cohesion due to solvent composition variations are characterized through mechanical testing. At a U/C molar ratio of 1:1.5, the eutectogel ($EG_{0.67-30}$) exhibited significantly lower modulus compared to gels with $EG_{0.5-30}$ and $EG_{0.8-30}$ (**Supplementary Fig. S17**).

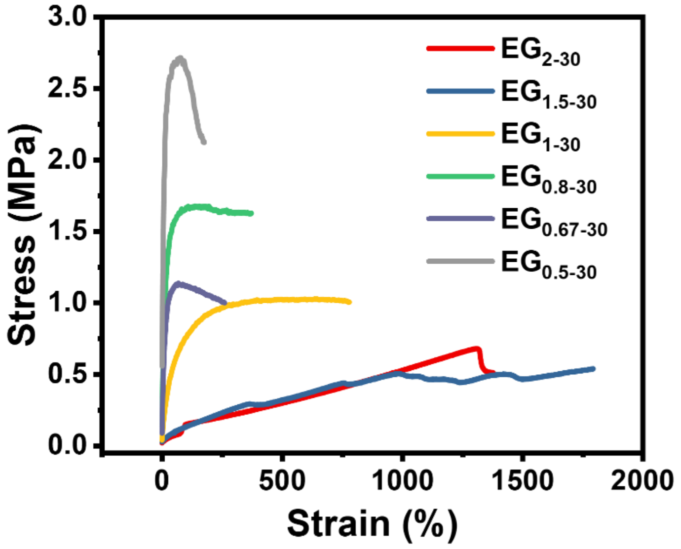

Fig. S17. Effect of solvent composition changes on the tensile curves of eutectogels (sample information: EG_{x-30}).

Comment 11. The absence of Dynamic Mechanical Analysis (DMA) is a notable omission for a study centered on temperature-dependent mechanical transitions.

Response: Thank you for your suggestion. We have added DMA data for three different molar ratios in the revised Supplementary Information. Dynamic mechanical analysis is further employed to investigate change of mechanical properties at different temperatures. The non-crystal eutectogel with (EG_{2-30}) only shows slight decrease in storage modulus from 180 kPa (30°C) to 100 kPa (95°C) upon heating (**Supplementary Fig. S19**). In contrast, the crystalline eutectogels show sharp decrease in the mechanical properties: the storge modulus of crystalline eutectogel ($EG_{0.67-30}$) decreases from 3500 kPa (30°C) to 150 kPa (70°C), and the storge modulus of crystalline eutectogel ($EG_{0.5-30}$) decreases from 5000 kPa (30°C) to 500 kPa (70°C)

(**Supplementary Fig. S20**). Due to the softening of the gel at high temperature and difficulty in DMA measurements, we also performed rheological measurements at wider temperature range.

Our revisions to the manuscript are as follows:

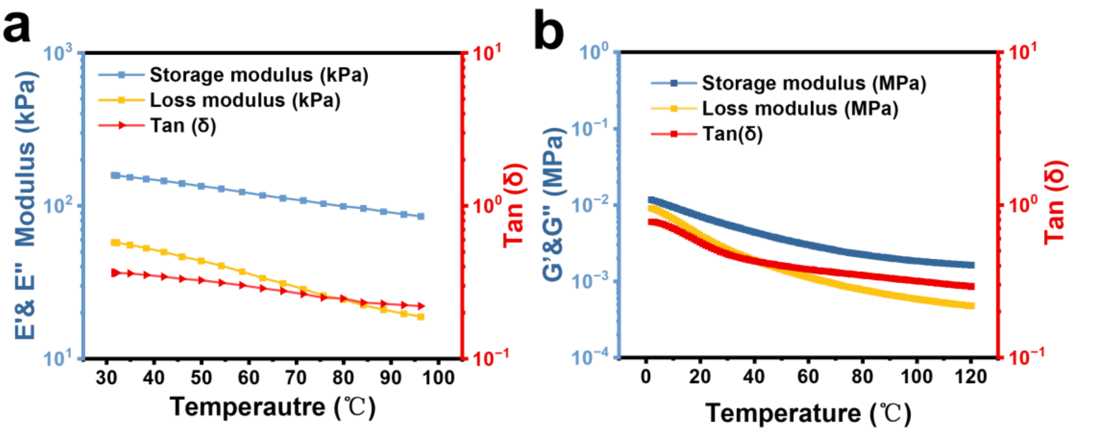

Fig.S19. Dynamic mechanical analysis (DMA) of amorphous eutectogel $EG_{2\text{-}30}$.

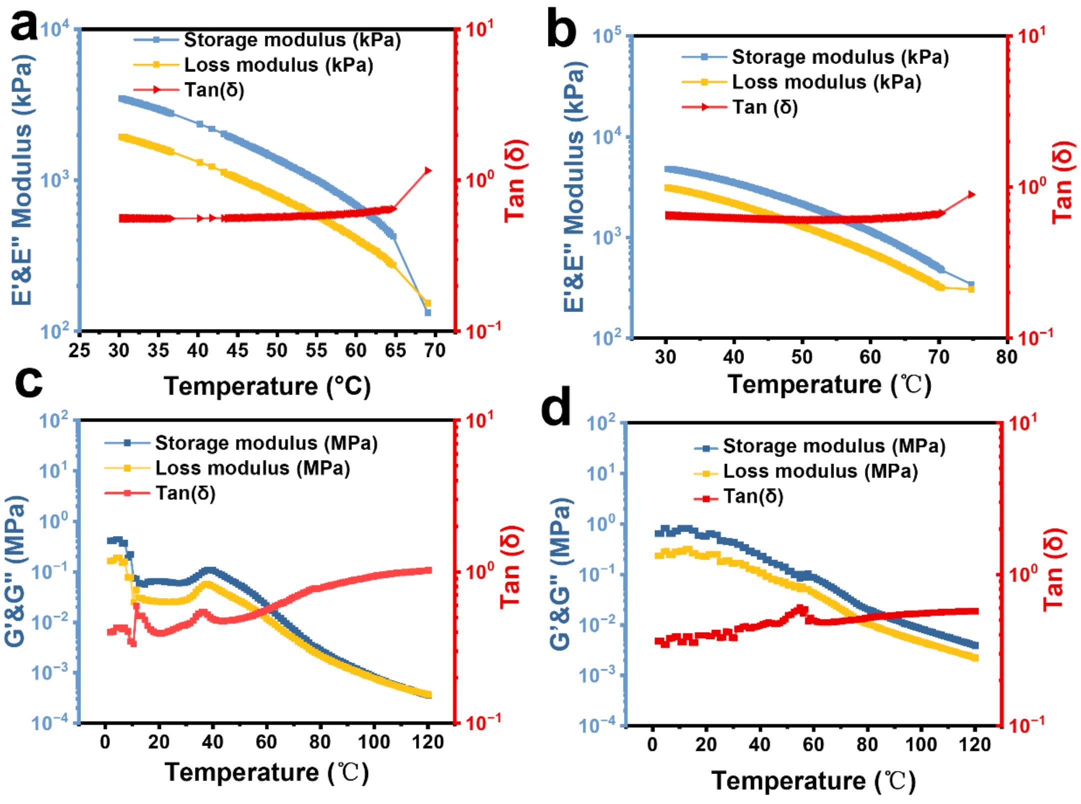

Fig.S20. (a) Dynamic mechanical analysis of eutectogel $EG_{0.67\text{-}30}$. (b) Dynamic mechanical analysis of eutectogel $EG_{0.5\text{-}30}$. (c) Rheological measurement of the eutectogel $EG_{0.67\text{-}30}$, showing a peak in tan δ at 38°C. (d) Rheological measurement of the eutectogel $EG_{0.5\text{-}30}$,

showing a peak in tan δ at 58°C.

On page 11 of the main text: Dynamic mechanical analysis is further employed to investigate change of mechanical properties at different temperatures. The non-crystal eutectogel with ($EG_{2\text{-}30}$) only shows slight decrease in storage modulus from 180 kPa (30°C) to 100 kPa (95°C) upon heating (**Supplementary Fig. S19**). In contrast, the crystalline eutectogels show sharp decrease in the mechanical properties: the storge modulus of crystalline eutectogel ($EG_{0.67\text{-}30}$) decreases from 3500 kPa (30°C) to 150 kPa (70°C), and the storge modulus of crystalline eutectogel ($EG_{0.5\text{-}30}$) decreases from 5000 kPa (30°C) to 500 kPa (70°C) (**Supplementary Fig. S20**). Rheological measurements also confirmed the softening of crystalline eutectogels at high temperature.

Comment 12. The theoretical model (Eq. 1, 2) is presented without adequate explanation of its terms or the underlying physics (e.g., the roles of h_{rms} and shape locking), rendering it incomprehensible to non-specialist readers.

Response: Thank you for your suggestion. We have added the relevant introduction to the manuscript.

Our revisions to the manuscript are as follows:

On page 15: Here we consider two types of surfaces, rough and smooth, whose areas are denoted as s and s_0, respectively. The pull-off forces of the M2C and molten states on a reference smooth surface are defined as P_0^{M2C} and P_0^{Melt}, while those on a rough surface are defined as P_c^{M2C} and P_c^{Melt}. For the rough surface, we introduce the roughness h_{rms} and its slope h'_{rms}; for the reference surface, these quantities are defined as h_{rms}^0 and h'^0_{rms}. The adhesion selectivity SR is defined as the ratio of the pull-off forces between the M2C and Melt states on a rough surface, which reflects the competitive relationship between the adhesive and deadhesive states. Previous studies have shown that SR is inversely proportional to h'_{rms}, as described in Eq. (1) [33]. We further find that P_c^{Melt} is inversely proportional to SR, as indicated in Eq. (2). The theoretical predictions and experimental results are presented in Fig. 3f, and the adhesion values of eutectogels in both the molten and M2C states are in good agreement with the theoretical models **(Eqs. 1 and 2, Fig. 3h)**.

With the above revision, hopefully we can resolve your questions. Thank you very much again for your insightful comments in improving our manuscript.

Reviewer #2 (Remarks to the Author):

Comment: The authors have conducted a systematic work on an interesting reversible adhesive. By utilizing the controllable crystallization of the eutectogels, the authors achieved programmable attachment/detachment of adhesives on various substrates, particularly rough surfaces. Comparing the previous works on reversible adhesives, the adhesion strength of these eutectogels is much higher, while the surface adaptability is better. Real-time monitoring of adhesion strength is very important for exploring adhesive-enabled devices. Both spectroscopy analyses and theoretical calculations were conducted to get insights into the reversible adhesion mechanism. Also, the authors verified the potential applications of these smart adhesives in grasping and climbing robots. Overall, this paper is acceptable for publication in Nature Communications after addressing the following concerns.

Response: We sincerely thank the reviewer for their positive assessment and for recognizing the significance of our work on reversible adhesives. We are encouraged by the feedback regarding programmable adhesion and the mechanical insights with spectroscopic and theoretical analyses. We have carefully addressed all the comments and suggestions, which we believe have further strengthened the manuscript.

Comment 1. In the melted state, the eutectogel still has an adhesion strength of ~100 kPa. This is a relatively large value. Is it possible to further reduce the adhesion strength by alternating the composition of the polymer networks while maintaining the crystallization-melting phase transition?

Response: We appreciate the reviewer's perspective on the adhesion values. The ~100 kPa strength in the melted state is a result of the high density of hydrogen bonding sites provided by the solvent. While adjusting the polymer network can influence the bulk elasticity, the interfacial adhesion is largely determined by the solvent-substrate interactions. Consequently, achieving extremely low adhesion solely through polymer network composition adjustments is challenging. We have updated the manuscript to reflect the influence of composition on these adhesion limits and discussed the potential for future optimization of the polymer scaffold.

We add corresponding statement:

On page 13: Due to the inherent hydrogen bonding provided by the DES at the interface, the adhesion force maintains a baseline of ~100 kPa. This suggests that the chemical nature of the solvent is the governing factor for adhesion in the melted state, limiting the impact of polymer network modifications.

Comment 2. In Figure S16, the red line represents: 70°C Crystal eutectogel, while the figure caption stated that "Tensile tests of crystalline eutectogels at high (80 °C) and room temperatures (20°C).". Figure S17 has the same mistake.

Response: We sincerely thank the reviewer for their careful reading and for catching this inconsistency. We apologize for the oversight. We have corrected the temperature labels in **Fig. S16** and **S17** to 70°C to ensure consistency with the experimental parameters and the figure captions.

Our revisions to the manuscript are as follows:

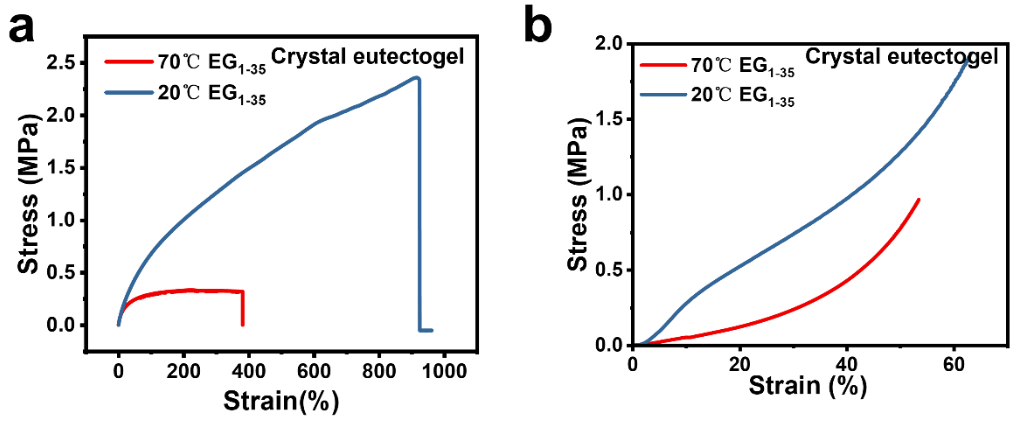

Fig. S18. Tensile (a) and compressive (b) tests of eutectogel $EG_{1\text{-}35}$ at high (70 °C) and room temperatures (20°C).

Comment 3. The size of the phase-change gel layer applied in the climbing robot should be clearly described in the experimental section.

Response: Thank you for your suggestion. The phase-change gel used in the legs of the three-legged robot has a size of 50 mm × 25 mm × 2 mm. Relevant annotations have been added to the text.

Our revisions to the manuscript are as follows:

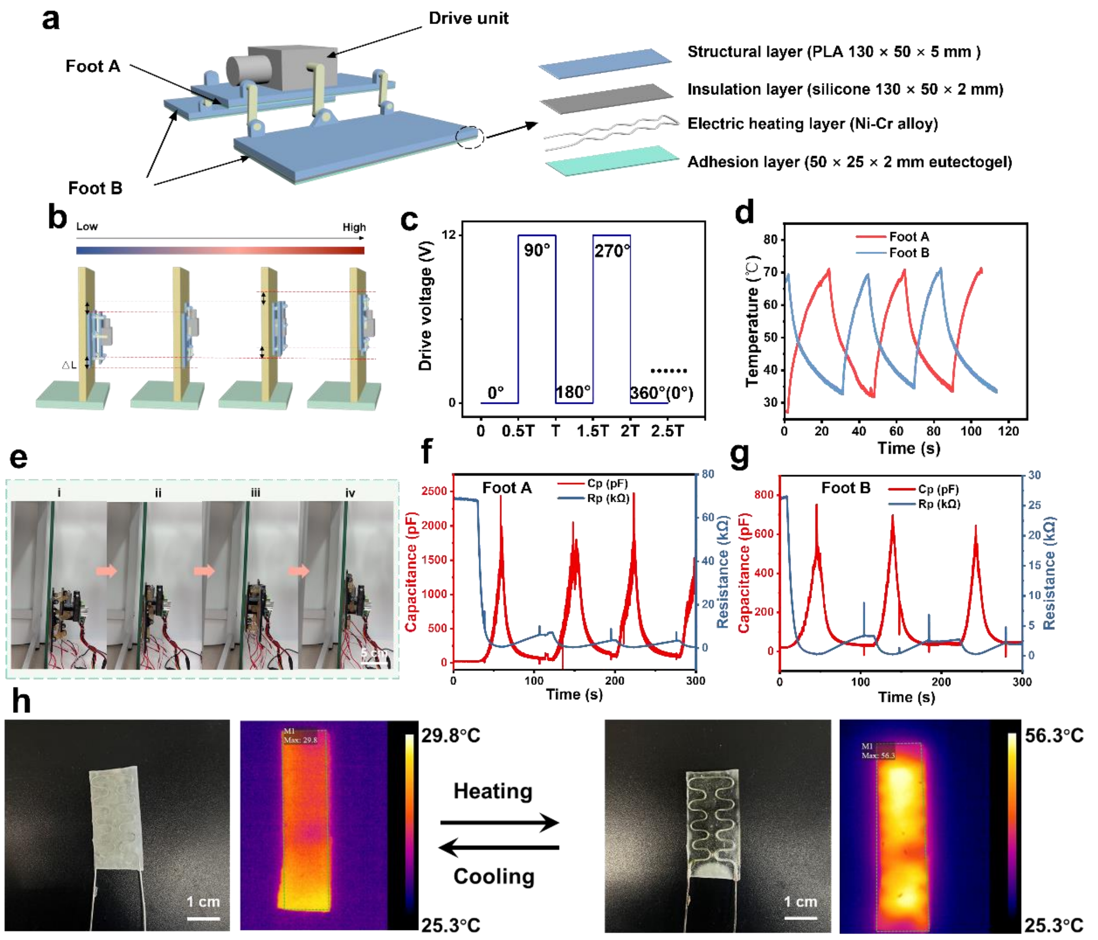

Fig. 6 Design principle and fabrication of the wall-climbing robot. (a) Illustration of the mechanical design of the three-feet wall-climbing robot (left), and layout of the foot including a supporting layer (size: 130 mm × 50 mm × 5 mm), an insulation layer (size: 50 mm × 25 mm × 2 mm), a metallic layer, and the eutectogel ($EG_{0.67\text{-}30}$) layer (size: 50 mm × 25 mm × 2 mm). (b) Schematic diagram of the climbing principle of the wall-climbing robot. The robot climbing motion is realized by human-coordinated control. Over each cycle, the soft robot climbs up by a distance of ΔL. (c) The drive motor rotates the shaft at 12V. Controlling on-off of the drive circuit realizes the intermittent rotation of the dual axes of the gear motor between 0-360°. (d) Temperature curve of eutectogel heating and cooling in robotic feet during three-legged robot crawling. (e) Images of a wall-climbing robot climbing on a 90° glass wall. (f) Electrical signal changes during the heating adhesion-cooling crystallization-heating peeling process of the middle foot (Foot A) of the wall-climbing robot. (g) Electrical signal changes during the heating adhesion-cooling crystallization-heating peeling process of the side foot (Foot B) of a wall-climbing robot. (h) Infrared images probing the heating process of eutectogel with a conductive circuit embedded in the gel ($EG_{0.67\text{-}30}$).

Comment 4. On page 3, it was claimed that “the adhesion strength can be tuned from 200 kPa to 1000 kPa”, while on page 5, it was claimed that “Specifically, the adhesion strength increases from ~100 kPa in the melted state to ~1 MPa in the melt-to-crystalline (M2C) state”. I suggest the authors give a consistent description.

Response:

We thank the reviewer for pointing out these inconsistencies. We have standardized the adhesion values throughout the manuscript to ensure clarity. Specifically, we have revised the text to consistently reflect an adhesion range of 10^2 kPa in the melted state to 10^3 kPa in the M2C state, highlighting the tenfold increase in adhesion strength triggered by the phase transition.

Corresponding revision:

On page 4: Upon electrothermal activation, the gel rapidly transitions from a crystalline state (10^1 kPa) to a molten state (10^2 kPa). Through the M2C cooling process, its adhesion properties are tuned between 10^2 kPa and 10^3 kPa, sufficient to support robotic climbing on vertical surfaces with varied materials and roughness.

On page 5: This phase transition induces a marked change in adhesion forces, analogous to that of ice. Specifically, the adhesion strength increases from ~100 kPa in the melted state to ~1 MPa in the melt-to-crystalline (M2C) state (**Fig. 1c**).

Comment 5. Please identify the samples used in Figure S11a and b.

Response: Thank you for your suggestion. To improve interpretability, we now have introduced a standardized nomenclature for eutectogels (e.g., $EG_{x\text{-}y}$, where x represents the U:C ratio and y denotes the monomer content), and add the description throughout the text and figures where necessary.

Our revisions to the manuscript are as follows:

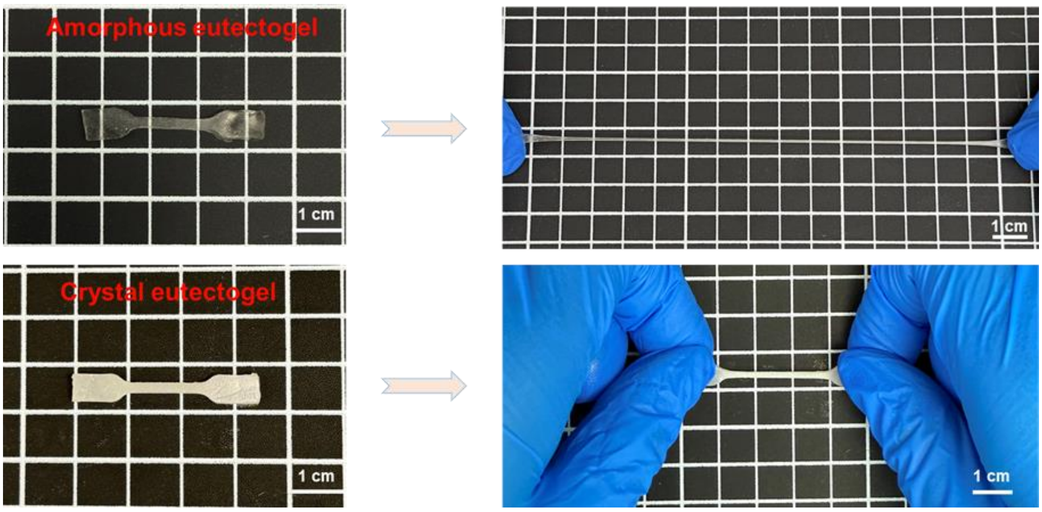

Fig. S11. Crystalline eutectogel $EG_{1\text{-}30}$ and amorphous eutectogel $EG_{2\text{-}30}$ while stretching.

Comment 6. In the caption of Figure 2, it was indicated that (f) Comparison of compression properties between crystalline and amorphous eutectogels, (g) Comparison of tensile properties between crystalline and amorphous eutectogels. However, Figures 2f and 2g are the same data. Please double-check the figures.

Response: We are very sorry for the mistake. We have already corrected the errors in Figures 2f and 2g. Our revisions to the manuscript are as follows:

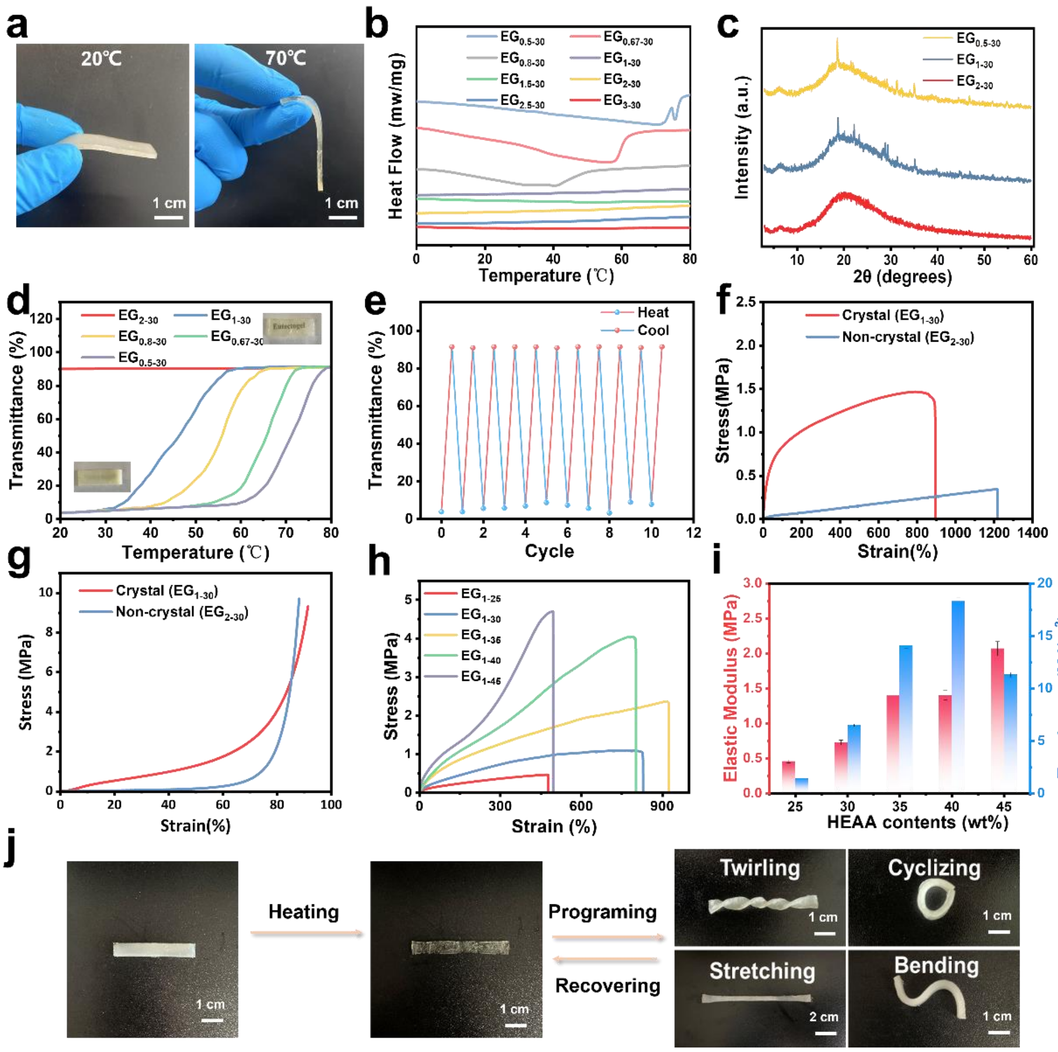

Fig. 2 Material characterization of eutectogels. (a) Digital photographs of eutectogel EG_{1-30} at different temperatures, showing an opaque, crystalline, and rigid state at low temperature (20°C), and a transparent, amorphous and soft state at high temperature (70°C). (b) Relationship between melting point and DES composition. The melting point increases with higher choline chloride contents. (c) XRD patterns of crystalline versus amorphous eutectogels. (d) Changes in light transmittance of eutectogels under the influence of temperature at different molar ratios. (e) Changes in light transmittance of crystalline eutectogel EG_{1-30} under temperature cycles of 20°C-80°C. (f) Comparison of tensile properties between crystalline and amorphous eutectogels. (g) Comparison of compression properties between crystalline and amorphous eutectogels. (h) Effect of monomer content on the tensile properties of eutectogels. (i) Modulus and toughness of eutectogels at different monomer contents. (j) Temperature-mediated shape memory properties of crystalline eutectogel EG_{1-30}.

Comment 7. Cyclic tensile tests indicate that the eutectogels undergo considerable mechanical strength reduction and permanent deformation during repeated stretching. This may not be beneficial for the practical application of the eutectogels in grasping and climbing

robots. I suggest the authors give a detailed description of their strategies for addressing these problems.

Response: Thank you for your valuable suggestions. During the overall adhesion performance process, eutectogel in its crystalline state exhibits minimal deformation when subjected to tensile forces. We have supplemented the compression cycle data for the eutectogel in its crystalline state under a 50 N external force, as shown in the figure below. It can be observed that a eutectogel sample with a height of 12 mm and a surface area of 2 mm^2 exhibits a strain of only about 8% after 10 compression cycles under a large external force of 50 N. Therefore, this has virtually negligible impact on subsequent applications.

Our revisions to the manuscript are as follows:

On page 9: The material's structural integrity is evidenced by a cyclic compression test. Under a constant load of 50 N over ten cycles, the gel exhibited a mere 8% strain, confirming that crystallization substantially reinforces its cohesive strength (**Supplementary Fig. S16**).

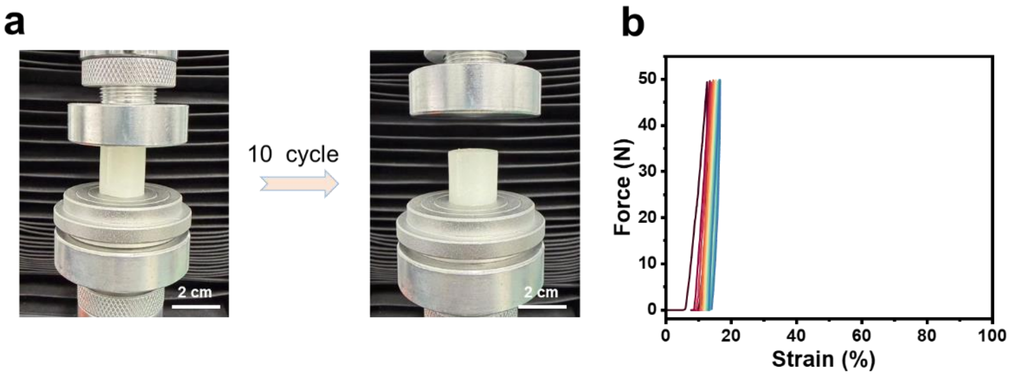

Fig. S16. (a) Comparison before and after ten compression cycles of sample $EG_{1\text{-}30}$ under a constant stress of 50 N/25 MPa. (b) Strain change of sample $EG_{1\text{-}30}$ after ten compression cycles at a constant stress of 50 N/25 MPa (strain = 8%).

Comment 8. The authors are suggested to clearly list the control parameters for the grasping and robotic climbing experiments.

Response: Thank you for your valuable suggestions. We agree that providing control parameters for the gripper and wall climbing robot would be very meaningful for the manuscript. Therefore, we have provided corresponding control parameters in the Supplementary Information as Table S3, Table S5 and Table S6.

Our revisions to the manuscript are as follows:

Table S3. Control parameters of eutectogel intelligent gripper for grasping target objects.

Gripper compose	Control parameters	
Synthesis information of eutectogel	$EG_{0.67\text{-}30}$	HEAA: 30 wt%<br>U/C = 1:1.5
Eutectogel size	25 mm × 25mm × 2 mm	
Heating system	Brand	XinKai electrical appliance PI heating film 001
	Size	50 mm×50 mm
	Operating voltage and current	Maximum power: 24 V, 16 W<br>Actual power: 20 V, 0.5 A
Cooling system	Brand	Ice Lord radiator<br>Silent semiconductor heat sink 008
	Size and weight	50 mm×50 mm<br>100 g
	Operating voltage and current	Voltage: 5 V<br>Current: 2 A/ 3 A
Heating time	15-20 s	
Cooling time	20-30 s	

Table S5. Parameters of two-axis geared drive motors used in the wall-climbing robot.

Essential parameter	
Product name	DC dual-axis reduction motor
Brand	Chiming motor
Gearbox size	46 mm by 32 mm by 22.5 mm
Axle size	5.5 mm by （14.5 +14.5 mm）
Operating voltage	12 V
Rotation speed	0.055 sec/360 degree(Adjustable)
Reduction ratio	340:1
Rated torque and torque capacity	7 kgf.cm<br>16 kgf.cm
Rated current and Full load current	0.4 A<br>1.4 A
Gear type	metal

Table S6. Control parameters of wall climbing robot.

Climbing robot compose	Control parameters	
Synthesis information of eutectogel	$EG_{0.67-30}$	HEAA: 30 wt%<br>U/C = 1:1.5
Eutectogel size	50 mm × 25 mm × 2 mm	
Heating system	Product name	Brand: Bo Xiang<br>Nichrome heating wire
	Size (diameter)	0.6mm
	Operating voltage and current	Foot A: 6 V, 2 A<br>Foot B: 12 V, 4 A
Drive motor	Brand	Chiming motor
	Size	Table S5
	Operating voltage and current	Voltage: 5 V<br>Current: 2 A/ 3 A
Heating time	15-20 s	
Cooling time	20-30 s	

Comment 9. Since the heating and cooling process of the adhesives can also be affected by the environmental temperatures, I suggest the authors clearly identify the temperature in grasping and robotic climbing experiments.

Response: Thank you for your valuable suggestions. We conducted the experiments in ambient environment where the surrounding temperature is in the range of 15°C-25°C. We have provided the temperature change curve of the gripper during the gripping process and the temperature change curve of the phase-change gel during the wall-climbing robot's movement.

Our revisions to the manuscript are as follows.

On page 25: To better assess the state changes of the eutectogel gripper during the gripping process, temperature sensors monitored the heating and cooling cycles on the gel's surface. As shown in **Fig. 5f**, when the surface temperature reached 70°C, the eutectogel entered a fully molten state due to thermal transfer efficiency. Similarly, when the temperature dropped to 20°C, the gel transitioned into a crystalline state. The entire switching process is maintained at approximately 100 seconds **(Fig. 5f)**.

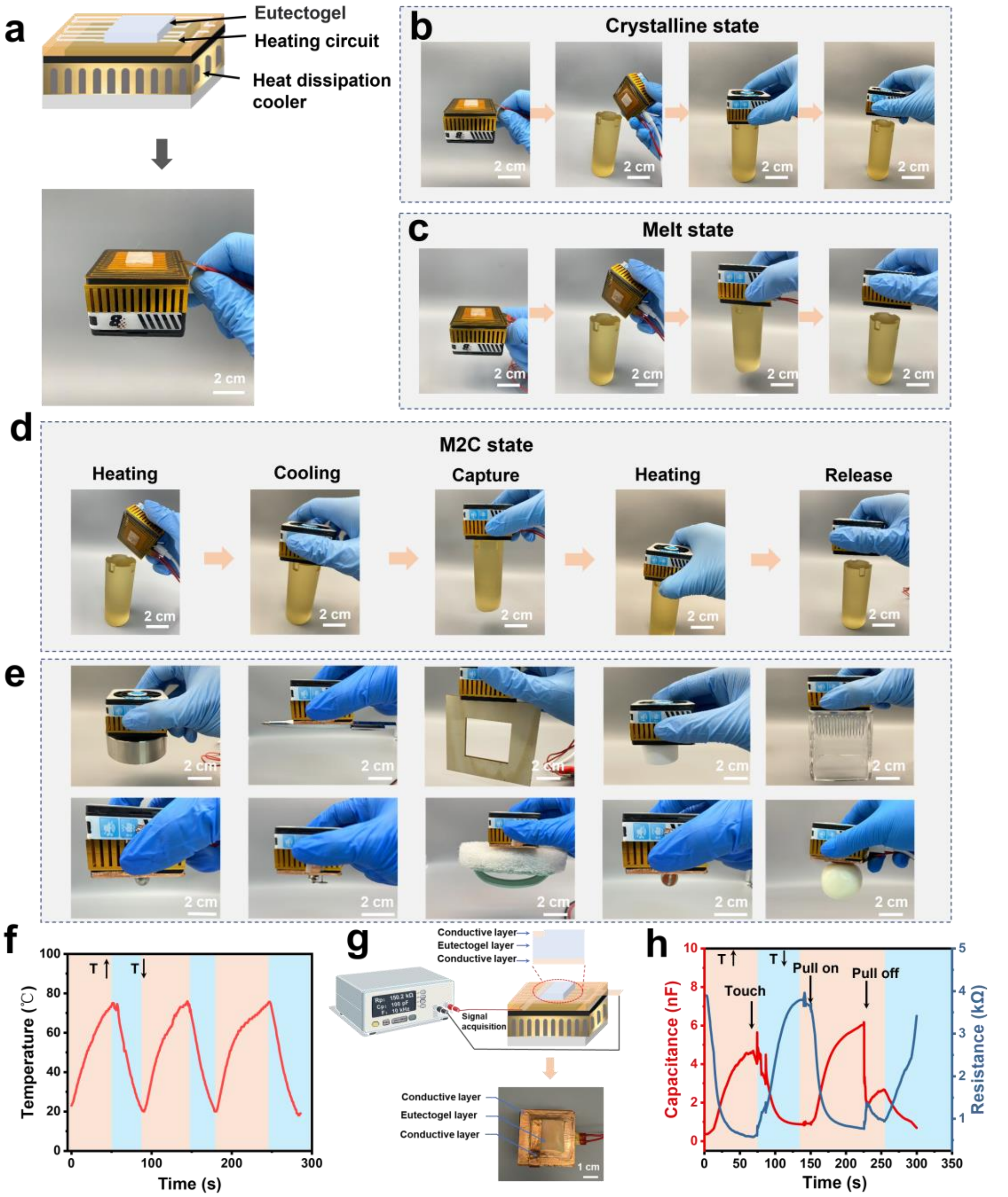

Fig. 5 Development of smart adhesive grippers based on crystalline eutectogels. (a) Schematic diagram of an intelligent adhesive gripper structure based on eutectogel. (b) In the crystalline state, eutectogel smart grippers have difficulty grasping target objects through adhesion. (c) The eutectogel smart gripper in the melt state tends to lose its adhesion to the target object during lifting. (d) Digital photos of the process of picking up target items using eutectogel smart gripper based on the M2C process. (e) The eutectogel ($EG_{0.67\text{-}30}$) smart gripper grasps target objects with diverse surfaces made from various materials, including metal block and sphere, PTFE hollow cylinder, wooden frame, prismatic metal pen, glass cup and glass sphere, metal thumbtack, porous sponge and egg. (f) Temperature curve of eutectogel ($EG_{0.67\text{-}}$

30) during heating and cooling in the operation of the eutectogel smart gripper. (g) Underlying mechanism diagram of electrical signals monitored by a digital bridge during the operation of a eutectogel ($EG_{0.67-30}$) holder. (h) Electrical signal changes during the process of the eutectogel ($EG_{0.67-30}$) smart gripper grasping target objects.

On page 29: As shown in **Fig. 6e** and **6f**, when the middle foot (Foot A) lifts, the eutectogel heats up, causing its resistance to decrease and capacitance to increase. Upon re-contact with the wall surface, the capacitance signal exhibits a marked increase. As the temperature drops, capacitance decreases and resistance increases. When the middle foot detaches from the wall, electrical signal fluctuations occur, characterized by decreased capacitance and increased resistance. This motion is synchronized with the heating and cooling cycles of the electrothermal gel layer (**Fig. 6e, Movie S8**)."

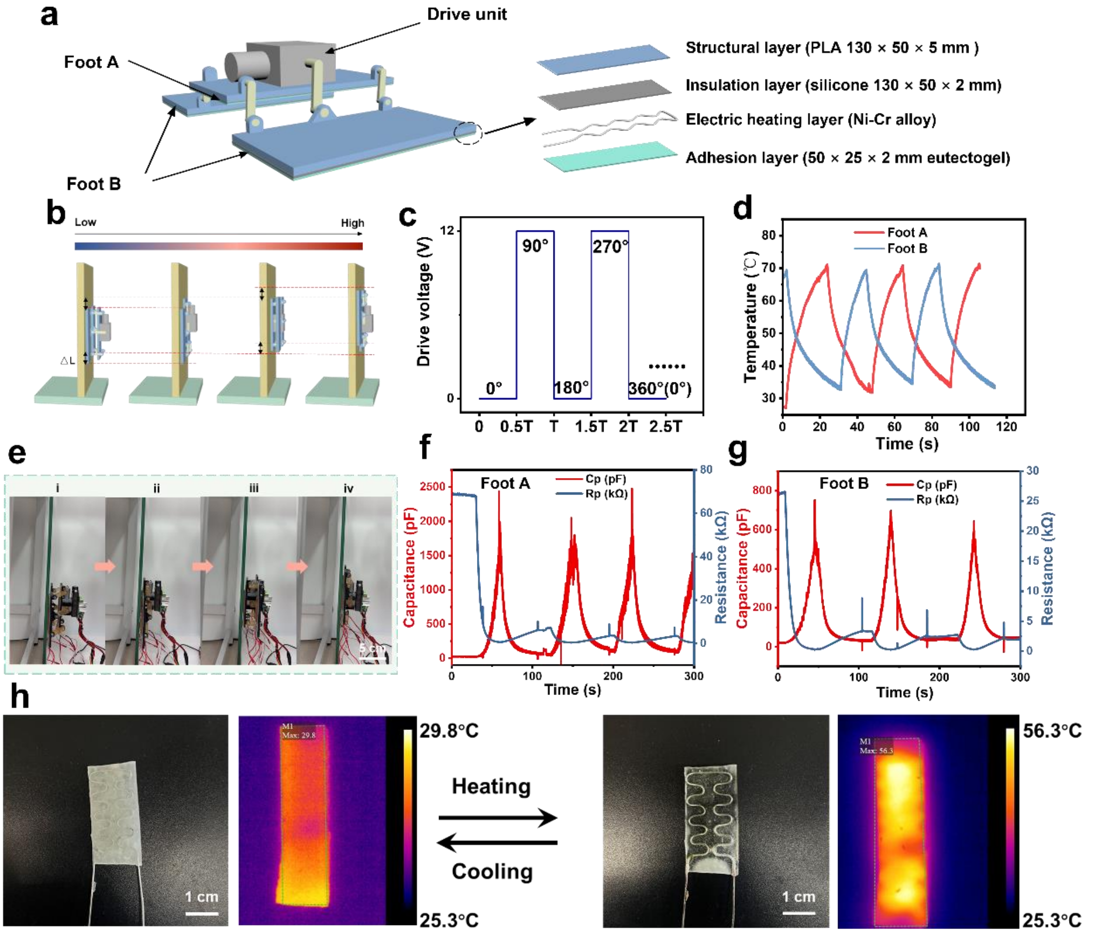

Fig. 6 Design principle and fabrication of the wall-climbing robot. (a) Illustration of the mechanical design of the three-feet wall-climbing robot (left), and layout of the foot including a supporting layer (size: 130 mm × 50 mm × 5 mm), an insulation layer (size: 50 mm × 25 mm × 2 mm), a metallic layer, and the eutectogel ($EG_{0.67-30}$) layer (size: 50 mm × 25 mm × 2 mm).

(b) Schematic diagram of the climbing principle of the wall-climbing robot. The robot climbing motion is realized by human-coordinated control. Over each cycle, the soft robot climbs up by a distance of ΔL. (c) The drive motor rotates the shaft at 12V. Controlling on-off of the drive circuit realizes the intermittent rotation of the dual axes of the gear motor between 0-360°. (d) Temperature curve of eutectogel heating and cooling in robotic feet during three-legged robot crawling. (e) Images of a wall-climbing robot climbing on a 90° glass wall. (f) Electrical signal changes during the heating adhesion-cooling crystallization-heating peeling process of the middle foot (Foot A) of the wall-climbing robot. (g) Electrical signal changes during the heating adhesion-cooling crystallization-heating peeling process of the side foot (Foot B) of a wall-climbing robot. (h) Infrared images probing the heating process of eutectogel with a conductive circuit embedded in the gel ($EG_{0.67\text{-}30}$).

In addition, we also investigated the feasibility of the eutectogel for switchable adhesion in relatively abnormal conditions (high/low environment temperature).

Corresponding revision:

On page 24: We also conducted heavy-load grasping tests at relatively high temperatures (40°C) and low temperatures (-10°C) to verify the external environment's impact on this gripper. Results indicate that the gripper maintains reliable grasping and release capabilities under these conditions (**Supplementary Fig. S53, Movie S5)**.

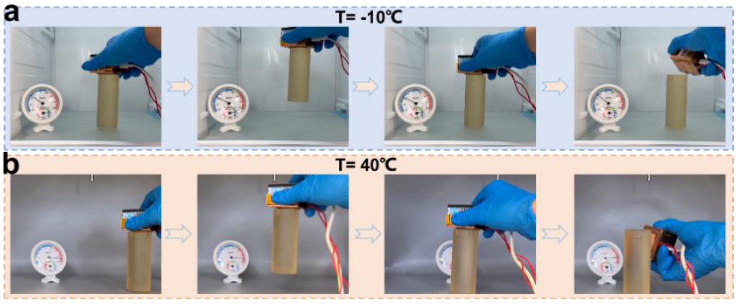

Fig. S53. (a) Eutectogel intelligent gripper grabs digital pictures of target objects at low temperature (-10°C). (b) Eutectogel intelligent gripper grabs digital pictures of target objects at high temperature (40°C).

We would like to sincerely thank you very much for your constructive comments in improving our manuscript.

Reviewer #3 (Remarks to the Author):

I co-reviewed this manuscript with one of the reviewers who provided the listed reports. This is part of the Nature Communications initiative to facilitate training in peer review and to provide appropriate recognition for Early Career Researchers who co-review manuscripts.

Response: Thank you very much for your effort in helping to review our manuscript.

Reviewer #4 (Remarks to the Author)

Comment Authors report an electrothermally switchable adhesion method for gripping and wall climbing. Electrothermally switchable adhesion has been shown before and the novelty the authors present here is their material selection. The material properties have been reported well in detail.

Response: We appreciate the reviewer's positive comments regarding our material selection and characterization. Thank you very much for recognizing the depth of our material analysis.

Comment 1. Authors also claim the sensing ability their material have. It is not clear for me how the proposed material can be used as a conformable gripper if it sandwiched between metal plates? A photograph from the demonstration would be helpful.

Response: Thank you for your valuable suggestions. We cover part of the eutectogel with copper to sense while the exposed part can work as functional unit. During the fabrication process, the copper is made in the same horizontal level with the eutectogel by removing a smart amount of eutectogel. We have supplemented the monitoring with corresponding digital photos and videos as per your suggestion. Furthermore, by monitoring capacitance and resistance signals, we can detect the status of the smart gripper in real time, as well as object contact and separation.

Our revisions to the manuscript are as follows:

On page 25: Additionally, due to the intrinsic ionic conductivity arising from the ionic composition of eutectogels, monitoring of electrical signals enables the tracking of the gripper's own state and the magnitude of contact forces during the object's contact and separation process. **Fig. 5g** and **5h** illustrate the schematic of the gripper detecting electrical signals and the curve of electrical signal changes during the gripping process. Part of the eutectogel is covered with conductive copper, therefore the conductivity/capacitance signal can be probed during gripping. The fluctuations in the electrical signal provide a direct visual representation of the gel's state on the gripper. When contacting and releasing objects, the electrical signal exhibits distinct opposite changes (**Fig. 5h**).

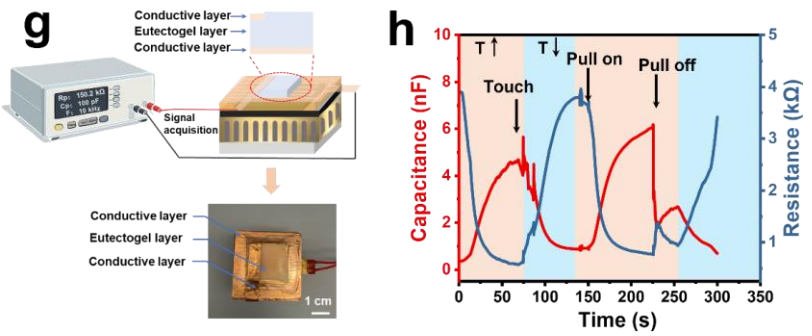

Fig. 5 (g) Underlying mechanism diagram of electrical signals monitored by a digital bridge during the operation of a eutectogel holder. (h) Electrical signal changes during the process of the eutectogel smart gripper grasping target objects.

Comment 2. I understood that the sensing capability is only for sensing if something has been dropped. Would it be possible to use this sensing method for analyzing when the gripper is in contact with the object?

Response: Thank you for your insightful question. We have extended our initially reported real-time detection system to achieve remote monitoring with early-warning capability for adhesive failure. The core advancement lies in reformulating the task from classifying the current adhesion state to predicting whether a failure will occur within the next second.

This fundamental shift required the following key modifications to our methodological approach:

(1) Input data restructuring: from instantaneous snapshot to temporal Sequence

The input was redefined from a single vector of instantaneous capacitance (C_t), resistance (R_t), and environmental temperature (T_t) to a temporal sequence capturing the evolution of these features over the 3-second window (containing 20 samples) immediately preceding the prediction point t. This sequence, [C_{t-20}, R_{t-20}, T_{t-20}, ..., C_{t-1}, R_{t-1}], along with the corresponding ambient temperature profile, allows the model to learn the dynamic precursors of failure.

(2) Model output and training target: from state label to future binary outcome

Consequently, the model's output and training target were changed. Instead of predicting a multi-class state label (e.g., 'fully adhered', 'partially failed') for time t, the model is now trained to perform binary classification for time t+1, predicting a single outcome: "failure within the next sample point" (1) or "no failure" (0).

(3) Training dataset composition: introducing predictive horizons

The training dataset was rebuilt to reflect this predictive aim. Each sample now consists of a 3-second feature sequence paired with the adhesive state label one sample after the sequence ends. This constructs the essential cause (sequence) → effect (future state) relationship for the model to learn.

(4) Evaluation metric emphasis: prioritizing early warning performance

While accuracy remains a measure, our evaluation now places greater emphasis on metrics critical for early warning, particularly the recall (sensitivity) for the "failure" class. Maximizing recall ensures that the system misses as few impending failures as possible, which is the paramount objective of a warning system.

Our revisions to the manuscript are as follows:

On page 22: To facilitate early warning of adhesive failure, we developed an integrated prediction system centered on an STM32 microcontroller. This system leverages onboard Bluetooth for real-time signal transmission to mobile devices (**Supplementary Fig. S48**). Using temperature as an external stimulus, we monitored and recorded the dynamic electrical signal changes of eutectogels during the adhesion and peeling processes across a range of

thermal conditions (**Fig. 4f, Supplementary Fig. S49**). To predict imminent adhesive failure within a one-second horizon, we formulated the challenge as a supervised machine-learning task utilizing a lightweight fully connected neural network (FCN) designed for rapid inference. The FCN was trained on an extensive dataset encompassing the temporal evolution of adhesion and non-adhesion states across 37 distinct temperature settings ranging from -20°C to 80°C. Each training sample is structured as a flattened input vector derived from a three-second temporal window, incorporating time-series features of capacitance, resistance, and ambient temperature. This input is paired with the adhesive state label immediately following the window, thereby establishing a predictive mapping from past signal evolution to the future adhesion state (**Fig. 4g**). The model reached its performance peak, characterized by minimum loss and maximum accuracy, at epoch 200 (**Fig. 4h**). The trained model achieved a robust test-set accuracy of 96.10%, with misclassifications restricted to a small fraction of the extensive 7,407-sample test set (**Fig. 4i**). Crucially for early-warning applications, where the cost of an undetected failure far outweighs that of a false alarm, the model prioritized the minimization of false negatives to yield a high recall rate of 96.39%. This high sensitivity ensures the reliable detection of impending structural compromise, validating that the integration of real-time sensing with machine-learning analysis offers a practical, high-fidelity solution for the remote monitoring of structural adhesive integrity.

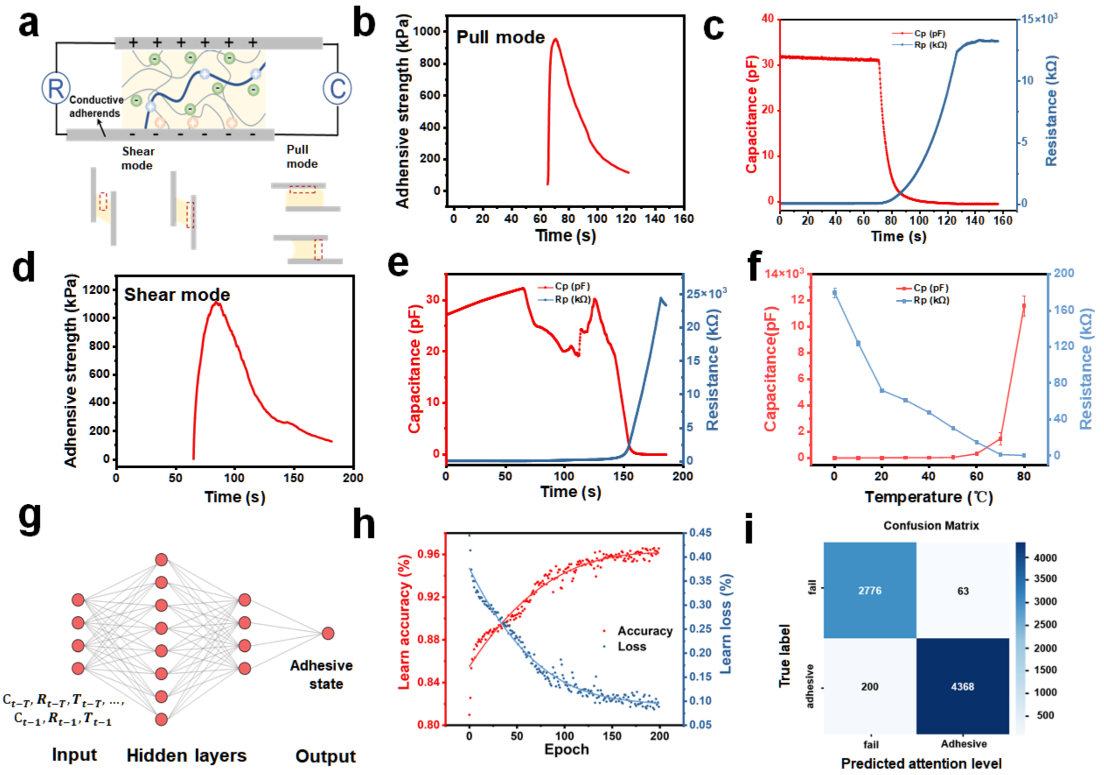

Fig. 4 Intelligent sensing of the adhesive state. (a) Schematic diagram of the intelligent sensing adhesive constructed from eutectogel and two layers of stainless-steel metal sheets. (b) Adhesion force of M2C eutectogel between metallic surfaces in the pull mode. The force on the vertical axis represents the applied force used to simulate the external force required to

change the adhesion state, thereby enabling the monitoring of adhesion state changes. (c) Corresponding change in capacitance/electrical resistance of eutectogel $EG_{0.67-30}$ in the pull mode. (d) Adhesion force of M2C eutectogel between metallic surfaces in the shear mode and (e) corresponding change in capacitance/electrical resistance of eutectogel $EG_{0.67-30}$. (f) The electrical resistance Rp and capacitance Cp values of eutectogel adhesives at different temperatures. Cp gradually increases while Rp decreases with increasing temperature. (g) Machine learning algorithm model diagram. (h) Learning accuracy and F1 score function over 9 epochs. (i) Corresponding prediction confusion matrix using eutectogel $EG_{0.67-30}$.

Comment 3. Because of the soft and conformable gripping surface, I thought the authors would show gripping of different shaped objects (round, sharp, very soft, porous…) and small objects (smaller than gripper diameter) but I did not see this. Is that possible with the suggested gripping method? Also, how long can the gripper hold the heavy objects before dropping them? I.e. What is the long-term behavior of the gripper?

Response: We supplemented experiments demonstrating the eutectogel gripper's ability to grasp objects of varying shapes (rectangular, cylinder, plate, etc), sizes, sharp, porous, and soft round objects.

Corresponding revision is on page 24 and Fig. 5: This temperature-programmed adhesion strategy gripper is widely applicable for grasping objects of varying materials, shapes, and sizes (both larger and smaller than its own diameter). (**Fig. 5e, Supplementary Movie S6**).

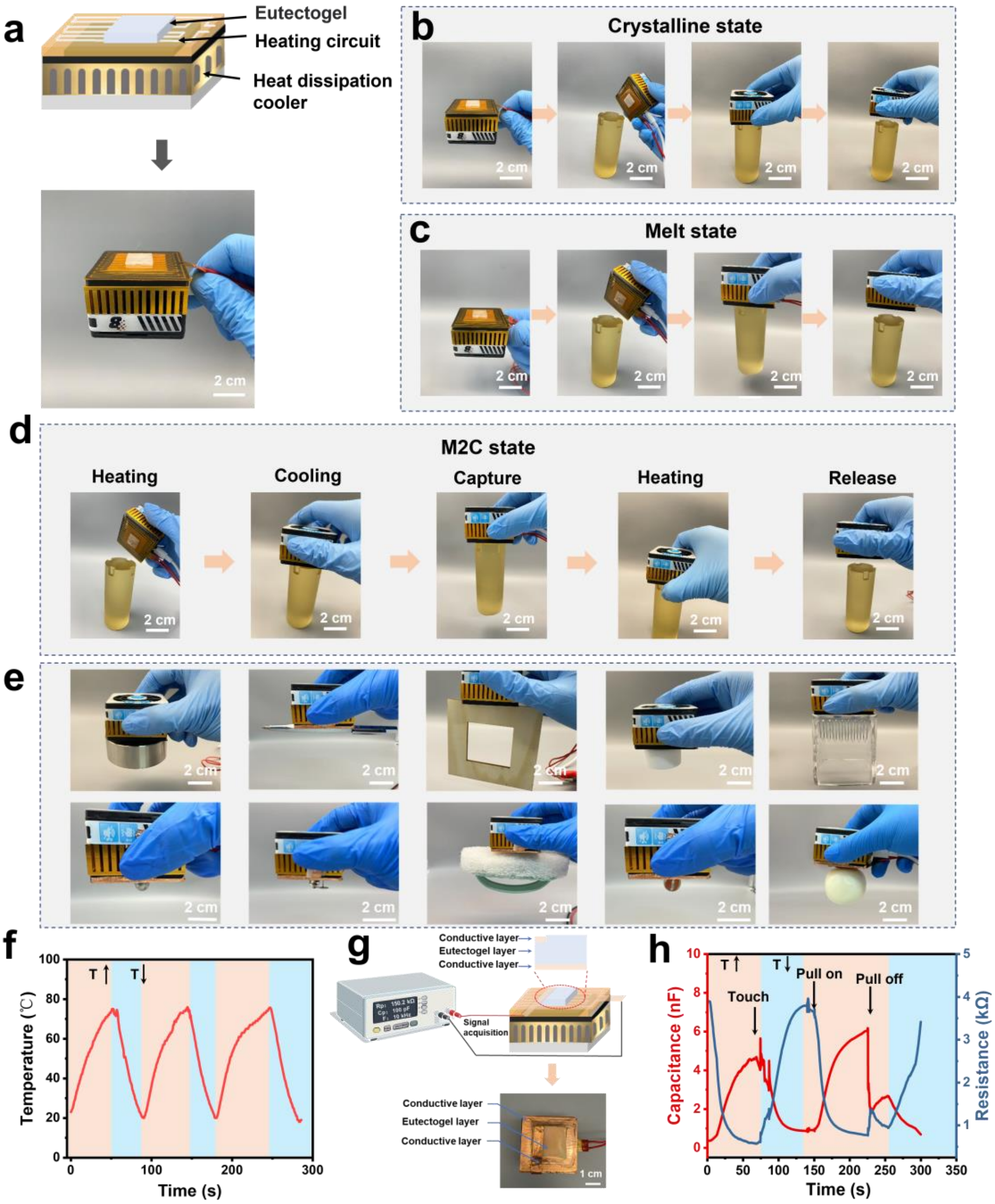

Fig. 5 Development of smart adhesive grippers based on crystalline eutectogels. (a) Schematic diagram of an intelligent adhesive gripper structure based on eutectogel. (b) In the crystalline state, eutectogel smart grippers have difficulty grasping target objects through adhesion. (c) The eutectogel smart gripper in the melt state tends to lose its adhesion to the target object during lifting. (d) Digital photos of the process of picking up target items using eutectogel smart gripper based on the M2C process. (e) The eutectogel ($EG_{0.67\text{-}30}$) smart gripper grasps target objects with diverse surfaces made from various materials, including metal block and sphere, PTFE hollow cylinder, wooden frame, prismatic metal pen, glass cup and glass sphere, metal thumbtack, porous sponge and egg. (f) Temperature curve of eutectogel ($EG_{0.67\text{-}}$

$_{30}$) during heating and cooling in the operation of the eutectogel smart gripper. (g) Underlying mechanism diagram of electrical signals monitored by a digital bridge during the operation of a eutectogel ($EG_{0.67\text{-}30}$) holder. (h) Electrical signal changes during the process of the eutectogel ($EG_{0.67\text{-}30}$) smart gripper grasping target objects.

Experiments revealed that after undergoing the M2C process, the gripper maintained a stable state for extended periods while holding heavy objects.

Our revisions to the manuscript are as follows.

On page 24: To investigate the long-term behavior of grippers when handling heavy objects, we demonstrated the sustained grasping performance of eutectogel in crystalline, molten, and M2C states. Observations revealed that in both crystalline and molten states, the gripper could not hold heavy objects for long periods. However, after undergoing the M2C process, the gripper maintained a stable state for extended periods while holding heavy objects (**Supplementary Fig. S50-52, Movie S4**).

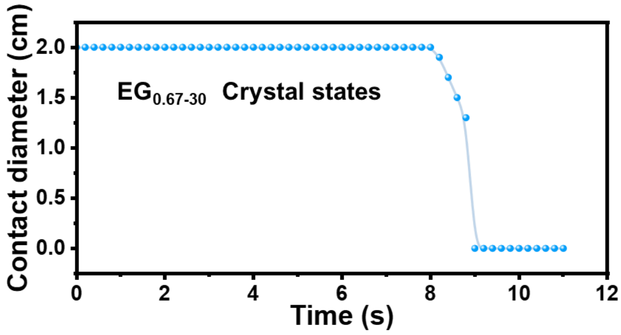

Fig. S50. The behavior of a eutectogel ($EG_{0.67\text{-}30}$) smart gripper when grasping an object (100 g) in the crystalline state.

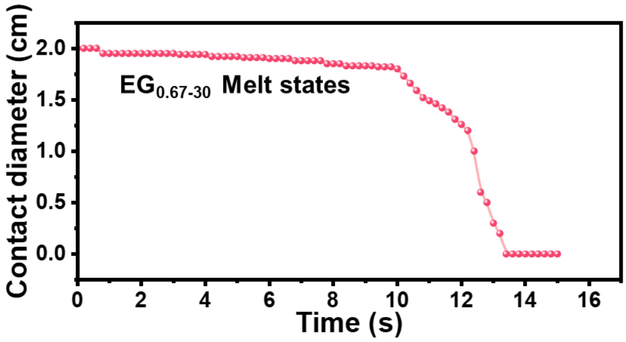

Fig. S51. The behavior of a eutectogel ($EG_{0.67\text{-}30}$) smart gripper when grasping an object (250 g) in the melting state.

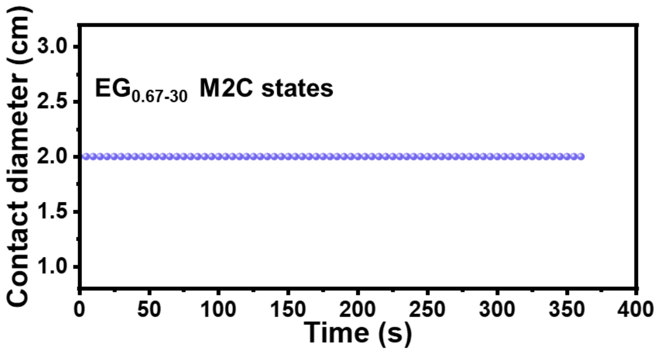

Fig. S52. The behavior of a eutectogel ($EG_{0.67\text{-}30}$) smart gripper when grasping an object (500 g) in the M2C state.

Comment 4. The results are well reported and analyzed but I don't see the significance of these results to the field. If the main novelty is the sensing ability, only sensing the fracture is not impressive. Can different forces be measured with this gripper? How the environment affects to the sensing accuracy?

Response: Thank you for your constructive comment. The novelty of our revised study is that we integrate sensing capability into the switchable adhesion part for various applications, including gripping, climbing robot, and adhesion joint.

While numerous studies have explored switchable adhesion utilizing triggers such as pneumatic pressure, (electro)magnetic fields, light, electric fields, and temperature, these systems often focus on the switching mechanism, rather than integration of adhesion sensing in the process. By capturing and analyzing adhesion states during the dynamic processes of gripping and climbing, our approach can effectively prevent unexpected failures and enable 'smart maintenance', which represent a notable step forward beyond existing studies. In other words, we propose an integration strategy that bridges the gap between adhesion switching and active state awareness. Specifically, apart from a system that can form switchable adhesion with surfaces of various materials and roughness, we demonstrate the system capable of real-time sensing of the adhesion interface while simultaneously retaining the flexibility to reprogram adhesion strength. By providing instantaneous data on the contact status and bond strength, this dual-functionality ensures that robotic adhesion can preemptively detect potential failures (e.g., partial detachment), thereby achieving a level of operational safety and autonomous 'smart' maintenance that was previously unattainable with switchable adhesion alone. This integration is a critical advancement, as it enables real-time monitoring of the adhesive interface, ensuring safer robotic operations and facilitating proactive 'smart' maintenance of the system.

To make it more clear to readers, we have revised the Introduction.

On page 3: Despite these impressive progresses, switchable adhesive surfaces suitable for adherends of versatile materials and roughness remain challenging. Especially, the approach of synergistically combining physical shape locking with reversible chemical interactions, such as hydrogen bonding or ionic bonding, to achieve switchable adhesion remains underexplored. Furthermore, the seamless integration of switchable adhesion and real-time sensing remains a

critical challenge in the development of advanced climbing robots. This integration will be essential for precisely monitoring the interface contact state and adhesion force, thereby preventing catastrophic falls caused by unreliable attachment under nonideal conditions. Such advancements will enable a higher level of bionic intelligence, bridging the gap between artificial systems and the sophisticated sensory-motor functions of natural organisms like geckos.

To overcome these limitations, here we develop crystallizable eutectogels that integrate electrothermally switchable adhesion with intrinsic electrical sensing. Eutectogels are a kind of gels consisting of polymer network with embedded deep eutectic solvents (DES)[51, 52, 53]. Most of them are conductive and have phase transition. The reversible crystallization-melting transition of DES enables a synergistic mechanism of physical shape locking and switchable molecular interactions with adherends, thereby achieving strong and reversible adhesion across diverse surfaces. Upon electrothermal activation, the gel rapidly transitions from a crystalline state (10^1 kPa) to a molten state (10^2 kPa). Through the M2C cooling process, its adhesion properties are tuned between 10^2 kPa and 10^3 kPa, sufficient to support robotic climbing on vertical surfaces with varied materials and roughness. The switching mechanism is elucidated through systematic investigation of the crystalline, molten, and transition states, supported by experimental characterization and molecular simulations. Notably, the inherent conductivity of eutectogels allows real-time monitoring of adhesion states via electrical parameters such as resistance and capacitance, which can be wirelessly transmitted through Bluetooth. Machine learning algorithms further enhance the performance by predicting adhesion states from electrical signals. The deep eutectic solvents used here are also environmentally friendly. The ability of eutectogels to achieve conformal contact in the molten state underpins robust, reversible adhesion on non-specific surfaces, highlighting their potential for intelligent soft robotic systems and adaptive structural applications.

Regarding the question about measuring different forces with gripper, we supplemented relevant experiments for different force sensing (Supplementary Fig. S55). The magnitude of force was determined by measuring the change in pressure-sensitive signals across the gripper's gel layer under fixed loads of varying weights (weights). As the weight increased, the electrical signals also exhibited a proportional change in magnitude. We adhered part of the gripper with copper to probe the change of resistance and capacitance of the system during gripping. These signals respond to the mechanical deformation and temperature-induced conductive changes during the "contact-load-detach" cycle, which allows for state monitoring even on non-conductive substrates. We demonstrated that electrical signals provide distinct, repeatable fluctuations during contact, lifting, and release phases, allowing the system to "know" if an object is securely held.

We made revision in the manuscript:

On page 25: Additionally, due to the intrinsic ionic conductivity arising from the ionic composition of eutectogels, monitoring of electrical signals enables the tracking of the gripper's own state and the magnitude of contact forces during the object's contact and separation process. **Fig. 5g** and **5h** illustrate the schematic of the gripper detecting electrical signals and the curve

of electrical signal changes during the gripping process. Part of the eutectogel is covered with conductive copper, therefore the conductivity/capacitance signal can be probed during gripping. The fluctuations in the electrical signal provide a direct visual representation of the gel's state on the gripper. When contacting and releasing objects, the electrical signal exhibits distinct opposite changes (**Fig. 5h**). Further testing with weights of varying masses generates different pressures to assess the gripper's sensitivity to contact force magnitude. The ratio of electrical signal fluctuations produced by weights ranging from 20 to 500 grams clearly demonstrates an approximate multiplicative relationship between different force magnitudes **(Supplementary Fig. S55).**

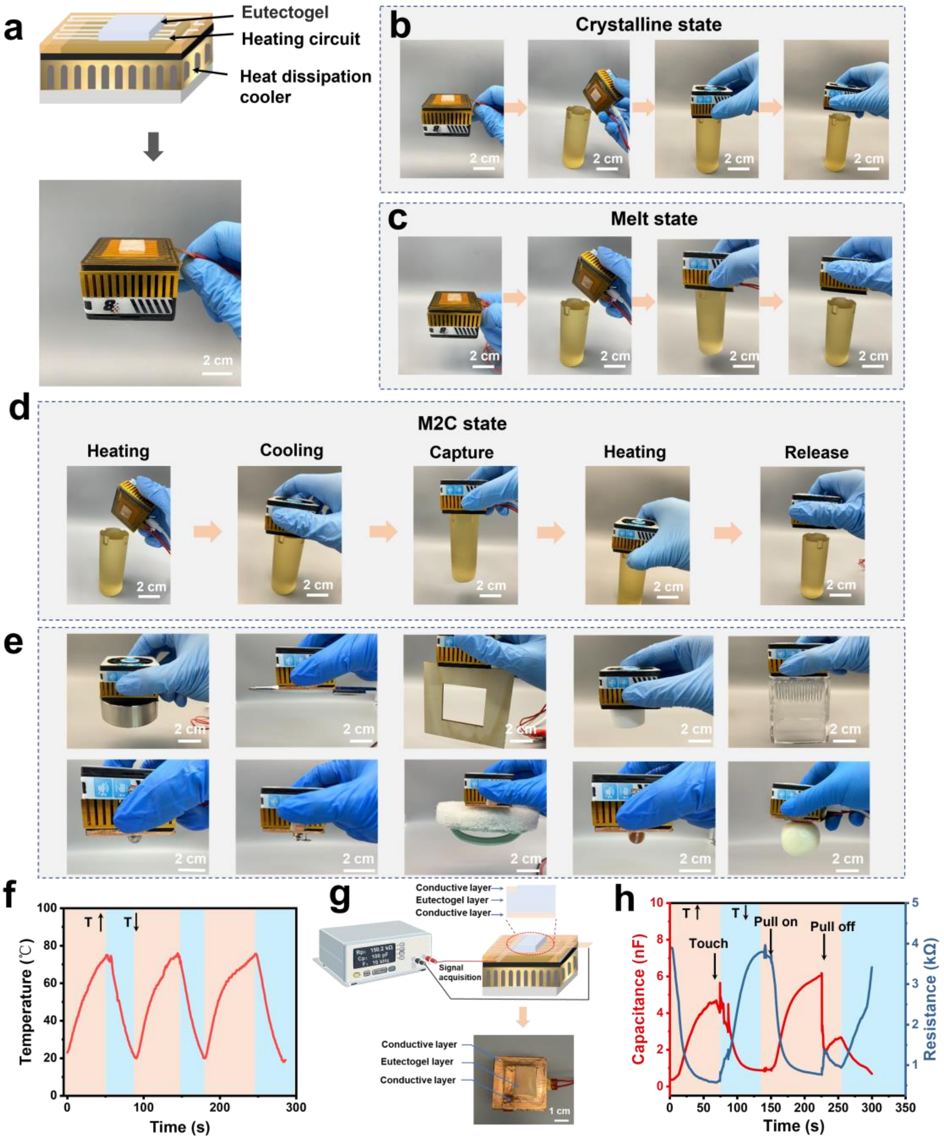

Fig. 5 Development of smart adhesive grippers based on crystalline eutectogels. (a) Schematic diagram of an intelligent adhesive gripper structure based on eutectogel. (b) In the crystalline state, eutectogel smart grippers have difficulty grasping target objects through adhesion. (c) The eutectogel smart gripper in the melt state tends to lose its adhesion to the

target object during lifting. (d) Digital photos of the process of picking up target items using eutectogel smart gripper based on the M2C process. (e) The eutectogel ($EG_{0.67\text{-}30}$) smart gripper grasps target objects with diverse surfaces made from various materials, including metal block and sphere, PTFE hollow cylinder, wooden frame, prismatic metal pen, glass cup and glass sphere, metal thumbtack, porous sponge and egg. (f) Temperature curve of eutectogel ($EG_{0.67\text{-}30}$) during heating and cooling in the operation of the eutectogel smart gripper. (g) Underlying mechanism diagram of electrical signals monitored by a digital bridge during the operation of a eutectogel ($EG_{0.67\text{-}30}$) holder. (h) Electrical signal changes during the process of the eutectogel ($EG_{0.67\text{-}30}$) smart gripper grasping target objects.

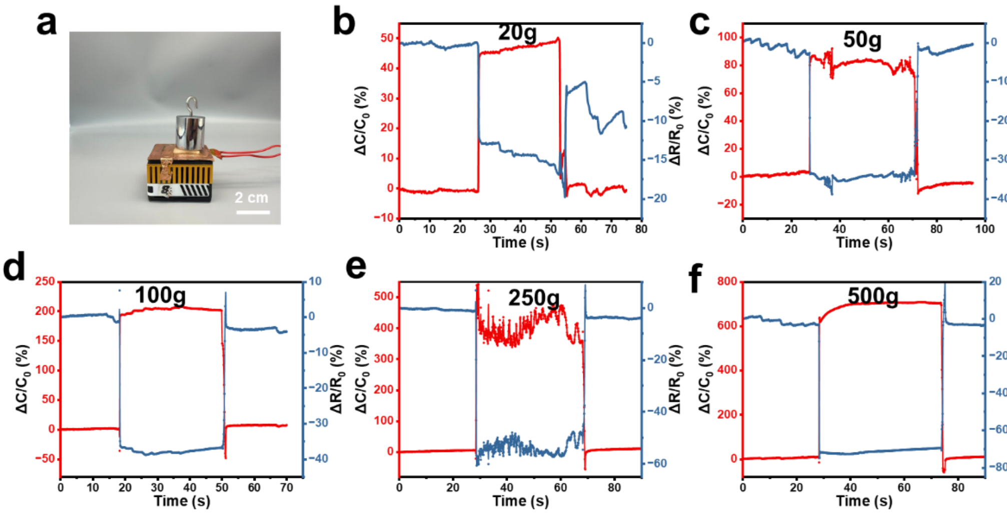

Fig. S55. The eutectogel smart gripper detects the magnitude of pressure exerted by different weights through electrical signals. (a) Digital images of weights placed on the surface of the gripper. (b-f) Electrical signal variation curve generated when 20 g-500 g weights are placed on the eutectogel ($EG_{0.67\text{-}30}$) surface of the gripper.

In addition, we have integrated adhesion sensing into the climbing robot and implemented real-time monitoring during the alternating tripod gait in the revised manuscript. The signal spikes correlate precisely with the contact and separation of the central and lateral legs from the wall, providing the robot with "proprioception" of its adhesion status during locomotion.

We have corresponding revision in the “Development and application of wall-climbing robots” section:

On page 29: The robot demonstrated stable climbing on rough vertical surfaces (**Supplementary Fig. 59**), aided by optimization of the gel heating/cooling balance. The state of the eutectogel in the robot's foot and the robot's crawling posture are monitored via electrical signals. Similar to the gripper section, part of the adhesive pad of climbing robot contacting wall surface is covered with conductive copper, such that the robot’s feet can sense adhesive status in all scenarios not only on conductive surface. As shown in **Fig. 6e and 6f**, when the

middle foot (Foot A) lifts, the eutectogel heats up, causing its resistance to decrease and capacitance to increase. Upon re-contact with the wall surface, the capacitance signal exhibits a marked increase. As the temperature drops, capacitance decreases and resistance increases. When the middle foot detaches from the wall, electrical signal fluctuations occur, characterized by decreased capacitance and increased resistance. This motion is synchronized with the heating and cooling cycles of the electrothermal gel layer (**Fig. 6e, Movie S8**). The heating cycles of the main and lateral feet are maintained in opposite phases: when one foot is lifted, its gel layer is heated to induce melting; when it is loaded, heating ceases and cooling is facilitated via the stainless-steel layer and wall contact.

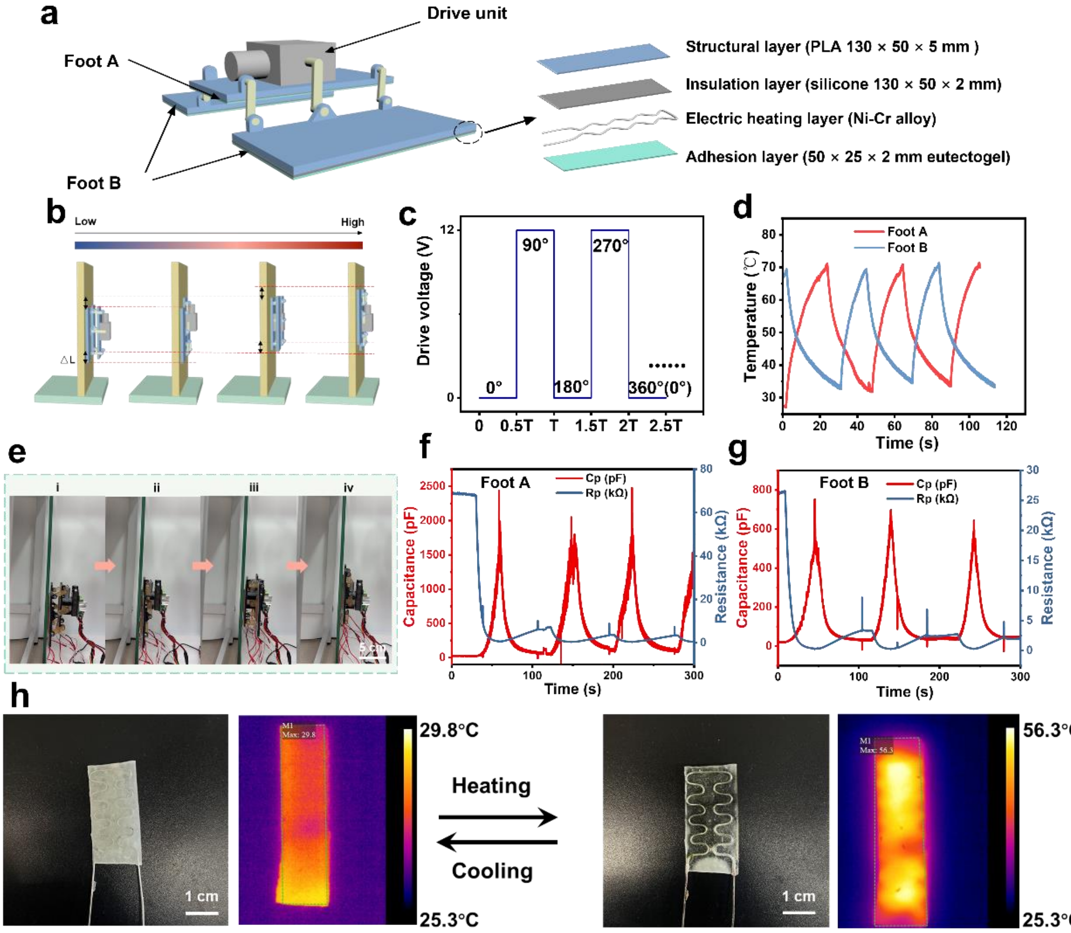

Fig. 6 Design principle and fabrication of the wall-climbing robot. (a) Illustration of the mechanical design of the three-feet wall-climbing robot (left), and layout of the foot including a supporting layer (size: 130 mm × 50 mm × 5 mm), an insulation layer (size: 50 mm × 25 mm × 2 mm), a metallic layer, and the eutectogel ($EG_{0.67\text{-}30}$) layer (size: 50 mm × 25 mm × 2 mm). (b) Schematic diagram of the climbing principle of the wall-climbing robot. The robot climbing motion is realized by human-coordinated control. Over each cycle, the soft robot climbs up by a distance of ΔL. (c) The drive motor rotates the shaft at 12V. Controlling on-off of the drive circuit realizes the intermittent rotation of the dual axes of the gear motor between 0-360°. (d)

Temperature curve of eutectogel heating and cooling in robotic feet during three-legged robot crawling. (e) Images of a wall-climbing robot climbing on a 90° glass wall. (f) Electrical signal changes during the heating adhesion-cooling crystallization-heating peeling process of the middle foot (Foot A) of the wall-climbing robot. (g) Electrical signal changes during the heating adhesion-cooling crystallization-heating peeling process of the side foot (Foot B) of a wall-climbing robot. (h) Infrared images probing the heating process of eutectogel with a conductive circuit embedded in the gel ($EG_{0.67-30}$).

Regarding the influence of environment on the performance of our gripper, we added experiments gripping at high temperature (50°C) and low temperature (-10°C). Corresponding revision in the manuscript on page 24:

We also conducted heavy-load grasping tests at relatively high temperatures (40°C) and low temperatures (-10°C) to verify the external environment's impact on this gripper. Results indicate that the gripper maintains reliable grasping and release capabilities under these conditions (**Supplementary Fig. S53, Movie S5)**.

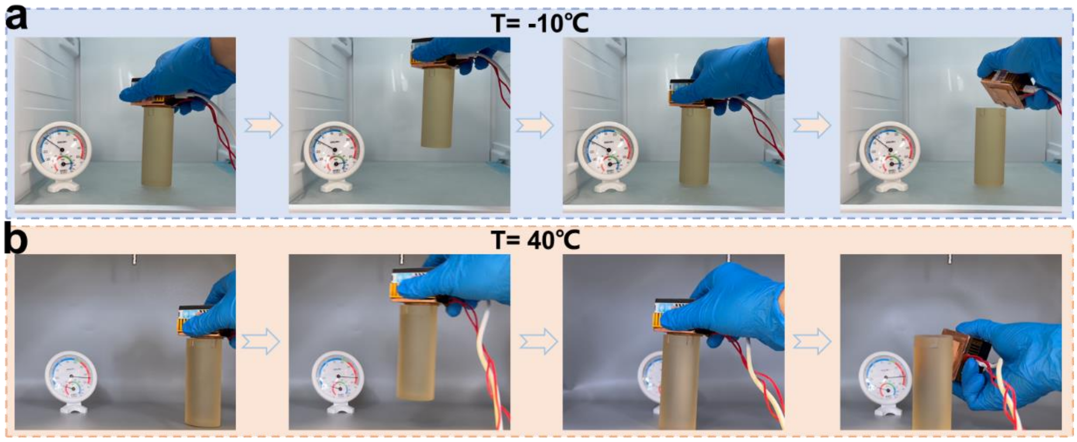

Fig. S53. (a) Eutectogel **(**$EG_{0.67-30}$**)** intelligent gripper grabs digital pictures of target objects at low temperature (-10°C). (b) Eutectogel **(**$EG_{0.67-30}$**)** intelligent gripper grabs digital pictures of target objects at high temperature (40°C).

We believe these additional experiments and the refined discussion effectively demonstrate the synergy of our system, addressing the perceived disconnection between sensing and mobility.

Comment 5. Authors cite some relevant literature in their paper but more detailed comparison table would be useful. How large adhesion forces this gripper can produce compared to similar size different technologies? How much energy does this gripper need compared to other gripping technologies? Some papers to consider for the comparison:

1. S. Song, D. Drotlef, C. Majidi, & M. Sitti, Controllable load sharing for soft adhesive interfaces on three-dimensional surfaces, Proc. Natl. Acad. Sci. U.S.A. 114 (22) E4344-E4353, https://doi.org/10.1073/pnas.1620344114 (2017).

2. Anastasia Koivikko, Dirk-Michael Drotlef, Metin Sitti, Veikko Sariola, Magnetically

switchable soft suction grippers, Extreme Mechanics Letters, Volume 44, 2021,101263, ISSN 2352-4316 https://doi.org/10.1016/j.eml.2021.101263.

Response: Thank you for your valuable suggestions. We have supplemented the manuscript with a recent overview of various crawler types and included comparative charts detailing the performance metrics of the crawler described herein against other crawler types.

Our revisions to the manuscript are as follows:

On page 25: By comparing various types of grippers recently reported across multiple dimensions—including adhesion force, surface adaptability, grasping time, and energy consumption—grippers constructed from eutectogels based on crystalline transformation demonstrated advantages such as high adhesion force, broad adaptability, and low energy consumption **(Supplementary Table S2)**.”

Table S2. Performance Comparison of Adhesion-Based Intelligent Grippers

Gripper type	Preload (N)	Gripper diameter (mm) or area	Max pull -off force (N)	Max lifting ratio	Diameter ratio limits	Surface condition s(surface roughnes s)	Time to acquire grip (s)	Power required to maintain grasp (W)	Reference
Granular jamming	150	86	100	N/A	0.1-0.85	N/A	0.1-1.1	N/A	10
Gecko-inspired gripper	N/A	180	43	200	> 0.5	N/A	<0.1	0	11
Elastomeric microfibrillar soft adhesive gripper	N/A	18	78 mN	N/A	> 1	Need relatively smooth surface	N/A	$4.1\ J/m^2$ $3.3\ J/m^2$	12
Magnetically switchable soft suction	1.5	20	7.5	80	>0.4	17.7	10	0	13
shape memory polymer gripper	N/A	7 – 60 (Depends on the shape and size of the shape memory polymer)	34.8	78.5	< 0.01 > 1	1 μm - 1 cm	N/A Depends on the heating time	0	14
Tensile-induced buckling gripper	65% stretching strain	240 x 40	0.012	N/A	< 0.5	N/A	N/A Depends on the stretching time	N/A The tensile deformation must be maintained.	15
Finger inspired rigid-flexible-soft structure	12	31 cm	4.0	0.11 (Two-finger clamp) 0.128 (Three-finger clamp)	0.806	N/A	3	N/A Depends on the gas actuator power	16
supramolecular gel-elastomer system	0	16 × 16	0.54 (2.12 kPa)	671	< 1	0.01-10	30	N/A 300 V~ 1 kV	17
Curved loop strip soft gripper	0	N/A Depends on the length of the strip		1.7×10^4	< 1	N/A	1.76	N/A	18
Magnetoactive bistable soft gripper	0	N/A	0.029 N	N/A	< 1	N/A	10	N/A	19
Our work	0 – 5 N	2 cm Depends on the length of the eutectogel size	100 N	88.23	> 1	0.01- 1 cm	10 – 20 Depends on the heating time	20V* 0.5 A Depends on the heating time	

Table S4. Recent reports on climbing robots with intelligent adhesion

Climbing robots: adhesion mechanism	Adhesive material	Adhesion Switching Performance	Wall material (Whether it adapts to different surface)	Wall Roughness (Whether suitable for rough surfaces)	Crawling speed	Payload (payload-to-weight ratio)	Energy density	Reference
Pneumatic	Ecoflex	6-8N	glass, sand paper, Acrylics	Smooth and semi-smooth wall (roughness <20 μm)	2.86 cm/min	40g(self mass) 200 g (5 times body weight)	N/A	20
Dielectric elastomer	Dielectric elastomer VHB	3.0533 N (32mm Diameter) 5.09 kPa – 0 kPa	Wood, paper, glass	Exact roughness value unknown, relatively flat.	530.76 cm/min (0.75 body lengths)	10 g	V = 6 kV 23 Hz	21
Electrochemical	Borate ester hydrogels	11.5 kPa - 0 kPa	Multiple materials: glass, metal, ceramics, plastics	Exact roughness value unknown, relatively flat	7.8 cm/min (0.8 body lengths)	175 g (self mass)	V = 3 V 5 s	22
Electro-permanent magnet	Permanent magnet	535.4 N (41*41mm^2) 318.26 kPa-0kPa	Ferromagnetic	Tolerant to rough surface	4200 cm/min (2.12 body lengths)	8 kg (self mass) 2 kg (0.4 body weight)	V = 29.6 V I = 35.18 A 4.07 J	23
Mushroom-shaped dry adhesion and microspike bioadhesive	PDMS and chitosan hydrogel	1 mN (0.3 mm^2) 3.3 kPa-0.33 kPa	Mucus tissue	Relatively smooth, roughness < 30 μm	N/A	20 times body weight, 3 times volume	N/A	24
Growing-climbing mechanism	Microstructured biofilms	7-10 kPa	Multiple materials	Tolerant to rough surface	N/A	521.1g 1330g (1.55 body weight)	N/A	25
Water-enhanced sliding suction mechanism	Water-silicone (capillary forces)	N/A	Polyoxymethylene, PMMA, aluminium alloy	Exact roughness value unknown, relatively flat.	114 cm/min (0.475 body length)	96 g (self mass) 1 kg (10.42 body weight)		26
Electroadhesive	Electroadhesive footpad	3N (4 kV , 39*84 mm^2) 0.92 kPa	Multiple materials	Exact roughness value unknown, relatively flat.	6-90 mm (self length) 24.6 cm/min (2.73 body length)	0.2-3 g(self mass)	V = 3 kV	27
Magnetic-controlled adhesion	magnetically triggered MR elastomers	1.35 N/cm^2-2.93N/cm^2 29.3kPa	Multiple materials: Ecoflex, silicon wafer, pigskin	Exact roughness value unknown, relatively flat.	N/A	N/A	0 T-0.36 T I = 9 A	28
Directional microprismatic structure bioadhesion	PDMS Microstructural friction	<70kPa	Multiple materials	Exact roughness value unknown, relatively flat.	1.4 body length /s (84 body length /min)	0.63g and 0.24g (self mass)		29
Our work	Phase changing eutectogels	Crstal state:10 kPa Melt state: 100kPa M-C state: 1000kPa	Multiple materials, including metals, plastics, ceramics, glass, etc.	Tolerate to rough surfaces, roughness can be up to millimeters.	8cm/min (0.65 body lengths)	500g (self mass) 200g (0.4 body weight)	V = 6 V I = 2 A >10 s	

Regarding the energy cost during gripping, it can be calculated via the voltage and current. The energy cost is around 20 V * 0.5A * 30 s *2 (pick up and release) = 600 J. We have added this into the revise manuscript:

Table S3 shows the control parameters of the eutectogel intelligent gripper in the process of grasping the target object. The energy cost of a pick and release (2 * 30 seconds) cycle can be calculated by the applied voltage and current, which is about 600 J.

Table S3. Control parameters of eutectogel intelligent gripper for grasping target objects.

Gripper compose	Control parameters	
Synthesis information of eutectogel	$EG_{0.67-30}$	HEAA: 30 wt%<br>U/C = 1:1.5
Eutectogel size	25 mm × 25mm × 2 mm	
Heating system	Brand	XinKai electrical appliance PI heating film 001
	Size	50 mm×50 mm
	Operating voltage and current	Maximum power: 24 V, 16 W<br>Actual power: 20 V, 0.5 A
Cooling system	Brand	Ice Lord radiator<br>Silent semiconductor heat sink 008
	Size and weight	50 mm×50 mm<br>100 g
	Operating voltage and current	Voltage: 5 V<br>Current: 2 A/ 3 A
Heating time	15-20 s	
Cooling time	20-30 s	

We also cited the related references that were pointed out by the reviewer in the manuscript.

On page 2: To achieve this, various switchable adhesion for surfaces of specific materials and roughness have been developed[12]: pneumatic/hydraulic control, sometimes combined with gecko-inspired elastomeric microfibrillar adhesive, have been effectively employed on surfaces with minimal roughness[13, 14, 15, 16, 17, 18];…

16. Koivikko A, Drotlef D-M, Sitti M, Sariola V. Magnetically switchable soft suction grippers. *Extreme Mechanics Letters* **44**, 101263 (2021).

17. Song S, Drotlef DM, Majidi C, Sitti M. Controllable load sharing for soft adhesive interfaces on three-dimensional surfaces. *Proceedings of the National Academy of Sciences of the United States of America* **114**, E4344-E4353 (2017).

18. Koivikko A, Drotlef D-M, Dayan CB, Sariola V, Sitti M. 3D-Printed Pneumatically Controlled Soft Suction Cups for Gripping Fragile, Small, and Rough Objects. *Advanced Intelligent Systems* **3**, 2100034 (2021).

Comment 6. Authors claim that the material they propose is sustainable and environmentally friendly, but these claims are not supported in the paper.

Response**:** We appreciate the reviewer's insightful comment. We have now provided specific clarifications to support the claims of sustainability and environmental friendliness in the

revised manuscript.

(1) Sustainability: The sustainability of the material is demonstrated by its recyclability. Due to the reversible physical interactions within the network, the eutectogel can be fully recovered and reused without significant loss of performance.

(2) Environmental friendliness: The deep eutectic solvent (DES) used in this work is composed of urea (non-toxic) and choline chloride (a biocompatible essential nutrient). Compared to traditional organic solvents or toxic monomers, these components present minimal environmental risk and biological toxicity.

Our revisions to the manuscript are as follows:

On page 12: Furthermore, as no covalent crosslinking exists within eutectogels, they can be recovered and reused through suitable solvents such as ethanol. The recovered eutectogel retains its crystallization properties (**Supplementary Fig. S22**). In addition, the DES used in this work is composed of urea (low toxicity) and choline chloride (a biocompatible essential nutrient). Compared to traditional organic solvents or toxic monomers, these components present minimal environmental risk and biological toxicity.

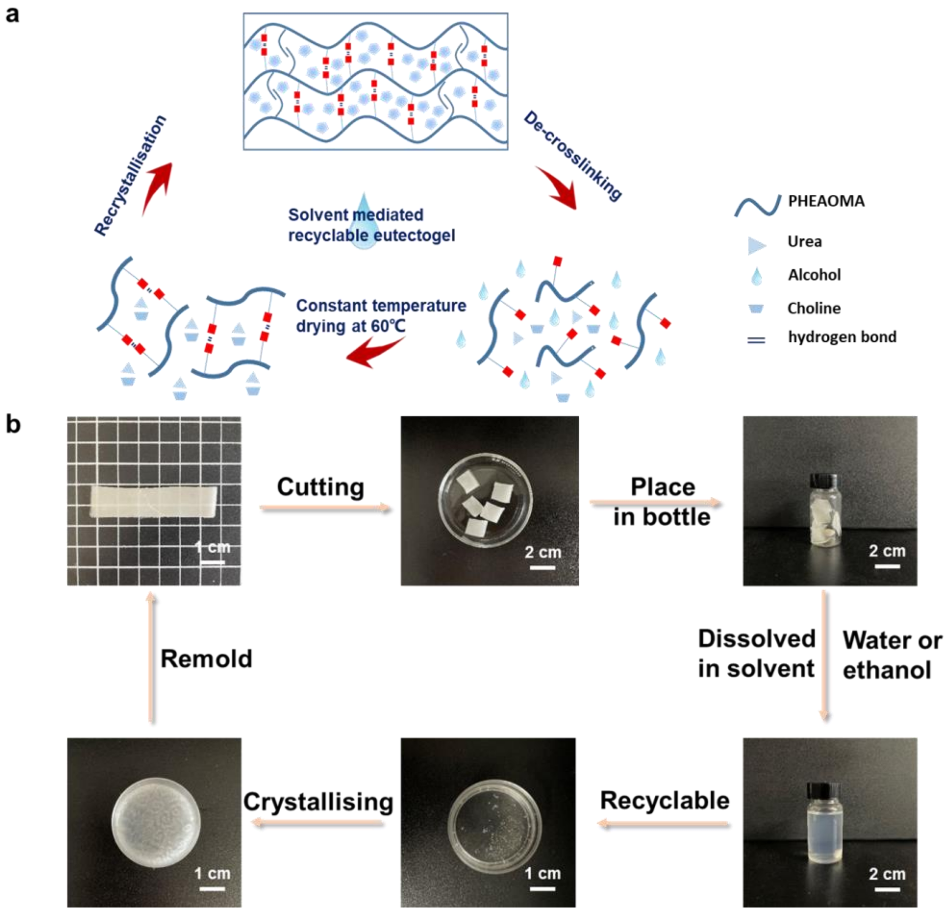

Fig.S22. Recyclability of eutectogels. (a) Schematic diagram of eutectogel recovery process. (b) Digital images of the eutectogel recovery process (sample information: EG_{1-30}).

Comment 7. The paper does not have conclusion section and the discussion is really brief. It is not clear for me how these results compared to other reported gripping and climbing methods (Table was provided but it was more list like no comparison there) in the field and what is the novelty this research brings to the field.

Response: We thank the reviewer for this constructive feedback. We agree that a more profound discussion and conclusion on the comparative advantages of our work was necessary.

Regarding the comparison, we have expanded the Discussion and Conclusion section to explicitly discuss how our eutectogels compare to state-of-the-art technologies (e.g., dry adhesives, phase-change materials, and soft grippers). We have highlighted that our material outperforms many existing solutions in terms of adhesion strength (1700 kPa) and substrate adaptability (rough vs. smooth).

Regarding the novelty: The novelty lies in the integration of a phase-transition-driven "shape-locking" mechanism with intelligent sensing, which is further demonstrated in the gripping and wall climbing process. Unlike previous "list-like" comparisons, we now provide a narrative in the Conclusion that explains why these metrics are superior for practical robotics applications.

Table Enhancement: We have also updated **Supplementary Table S2** in the Supplementary Information to compare representative switchable grippers.

Our revisions to the manuscript are as follows:

Discussion and Conclusion

In summary, we have demonstrated a strategy of (electro)thermally switchable eutectogels that overcome critical limitations of current switchable adhesives. By leveraging a reversible crystalline-to-melt phase transition, these eutectogels achieve robust, shape-locking adhesion across an exceptionally wide range of substrates, regardless of material composition or surface roughness. Compared to existing gripping and climbing technologies, our system offers three distinct advantages. **(1)** High adhesion strength on rough surfaces: While traditional dry adhesives (e.g., gecko-inspired structures) often fail on non-smooth or dusty surfaces, and liquid/gel adhesives lack mechanical stability, our eutectogel achieves an adhesion strength of up to 1700 kPa. This is notably higher than many reported supramolecular gels and stimuli-responsive polymers, particularly on challenging porous or textured substrates. **(2)** Material versatility and environmental tolerance: unlike adhesives limited to specific polymers or metals, the "shape-locking" mechanism allows our material to conform to diverse interfaces, bridging the gap between soft robotics and heavy-duty industrial grippers. **(3)** Integrated intelligence: The synergy between high-strength adhesion and real-time remote monitoring (via conductance/capacitance sensing) provides a closed-loop feedback mechanism for wall-climbing robots that is often absent in purely mechanical gripping systems. These advances not only broaden the scope of applicable adherends but also provide a scalable strategy for developing multifunctional, "smart" adhesive interfaces. Future work will focus on optimizing the switching speed and investigating the long-term durability of the eutectogels under extreme

environmental conditions, further positioning them as a versatile tool for next-generation soft robotics and automated manufacturing.

Table S2. Performance comparison of adhesion-based intelligent grippers

Gripper type	Preload (N)	Gripper diameter (mm) or area	Max pull -off force (N)	Max lifting ratio	Diameter ratio limits	Surface condition s(surface roughnes s)	Time to acquire grip (s)	Power required to maintain grasp (W)	Reference
Granular jamming	150	86	100	N/A	0.1-0.85	N/A	0.1-1.1	N/A	10
Gecko-inspired gripper	N/A	180	43	200	> 0.5	N/A	<0.1	0	11
Elastomeric microfibrillar soft adhesive gripper	N/A	18	78 mN	N/A	> 1	Need relatively smooth surface	N/A	4.1 J/m^2 3.3 J/m^2	12
Magnetically switchable soft suction	1.5	20	7.5	80	>0.4	17.7	10	0	13
shape memory polymer gripper	N/A	7 – 60 (Depends on the shape and size of the shape memory polymer)	34.8	78.5	< 0.01 > 1	1 μm - 1 cm	N/A Depends on the heating time	0	14
Tensile-induced buckling gripper	65% stretching strain	240 x 40	0.012	N/A	< 0.5	N/A	N/A Depends on the stretching time	N/A The tensile deformation must be maintained.	15
Finger inspired rigid-flexible-soft structure	12	31 cm	4.0	0.11 (Two-finger clamp) 0.128 (Three-finger clamp)	0.806	N/A	3	N/A Depends on the gas actuator power	16
supramolecular gel-elastomer system	0	16 × 16	0.54 (2.12 kPa)	671	< 1	0.01-10	30	N/A 300 V~ 1 kV	17
Curved loop strip soft gripper	0	N/A Depends on the length of the strip		1.7×10^4	< 1	N/A	1.76	N/A	18
Magnetoactive bistable soft gripper	0	N/A	0.029 N	N/A	< 1	N/A	10	N/A	19
Our work	0 – 5 N	2 cm Depends on the length of the eutectogel size	100 N	88.23	> 1	0.01- 1 cm	10 – 20 Depends on the heating time	20V* 0.5 A Depends on the heating time	

Comment 8. Methodology and analysis methods seem overall sound and well justified. The sensing experiment in Fig 7 however is not clear. b and c and simultaneous graphs but b dos not have time axis so it is difficult to see when the fracture happens exactly.

Response: Thank you for the positive comment on our methodology and analysis. We have revised and supplemented the relevant experiments on sensing. We have modified the adhesion force-displacement curve to an adhesion force-time curve and standardized the time axis.

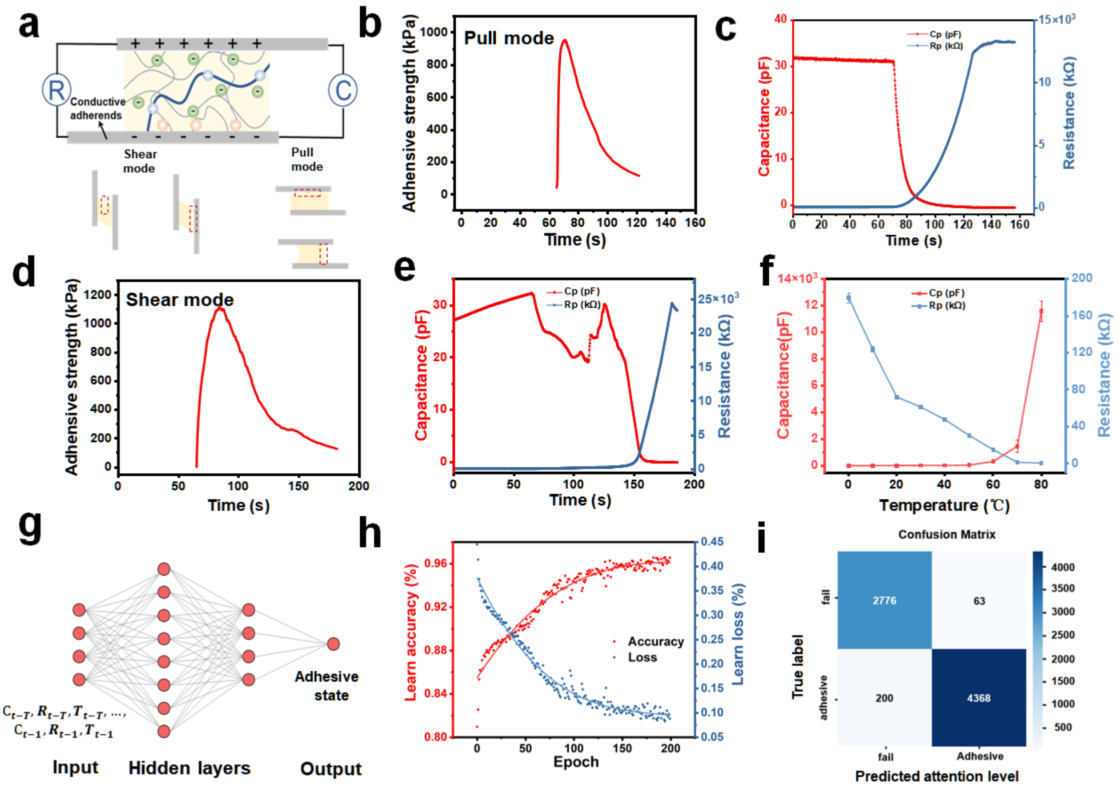

Fig. 4 Intelligent sensing of the adhesive state. (a) Schematic diagram of the intelligent sensing adhesive constructed from eutectogel and two layers of stainless-steel metal sheets. (b) Adhesion force of M2C eutectogel between metallic surfaces in the pull mode. The force on the vertical axis represents the applied force used to simulate the external force required to change the adhesion state, thereby enabling the monitoring of adhesion state changes. (c) Corresponding change in capacitance/electrical resistance of eutectogel $EG_{0.67-30}$ in the pull mode. (d) Adhesion force of M2C eutectogel between metallic surfaces in the shear mode and (e) corresponding change in capacitance/electrical resistance of eutectogel $EG_{0.67-30}$. (f) The electrical resistance Rp and capacitance Cp values of eutectogel adhesives at different temperatures. Cp gradually increases while Rp decreases with increasing temperature. (g) Machine learning algorithm model diagram. (h) Learning accuracy and F1 score function over 9 epochs. (i) Corresponding prediction confusion matrix using eutectogel $EG_{0.67-30}$.

Comment 9. The gripper is electromechanically switchable. How does the surrounding environment changes affect the gripper's performance?

Response: We supplemented gripping experiments at high and low temperatures. The results indicate that external environmental changes have minimal impact on the gripper. As we have active heating and cooling unit in the gripper system, normal environmental temperature variations (-10°C to 40°C) merely influence the performance of the gripper.

Our revisions to the manuscript are as follows:

We also conducted heavy-load grasping tests at relatively high temperatures (40°C) and low temperatures (-10°C) to verify the external environment's impact on this gripper. Results indicate that the gripper maintains reliable grasping and release capabilities under these conditions (**Supplementary Fig. S53, Movie S5)**

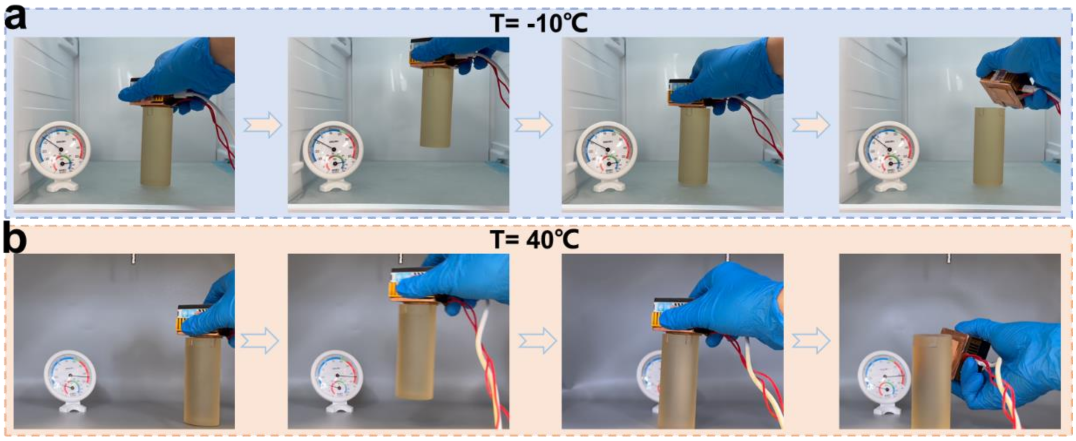

Fig. S53. (a) Eutectogel ($EG_{0.67\text{-}30}$) intelligent gripper grabs digital pictures of target objects at low temperature (-10°C). (b) Eutectogel ($EG_{0.67\text{-}30}$) intelligent gripper grabs digital pictures of target objects at high temperature (40°C).

Minor comments:

What is the x-axis in 3 b and d, same than c and e?

Fig. 4b what are work and forces 0 and 1? Same with 4c

Response: Sorry for the unclarity. The x-axis in Figures 3b and d is consistent with Figures 3c and 3e, and we have relabeled it accordingly.

We have modified the original Figure 4b. In this figure, force represents the peel force, and work denotes the fracture energy of adhesion processes.

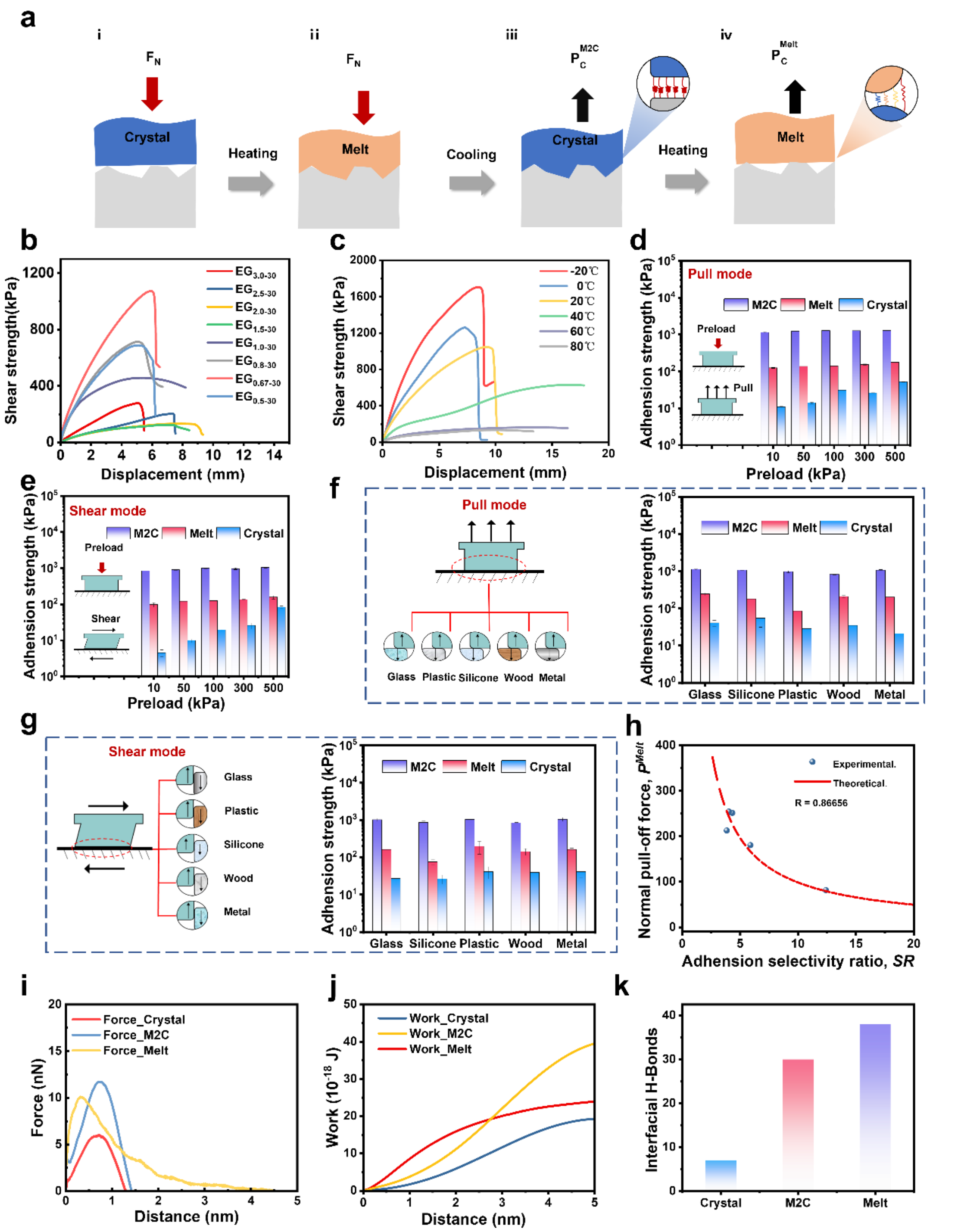

Fig. 3 Mechanism of crystallization-enhanced adhesion in eutectogels. (a) Schematic representation of eutectogel adhesion on rough surfaces in crystal (C), melt (M), and melt-crystal states(M2C). (b) Effect of different solvent ratios on the adhesion properties of eutectogels. (c) Effect of temperature on the adhesion properties of eutectogel $EG_{0.67\text{-}30}$ in shear adhesion test mode. (d-e) Influence of preload on adhesion strength under pull (top) and shear (bottom) modes (measured on smooth glass, aspect ratio = 1). The melt and melt-crystalline states reach adhesion equilibrium at a smaller pre-load pressure (100 kPa), while the crystalline

state is difficult to equilibrate (Sample information: $EG_{0.67-30}$). (f-g) Adhesion strength on various substrates under pull (top) and shear (bottom) modes (aspect ratio = 1 and preload = 200 kPa) of eutectogel $EG_{0.67-30}$. Adhesion characteristics, including high M2C adhesion, low melt adhesion, and minimal crystal adhesion, are consistent across substrates. (h) Molecular dynamics simulations of force conditions (i) and fracture work (j) during adhesion at crystallization, melting, and M2C states for eutectogels. The force required to release adhesion and fracture work during the M2C adhesion process is significantly greater than that required for the Melt and Crystal adhesion processes. (k) The number of interfacial hydrogen bonds formed between eutectogel $EG_{0.67-30}$ and SiO_2 substrate in crystalline, melting, and M2C states (calculated via molecular dynamics simulations).

We would like to thank you very much again for the constructive comments. Hopefully the above revisions can resolve the questions.

Response letter

Dear Reviewers,

Thank you very much for reviewing our manuscript entitled "Dynamic adhesion switching and sensing functionality in crystallization-controlled eutectogel networks" (Research Article, No. NCOMMS-25-79283). We also would like to sincerely thank you all for insightful and helpful comments. Here we respond to comments point by point and have made corresponding revision in the manuscript.

In the following section, the comments of reviewers are in black color, and our response is in blue while the revision in the formal manuscript is in red.

Reviewer #1 (Remarks to the Author):

I thank the authors for their careful and extensive revision and for addressing my earlier concerns in a constructive manner.

At the initial stage, I was not fully convinced that the novelty was sufficiently differentiated from prior studies, as phase-change–enabled switchable adhesion has been widely explored, and the additional use of electrical sensing and machine-learning tools did not immediately appear to provide a clear conceptual distinction. In particular, I initially viewed the machine-learning component as potentially ancillary to the core contribution, and I was uncertain whether it materially strengthened the fundamental advance beyond existing approaches.

However, through the revision process, the authors have clarified the central contribution more effectively. The integration strategy is now presented in a way that aligns well with the demonstrated adhesion performance and the system-level demonstrations, and the manuscript reads as a coherent and well-integrated materials–device–system study rather than a collection of loosely connected elements.

Overall, I am satisfied that the revision has substantially improved the manuscript's clarity and positioning, and I support publication in its current form.

Response: We sincerely appreciate the reviewers' positive comments and previous comments for the improvement of our manuscript.

Reviewer #2 (Remarks to the Author):

The authors have conducted supplementary experiments to answer my concerns. The manuscript was modified well. I have no additional questions.

Response: We sincerely thank the reviewer for the helpful comments which have improved our revised manuscript.

Reviewer #3 (Remarks to the Author):

I co-reviewed this manuscript with one of the reviewers who provided the listed reports. This is part of the Nature Communications initiative to facilitate training in peer review and to provide appropriate recognition for Early Career Researchers who co-review manuscripts.

Response: Thank you very much for your effort in helping to review our manuscript.

Reviewer #4 (Remarks to the Author):

The authors have responded all the questions I raised previously. The authors have increased the significance of their research by focusing on the sensing capabilities of their adhesive, but the work is still not innovative or novel enough. Some of the answers were not sufficient enough. Additionally, after reading the paper draft I still have major concerns listed below:

Response: We sincerely thank the reviewers for their rigorous and critical evaluation of our manuscript. We appreciate the time and effort they devoted to identifying areas where our study required improvement in terms of scientific rigor and data presentation. We take these concerns very seriously and have conducted a comprehensive revision of the manuscript.

1. The whole manuscript is difficult to follow, especially the introduction chapter. The very start of the introduction has long sentences with complicated structure and it feels like the text does not have focus and is a bit all over the place. I recommend the authors would rewrite the draft keeping extra focus in the readability and flow.

Response: We are deeply appreciative of the reviewer's comments regarding the issues in the introduction and the challenges raised regarding the novelty of our work. We have carefully revised the manuscript based on your feedback and incorporated additional content.

We revised the introduction section with the following text.

Strong yet reversible adhesion is essential for many interfacial systems, including climbing robotics[1, 2, 3, 4, 5, 6], transfer printing[7, 8], human–machine interfaces[9, 10], and biomedical engineering[11]. In these applications, the interface must provide sufficient adhesion to support loads while also allowing rapid and controllable detachment. Achieving this capability on surfaces with diverse materials and roughness, however, remains a significant challenge. The fundamental difficulty lies in reconciling two competing requirements: strong interfacial contact that maximizes adhesion and efficient release that enables reversible operation. In addition, practical adhesive systems must adapt to surface irregularities and avoid leaving residues on contacted substrates[12]. These competing requirements make it difficult to realize switchable adhesion that is both strong and universally applicable.

Existing strategies for switchable adhesion mainly rely on three types of mechanisms. One approach uses structural control to regulate contact with the substrate. Examples include pneumatic or hydraulic actuation combined with gecko-inspired elastomeric microfibrillar

adhesives[13, 14, 15, 16, 17, 18], interlocked structures[19], electromagnetic adhesion for ferromagnetic surfaces[3], dielectric elastomer actuators used in miniaturized climbing robots[20], and hierarchical microstructures inspired by gecko footpads[21, 22, 23, 24]. A second strategy utilizes phase transitions to modulate interfacial contact. Shape-memory polymers and liquid crystal elastomers can undergo reversible transitions between rubbery and glassy states[8, 25, 26, 27, 28, 29, 30], enabling conformal contact with rough surfaces followed by detachment through changes in stiffness[31]. Similarly, crystal gels with reversible phase transitions can achieve strong adhesion by forming conformal contact in the soft state and maintaining adhesion in the glassy state[32, 33, 34, 35, 36, 37, 38, 39]. The third approach involves switchable molecular interactions. For example, linear or weakly crosslinked polymers[40, 41, 42, 43] containing photoresponsive groups such as azobenzene can modulate adhesion through light-induced isomerization[44, 45], while cross-linked systems such as thermo-responsive hydrogels have been developed to reduce residue contamination during detachment[46, 47]. Despite these advances, most existing systems rely predominantly on a single mechanism, either physical shape locking or reversible molecular interactions. As a result, their performance often becomes limited when applied to adherends with diverse surface chemistry and roughness.

Besides switchable adhesion, another important feature of biological adhesive systems is the integration of adhesion with sensing and regulation. Many organisms achieve stable locomotion by coupling adhesive contact with sensory feedback and muscular control[48, 49]. Inspired by this concept, Wang, Li and coworkers demonstrated integrated sensing and switchable adhesion during gripping by combining capacitive force sensors with stiffness-tunable dry adhesives or micro-adhesive structures[48, 50]. Nevertheless, most artificial adhesive systems still treat adhesion and sensing as separate components. The lack of intrinsic sensing capability makes it difficult to monitor the interfacial contact state in real time, which is particularly critical for applications such as climbing robots where unreliable attachment may lead to catastrophic failure. Developing adhesive materials that can simultaneously provide strong, switchable adhesion and intrinsic sensing functionality therefore remains an important challenge.

Here we address these challenges by developing crystallizable eutectogels that integrate electrothermally switchable adhesion with intrinsic electrical sensing. Eutectogels are gels composed of a polymer network containing deep eutectic solvents (DES) [51, 52, 53]. These materials are typically conductive and exhibit reversible phase transitions. In our system, the crystallization–melting transition of DES enables a synergistic mechanism that combines physical shape locking with switchable molecular interactions at the interface, allowing strong and reversible adhesion across diverse surfaces. Upon electrothermal activation, the gel rapidly transitions from a crystalline state (10^1 kPa) to a molten state (10^2 kPa). During the M2C cooling process, the adhesion strength can be tuned between 10^2 kPa and 10^3 kPa, which is sufficient to

support robotic climbing on vertical surfaces with varied materials and roughness. The switching mechanism is further elucidated through systematic investigations of the crystalline, molten, and intermediate states using experimental characterization and molecular simulations. Importantly, the intrinsic conductivity of eutectogels enables real-time monitoring of adhesion states through electrical parameters such as resistance and capacitance, which can be wirelessly transmitted via Bluetooth. Machine learning algorithms are further employed to predict adhesion states from electrical signals, enhancing system intelligence and reliability. The deep eutectic solvents used in this work are also environmentally friendly. The ability of eutectogels to achieve conformal contact in the molten state enables robust and reversible adhesion on non-specific surfaces, highlighting their potential for intelligent soft robotic systems and adaptive structural applications.

2. In Fig. 4 b and c, are the x-axes identical and the events happening simultaneously? It is difficult to match the two graphs since they are not in the same picture or on top of each other. Can authors add some labels so the figures are easier to read? Can you present the resistance and capacitance signal as function of time similar to pull experiment but you re not pulling anything? I would like to see if they behave identically and you re actually measuring just temperature.

Response: We are very grateful to the reviewer for pointing out that the presentation of data in Figure 4 of our manuscript was unscientific and unclear. Based on your valuable feedback, we have made the following revisions.

Yes, the events in original Fig. 4 b and c happened simultaneously. To demonstrate the synchrony between electrical and mechanical signals, we plotted them on a shared time axis. The revised Figures 4b and 4c display the force magnitude alongside corresponding electrical changes for both shear and tensile modes. It is evident that when the gel is subjected to a force, the electrical signal undergoes a dramatic change in a synchronous manner. This indicates that the behavior of the gel under mechanical stress can be observed through fluctuations in the electrical signal. To illustrate the resistance variations more clearly, the y-axis of the resistance plot has been converted to a logarithmic scale. The capacitance appears to reach its plateau (or decay) earlier with distance because it is inversely proportional to the distance ($C \propto 1/d$).

Regarding the concern about temperature variations, we apologize for any confusion. During the monitoring of electrical signals under tensile and shear forces, no heating was applied. Consequently, we have moved the plots showing the relationship between temperature, resistance, and capacitance to the Supplementary Information to avoid further misunderstanding.

We have made the following revisions to the manuscript.

On page 21: As shown in **Fig. 4b**, in the tensile mode, the baseline capacitance and

resistance remain stable in the absence of external force. During tensile loading, the capacitance decreases as the interfacial distance increases, and its fluctuations correspond to the formation of interfacial defects. The subsequent increase in resistance indicates that internal gel damage has begun to occur. During the final fracture stage, the capacitance drops sharply while the resistance increases significantly, consistent with the rapid propagation of cracks at the interface between the gel and the adhesive. The fluctuations in the electrical signals and the external force loading behavior throughout the entire process are almost perfectly aligned on the time axis. A similar trend is also observed in the shear mode (**Fig. 4c**).

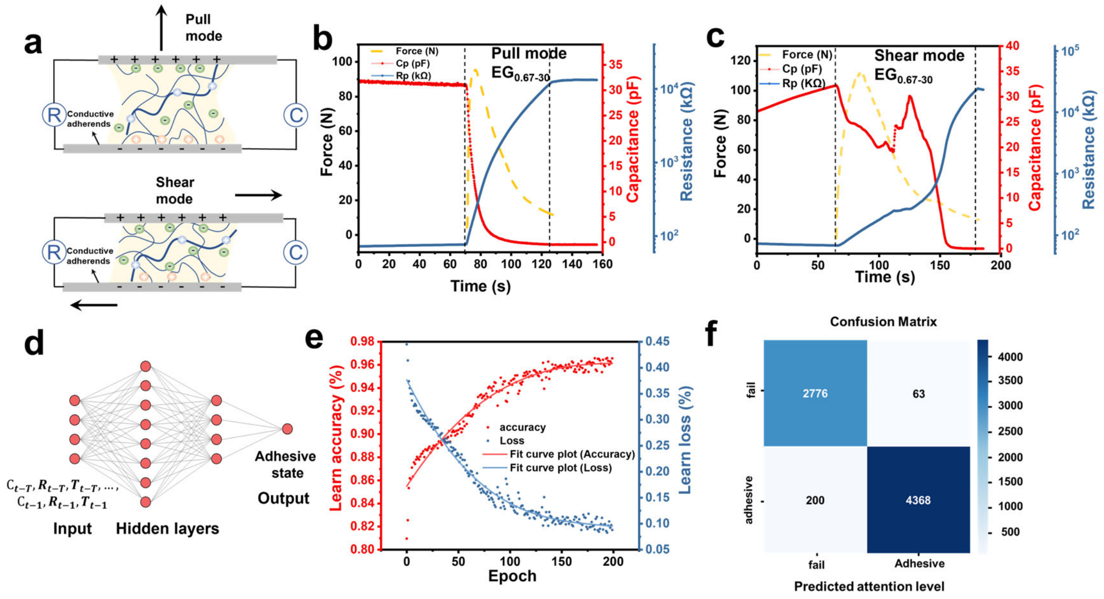

Fig. 4 Intelligent sensing of the adhesive state. (a) Schematic diagram of the intelligent sensing adhesive constructed from eutectogel and two layers of stainless-steel metal sheets. (b) Adhesion of the M2C eutectogel $EG_{0.67\text{-}30}$ to metal surfaces under tensile loading, and the corresponding changes in capacitance and resistance. The force on the vertical axis represents the applied pull force used to simulate the external force required to change the adhesion state, thereby enabling the monitoring of adhesion state changes. (c) Applied shear force on M2C eutectogel between metallic surfaces in shear mode and corresponding change in capacitance/electrical resistance of eutectogel $EG_{0.67\text{-}30}$. (d) Machine learning algorithm model diagram. (e) Learning accuracy and F1 score function over multiple epochs. (f) Corresponding prediction confusion matrix using eutectogel $EG_{0.67\text{-}30}$.

3. The smart gripper experiments need more attention. In the Figure 5h, electrical signals from the gripper are shown. The resistance and capacitance follow the heating cycle quite clearly. How can the information about gripping be extracted here? The resistance and capacitance would behave similarly even if the gripper is not picking anything? Fully decoupled experiment is needed where it can be seen that the sensors are capturing attachment and detachment, not just variations in the temperature.

Response: We sincerely thank the reviewer for the insightful comment regarding signal

decoupling. We fully agree with your point regarding the decoupling of signals related to heating and cooling processes. We have performed a difference calculation on the entire process to isolate and display the signal fluctuations associated with the gripping and release phases of the object.

We have made the following revisions to the manuscript.

On page 25-26: To facilitate a more intuitive comparison between the gripper's grasping/releasing actions and the signal variations, thermal interference from heating and cooling cycles was eliminated via differential calculation. As shown in **Fig. 5f** indicating the electrical signal change decoupled from the temperature variation, when the gripper contacts the target object, the capacitance signal increases significantly, while the corresponding resistance signal decreases. Conversely, when the object is released, capacitance decreases and resistance increases. Further testing with weights of varying masses generates different pressures to assess the gripper's sensitivity to contact force magnitude.

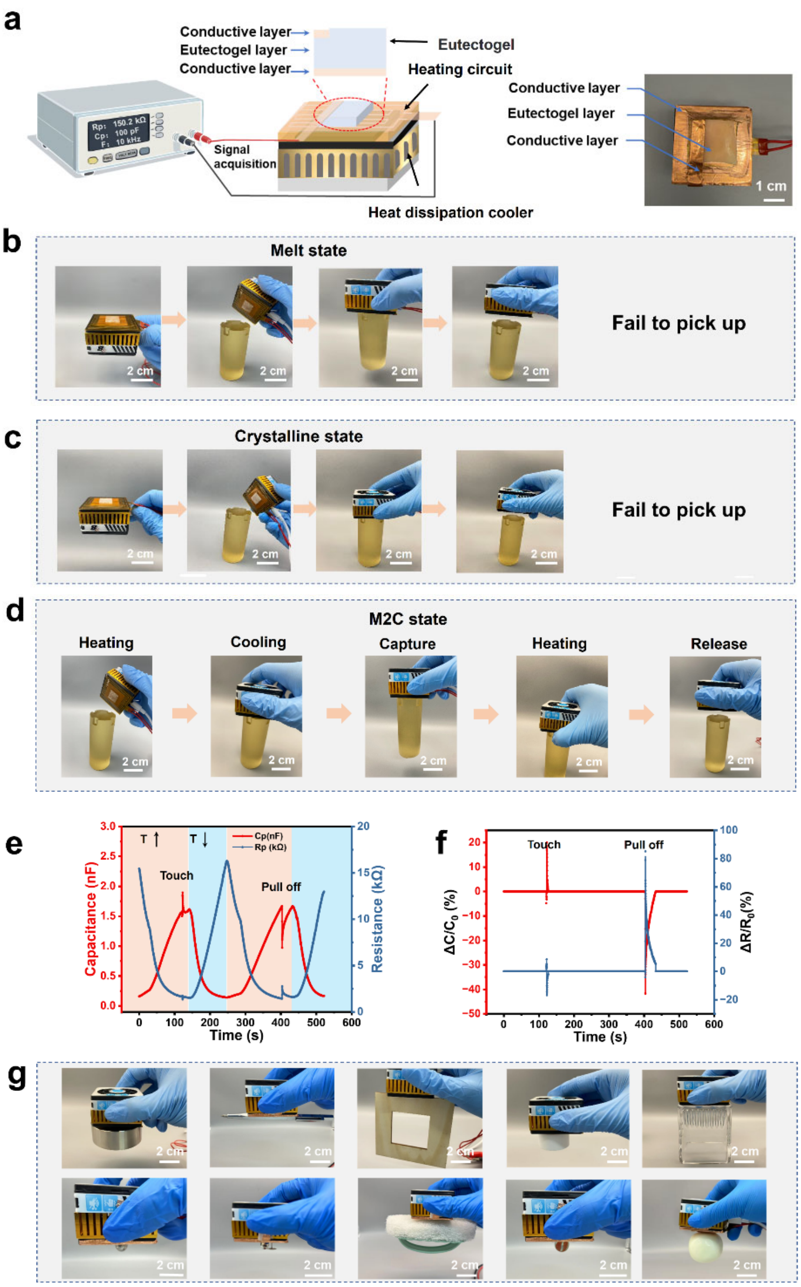

Fig. 5 Development of smart adhesive grippers based on crystalline eutectogels. (a) Schematic diagram and photo of an intelligent adhesive gripper structure based on eutectogel. (b) The eutectogel smart gripper in the melt state tends to lose its adhesion to the target object during lifting. (c) In the crystalline state, eutectogel grippers have difficulty grasping target objects through adhesion. (d) Digital photos of the process of picking up target items using eutectogel ($EG_{0.67\text{-}30}$) smart gripper based on the M2C process. (e) Electrical signal changes during the process of the eutectogel ($EG_{0.67\text{-}30}$) smart gripper grasping target objects. (f) After signal decoupling, the electrical signal shows distinct fluctuation when the intelligent gripper grasps and releases the target object. (g) The eutectogel ($EG_{0.67\text{-}30}$) smart gripper is capable of grasping objects with diverse surfaces made from various materials, including metal block and sphere, PTFE hollow cylinder, wooden frame, prismatic metal pen, glass cup and glass sphere, metal

thumbtack, porous sponge and egg.

4. In Figure 6f and g, it is not clear how the legs are actuated, are the figures f and g corresponding to figures c and d? When are they actually detaching and attaching? Can you mark these events to the figure or even overlap with the control signal.

Response: We apologize for the lack of clarity in the previous version of manuscript. To better illustrate the robot's locomotion and control strategy, we have revised Figure 6b with detailed schematics and supplementary explanatory text. Specifically, we have integrated synchronized time-course data representing the gait control, heating cycles, and adhesion sensing to provide a more comprehensive view of the robot's operational process.

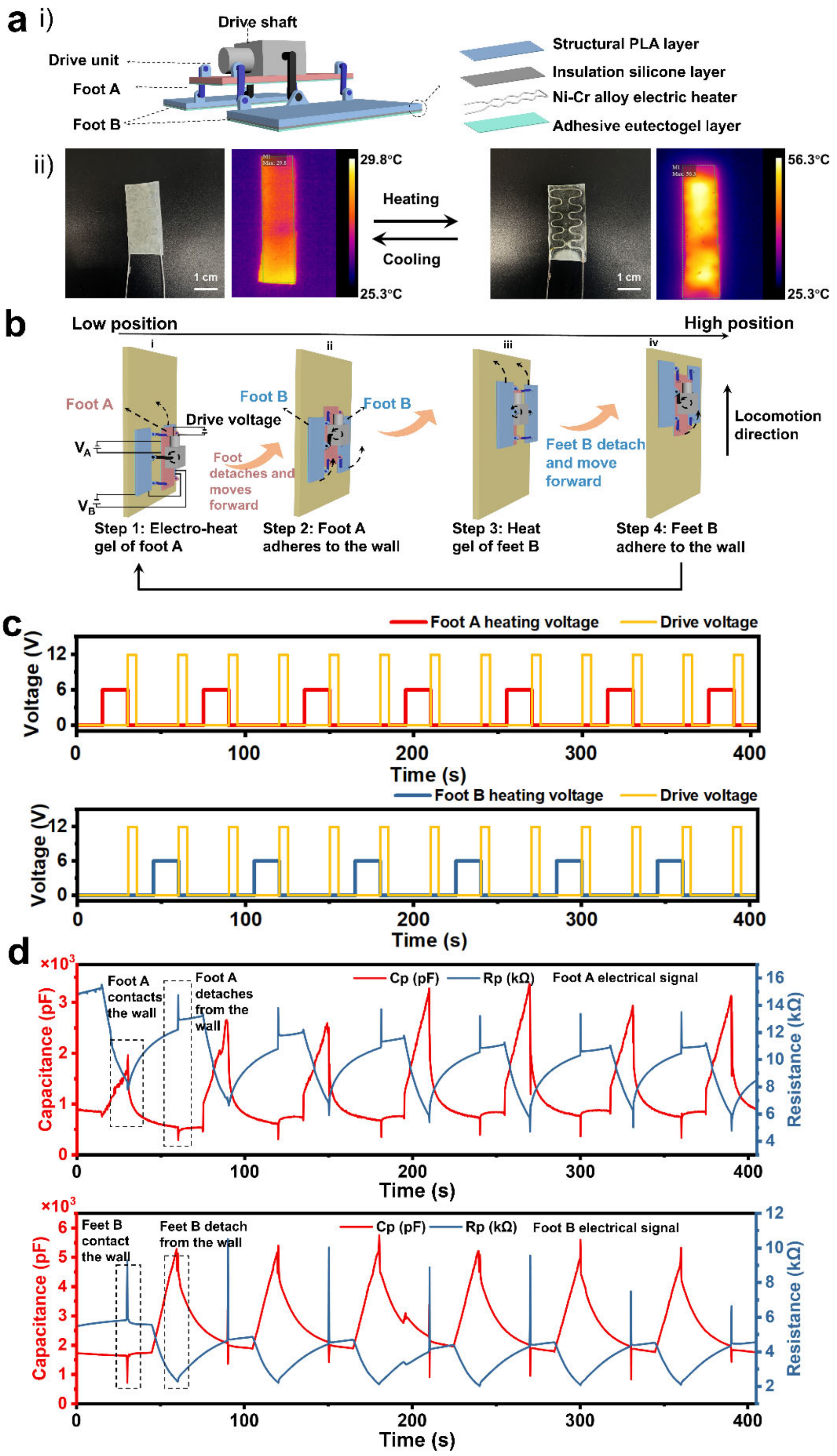

Fig. 6 Design principle and fabrication of the wall-climbing robot. (a) (i) Illustration of the mechanical design of the three-feet wall-climbing robot (left), and layout of the foot including a supporting layer (size: 130 mm × 50 mm × 5 mm), an insulation layer (size: 50 mm × 25 mm × 2 mm), a metallic layer, and the eutectogel ($EG_{0.67\text{-}30}$) layer (size: 50 mm × 25 mm × 2 mm). (ii) Infrared images probing the heating

process of eutectogel with a conductive circuit embedded in the gel ($EG_{0.67-30}$). (b) Schematic diagram of the climbing principle of the wall-climbing robot. The robot climbing motion is realized by human-coordinated control. (c) Control signals for the foot A and feet B of the wall-climbing robot during the heating-adhesion-cooling-crystallization-heating-peeling process, including the heating voltage (6V, 2A) and drive voltage (12V, 3A). The drive voltage is used to drive the motor of shaft while the heating voltage is used to apply on foot A and feet B, respectively. (d) The electrical sensing signals of foot A and feet B change during locomotion upon contacting and detaching from the wall, indicating the real-time status of feet.

We have made the following revisions to the manuscript.

On page 29: The robot climbing mechanism and locomotion sequence are illustrated in Fig. 6b. The movement cycle consists of several distinct steps: a dual-axis gearmotor mounted on Foot A drives the rotary shaft in incremental segments. This segmented rotation, coordinated between the drive and support shafts, causes the main foot (Foot A) and the two auxiliary feet (Foot B) to lift and lower alternately. This motion enables the robot to progress forward (Supplementary Fig. 62). Table S6 lists the control parameters for the eutectogel-based wall-climbing robot during the crawling process. Calculations based on these parameters indicate that the energy consumption per crawling cycle is approximately 1080 J.

On page 29-30: The adhesive state of eutectogel and the robot's crawling posture are monitored via electrical signals. Similar to the gripper section, part of the adhesive pad of climbing robot contacting wall surface is covered with conductive copper, such that the robot's feet can sense adhesive status in all scenarios not only on conductive surface. As shown in **Fig. 6c**, when the middle foot (Foot A) is lifted and the heating switch is activated, the eutectogel heats up to its molten state, causing the resistance to decrease and the capacitance to increase, placing the gel in a viscous state. When the drive switch is activated and the middle foot contacts the wall, the capacitance signal increases significantly. When the heating switch is turned off, the gel temperature decreases, causing the capacitance to decrease and the resistance to increase. When the middle foot detaches from the wall, opposite electrical signal fluctuations occur, manifested as a decrease in capacitance and an increase in resistance. This movement is synchronized with the heating-cooling cycle of the electrothermal gel layer (**Movie S8**). Similar changes also occur in the side feet (Foot B) (**Fig. 6c**). The heating cycles of the main and lateral feet are maintained in opposite phases: when one foot is lifted, its gel layer is heated to induce melting; when it is loaded, heating ceases and cooling is facilitated via the stainless-steel layer and wall contact. The electrical signals fluctuate in response to wall contact (**Fig. 6d**), providing a dual sensing function: detecting initial contact and confirming whether the foot has achieved a secure grip. On walls with inclinations of 30°, 60°, and 90°, each lift-adhere cycle requires approximately 15 s of heating to achieve full melting, and 30 s of cooling for recrystallization (**Movie S9**).

5. In figure S55e, why is the signal so distorted?

Response: We sincerely thank the reviewer for thorough examination and identifying the signal distortion in Figure S55e (now Figure S56). We have repeated the experiment and updated the figure accordingly. Our investigation revealed that the fluctuations occurred when the digital bridge was operated in "fast mode" to test the gel, as the capacitance signal is exceptionally sensitive to the contact area. In that specific sample, the copper foil was not perfectly adhered to the gel surface, leading to interfacial instability and a microscopic gap. This gap rendered the signal susceptible to environmental disturbances, such as cable movement and benchtop vibrations. By refining the sample preparation process to ensure robust contact, we have resolved this issue in the revised manuscript. We are grateful to the reviewer for this insightful observation.

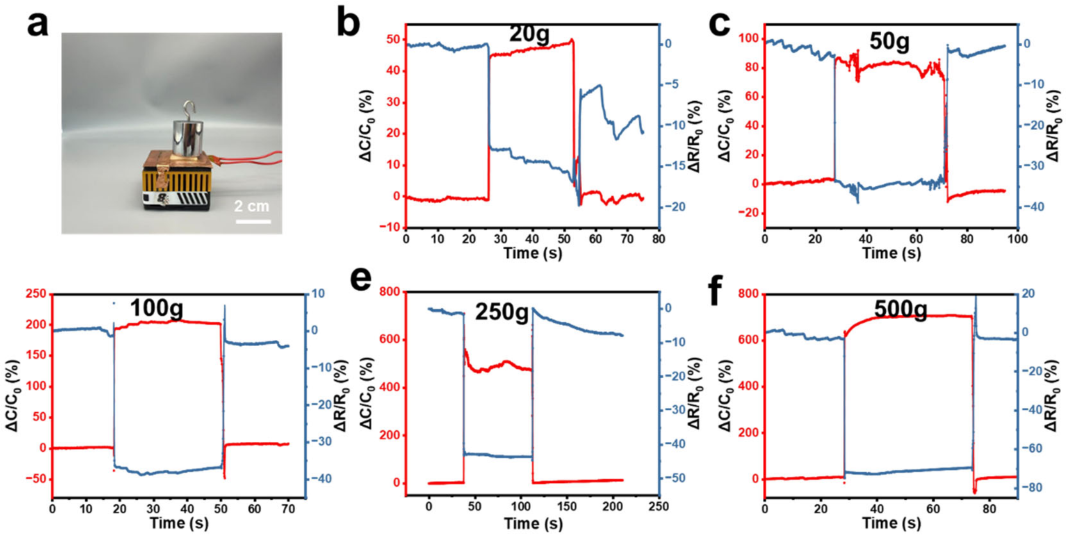

Fig. S56. The eutectogel smart gripper detects the magnitude of pressure exerted by different weights through electrical signals. (a) Digital images of weights placed on the surface of the gripper. (b-f) Electrical signal variation curve generated when 20 g-500 g weights are placed on the eutectogel ($EG_{0.67-30}$) surface of the gripper.

Through these revisions, we hope we have addressed your concerns. We once again express our sincere gratitude for your insightful comments and valuable suggestions.

Other notable revision:

- We have revised the title to "Switchable adhesion of phase-transition eutectogels with integrated machine learning-enhanced intelligent adhesion sensing" to better illustrate the main research topic of this study.
- The affiliation of some authors is updated due to internal re-organization while keeping the author list unchanged.
